# Poster Presentations - Abstracts of the 28^th^ Annual Congress of the SBRA. Florianópolis/SC - Brazil, 2024

**DOI:** 10.5935/1518-0557.20240063

**Published:** 2024

**P-01. Analysis of the metabolome of blastocyst culture medium in relation to oocyte age: a preliminary study**

**Fernanda Souza Peruzzato^1^, Iasmim Lopes de Lima^2^, Luana Teixeira Souza Rodrigues^3^, Caroline Pais Carvalho^2^, Marcos Nogueira Eberlin^2^, Ricardo Nascimento^4^, Jean Louis Maillard^4^, Ana Lucia Bertini Zarth^4^, Marcelo Costa Ferreira^4^, Vanessa Mesquita Prada^4^, Evelise Maria Nazari^5^, Fernando Prado Ferreira^6^, Edson Guimarães Lo Turco^3^, Yara Maria Rauh Muller^5^**

^1^Department of Cell Biology, Embryology and Genetics, Federal University of Santa Catarina and Fecondare - Florianópolis - SC - Brazil

^2^Mackenzie Institute for Research in Graphene and Nanotechnologies - São Paulo - SP - Brazil

^3^Urology Department, Sao Paulo Federal University - São Paulo - SP - Brazil

^4^Fecondare - Florianópolis - SC - Brazil

*^5^Postgraduate Program in Cellular and Developmental Biology, Federal University of Santa Catarina, - Florianópolis - SC - Brazil*

*^6^Neo Vita - São Paulo - SP - Brazil*

**Objective:** The present study aims to investigate whether the metabolome of the blastocyst culture medium from *in vitro* fertilization samples is modified by the age of the oocyte.

**Methods:** A prospective study was carried out with 100 blastocysts from 24 patients undergoing controlled ovarian stimulation and *in vitro* fertilization, separated into three groups according to the age of the oocytes that originated the blastocysts: Group 1 (≤ 35 years, n=50 in a total of 11 patients); Group 2 (36-39 years, n=31 in a total of 7 patients) and Group 3 (≥40 years (n=19 in a total of 6 patients). The culture media of each blastocyst was collected on day 5 or 6 and the secretomics were evaluated by a direct infusion system coupled to an LTQ XLTM Linear Ion Trap mass spectrometer. Multivariate analyses were performed using the MetaboAnalyst v6.0 platform. This study was approved by the National Research Ethics Committee (CONEP) under number 4.910.131.

**Results:** Both multivariate supervised models proved helpful in discriminating among the age groups, showing that there is a clear distinction between Group 1 and Groups 2 and 3, indicating a distinctive separation in the metabolic profile secreted by blastocysts of younger women (≤ 35 years) from those of women older than 36 years. Partial Least Squares-Discriminant Analysis (PLS-DA) and Random Forest (RF) models provided nine equivalent potential predictive high-ranked features for group classification. The ions of m/z 301, 309, 250, 249 and 203 were the main discriminant features for Group 1, while the ions of m/z 238, 314 and 291 were discriminants for Group 2, and m/z value of 308 for Group 3. The exploratory Receiver Operating Characteristic (ROC) multivariate analysis highlighted a putative metabolomic signature of blastocysts, reflecting their substantial diversity among different oocyte`s age groups.

**Conclusion:** The present study suggests that the age of the oocyte would be modifying the blastocyst secretory function.

**P-02. Can the metabolic profile predict the gestation rate of women undergoing *In Vitro* Fertilization? A systematic review**

**Gabriela Bellini de Souza^1^, Mariana F Cornatione^1^, Antonietta B Rosetto^1^, Wanderley M Bernardo^2^, Edson Lo Turco^2^, Luca S Tristão^3^, Guilherme Tavares^3^, Irineu Massais^4^, José M Aldrighi^4^**

*^1^Faculdade de Ciências Médicas da Santa Casa de São Paulo - São Paulo - SP - Brazil*

*^2^Universidade de São Paulo - São Paulo - SP - Brazil*

^3^Centro Universitário Lusíada - Santos - SP - Brazil

^4^Faculdade de Ciências Médicas da Santa Casa de São Paulo - São Paulo - SP - Brazil

**Objective:** Despite the significant increase in the demand for assisted reproduction, most *In Vitro* Fertilization (IVF). cycles do not yield satisfying results. This study aims to conduct a systematic review of the literature concerning the role of metabolomic profiles in predicting pregnancy success among patients undergoing IVF.

**Methods:** This review involved selecting studies from October 2019 to March 2020, retrieved from the EMBASE and MEDLINE databases. The research strategy was structured around specific questions concerning patients, intervention, control, and outcome. The review focused on studies involving women of fertile age undergoing metabolomic analysis during the IVF process. These studies compared metabolite differences between two distinct groups: a pregnancy-success group and a pregnancy-failure group undergoing IVF. The search strategies used were: (fertilization *in vitro* OR *in vitro* fertilizations OR test-tube fertilization OR test-tube fertilizations OR test-tube baby OR test-tube babies OR embryo transfer OR infertility OR IVF OR follicular fluid OR cervical mucus OR endometrium OR endometrial fluid) AND (metabolomic OR metabolomics OR metabonomic OR metabonomics). Studies were excluded if they involved animals, used embryonic material for metabolic analysis, compared IVF success rates based on specific pathologies, or examined intermediate endpoints. The primary outcomes of interest included positive pregnancy (success) and negative pregnancy (failure), as well as the identification of metabolites exhibiting up-regulation and down-regulation in the context of IVF. The results were synthesized using absolute numbers, means, percentages, and variations (standard deviation or confidence interval), with a confidence level set at 95%.

**Results:** During the initial search, 239 studies were found in the MEDLINE database and 698 in the EMBASE database. After removing duplicates, 562 studies remained. Following a title analysis, 76 studies were selected. Subsequently, upon applying inclusion and exclusion criteria, a total of 6 articles were included. A manual and grey literature search added 2 more studies for inclusion. Due to the disparate methodologies of the selected articles-whether in metabolites, extraction techniques, or analysis methods-grouping them for meta-analysis was not feasible, and descriptive approach was chosen. Bias analysis was conducted based on the Joanna Briggs questionnaire for case series. All metabolites with significant outcomes (*p*<0.05) were analyzed, and their findings were unified based on whether they increased or decreased in groups experiencing success or failure in IVF. The metabolites analyzed included amino acids, lipids and their derivatives (steroids, fatty acids, and cholesterol), carbohydrates, and others. In the group experiencing fertilization failure, multiple studies indicated a clear increase in amino acids and their metabolites, such as valine, lysine, ornithine, and threonine. Glucose was found in higher quantities in cases of procedure failure among the analyzed carbohydrates. Regarding lipids and their derivatives, a high concentration of phosphatidic acid, phosphatidylglycerol, and triacylglycerol, which have direct roles in cellular communication and energy regulation, were observed in the successful group. In contrast, lipids associated with apoptosis and cellular differentiation, such as total saturated fatty acids, were more abundant in patients unable to achieve pregnancy.

**Conclusion:** Studies show higher amino acid levels in IVF failure cases, possibly due to reduced energy use and protein synthesis by oocytes. Altered lysine and threonine levels are linked to inflammation and vascular dysfunction, while elevated ornithine suggests reduced Nitric Oxide synthase activity, potentially leading to implantation failure. Higher glucose levels in IVF failure cases need further research to understand their impact on oocyte development. High lipid concentrations may aid implantation and pregnancy maintenance, while lipid-related apoptosis indicates a role in IVF failure. Study limitations include small sample sizes, lack of standardization, and variability in analyzed metabolites. Thus, more multicentric, well-designed longitudinal studies are needed for meaningful metabolic selection and improved clinical applicability in predicting IVF outcomes.

**P-03. High exposure to air pollution during follicular phase of IVF cycles increases aneuploidy rates in human embryos across different chromosomes**

**Paulo Otavio Maluf Perin^1^, Aline Macedo Vaz^1^, Patricia Donadon Souza Aranha^1^, Mariangela Maluf^1^, Paulo Marcelo Perin^1^**

^1^CEERH - Centro Especializado em Reprodução Humana - São Paulo - SP - Brazil

**Objective:** Does exposure to ambient air pollution during the follicular phase increase the aneuploidy rate in human embryos? Previous studies have shown an association between higher levels of air pollution and an increased risk of spontaneous abortion. Some studies have suggested that exposure to ambient air pollutants may induce oxidative stress, systemic inflammation and alterations in DNA methylation and microRNA expression that can lead to pregnancy loss. However, to our knowledge, no studies investigating the association between short-term preconception exposure to ambient air pollution and aneuploidy rates in human embryos, the most common cause of miscarriage, have been performed.

**Methods:** A retrospective cohort study was carried out to assess the impact of short-term exposure to ambient air pollution (particulate matter, PM10µm and PM2.5µm; SO2; CO; NO2; O3) during the follicular phase on embryonic aneuploidy rates of couples undergoing their first IVF cycle for preimplantation genetic screening (n=235). The study was conducted between January 2013 and December 2017. Analysis of all 22 autosomes and both sex chromosomes was performed by next-generation sequencing (NGS). Average concentrations of pollutants for the 15 days predating oocyte retrieval represented the exposure of interest. Exposure risk was divided into two groups in which average concentrations for the pollutants were in the upper (Q4 period) or lower (Q1-Q3 period) quartiles. The effect of maternal age at the time of trophectoderm biopsy on aneuploidy was evaluated by dividing the patients into two groups (<40 and ≥ 40 years old).

**Results:** The estimated means of PM10, PM2.5, SO_2_, NO_2_, O_3_ and CO for Q1-Q3 and Q4 periods were 27.5, 16.4, 2.4, 36.9, 31.3 µg/m^3^ and 0.65 ppm and 42.2, 25.5, 4.5, 48.0, 50.4 µg/m^3^ and 0.94 ppm, respectively. A significant increase (*p*<0.001) in the aneuploidy rate for chromosomes 11, 12, 15, 17, 19, 20, 21 and 22 was observed in the older group. The aneuploidy rate for chromosome 21 was significantly increased by the exposure to higher levels (Q4 period) of PM10, PM2.5, SO2 and NO_2_ (*p*<0.001). Additionally, ambient air pollutants increased aneuploidy rates for chromosomes 11 (PM10, PM2.5, CO; *p*≤0.04), 17 (PM2.5, SO_2_, CO; *p*≤0.02), 19 (PM10, PM2.5, NO_2_; *p*≤0.02), and 22 (NO_2_, O_3_; *p*≤0.04). Interactions between age and air pollutants showed a disproportionate rise of aneuploidy rates in the older group for chromosomes 8 (O_3_; *p*=0.01), 11 (PM10, CO, O_3_; *p*≤0.03), 13 (O_3_; *p*=0.005), 16 (CO; *p*=0.03), 17 (PM2.5, SO_2_, CO; *p*≤0.04), 19 (PM10, PM2.5; *p*≤0.001), and 21 (PM10, PM2.5, SO_2_, NO_2_; *p*≤0.03). The highest rates of chromosome aneuploidy were found for chromosomes 19 (PM10, 20.9%; PM2.5, 19.2%; NO_2_, 19.0%) and 21 (PM10, 25.6%; PM2.5, 25.5%; SO_2_, 26.0%, NO2, 22.4%) in the older group exposed to the Q4 period.

**Conclusion:** Older women exposed to increased concentrations of air pollutants during the IVF cycle follicular phase showed higher aneuploidy rates, particularly for chromosomes 19 and 21. Our study suggests that exposure to high levels of pollutants during the follicular phase increases the risk of aneuploidy in human embryos, particularly in older women. Although more research is needed, this finding could help to explain the increased risk of early pregnancy loss in polluted cities around the world. Limitations, Reasons for Caution: Retrospective study. Exposure assessment based on pollutant levels derived from a network average across city sites may not adequately account for personal exposure. Patients included were those with embryos achieving the blastocyst stage, which were biopsied and genetically tested, limiting the extrapolation of the results to the general population.

**P-04. Time-Lapse parameters related to human embryo ploidy: Investigating the use of Time-Lapse imaging towards non-invasive embryo selection**

**Letícia Saldanha Camargos Aires^1^, Maria Fernanda Araújo Lima^1^, Luiz Carlos Pinheiro Júnior^2^, Íris Cabral^2^, Adelino Amaral Silva^2^, Bruno Ramalho Carvalho^3^**

^1^Faculdade de Ciências da Educação e Saúde do Centro Universitário de Brasília (FACES/CEUB) - Brasília - DF - Brazil

^2^Genesis Centro de Assistência em Reprodução Humana - Brasília - DF - Brazil

^3^Bruno Ramalho Reprodução Humana - Brasília - DF - Brazil

**Objective:** To assess the association between morphokinetic paratmeters of embryo division (st2, t2, t3, t4, t5, t8, tSC) and blastocyst formation (tSB, tB) to embryo ploidy.

**Methods:** Retrospective, observational study, of 84 embryos from 18 couples attempting 25 ICSI cycles, from September 2022 to January 2024, addressed to preimplantation genetic testing for aneuploidies (PGT-A). The mean age of the female partners was 38.83±3.2 years (ranging 22-44 years). A total of 41 embryos were euploid, resulting in a 42.8% global euploidy rate.

**Results:** Among the morphokinetic parameters evaluated, t5, cc3 (t5-t3), t5-t2, s3 (t8-t5) and tB-tSB showed a high association with ploidy status, being shorter for euploid blastocysts. The respective mean times/intervals for euploid and aneuploid embryos were as follows: t5, 44.71±7.58 *vs*. 49.48±6.53 (*p*=0.0038); cc3, 9.52±5.62 *vs*. 13.02±4.77 (*p*=0.0024); t5-t2, 19.74±6.66 *vs*. 23.88±5.58 (*p*=0.0015); s3, 9.95±6.54 *vs*. 7.54±8.91 (*p*=0.0318); and tB-tSB, 7.91±3.44 *vs*. 9.96±4.17 (*p*=0.0233). A moderate predictive ability of these significant parameters to identify euploid embryos was found from the area under ROC curves. Also, initial cytoplasmatic movements prior to the first cell cleavage were annotated; pattern1 (vibration-like movements) (39% *vs*. 55.8%, respectively), pattern 2 (circular wave movements) (41.5% *vs*. 32.5%, respectively), or pattern 0 (the absence of detectable movements) (19.5% *vs*. 11.6%, respectively) were not significantly correlated to ploidy.

**Conclusion:** Morphokinetic parameters present only a moderate ability to predict euploidy, but time-lapse imaging may be a tool for selecting the embryos to be biopsied for PGT-A, since the euploid appear to reach certain cell division (t5, t5-t3, t5-t2, t8-t5) and blastocyst formation (tB-tSB) moments/intervals faster than the aneuploid ones. Further studies are necessary to confirm our results.

**P-05. Analysis of the national system of Human Reproduction treatments in the last fifteen years**

**Aline Gabriele Etur Santos^1^, Luiz Fernando Pina Carvalho^1^, Tatiana Ribeiro Campos Mello^1^**

^1^Universidade de Mogi das Cruzes - Mogi das Cruzes - SP - Brazil

**The authors did not present the abstract**

**P-06. Post-chemotherapy egg freezing in a patient with Hodgkin’s lymphoma, resulting in live birth: Case report**

**Catharina Maria Facciolli Blum^1^, Bruno Ramalho Carvalho^2^, Natália Paes Barbosa Valadares^1^, Maria Eduarda Bonavides Amaral^1^, Iris de Oliveira Cabral^1^, Hitomi Miura Nakagawa^1^, Adelino Amaral Silva^1^**

^1^Genesis - Brasília - DF - Brazil

^2^Bruno Ramalho Reprodução Humana - Brasília - DF - Brazil

**Objective**: To present a case of post-chemotherapy ovarian stimulation conducted in a patient with Hodgkin’s lymphoma (HL), resulting in live birth nine years after oocyte cryopreservation.

**Methods:** 29-year-old patient diagnosed with HL underwent chemotherapy using doxorubicin, bleomycin, vinblastine, and dacarbazine (ABVD) in October 2013. During subsequent months of oncological follow-up, abdominal cancer recurrence was identified. Consequently, she was referred to our clinic for fertility preservation (FP) counseling, attempting oocyte cryopreservation before the second round of chemotherapy and a bone marrow transplant. To facilitate emergency FP, immediate controlled ovarian stimulation in an antagonist protocol was initiated.

**Results:** A total of 8 metaphase II oocytes were retrieved and vitrified. Nine years later, she returned to pursue pregnancy. Intracytoplasmic sperm injection of the 8 thawed oocytes was performed, and 7 eggs were fertilized. Two fresh embryos were transferred on the third day of development, resulting in a normal, healthy singleton pregnancy, and the live birth of a healthy infant at 39 weeks of gestation, by cesarean section.

**Conclusion:** The viability and fertilization potential of oocytes retrieved from patients with prior chemotherapy may be compromised, and they must be warned about higher rates of ovarian stimulation cancellation reported in literature. However, post-chemotherapy patients may do equally well as pre-chemotherapy patients if they reach oocyte retrieval and successful ICSI, evolving with healthy pregnancy and live birth.

**P-07. Sperm DNA fragmentation caused by oxidative stress: A systematic review**

**Letícia Rodrigues Sobrinho^1^, Valéria Cardoso Franco^1^**

^1^Universidade do Vale do Paraíba - São José dos Campos - SP - Brazil

**Objective:** To evaluate the occurrence of sperm DNA fragmentation caused by oxidative stress.

**Methods:** Systematic literature review conducted between June 2005 and September 2022. Eighteen articles were included, focusing on clinical trial cases, evaluating whether excessive oxidative stress can contribute to sperm DNA fragmentation and, thus, result in infertility.

**Results:** Fifteen of the analyzed articles reported that oxidative stress plays a fundamental role in sperm DNA fragmentation, where the release of electrons from sperm mitochondria generates reactive oxygen species (ROS), thus overcoming the limited endogenous antioxidant defenses of the gamete and inducing DNA damage. However, three studies that treated with seminal antioxidants and then analyzed the concentrations in participants' bodies stated that there was no statistically significant relationship between decreasing DNA fragmentation proportion and increasing antioxidants.

**Conclusion:** Investigating sperm DNA damage proves to be an indispensable area of study as it enriches semen evaluation parameters, directly influencing male reproductive health. Furthermore, elucidating the interplay between oxidative stress and sperm genetic material fragmentation assumes a role of paramount relevance for advancing the development of therapeutic strategies aimed at idiopathic infertility.

**P-08. The effect of dydrogesterone-primed ovarian stimulation on pregnancy rates**

**Salomão Nassif Sfeir-Filho^1^, Kamila Naiara Cypriano^1^, Caroline Kroll^1^**

^1^Clínica Fertilizar - Joinville - SC - Brazil

**Objective:** The dydrogesterone (DYD) is an alternative progestin used in progesterone primed ovarian stimulation (PPOS) protocol, for suppressing a premature luteinizing hormone (LH) surge during the follicular phase. The objective of the study was to compare the effects of progestin-primed ovarian stimulation using dydrogesterone and a gonadotropin-releasing hormone (GnRH) antagonist protocol on biochemical and clinical pregnancy rates in freeze-all cycles.

**Methods:** This retrospective case-control study included all IVF cycles performed in a Reproductive Medicine Center in Southern Brazil, between 2022 and 2023. The inclusion criteria were patients using their own and fresh oocytes; undergoing *in vitro* fertilization treatment through the intracytoplasmic sperm injection (ICSI) technique; with freeze-all cycles; with cycles of oocyte pick-up and embryo transfer carried out between 2022 and 2023 realized by the same doctor and same embryologist. The case group included patients using dydrogesterone from the first day of the cycle until the trigger day. The control group included patients receiving a GnRH antagonist. The outcomes analyzed were biochemical pregnancy and clinical pregnancy.

**Results:** A total of 129 patients were included in the study. The biochemical pregnancy rate was higher in DYD-case group (60.5%) compared to the control group (35.5%), with statistically significant results (*p*=0.007). The clinical pregnancy rate was also more prevalent in DYD-case group (39.5%) compared to the control group (26.4%), however without statistically significant result (*p*=0.128). Logistic regression analysis results demonstrated that patients who used dydrogesterone were 2.89 times more likely to have a biochemical pregnancy compared to the control group (95% CI 1.22-6.80; *p*=0.015), regardless the maternal age or the embryo stage (cleavage-stage embryo or blastocyst-stage embryo). There was no significant effect of the use of dydrogesterone compared to the control group for clinical pregnancy (OR=1.65; 95%CI 0.68-3.97; *p*=0.267).

**Conclusion:** The dydrogesterone-primed ovarian stimulation seems to be a positive effect on the biochemical pregnancy rate to the GnRH antagonist protocol for freeze-all cycles. The PPOS protocol can suppress the premature LH surge during follicular phase, with lower cost to the patient, greater safety and easy administration (oral), being an alternative to the use of GnRH antagonists. More studies are needed to validate the effect of dydrogesterone.

**P-09. Association of FSHR, LH, LHR Polymorphisms with Polycystic Ovary Syndrome in patients undergoing in vitro fertilization**

**João Paolo Bilibio^1^, Arivaldo José Conceição Meireles^2^, Martina Cordini^1^, Ana Carolina Martinhago^1^, Leticia Avi^3^, Pânila Longhi Lorenzzoni^1^**

^1^FertiBC - Balneário Camboriú - SC - Brazil

^2^Pronatus - Belém - PA - Brazil

^3^Univali - Balneário Camboriú - SC - Brazil

**Objective:** This paper aimed to assess the correlation between FSHR, LH and LHR polymorphisms, which are related to follicular development, are associate with polycystic ovary syndrome (POS) in patients undergoing *in vitro* fertilization (IVF).

**Methods:** This age-matched case-control study included two or three controls per woman undergoing controlled ovarian stimulation. Controls were women without POS group, and cases were women with POS group. DNA was extracted from peripheral blood and potential associations with gene polymorphisms related to follicular development FSHR (p.Asn680Ser), LH (p.Trp8Arg and p.Ile15Thr), LHR (18insLQ) were analyzed.

**Results:** Sixty-tree patients were included, 44 without POS and 19 with POS. The age (35.9 versus 32.7, *p*=0.001), infertility time (4.1 *versus* 3.8, *p*=0.705), antral follicle count (8.5 *versus* 16.3, *p*<0.001), AMH (1.0 *versus* 3.1, *p*<0.001) were found in No POS Group and POS Group, respectively. The presence of homozygosis mutant of FSHR (Ans680Ser) were 18.2% *versus* 15.8%, *p*=0.848 in No POS Group and POS Group, respectively. The were no presence of homozygosis mutant of LH (Trp8Arga, *p*=0.628) and LH (Ile15Thr, *p*=0.688) in No POS Group and POS Group. The presence of homozygosis mutant of LHR (18insLQ) were 6.8% versus 0%, *p*=0.507 in No POS Group and POS Group, respectively.

**Conclusion:** In this study we evaluated whether PCOS could be associated with FSHR, LH and LHR polymorphisms as studies have already demonstrated that the presence of these polymorphisms has an impact on the ovarian response to IVF. We did not find an association between these polymorphisms and PCOS, which leads us to the need to research other mechanisms in order to understand ovarian function.

**P-10. A step back? Could fresh embryo transfer at D3 be a good strategy for patients under 35 years of age?**

**João Paolo Bilibio^1^, Arivaldo Meireles^2^, Pablo Sales^2^, Waleria Placido^3^, Panila Longhi Lorenzzoni^1^**

^1^FertiBC - Balneário Camboriú - SC - Brazil

^1^Pronatus - Belém - MG - Brazil

^1^UFPA - Belém - PA - Brazil

**Objective:** To evaluate whether, in young patients under 35 years of age, the strategy of returning to fresh embryo transfer on D3 would have the same pregnancy and miscarriage rates as fresh embryo transfer into blastocysts after IVF.

**Methods:** A retrospective cohort study was carried out in women under 35 years of age in which fresh embryos were transferred. 149 women who underwent IVF treatment with an antagonist protocol using r-FSH and recombinant GnRH were included, divided into two transfer groups, group D3 (transfer of 3-day embryos) group D5 (transfer of embryos at the blastocyst stage). Only patients who underwent transfer of just one embryo, either on D3 or D5, were included in the study.

**Results:** The mean age in the D3 group was 31.6 years while in the D5 group it was 32.1 years (p=0.121). The mean time of infertility was 4.1 years for the D3 group and 4.3 years for the D5 group (p=0.776). The average number of MII oocytes collected was 9.8 on D3 and 9.3 on D5 (p=0.810). The pregnancy rate defined with the presence of a gestational sac was 55.1% in the D3 group and 57.1% in the D5 group (p=0.950). The miscarriage rate was 16.3% in the D3 group and 14.5% in the D5 group (p=0.288). No differences were found in the number of antral follicles, FSH, LH and estradiol values on the third day of the cycle, FSH, LH and estradiol values on the hCG trigger day and endometrial thickness between groups D3 versus D5.

**Conclusion:** The transfer of a single fresh embryo at the blastocyst stage in young women under 35 years of age did not increase the pregnancy rate, nor did it decrease the miscarriage rate when compared to the transfer of a single fresh embryo at D3. Therefore, despite advances in culture media, pre-implantation genetic analysis, among others, the strategy of taking a step back and transferring fresh D3 embryos in young patients can be useds.

**P-11. Poor online visibility of the multidisciplinary care team in Brazilian IVF clinics**

**Maria Clara Amaral^1^, Laura Magalhães Amaral^2^, Renata Lima Bossi^1^, Rodrigo Hurtado^1^, Marcos Sampaio^1^, Marcia Mendonça Carneiro^1^**

^1^ ORIGEN Centro de Medicina Reprodutiva - Belo Horizonte - MG - Brazil

^2^ Faculdade Ciências Médicas de Minas Gerais - Belo Horizonte - MG - Brazil

**Objective:** To evaluate the online visibility of the multidisciplinary team in Brazilian IVF clinics registered in the 11^th^ Report of the National Embryo Production System (SisEmbrio, 2017) by searching these clinics websites.

**Methods:** We performed an online cross-sectional analysis on April/2024 on the 11^th^ SisEmbrio registered clinics websites so as to search for the professional representation of the multidisciplinary care team including specialties, the presence of headshots, biographies/CV and any information regarding their work. Availability of care during IVF was also assessed. For our study purposes the multidisciplinary team was composed of: nurses, psychologists, nutritionists, andrologists and geneticists. Institutional review board approval was not required for this study according to Ministério da Saúde (Resolução nº 510 de 07 de abril de 2016 do Conselho de Ética em Pesquisa -COEP). All data presented here were publicly available and did not involve any patient nor protected information.

**Results:** 163 SiSEmbrio-registered clinics websites were evaluated: 141 (86.5%) were private and 9 (5.5%) public but for 13 (7.9%) no such information was available. 108 clinics informed they had a multidisciplinary team while 36 did not and 19 did not carry any information on the topic. As for the composition of the team websites displayed: nurses (n=67; 41.1%), psychologists (n=47; 28.8%), andrologist (n=59; 36.1%), nutritionist (n=21; 12.8%), and geneticists (n=7; 4.2%). Headshots were available for 78 (47.8%) and CV in 65 (39.8%). Online information on the type of work done by nurses was displayed by 4 websites and in 15 for psychologists. Psychological (n=19; 11.6%) and nutritionist (n=8; 4.9%) care during IVF was offered in a small number of clinics. Other types of care offered included physiotherapy (n=4; 2.4%), acupuncture (n=5; 3.0%) and social worker (n=2; 1.2%).

**Conclusion:** Our results show that despite the fact that the majority of the Brazilian IVF clinics websites evaluated informed they had a multidisciplinary team, we found low levels of professional visibility. Nurses and psychologists had the highest visibility whereas nutritionist and geneticists had the lowest. Male factor may account to up to 40% of infertility causes but only 36% of the clinics studied informed they had an andrologist. Psychological and nutrition care is also extremely important but were offered by a small number of Brazilian fertility clinics. Moreover, there was a paucity of information on the type of work done by the multidisciplinary team. IVF is a labor-intensive procedure with a rather stressful patient journey. The presence of a multidisciplinary team may ease patient journey and improve success rates. As a significant proportion of patients nowadays are internet-savvy and often consult fertility clinic websites as their primary source of information, providing adequate information on the type of care offered is important to allow patient informed choice when deciding where to be treated. The healthcare professionals other than the gynecologist involved in fertility care should also receive recognition for their work. Thus, fertility clinics should consider providing online visibility to the multidisciplinary team as this could enhance the recognition of their team, and provide patients with accessible information about the skills and expertise of healthcare professionals involved in the treatment journey.

**P-12. #Infertility in Instagram: A content and reliability analysis in Brazil**

**Debora Alvarenga^1^, Marisa Mendonça Carneiro^2^, Renata Lima Bossi^1^, Tatiana Mattos^1^, Ana Carolina Xavier^1^, Rodrigo Hurtado^1^, Marcos Sampaio^1^, Marcia Mendonça Carneiro^1^**

^1^ORIGEN Centro de Medicina Reprodutiva - Belo Horizonte - MG - Brazil

^1^Faculdade de Letras da Universidade Federal de Minas Gerais - Belo Horizonte - MG - Brazil

**Objective:** We aimed at determining the prevalence, authorship, and reliability of educational fertility-related information shared on Instagram in Brazil using the DISCERN tool.

**Methods:** A list of 11 hashtags consisting of fertility terms commonly used on Instagram was chosen according to https://www.tagsfinder.com/, which included: #infertility, #ivf #endometriosis, #tryingotconceive, #maternity, #humanreproduction, #pregnancy, #invitrofertilization, #assistedreproduction, #pregnant, #difficulttogetpregnant. A new Instagram account, @resmedgob was created to avoid the influence of the platform’s algorithm. Authorship was divided into healthcare professional (HCP) or lay people (LP). Posts were obtained in March 2021 on the most recent 20 ones were included in the analysis. Only educational topics of posts written in Portuguese were evaluated. Educational posts included those aimed at providing education or information on fertility themes such as infertility diagnosis or work up. Video and commercial posts were excluded. Two authors used the DISCERN tool to check the quality and reliability of posts. The DISCERN instrument (http://www.discern.org.uk/discern_instrument.php) comprises 16 questions divided into 3 sections: reliability (1-8); quality of information on treatment choices (9-15) and overall rating (16). Each question was scored from 1 to 5 (1 = no agreement ; 5 = highest agreement). For this study questions 1 to 8 were used: (1) Are the aims clear? (2) Does it achieve its aims? (3) Is it relevant? (4) Is it clear what sources of information were used to compile the publication (other than the author or producer)? (5) Is it clear when the information used or reported in the publication was produced? (6) Is it balanced and unbiased? (7) Does it provide details of additional sources of support and information? (8) Does it refer to areas of uncertainty? Eventual divergences were settled among authors. This study is exempt from ethical approval because it did not include any interaction or intervention with human subjects according to Ministério da Saúde (Resolução nº 510 de 07 de abril de 2016 do Conselho de Ética em Pesquisa -COEP).

**Results:** Thirty-seven posts met inclusion criteria and were analyzed. The majority were authored by healthcare professionals (n=33; 89.2%) 24 of which were doctors, 5 fertility clinics; 4 allied healthcare professionals, one magazine and only 3 were lay people. The majority of posts (n=15) were about fertility treatments; other topics included information about diseases such as endometriosis, exams and diagnosis. Mean Discern analysis for each question was: (1) Are the aims clear? 4.86. (2) Does it achieve its aims? 4.75. (3) Is it relevant? 4.47. (4) Is it clear what sources of information were used to compile the publication (other than the author or producer)? 1.58. (5) Is it clear when the information used or reported in the publication was produced? 1,59. (6.) Is it balanced and unbiased? 1.59. (7) Does it provide details of additional sources of support and information? 1,04. (8) Does it refer to areas of uncertainty? 1.39.

**Conclusion:** Doctors were responsible for the majority of educational infertility-related posts on Instagram. Unfortunately, the reliability of such posts measured by the DISCERN instrument is poor. Although posts clearly state the aim and the relevancy, important issues such as the source of information used to produce the post, details about additional sources of support and information were not available. The posts here analyzed also failed to be balanced and unbiased and worst of all, they did not inform about possible areas of uncertainty. In Brazil, Instagram is a frequent source for health-related information and may offer a great opportunity to educate patients and their families on infertility. The quality of the information provided however should be improved so as to improve reliability. More studies on the accuracy and reliability of infertility-related content posted on social media such as Instagram are warranted.

**P-13. Comparison of fertility care among regions in Brazil - A focus on the country's alarming disparity**

**Marta Ribeiro Hentschke^1^, Júlia Prauchner de Castilhos^2^, Carolina Comissoli Fernandes^2^, Laura Randon Chapochnicoff^2^, Sophia Abur Said^2^, Victória Campos Dornelles^1^, Natália Fontoura Vasconscelos^1^, Alvaro Petracco^1^, Mariangela Badalotti^1^**

^1^Fertilitat - Reproductive Medicine Center - Porto Alegre - RS - Brazil

^2^Pontifical Catholic University of Rio Grande do Sul - Porto Alegre - RS - Brazil

**Objective:** To compare assisted reproduction center’s data among regions of Brazil.

**Methods:** Epidemiological, retrospective, observational study using data extracted from the annual report of SisEmbrio, ANVISA and IBGE, assessed on May, 2024. Data was extracted from 2020-2023 and presented as numbers and percentage (%). Variables related to the population density; income per capita data by region; number of assisted reproductive center (ARC) per region and per million inhabitants; number of IVF cycles per region and per million habitants; variation of cycles/year per region, and the number of embryos transferred in 2023, were considered.

**Results:** Brazil is an extremely large country with a population of 203,080.756, which faces socio economic disparities. Comparing country regions, Southeast has the highest number of population density (84,847.187), followed by the Northeast (54,644.582), South (29,933.315), North (17,349.619) and Midwest (16,287.809). The income per capita data by region are Midwest (2,011), South (1,983), Southeast (1,842), North (1,261) and Northeast (1,053). In 2023, there was 187 ARC: Southeast with highest concentration [105 (56.14%)], South [39 (20.85%)], Northeast [24 (12.83%)], Midwest [14 (7.48%)] and in the North, [5 (2.67%)]. When the number of ARC per million inhabitants was considered, the South presented the most significant number (1.30), followed by the Southeast (1.24), Midwest (0.86), Northeast (0.44) and North (0.29). The total number of IVF cycles in 2023 was also demonstrated: Southeast (35,176), Northeast (9,614), South (7,184), Midwest (3,952); and North (652); and the IVF cycles per million inhabitants were: Southeast (414.6); Midwest (242.6); South (240.0); Northeast (175.9); North (37.6). Regarding to the variation of IVF cycles during the years, the following results were found, considering the South, Southeast, Midwest, Northeast and North regions, respectively: Cycle variation/year, 2020-2021, % (20.13; 30.93; 39.21; 57.16; 7.57); 2021-2022, % (-3.76; -14.94; -8.47; -11.17; -11.06), and 2022-2023, % (-7.25; 1.61; -3.10; 84.03; -7.91). The southeast has the highest number of embryos transferred in 2023 (5,491), followed by the South (2,857); Midwest (963), Northeast (602) and North (207).

**Conclusion:** Brazil, with its vast population diversity, faces significant regional disparities, especially evident in population density and per capita income. The Assisted Reproductive field is also a subject where the disparity is evident. The highest number of ARC and the number of centers/million inhabitants are concentrated in the Southeast and South regions. This distribution is directly related to the region's economic development and may be linked to the lower access to income per capita in the North and Northeast regions, which consequently also have less ARC. Meanwhile, since 2020, the Northeast has shown the highest positive variation among the country's regions, being negative only during the Covid pandemic period, when all regions had a negative variation. The increase in IVF cycles in the northeast in 2023 may be better analyzed. In addition to the difficulty imposed by the high cost of infertility treatment, the scarcity of fertility care in a continental and developing country like Brazil is a significant barrier to equitable infertility treatment. Therefore, there is still a need to intensify efforts to provide equal access to reproductive health care, regardless of socioeconomic status, ethnicity, or geographical location.

**P-14. What is and how to calculate the cumulative ongoing pregnancy rate in an IVF cycle?**

**Carlos Augusto Zarate Nissel^1^, Tatiana C S Bonetti^2^, Patrícia Flórido^1^, Mariana Gomes Fujii^3^, Amanda Turato Barbosa do Amaral^3^, Pedro Augusto Araujo Monteleone^1^**

^1^Centro de Reprodução Humana Governador Mario Covas. Disciplina de Ginecologia. Departamento de Obstetrícia e Ginecologia. Hospital das Clínicas da Faculdade de Medicina da Universidade de São Paulo (HCFMUSP) - São Paulo - SP - Brazil

^2^Departamento de Ginecologia. Escola Paulista de Medicina - Universidade Federal de São Paulo (EPM-UNIFESP) - São Paulo - SP - Brazil

^3^Centro de Reprodução Humana Monteleone - São Paulo - SP - Brazil

**Objective:** One of the greatest desires of patients undergoing assisted reproductive technologies is to know exactly what their chances are of having a child. However, due to the multistage nature of the treatments involved, where patients must pass through a series of milestones for successful treatment, it presents distinctive methods to analyze success. One consequence is that analyses can be carried out using a variety of denominators, excluding participants who have not reached a certain milestone. Hence, success rates may be calculated per cycle started, per egg collection, per transfer, or using some other choice of denominator. Moreover, some interventions, such as preimplantation genetic test for aneuploidy (PGT-A), promise that they could reduce implantation failures and miscarriage, and promote higher pregnancy rates, seeming extremely attractive to patients. Considering all these factors, the real chances of having a child can only be estimated if those calculations are well understood. In this context, the aim of our study was to describe the cumulative ongoing pregnancy rate up to the fourth euploid embryo transfer and discuss the underlying nuances and particularities of these calculations.

**Methods:** This is a retrospective analysis including all patients who underwent single embryo transfers (SET) of post PGT-A euploid embryos, from 2019 to 2023, in a single center in brazil. A total of 348 euploid SET were performed among 257 couples included. The ongoing pregnancy rate was analyzed for each SET and for the cumulative rate.

**Results:** The average age of women at the time of the SET was 37.5±3.3. Most of the transfers were performed in women between 38-40 years (35.9%) and 35-37 years (26.4%). The ongoing pregnancy rates per transfer were 33.8%; 32.5%; 41.2%; 41.7% for the 1^st^ (n=257), 2^nd^ (n=77), 3^rd^ (n=17) and 4^th^ or more (n=12) SETs, respectively (*p*=0.999). To calculate the expected cumulative ongoing pregnancy rate in this population, based on outcomes obtained in our data, we applied the medium pregnancy rate achieved for each transfer, for example, first transfer 33.8% ongoing pregnancies among all patients, then, in the second transfer the rate, 32.5%, was applied on all patients that did not achieved an ongoing pregnancy on the first SET, 100% minus 33.8%, 62.2%, and then subsequently, until the fourth SET. In this calculation the final cumulative pregnancy rate would be approximately 85%. This reflects a hypothetical situation in which all patients had sufficient euploid embryos available and returned for a subsequent SET after an unsuccessful SET, until all available embryos were exhausted. However, when we look for the total ongoing pregnancy rate by couples included in this analysis, this rate was only 46.3%, reflecting the considerable number of patients that did not return for a subsequent transfer after a failure, either because they did not have surplus euploid embryos or because they decided not to return, at least yet. In our data of that period, among patients who failed in the 1st ET and had a surplus embryo, 82.7% (n=77/93) had in fact the 2^nd^ ET; in patients who failed in the 2^nd^ ET and had a surplus embryo, only 47.2% (17/36) had in fact the 3^rd^ ET.

**Conclusion:** Unfortunately, some commonly used denominators do not represent the population, nor statistically established options. Although this estimated success rate may seem extremely attractive, it is important to note that it includes only patients who had the opportunity to reach the transfer, hiding patients who did not have aspirated eggs, expanded blastocysts, had only aneuploid embryos after analysis or had a limited number of euploid embryos available for transfer. Therefore, this desired information by patients may be only a statistical trap and a marketing strategy.

**P-15. Egg freezing in a patient with panhypopituitarism due to total pituitary resection for an epidermoid cyst**

**Flávia Burim Scomparini Xavier^1^, Laura Britz Soares^1^, Laura da Silva Padilha^1^, Gabriela Rachadel Lohn^1^, Victor Quarentei Ciaccio^1^, Giovanna Locks Silvey^1^, Giulia Salvador Martins^1^, Beatriz Fernandes Silva^1^, Dâmaris Assunção Alencar^1^**

^1^Universidade do Sul de Santa Catarina - Palhoça - SC - Brazil

**Objective:** Epidermoid cysts are benign tumors, usually asymptomatic and requiring surgical excision, with panhypopituitarism (PHIP) being a common consequence. PHIP is an endocrine disorder characterized by partial or total deficiency of the pituitary gland, affecting more than one pituitary axis. Consequently, the deficiency of gonadotropic hormones, GH, ACTH, ADH, and thyroid-stimulating hormone during childhood impairs overall organic function and gestational capability. The resulting anovulation and ovarian atrophy due to lack of FSH and LH stimulation make pregnancy rare following complete loss of pituitary function.

**Methods:** Case Report.

**Results:** D.R.B.A.M., 28 years old, nulligravida, diagnosed with PHIP since 2010 following total pituitary resection due to an epidermoid cyst, presenting with Central Hypothyroidism, Diabetes Insipidus, and Hypogonadotropic Hypogonadism. Currently experiencing regular menstrual cycles (induced by the use of ethinylestradiol + dienogest), with a moderate flow and duration of up to 4 days. Currently, in use of Levothyroxine, Prednisone, Vitamin D, Methylcobalamin, and Calcium. Medical attention was sought for primary infertility treatment. Transvaginal ultrasound revealed hypotrophy of both ovaries, with the left ovary being difficult to visualize. Despite this finding, Anti-Mullerian Hormone levels were consistent with the patient's age group (2.33 in May 2023), alongside reduced levels of adenohypophyseal hormones. A thorough investigation of the male partner was conducted, with all tests showing normal results. Regardless of being advised on the possibility of achieving pregnancy through low-complexity treatment post-stimulation with gonadotropins, the couple opted for social egg cryopreservation for future pregnancies. Ovarian stimulation was performed using Pergoveris 300IU, later switched to Gonal 150IU and Menopur 150IU due to slow follicular growth. Ovarian puncture on the 17^th^ day of the cycle resulted in the retrieval of 6 MII oocytes. During a follow-up consultation, a decision was made for another ovarian stimulation and embryo formation, using Gonal and Menopur combined with GH 4IU (growth hormone) therapy. The ovarian stimulation response exhibited a more rapid onset in this instance, with retrieval on the 14^th^ day of the cycle yielding 6 MII oocytes, the same number as without GH use.

Authors Contributions: PHIP arises from various causes, resulting in partial or severe hormonal deficiency in more than one pituitary axis, as described in the case due to the surgical resection of an epidermoid cyst. Pregnancy in this condition is considered rare and high-risk, impacting the hypothalamic-pituitary-ovarian axis and leading to obstetric complications. Advances in human reproduction treatments have increased success rates, although the response to ovarian stimulation remains low. The above case describes a patient with PHIP undergoing fertility preservation through egg freezing and desiring spontaneous pregnancy. It is important to highlight the potential infertility, a condition associated with hormonal disturbances caused by the overall impairment of the pituitary gland and the release of gonadotropic hormones. The use of GH in ovarian stimulation protocols for poor responders has been controversial. Some studies indicate a positive correlation between the oocyte's ability to morphologically progress to a normal embryo and high average GH concentration in the follicular fluid, along with a reduction in the time to achieve conception.

**Conclusion:** These findings correlate with the aforementioned case, where the number of follicles did not change, but the response to stimulation was quicker, proving to be an important tool in fertility treatment. Therefore, it is necessary to emphasize the importance of cryopreservation and fertilization treatments, highlighting the positive impact on the lives of patients and the success of future pregnancies.

**P-16. The influence of autoimmune factors in ovarian reserve and polycystic ovary syndrome**

**João Paolo Bilibio^1^, Ivan Sereno Montenegro^2^, Pablo Sales^1^, Martina Cordini^1^, Leticia Avi^3^, Pânila Longhi Lorenzzoni^1^**

*^1^FertiBC - Balneário Camboriú - SC - Brazil*

^2^HCPA - Porto Alegre - RS - Brazil

^3^Univali - Itajaí - SC - Brazil

**Objective:** The cause(s) of diminished ovarian reserve (DOR) are still unknown. It is unclear whether DOR represents a physiological and individual condition or stems from a pathological condition that causes a greater loss of follicles. A loss of oocytes and fertility potential are associated with exposure to systemic chemotherapy, pelvic irradiation, and genetic abnormalities. On the other hand, polycystic ovary syndrome (PCOS) appears to be the opposite spectrum of diminished ovarian reserve. There is much debate about the influence of autoimmune factors and their influence on DOR. Due to this, the objective of this study was to evaluate the possible association of alloimmune factors in ovarian reserve and polycystic ovary syndrome.

**Methods:** A cross-sectional study was carried out with women between 18 and 35 years of age with an indication for IVF due to tubal factor or male factor. All included participants had ovarian reserve assessment with measurement of FSH and estradiol, anti-Mullerian hormone and antral follicle count on the third day of the menstrual cycle.

The patients were then divided into 3 groups:

- Decreased ovarian reserve (DOR) group (antral follicle count less than 5) n=18.

- Normal ovarian reserve (NOR) group n=45 in which all ovarian reserve parameters were normal (FSH, HAM and CFA).

- Polycystic ovary syndrome (PCOS) group n=34, who met the criteria for PCOS.

All of them collected the following tests to evaluate autoimmune factors: antinuclear factor (ANA), anti-DNA antibody, anti-thyroperoperoxidase antibody (TPO) and anti-thyroglobulin (anti-Tg).

**Results**: The mean age was similar in the three groups: DOR 33.0±3.1, NOR 33.3±2.0 and PCOS 32.2±2.7 years, *p*=0.376. The antral follicle count was: DOR 4.1, NOR 14.1 and PCOS 25,8 groups were different *p*<0.001. The presence of antinuclear factor reactive: DOR 11.1%, NOR 6.6% and PCOS 8.5%, *p*=0.943; Anti-DNA Reactive: DOR 0%, NOR 2.2% and PCOS 0%, *p*=0.943; and TSH elevated (>5.0 um/L) DOR 5.5%, NOR 8.8% and PCOS 11.8%, *p*=0.915 were similar between the tree groups. The presence of Anti-Thyroglobulin Reactive % (>35 UI/mL) was grater in DOR 44.4% versus NOR 13.3% and PCOS 14.2%, p=0.041. The OR DOR X NOR 5.2 (1.4-18.4), *p*=0.010 and OR DOR X PCOS 5.6 (1.4-20.9), *p*=0.010. The presence of Antithyroperoxidase Reactive (>30 U/ml) was grater in DOR 55.5% versus NOR 24.4% and PCOS 17.1%, *p*=0.028. The OR DOR X NOR 3.4(1.1 - 10.6), *p*=0.032 and OR DOR X PCOS 5.3 (1.5 - 18.9), *p*=0.008. The presence of TRAB Reactive (>0.55 UI/L) was grater in DOR 50.0% *versus* NOR 14.3% and PCOS 25.7%, *p*=0.035. The DOR X NOR 8.0 (2.1-29.6), *p*=0.001 and DOR X PCOS 2.8 (0.8 - 9.4), *p*=0.081.

**Conclusion:** We found an association between the presence of autoimmune markers and PAIN. Probably the presence of active markers of Anti-Thyroglobulin, Antithyroperoxidase and TRAB, even with TSH levels within normal parameters, negatively influence ovarian reserve. We found no association between autoimmune markers and PCOS. Patients with the presence of autoimmune factors should be alerted when there is a possible decrease in ovarian reserve at an earlier stage.

**P-17. AMH and AMH-R2 polymorphisms are associated with polycystic ovary syndrome?**

**João Paolo Bilibio^1^, Arivaldo Meireles^2^, Fabio Costa do Nascimento^2^, Pânila Longhi Lorenzzoni^1^**

^1^FertiBC - Balneário Camboriú - SC - Brazil

^2^Pronatus - Belém - PA - Brazil

**Objective:** Polycystic Ovary Syndrome (PCOS) is a complex genetic disorder in reproductive-aged women which is associated with comorbidities of reproductive, metabolic, cardiovascular, endocrine, and psychological nature. Pathogenesis of PCOS involves strong interaction between environmental and genetic factors. Many Single-Nucleotide Polymorphisms (SNPs) have been associated with PCOS in different populations. AMH (Ile49Ser) and AMHR polymorphisms (482A>G) have been associated with variations in estradiol levels and may modulate FSH sensitivity. This paper aimed to assess the correlation between AMH and AMH-R2 polymorphisms are associate with polycystic ovary syndrome (PCOS) in patients undergoing *in vitro* fertilization (IVF).

**Methods:** This age-matched case-control study included two or three controls per woman undergoing controlled ovarian stimulation. Controls were women without PCOS (control group), and cases were women with PCOS group. DNA was extracted from peripheral blood and potential associations with gene polymorphisms related to follicular development AMH (Ile49Ser) and AMHR polymorphisms (482A>G) were analyzed.

**Results:** Sixty-tree patients were included, 44 without PCOS and 19 with PCOS. Evaluating whether the presence of the AMH polymorphism (Ile49Ser) could influence serum AMH levels, we found no difference between the groups. On the other hand, the presence of the G allello, whether homozygous or heterozygous, was greater in the control group (59.1%) *versus* the PCOS group (31.6%) with OR 3.1 (CI 1.002 - 9.774). We found no differences in AMH levels and the presence of AMHR polymorphisms (482A>G), and we also found no association between this polymorphism and PCOS, *p*=0.225.

**Conclusion:** We know that patients with PCOS have higher AMH levels, however we do not know exactly by what mechanisms. We found that the presence of the G allello, whether homozygous or heterozygous, was greater in the control group (59.1%) *versus* the PCOS group (31.6%), but without influencing AMH levels. This finding may suggest that genetic polymorphisms could phenotically modify ovarian function, even without altering hormonal levels.

**P-18. Deep learning for embryo assessment: A comprehensive validation study for noninvasive pregnancy probability prediction**

**Daniella Gilboa^1^, Marcos Meseguer^2^, Maya Shapiro^1^, Nicole Lustgarten^1^, Yuval Amar^1^, Yishay Tauber^1^, Akhil Garg^2^, Daniel Seidman^1^**

^1^IVF Research Department, AIVF Ltd. - Israel

^2^IVIRMA Global Research Alliance, IVIRMA Valencia - Spain

**Objective:** To discern the capacity of a deep learning pregnancy prediction model to handle variations and complexities inherent to real-world data, and establish a foundation for its prospective deployment.

**Methods:** We describe a comprehensive four-step methodology for the development and validation of the deep learning model; 1) curation of diverse datasets that are annotated appropriately and represent the intended clinical use case, 2) model training and optimization, 3) statistical analysis and performance evaluation on variable datasets not used during training, and, lastly, 4) interpretability and explainability by visualizing correlation to relevant embryo features. The train/validation dataset (n=16,935 embryos) was used for model development and optimization; the blind test dataset (n=1,708 embryos; 3 clinics) and an independently collected real-world dataset (n=7,445 embryos; 7 clinics) were used to assess performance, interpretability and explainability.

**Results:** The model was developed using time-lapse sequence data for training/validation without user input. When tested on the test set and real-world dataset, a significant difference in model scores was observed between different age categories (<35, 35-37, 38-40, 41-43, and >43 years) (p<0.01), with ascending age inversely correlated with descending AI scores (p<0.01). Likewise, scores for embryos that resulted in a clinical pregnancy, defined by the presence of a fetal heartbeat by ultrasound, (FH+) were significantly higher than for those that did not (FH-) in every age category (p<0.01). Significant positive associations were also observed between ascending scores and increasing trophectoderm quality, inner cell mass quality, and overall ASEBIR grade (p<0.001). To assess clinical utility, two score brackets were defined, labeled G4 (score ≥ 7.5; N=381 embryos) and G5 (score ≥ 8.9; N=55 embryos). Importantly, all embryos sorted into G4/G5 score brackets were annotated as top-quality (grade-A) by embryologists. The pregnancy rate for all grade-A embryos was 68.6%; pregnancy rate for all G4 embryos was 64.6%; pregnancy rate for all G5 embryos was 74.5%. Superior clinical pregnancy rates for embryos scored ≥ 8.9 when compared to embryos scored (≥ 7.5) reflects the model’s ability to discern relevant differences between top-quality morphologically similar embryos.

**Conclusion:** Deep learning for pregnancy prediction shows robust biological validity when employed on variable data, and shows superior ranking utility when applied to the subset of similar looking top-quality embryos most eligible for selection. In addition, the methodology described here sets the standard for validating the preclinical development and performance of deep learning models in the IVF clinic. This is crucial for establishing confidence and transparency before clinical adoption.

**P-19. The impact of endometriosis on embryo development and ploidy**

**Daniella Gilboa^1^, Nicole Lustgarten^1^, Maya Shapiro^1^, Yuval Amar^1^, Itamar Sayegh^1^, Yishay Tauber^1^, Daniel Seidman^1^**

*^1^IVF Research Department, AIVF Ltd. - Israel*

**Objective:** To evaluate the capability of an automated non-invasive artificial intelligence (AI)-based ploidy screening tool to provide real-time, reliable identification of euploid Day 5 embryos within patient cohorts.

**Methods:** The AI model was developed as a deep learning classifier designed for spatio-temporal sampling of the embryo time-lapse video. The AI model was trained and validated using 2,802 embryos and associated metadata. Known ploidy (1498 euploid; 1304 aneuploid) and live-birth outcomes served as ground truth labels for model development. Maternal age and embryo quality scores were incorporated into the final model’s architecture using a tabular calibrated classifier with logistic regression. Embryo quality scores were calculated using a previously developed AI model that generates a score for blastocyst-stage embryos that correlates with the likelihood of clinical pregnancy. The data were randomly partitioned into a two-part 85:15% train/validation data split. A blind test dataset (n=700 embryos; mean±SD patient age: 35.9±5.4 years) consisting of “unseen” data not used for model training and development was used to evaluate the model's predictive accuracy under various clinical scenarios.

**Results:** Embryos within the blind test dataset were retrospectively assigned an AI score ranging from 1-99, correlating with each embryo's likelihood of euploidy. Initially, all embryos were sorted into four AI score quartiles, demonstrating a significant linear relationship between increasing AI scores and ascending euploidy rates (*p*<0.001; Cochran-Armitage Test). To assess the AI model's utility in identifying euploid embryos within patient cohorts, only embryos from patients with at least one euploid and one aneuploid embryo in their cohort (n=163; 66 patients) were considered. Within this subset, euploid embryos had higher AI scores than aneuploid embryos across all patients (*p*<0.01, Mann-Whitney test), confirming robust discriminative performance. Subsequent analysis of embryos with known clinical pregnancy outcomes (n=131) showed that AI scores for embryos resulting in clinical pregnancy were statistically higher than those for non-pregnancy outcomes (*p*<0.01; Mann-Whitney test), further confirming the association between AI scores and clinical outcomes.

**Conclusion:** Using an AI-based screening tool for non-invasively assessing embryo ploidy shows robust biological validity in identifying euploid embryos within patient cohorts and demonstrates predictive capacity for triaging embryos with higher likelihood of resulting in a clinical pregnancy. The importance of such tools is underscored by the growing demand for non-invasive alternative screening methods that minimize the risks associated with conventional invasive genetic testing for aneuploidies. This approach offers a solution for patients unfit for invasive procedures or those undergoing fresh transfer, while also enhancing efficiency in IVF clinics due to its automated and real-time capabilities.

**P-20. Bariatric surgery and the impact on fertility**

**Mariana Kasuga Morya^1^, Stephanie Tasselli Alencar da Assunção^1^, Victoria Barchi Cordts^1^, Gabriel Monteiro Pinheiro^1^**

^1^Universidade Santo Amaro - São Paulo - SP - Brazil

**Objective:** The objective of the present study was to assess existing scientific evidence of the impacts in bariatric surgery on patients fertility.

**Methods:** This bibliographic review was elaborated through a search in the PUBMED database from the descriptors “Bariatric surgery”; “fertility ” and “impact”, from the last 5 years, which included clinical trial, randomized controlled trial and systematic review.

**Results:** Bariatric surgery has been shown to increase fertility rates in obese women. This is attributed to significant weight loss and the reduction of comorbidity conditions such as polycystic ovary syndrome (PCOS), which is often associated with obesity and can lead to infertility. It has been evidenced that bariatric surgery reduced the incidence of PCOS from 45% to 6.8%. A non-randomized prospective study compared reproductive function after bariatric surgery with medical therapy, showing that surgery reduced intermenstrual days and increased the number of ovulations. The results on the impact of bariatric surgery on anti-mullerian hormone (AMH) levels, a marker of ovarian reserve, are conflicting. Some studies found a significant increase in AMH levels six months after surgery, while others reported a significant decrease. Studies also presented varied results on the effect of bariatric surgery on ovarian volume, with some showing a significant decrease in volume 12 months after surgery, while others found no significant changes. Regardless of fertility markers, bariatric surgery significantly improved pregnancy rates in women with obesity and PCOS. Although there are not many studies directly evaluating the impact of bariatric surgery on reproductive outcomes after assisted reproductive therapy, there are indications that surgery may improve the effectiveness of *in vitro* fertilization and other assisted reproductive methods in women with obesity and PCOS. Overall, studies indicate that pregnancy rates may increase to 47% after bariatric surgery. A meta-analysis showed a high incidence (58%) of successful pregnancy in infertile women after surgery. This includes the reduction of risks for gestational diabetes, hypertension, and preeclampsia. After bariatric surgery, it is recommended to wait for a certain period before attempting to conceive, as this waiting period allows for weight stabilization and the resolution of any immediate post-surgical complications. It is important to emphasize that adequate supplementation and medical supervision are essential to ensure maternal and fetal health after bariatric surgery. Besides fertility and pregnancy outcomes, bariatric surgery positively impacts other aspects, such as being associated with better outcomes in reducing the risks of macrosomia and childhood obesity in children of obese mothers, an increase in the rate of normal vaginal deliveries compared to cesarean sections (73.7% vs. 26.3%), and a substantial positive impact on female fertility and sexual function.

**Conclusion:** Bariatric surgery has been shown to significantly increase fertility rates in obese women, attributed to substantial weight loss and the reduction of conditions as PCOS. It reduces the incidence of PCOS from 45% to 6.8%, decreases intermenstrual days, and increases ovulation. Conflicting results exist regarding its impact on AMH levels and ovarian volume, but overall, pregnancy rates improve significantly, with studies showing a rise to 47% and a high incidence of successful pregnancies (58%) in infertile women. Bariatric surgery also reduces risks of gestational diabetes, hypertension, and preeclampsia. A recommended waiting period post-surgery allows for weight stabilization and complication resolution. Adequate supplementation and medical supervision are crucial for maternal and fetal health, with additional benefits including reduced risks of macrosomia and childhood obesity, higher rates of normal vaginal deliveries, and improved female fertility and sexual function.

**P-21. Cryopreservation of oocytes for oncological reasons: A retrospective analysis**

**Jhuly Nunes^1^, Lucileine Keico Nishikawa^1^, Álvaro Ceschin^1^, Nathan Ceschin^1^, Onel Goitia^1^, Pamella Kreling^1^**

^1^Feliccità Instituto de Fertilidade - Curitiba - PR - Brazil

**Objective:** To discuss the cryopreservation of oocytes for oncological reasons, based on data collected over a seven-year period at the Institute.

**Methods:** Patients who cryopreserved their oocytes with the aim of preserving fertility, before starting oncological treatment, were included in this review. Data were collected during the period from 2017 to 2023. Patients were grouped according to age group into: ≤25 years (Group 1), 26-29 years (Group 2), 30-34 years (Group 3) and ≥35 years (Group 4). The number of patients treated, the number of cryopreserved oocytes, and the type of tumor were evaluated.

**Results:** During the period evaluated, 36 patients sought the institute for oocyte cryopreservation for oncological reasons, of which 6 patients aged ≤25 years, 12 patients aged 26-29 years, 11 patients aged 30 to 34 years and 7 patients with ≥35 years. In Group 1, patients cryopreserved an average of 15 oocytes, in Group 2 the average number of oocytes was 8, and in Groups 3 and 4 the average stored was 5 oocytes. The cancer with the highest incidence in patients ≤25 years old was of hematological origin (67%), while in the other groups the highest incidence was breast tumors, 42% in patients aged 26-29 years, 64% in patients aged 30-34. years and 43% in patients ≥35 years. Only 1 patient in Group 1 (17%) sought the service due to a breast tumor.

**Conclusion:** With the increase in life expectancy after oncological treatments and the satisfactory results of the vitrification technique, the search for fertility preservation prior to chemotherapy and radiotherapy becomes natural. It was observed that the greatest demand is in patients who have undergone treatment for breast tumors, followed by hematological tumors. Genital tract and bone cancers were also observed in our Institute, but at a lower incidence. In addition to preserving the patient's reproductive capacity, oocyte cryopreservation helps provide hope and emotional support, and supports patients' dreams of establishing a future family. Although successful, demand for the technique is still low, whether due to financial reasons, lack of incentive and information.

**P-22. Embryonic aneuploidy rate and the importance of internal evaluation of results**

**Lucileine Nishikawa^1^, Jhuly Nunes^1^, Ianae Ceschin^1^, Álvaro Ceschin^1^, Nathan Ceschin^1^, Onel Goitia^1^, Pamella Kreling^1^**

*^1^Feliccità Instituto de Fertilidade - Curitiba - PR - Brazil*

**Objective:** To evaluate the results of embryonic chromosomal genetic analysis from the present center (internal) compared to an analysis provider (external).

**Methods:** The study included embryos at the blastocyst stage that underwent trophectoderm biopsy to perform pre-implantation genetic testing for aneuploidy (PGT-A) from 2017 to 2023. The aneuploidy rate of the embryos was assessed according to the range maternal age. The patients were divided into 4 groups. Group 1: ≤35 years, Group 2: 36-37 years, Group 3: 38-39 years and Group 4: ≥40 years. The internal rates were compared with the genetics laboratory groups, as follows: Group 1: ≤35 years, Group 2: 35-37 years, Group 3: 38-40 years, Group 4: 41-42 years, and Group 5: >42 years. The data was compared with an (external) analysis provider that has approximately 60,000 embryo biopsy samples in its database.

**Results:** 171 embryos from 82 patients were evaluated, biopsied and genetically analyzed. In Group 1, 28 embryos were analyzed, with an aneuploidy rate of 22%. In Group 2, 24 embryos were evaluated with an aneuploidy rate of 42%. In Group 3, of the 54 embryos, 49% of them were aneuploid. In Group 4, with 65 embryos, and a 77% aneuploidy rate. Compared to analysis provider (external) aneuploidy rates based on each group were: Group 1: 51.8%, Group 2: 54.4%, Group 3: 67.9%, Group 4: 77, 9% and Group 5: 79.8%.

**Conclusion:** Based on the data collected, it was observed that there are differences in aneuploidy rates in relation to maternal age at the center and the respective external provider. The study emphasizes the importance of each center validating its aneuploidy rates according to maternal age, since there are heterogeneous population variables that can influence the analysis, a fact that directly impacts reproductive genetic counseling. This work highlights the importance of validating the aneuploidy rate in each center, in order to offer greater accuracy for personalizing *in vitro* fertilization treatment.

**P-23. Influence of obesity on laboratory rates in RHA treatment**

**Jhuly Nunes^1^, Lucileine Nishikawa^1^, Álvaro Ceschin^1^, Nathan Ceschin^1^, Onel Goitia^1^, Pamella Kreling^1^**

^1^Feliccità Instituto de Fertilidade - Curitiba - PR - Brazil

**Objective:** To compare laboratory results of oocytes from patients with normal body mass index (BMI), versus oocytes from patients with BMI of different degrees of overweight.

**Methods:** This retrospective study was based on cycles carried out between 2017 and 2023. Only patients with fresh cycles and Blastocyst stage transfer were included in the study. For analysis, the following data were used: number of oocytes aspirated; numbers of mature oocytes; fertilization rate; cleavage rate; blastocyst development rate and clinical pregnancy rate. Patients were divided according to BMI (kg/m2) into the following categories: Group 1 - normal weight with BMI of 18.5 - 24.99; Group 2 - overweight with BMI of 25 - 29.99; Group 3 - grade I obesity with a BMI of 30 - 34.99 and Group 4 - grade II obesity with a BMI of 35 - 39.99.

**Results:** In total, 595 patients were evaluated, 356 from Group 1, 141 from Group 2, 53 from Group 3 and 15 patients from Group 4. The average age of patients in the groups was similar, 35.88; 35.35; 35.62 and 36.13, respectively. The maturation rate of aspirated oocytes and the fertilization rate were, respectively: 74.85% and 82.78% (Group 1); 70.27% and 81.70% (Group 2); 75.97% and 80.81% (Group 3) and 78.67% and 76.27% (Group 4). The rate of cleavage and development into Blastocyst were, respectively: Group 1 - 96.08% and 53.19%; Group 2 - 98.33% and 54.05%; Group 3 - 98.33% and 59.04%; Group 4 - 100% and 68.18%. The clinical pregnancy rate in Group 1 was 47.61%, Group 2 56.25%, in Group 3 62.5%. There was no embryo transfer in Group 4.

**Conclusion:** Based on this study, it was observed that laboratory results were similar between the groups, demonstrating that obesity did not significantly affect laboratory rates. Group 4 presented a lower fertilization rate when compared to the other groups, on the other hand it demonstrated a more satisfactory Blastocyst development. Therefore, more studies are needed to highlight the influence of female obesity on the results of RHA treatments.

**P-24. Revisiting neonatal uterine bleeding: unraveling the mystery behind reddish spots on diapers**

**Maíra Casalechi^1^, Marco Reschini^1^, Giorgia Di Stefano^1^, Giorgia Carullo^1^, Irene Mondini^1^, Davide Marinello^1^, Bianca Magni^1^, Claudia Ferraro^1^, Giada Polenghi^1^, Paola Viganò^1^**

^1^Fondazione IRCCS Ca' Granda Ospedale Maggiore Policlinico Milano - Italy

**Objective:** Neonatal uterine bleeding is a menstrual-like bleeding that occurs during the first days of life in female newborns. Although epidemiological studies have reported an estimated incidence of 3-5%, this evidence is outdated. Moreover, urate crystals are commonly present in urine of newborn babies and can produce a red discoloration of the diaper, which can be erroneously perceived as blood. The study aimed to further investigate genital crises and the potential confounding factor due to urate crystals.

**Methods:** This prospective cross-sectional study involved 628 women carrying a singleton fetus before delivery (520 women carrying a female and 108 a male fetus). Upon recruitment, participants were asked to complete a questionnaire regarding general clinical characteristics and personal habits. Data on the mode of delivery, breastfeeding, and neonatal health were extracted from patients’ charts. Moreover, a follow-up phone call was conducted two weeks after delivery to investigate whether blood spots had been noticed on the baby’s diaper and their duration. Finally, patients were asked to preserve the diaper on which they observed reddish spots, and a specific commercial kit (Seratec® - Germany) was used to detect the presence of menstrual blood and/or peripheral blood.

**Results:** Reddish spots, perceived by mothers as blood, were documented in 118 female babies, corresponding to 23% (95% CI: 19-27%), and in 22 males, corresponding to 20% (95% CI: 14-29%). There was no significant difference observed in the frequency between newborns of the two sexes (p=0.70). The median [Interquartile range] time since delivery in girls and boys was 2 [1-3] and 1 [0-1] day, respectively (*p*=0.001), while the duration of the episode was 2 [1-4] and 2 [1-2] days, respectively (*p*=0.33). Furthermore, the blood detection kit was used on the diapers of 46 infants showing reddish stains (21 male newborns and 25 females). The kit detected the presence of blood in 7 (33%) diapers of male newborns and 8 (32%) of female newborns (*p*=1.00), with only one case showing a faint presence of menstrual blood.

**Conclusion:** The comparable incidence of reported reddish spots in both females and males suggests that neonatal uterine bleeding can frequently be mistaken for the presence of urate crystals. Furthermore, urate crystals could cause microlesions in the urinary tract of newborns, resulting in the presence of red blood cells in urine, as shown by positive test results even in male newborns. Despite this, there seems to be a difference in the timing of onset between males and females, possibly due to an early appearance of urate crystals compared to neonatal uterine bleeding. This way, the mere observation of reddish spots on a baby's diaper may not be adequate to distinguish neonatal uterine bleeding, even because the presence of urate crystals could lead to the presence of red blood cells. Therefore, despite some authors speculate on the potential role of neonatal uterine bleeding in the pathogenesis of endometriosis, considering the potential for overestimation of the phenomenon, further studies are necessary to establish an effective correlation between the two events.

**FUNDING:** Italian Ministry of Health - Ricerca Finalizzata

**P-25. The Kallikrein activities in normoviscous and hyperviscous seminal plasma and its association to infertility**

**Maria Eduarda Leite Simões^1^, Amanda Wink Santos^1^, Charles Teilor Rodrigues^2^, Luciane Cabreira Baptista^2^, Ilma Simoni Brum^3^, Paula Barros Terraciano^1^, Edison Capp^4^, Markus Berger Oliveira^1^**

^1^Grupo de Reprodução e Farmacologia Celular (REPROFARM), Centro de Pesquisa Experimental - Hospital de Clínicas de Porto Alegre - Porto Alegre - RS - Brazil

^2^Unidade de Reprodução Humana, Hospital Femina - Grupo Hospitalar Conceição - Porto Alegre - RS - Brazil

^3^Instituto de Ciências Básicas da Saúde, Departamento de Fisiologia - Universidade Federal do Rio Grande do Sul - Porto Alegre - RS - Brazil

^4^Programa de Pós-Graduação em Ciências da Saúde - Ginecologia e Obstetrícia, Faculdade de Medicina - Universidade Federal do Rio Grande do Sul - Porto Alegre - RS - Brazil

**Objective:** Hyperviscous semen is a physical condition where seminal plasma strongly adheres to itself after coagulum degradation. It occurs in 11%-32% of ejaculates, but its etiology and impact on male fertility are not well understood. This study aims to evaluate the mucoprotein content and measure total protease and kallikrein activities in the seminal plasma of normoviscous and hyperviscous samples from fertile and infertile patients.

**Methods:** Patients were recruited and divided into four groups: i. Fertile normoviscous (n=11); ii. Fertile hyperviscous (n=6); iii. Infertile normoviscous (n=7); iv. Infertile hyperviscous (n=12). Clinical data, mucoprotein content, and protease activities in seminal plasma were analyzed.

**Results:** We found an association between hyperviscosity and seminal characteristics of infertility, including reduced volume, sperm concentration, motility, and vitality. Additionally, hyperviscous semen showed a significant increase in neutrophil counts (0.06±0.01x106/mL in fertile normoviscous vs. 0.32±0.052x106/mL in infertile hyperviscous). The mucoprotein content was reduced by 20% in hyperviscous and infertile samples, indicating a deficiency in seminal plasma components. These results suggest that hyperviscous semen is of lower quality compared to normoviscous semen. Caseinolytic analyses revealed increased activity in fertile hyperviscous semen (1.67 times higher). Similarly, gelatinolytic analyses showed increased metalloprotease activity in both hyperviscous and infertile groups. Specific analysis of kallikreins revealed a 60% increase in tissue kallikrein activity in hyperviscous semen, and a 32% increase in plasmatic kallikrein activity in both hyperviscous and infertile samples. The increased protease activity suggests that hyperviscosity is not due to a lack of seminal coagulum liquefaction.

**Conclusion:** Given that increased proteolytic enzyme activity is associated with inflammatory processes and that these proteases are secreted from the prostate, we suggest that an inflammatory process in the prostate gland is occurring in patients with hyperviscous semen.

**P-26. Incidence of endometritis diagnosed by hysteroscopy prior to elective transfer of a single euploid frozen embryo (e-SET)**

**Victoria Barchi Cordts^1^, Beatriz Gordilho Bacos^1^, Emerson Barchi Cordts^2^, Caroline Aragão Carvalho^2^, Gabriel Monteiro Pinheiro^1^**

^1^Universidade Santo Amaro - São Paulo - SP - Brazil

^2^Ideia Fértil - São Paulo - SP - Brazil

**Objective:** This study’s objective is to evaluate the number of women diagnosed with endometritis via hysteroscopy before undergoing an elective transfer of a single euploid frozen embryo (e-SET)

**Methods:** This is a cross-sectional study that analyzes the medical records of patients treated at a private clinic who underwent hysteroscopy prior to the elective transfer of a single euploid frozen embryo (e-SET) between January 2022 and May 2024.

**Results:** The chronic endometritis (CE) has been identified as a significant cause of infertility in women, also contributing to decrease the taxes of success in *in vitro* fertilization (IVF) cycles. This disease is often associated with several bacterias contributing to persistent inflammation of the endometrium. Among the main bacteria detected are Streptococcus spp., Staphylococcus spp., Escherichia coli (E. coli), Enterococcus faecalis, Mycoplasma spp. (including Mycoplasma hominis), Ureaplasma spp., and Chlamydia trachomatis. The presence of these bacteria can significantly alter the microenvironment, affecting endometrial receptivity and embryo implantation, which can lead to repeated failures in IVF and recurrent miscarriages. This highlights the importance of the early and precise detecting and appropriately treating endometrial infections in women with unexplained infertility and implantation failures, that is why hysteroscopy has a crucial role in the evaluation and management of women in treatment for infertility, contributing to more effective therapeutic strategies because targeted antibiotic treatment against these bacteria can significantly improve reproductive outcomes, reinforcing the need for a comprehensive diagnostic approach that includes testing for these common pathogens. In addition to hysteroscopy and tests for the pathogens mentioned above, immunohistochemical examination for CD56 and CD138 can also be performed, which plays a crucial role in the diagnosis and management of infertility, especially in cases of CE and repeated implantation failures. CD56 is a marker of uterine natural killer cells (NK), which play a vital role in modulating the endometrial immune response and preparing the uterus for embryo implantation. Studies show that altered expression of NK cells (CD56+) is associated with unexplained infertility and recurrent IVF failures. On the other hand, CD138 is a marker of plasma cells used to identify the presence of chronic inflammation in the endometrium. The presence of CD138+ plasma cells is a reliable indicator of CE. Its detection through immunohistochemistry improves the accuracy of CE diagnosis, allowing for more targeted antibiotic treatment and consequently increasing reproductive success rates. However, it is still not a widely used test, as only 21% of the sample underwent it, and among these women, all tested positive only for CD56, so none presented CD138. This study analyzed 33 patients without any signs and symptoms of endometritis, but had difficulties in reproductive success therefore being indicated for the investigative hysteroscopy. Among these 33 patients, 16 (48.5%) were diagnosed with endometritis. Inside this group of 16 after the treatment with azithromycin and metronidazole, 10 (62.5%) managed to get pregnant.

**Conclusion:** In summary, we can conclude that among all the women who underwent hysteroscopy due to infertility in the past two years, almost half had endometritis, a condition that hinders natural pregnancy, as evidenced by the successful pregnancies after treatment in the majority of patients in our sample. This demonstrates that endometritis is a significant condition in human reproduction that should be investigated before undergoing reproductive treatment, as there is a high chance of it being the cause of infertility.

**P-27. At-home Insemination and Motherhood: Mental Health of lesbian women trying to get pregnant**

**Bruna Mendes Roza Rodrigues^1^, Ana Cristina Barros da Cunha^1^**

^1^Universidade Federal do Rio de Janeiro - Rio de Janeiro - RJ - Brazil

**Objective:** Home Insemination (HI) is an unconventional and low-cost human reproduction method, which carried out outside specialized medical clinics, and that has been used by lesbian women to become mothers. Considering the psychosocial vulnerability of the LGBTQIA+ population, as well as the biological and psychological risks involved in using an unconventional method of conception, the objective of this study was to investigate the mental health of lesbian women who used HI to become pregnant.

**Methods:** This is a quantitative study with 134 Brazilian women over 18 years old, self-declared lesbians and using HI, who were recruited from Facebook groups. They all responded to a Google form with the following instruments: 1) Brazilian version of the Depression, Anxiety and Stress Scale - DASS-21, to investigate mental health indicators, such as symptoms of depression, anxiety and stress; and 2) General data questionnaire, to identify the sociodemographic profile of the participants. The occurrence of mental health indicators (anxiety, stress and depression) was analyzed by calculating means, standard deviations and frequencies of stress, anxiety and depression scores obtained by DASS-21.

**Results:** The average age of women was 28 years old and 81.1% of them did not have children. Furthermore, 56% had higher education and 54.5% was employed with 1 to 3 minimum wages. A higher mean stress score (SD=11.37) was observed, in addition to a higher occurrence of stress levels (15.57%), followed by anxiety (7.55%) and depression (7.12%). Although it has become an important method of conception for this public, because it is accessible, financially viable and promote the couple autonomy to get pregnant, HI is surrounded by social stigmas, biological risks and bioethical issues, which makes women who use it more psychically vulnerable.

**Conclusion:** Our findings suggest that these women may be more susceptible to stress, which can result in even more serious psychological distress, such as anxiety and depression. The importance of training health professionals, including doctors, to provide comprehensive care to this population is highlighted. They can offer support and multidisciplinary care to these women, attended in health services regardless of the method of conception.

**P-28. The use of embryo biopsy as a tool for screening genetic diseases: A systematic review**

**Larissa Alves Honorato Ferreira^1^, Alicia de Oliveira Fernandes Felipe^1^, Ana Carolina Revoredo Galvão^1^, Ana Paula de Oliveira Dantas^1^, Giovana Azevedo Braga^1^, Giovanna Karen de Souza Rodrigues^1^, Hailton Pereira de Melo Júnior^1^, Heloisa Campos de Amorim^1^, Michelle Moreira de Lima^1^, Melissa Tomé Varela^1^, Raissa dos Santos Belarmino^1^, Ricardo Gabriel de Lima Bisneto^1^, Vitória Maria de Souza^1^, Julliane Tamara Araújo de Melo Campos^1^**

^1^Liga Acadêmica de Fertilidade e Reprodução Assistida (LAFRA) - Universidade Federal do Rio Grande do Norte (UFRN) - Natal - RN - Brazil

**Objective:** This review aims to understand the importance and challenges of embryo biopsy as a support for managing genetic tests and diagnosing diseases, syndromes, or specific disorders.

**Methods:** For this review, we searched the keywords “embryo biopsy” and “genetic” in PubMed and the Web of Science database. We considered publications during the period between 2019 and 2024. We found 93 and 104 articles in PubMed and the Web of Science database, respectively. After excluding duplicated articles, 117 publications were found. After, we excluded articles that were not available in English or Spanish; articles focused on the history of preimplantation genetic testing (PGT); articles related to artificial intelligence; research carried out with animals; articles that are not free; articles that do not relate to embryo biopsy to genetics; articles related to ethical and religious issues; and articles that did not use embryo biopsy. Based on these criteria, 61 articles were included.

**Results:** Embryo biopsy for PGT is crucial for couples at risk of transmitting severe genetic diseases to their offspring, such as Turner syndrome, cystic fibrosis, Huntington's disease, or congenital lipodystrophies. PGT also helps to identify gender-related disorders and structural and numerical chromosomal changes. This technique allows the selection of suitable embryos, increasing the chances of a healthy pregnancy and reducing the risk of miscarriage, especially in older women, who present a higher frequency of abnormal chromosomes and mosaicism and lower embryo quality. However, this practice can present potential risks. Limited evidence suggests some caution, as PGT is associated with risks of hypertensive disorders and preterm births, depending on the biopsy stages and the type of embryo transfer. Further, factors such as embryonic mosaicism, where cells from the same embryo have different genetic compositions, can affect the accuracy of these tests, especially preimplantation genetic testing for aneuploidy (PGT-A), leading to clinical outcomes full of uncertainties. Regarding stages, day-5 embryo biopsy (blastocyst) for PGT is more accurate and can help to reduce inconclusive results, as well as improve survival and pregnancy outcomes post-transfer compared to day-3 biopsy (cleavage stage). Finally, blastocoel fluid analysis and non-invasive PGT (niPGT) techniques emerge as promising solutions to avoid the risks of traditional biopsy, and they can provide additional information on the embryo's potential.

**Conclusion:** Embryo biopsy is essential to PGT, helping screen hereditary diseases and possible disorders newborns can develop during their lifespan. However, as with every tool, there are advantages and disadvantages. For embryo biopsy, as the embryo is very fragile, collecting the sample may result in other problems beyond the genetic diseases that it aims to identify, such as hypertensive disorders and preterm birth. Therefore, embryo biopsy is a subject that requires more research concerning how to increase its accuracy and minimize its flaws. In conclusion, embryo biopsy is a promising tool for screening genetic diseases. Here, we reviewed its importance, highlighting points that need improvement and seeking better patient outcomes.

**P-29. Relationship between hypothyroidism and female infertility: Integrative review**

**Letícia Aimí Moraes^1^, Lenara Carvalho^1^, Natalia Fabricio^1^, Brenda Santos Oliveira^1^, Ana Livia de Oliveira Moreno^1^**

^1^Universidade de Marília - Marília - SP - Brazil

**Objective:** To discuss the relationship between hypothyroidism and female infertility.

**Methods:** Integrative review carried out between May and June 2024 using the PICO strategy: Population (P), Women with hypothyroidism; Intervention (I), Diagnosis and monitoring of hypothyroidism; Comparison (C), Women without hypothyroidism or with well-controlled hypothyroidism; and Outcome (O), Prevalence of infertility and therapeutic results. The central question was: "What is the relationship between hypothyroidism and female infertility?". PubMed, Latin American and Caribbean Health Sciences Literature (LILACS), and Scientific Electronic Library Online (SCIELO) databases were consulted using descriptors (MeSH): "Association", "Hypothyroidism", "Female infertility" and their respective Portuguese terms combined with the Boolean operator AND. Prevalence studies, clinical trials, cross-sectional studies, and prospective studies in Portuguese or English from 2010 to 2024 were included. Duplicate articles, reviews, and studies that did not meet the objective were excluded. Two independent reviewers selected the data and resolved disagreements through discussion or consultation with a third reviewer.

**Results:** Eight studies were selected. The investigation revealed that the majority of women studied had secondary infertility, often related to subclinical hypothyroidism. The mean age of participants was 31 years, with 80% experiencing primary infertility and 20% secondary infertility, including cases of recurrent miscarriages and term pregnancies. The average duration of the problem was 4.5 years, and approximately half of the women experienced a period of infertility between two and five years, with a prevalence of overweight. Nearly half of the women with infertility had a thyroid-stimulating hormone(TSH) value above 2.5 µIU/mL. Women with primary infertility showed higher average thyroid-stimulating hormone levels, with significantly lower levels of serum triiodothyronine. After one year of treatment with thyroid hormone in 59 women, 25.42% achieved pregnancy, with a statistically significant difference. Conversely, another study associated women with TSH ≥2.5mIU/L or thyroid autoimmunity with no increased risk of miscarriage, reduced live birth rate, or decreased fecundability compared to those with TSH <2.5mIU/L, after adjusting for age and body mass index.

**Conclusion:** Early diagnosis of thyroid disorders in women results in financial savings and improvements in subsequent pregnancy rates. Treatment was correlated with an increase in the proportion of successfully completed subsequent pregnancies.

**References**

Acosta-Chacaltana et al. Rev Soc Peru Med Intern. 2011; 24: 113-115.

Bartáková *et al*. BMC Pregnancy Childbirth. 2013;13:217.

Cai *et al*. Reprod Biol Endocrinol. 2017;15:39.

Del Valle. Rev Med Tucumán 2010; 15: 15-21.

KIM *et al*. Fertil Steril. 2011;95(5):1650-4.

Lugo Montoya *et al*. Horiz Sanitário. 2019;18:319-324.

Plowden *et al*. Endocrinol Metab. 2016;101:2358-65.

Valle-Pimienta *et al*. Arch Méd Camaguey 2020; 24: e7362

**P-30. Ovariectomy-Induced menopausal hypertension elevates Aortic Chymase activity and vascular wall thickness in experimental study**

**Amanda Wink Santos^1^, Cristiana Palma Kuhl^1^, Maria Eduarda Leite Simões^1^, Eduardo Pandolfi Passos^1^, Paula Barros Terraciano^1^, Markus Berger Oliveira^1^**

^1^Grupo de Reprodução e Farmacologia Celular (REPROFARM), Grupo de Pesquisa Experimental - Hospital de Clínicas de Porto Alegre - Porto Alegre - RS - Brazil

**Objective:** Vascular remodeling is a complex process that contributes to cardiovascular diseases, particularly in postmenopausal women. This study investigates the molecular mechanisms underlying vascular remodeling following ovariectomy in both normotensive and spontaneously hypertensive rats.

**Methods:** Female rats of the Wistar Kyoto (WKY) strain and the Spontaneously Hypertensive Rat (SHR) strain were subjected to either a sham procedure (SHAM) or a surgical ovariectomy (OVX) and monitored for 150 days while receiving a specific soy-free diet. This procedure resulted in four experimental groups (n = 10/group): i. WKY-SHAM, ii. WKY-OVX, iii. SHR-SHAM, and iv. SHR-OVX. During the experimental period, cardiovascular parameters were obtained, and at the end, blood, aorta, and uterus were collected for analysis.

**Results:** Ovariectomy induced significant body mass increases and uterine atrophy, confirming estrogen reduction. Additionally, it elevated systolic blood pressure and exacerbated hypertension in SHR rats. Blood pressure changes correlated directly with uterine atrophy, indicating a link between estrogen reduction and blood pressure regulation. Histological analysis of the abdominal aorta revealed increased thickness in major arterial layers, particularly the medial layer (tunica media). Quantification of layer areas showed significant increases in the adventitial and medial layers without changes in the vessel lumen. Tunica media hypertrophy was associated with reduced elastic fiber density. Proteolytic enzyme activities in aorta and peritoneal cells, demonstrating increased caseinolytic, elastolytic, chymase, tryptase, and elastase activities post-ovariectomy. This pattern was consistent regardless of baseline hypertension. Chymase expression in the aorta also increased, along with the expression of growth factors like VEGF, EGF, and TGF-β. *In vitro* experiments with purified chymase showed its ability to increase vascular smooth muscle cell (VSMC) viability and stimulate VSMC migration in a dose-dependent manner.

**Conclusion:** Overall, these findings suggest that ovariectomy-induced estrogen reduction leads to vascular remodeling characterized by arterial wall thickening, decreased elastic fiber density, altered proteolytic enzyme activity, and upregulation of growth factors. Chymase, in particular, appears to play a crucial role in VSMC proliferation and migration, contributing to the vascular changes observed in this model.

**P-31. Results of *in vitro* fertilization treatments analyzing different parameters and seminal processes**

**Kazue Harada Ribeiro^1^, Mila Harada Ribeiro Cerqueira^1^, Gustavo Cerqueira Silva^1^, Roberta Marcolino Tesch^1^, Vanessa Nayane Perez^1^**

^1^CLINIFERT - Florianópolis - SC - Brazil

**Objective:** To evaluate fertilization rates, embryonic development, pregnancy rate and euploidy rate according to different seminal parameters in couples undergoing *in vitro* fertilization (IVF) treatments.

**Methods:** Retrospective cohort study including 710 intracytoplasmic sperm injection (ICSI) cycles during the period from January 2022 to December 2023. The samples included in the study were ejaculated semen whose initial sample concentration was greater than 1 million motile sperm per milliliter (mL). The seminal parameters used in the analysis of results and rates were those pre-capacitation, and therefore we divided the groups analyzed according to the indication of seminal processing for treatment - resulting in three groups: sperm wash for samples with total concentration up to 5 million/ mL (GROUP 1 - 248 cycles); microfluidics - zymot^®^ for samples showing teratozoospermia, high sperm DNA fragmentation and patients with diminished ovarian reserve (GROUP 2 - 93 cycles); swim-up for seminal samples with little or no change (GROUP 3 - 369 cycles). For data analysis, the Kruskal-Wallis statistical test was used and a value of *p*<0.05 was considered significant, and to investigate the difference between groups, the Dunn's podhoc test was used and a value of *p*<0.5 was considered significant.

**Results:** The parameters showed significant differences between the groups. The results suggest that samples indicated for swim-up and zymot^®^ had higher sperm concentration and motility compared to those indicated for sperm wash (*p*<0.01). There was a predominance of samples with indication parameters for sperm wash (69.4%), followed by zymot^®^ (54.8%) and swim-up (20.60%) (*p*<0.01), although more Half of the cases that used zymot^®^ also presented at least one altered seminal parameter. There were no differences in the rate of fertilization and cleavage between the groups, but rather in the formation of blastocysts between GROUP 2 *vs*. GROUP 1 and between GROUP 3 *vs* GROUP 1 (*p*<0.01). There was no significant difference between GROUP 2 *vs*. GROUP 3 (*p*>0.99), indicating a possible impact of the techniques from the third day of embryonic development, with zymot^®^ and swim up being superior and similar to each other. No significant differences were observed in the rate of positive serum β-hCG levels and formation of euploid embryos between the groups (*p*=0.058 and *p*=0.23, respectively).

**Conclusion:** Based on the results of the study, it is possible to conclude that sperm training techniques for ICSI obtained favorable results in different proportions. Although the results of GROUP 2 and GROUP 3 did not show any statistical difference between them, both were superior to the results of GROUP 1 in relation to the formation of blastocysts - an important difference in the success of *in vitro* fertilization treatment. Therefore, it is worth noting that microfluidics can be a good tool in cases with male factor infertility, since its results were similar to those with mild or absent male factor. However, we emphasize that the analysis was carried out on a heterogeneous population sample and the results cannot be applied to the general population. It is also important to highlight that the choice of sperm selection technique must be individualized, taking into account the specific characteristics of the couple, to obtain better results.

**P-32. The Use of Encapsulated Chitosan for Dose Reduction of GnRH and Optimization of Results in Assisted Reproductive Techniques**

**Qiuxin Lin Carretoni^1^, Amanda Cerquearo Rodrigues Santos^2^**

^1^Universidade do Vale do Paraíba - São José dos Campos - SP - Brazil

^2^Reproferty - São José dos Campos - SP - Brazil

**Objective:** To evaluate the efficacy of chitosan nanoparticles (CN) as intermediaries for the release of gonadotropin-releasing hormone (GnRH) in assisted reproductive techniques.

**Methods:** To conduct this literature review, searches for scientific articles were carried out in the PubMed, SciELO, Scopus, and Google Scholar databases, using the descriptors "chitosan nanoparticles" and "GnRH," from 2011 to 2024, written in English. The inclusion criterion was experimental studies that specifically addressed the use of CN for GnRH application during the oocyte maturation phase in assisted reproductive treatment. Articles that did not meet the established criteria and study objectives were excluded.

**Results:** From the initial search, 39 articles were identified, but only 14 met the established inclusion criteria. Notably, all studies addressed animal assisted reproduction and were organized into tables for easier understanding and analysis. Upon reviewing the pre-selected articles, it was found that in 10 out of 14 studies, the use of CNs was effective in improving reproductive outcomes. This is attributed to the controlled release properties of the encapsulated hormone, indicating a significant advancement in assisted reproduction techniques. According to Rather *et al*. (2013), CNs increased the fertilization rate by 13% compared to pure GnRH. Kookaram *et al*. (2021) observed that CNs accelerated ovarian development, reducing the time to maturity by 20 to 30 days, compared to 30 to 40 days in the control groups, representing an improvement of 28.6% to 50%. Gallab *et al*. (2022) noted that the modified protocol with CNs resulted in an 80% higher estrus induction rate and increased conception rate to 75%, compared to 40% with the standard protocol. Amin & Said (2021), as well as Hashem *et al*. (2023), reported a conception rate improvement of about 30% in the group treated with CNs. Additionally, six articles highlighted that the use of encapsulated chitosan hormones resulted in a reduction in the required hormone dose without affecting fertility. These articles reported a total dose reduction of 25% to 75% of GnRH, with approximately 50% of these results being statistically significant, achieving a *p*-value < 0.05.

**Conclusion:** Based on the analyzed studies, CNs containing GnRH have proven to be a promising technique for enhancing fertilization rates and other aspects of animal assisted reproduction, offering improvements in estrus induction, conception rate, and oocyte maturation. To solidify the applicability of CNs, additional studies on their long-term stability, safety, and efficacy under various clinical conditions are necessary. These results have the potential to shape future research perspectives in human assisted reproduction, highlighting the need for dedicated studies to ensure the safety and efficacy in transitioning these technologies to human applications.

**P-33. Oncofertility: what is the quality of the information provided by Dr Google in Brazil?**

**Laudislena Colodetti^1^, Bruna Oliveira Martins^1^, Marcos Sampaio^2^, Marisa Mendonça Carneiro^3^, Marcia Mendonça Carneiro^2^**

^1^ART BH Medicina Reprodutiva - Belo Horizonte - MG - Brazil

^2^Origen Centro de Medicina Reprodutiva - Belo Horizonte - MG - Brazil

^3^Faculdade de Letras da UFMG - Belo Horizonte - MG - Brazil

**Objective:** To investigate oncofertility care policies, guidelines, recommendations and services as well as the availability of oncofertility care in Brazil through electronic search of public available data.

**Methods:** A search was performed on Google in January 2024 using the terms: “oncofertility", "cancer and pregnancy", “breast cancer and pregnancy”, "fertility preservation” and "fertility preservation and cancer”. Pages containing information related to oncofertility targeted to the public audience were eligible. As individuals rarely examine more than the first three pages of a Google search, information obtained from the first 3 pages in Google search were evaluated. Information available in the selected webpages was evaluated using the DISCERN tool to check the quality and reliability of posts. The DISCERN instrument (http://www.discern.org.uk/discern_instrument.php) comprises 16 questions divided into 3 sections: reliability (1-8); quality of information on treatment choices (9-15) and overall rating (16). Each question was scored from 1 to 5 (1 = no agreement; 5 = highest agreement). For this study questions 1 to 8 were used: (1) Are the aims clear?;(2) Does it achieve its aims?; (3) Is it relevant?; (4) Is it clear what sources of information were used to compile the publication (other than the author or producer)?; (5) Is it clear when the information used or reported in the publication was produced?; (6) Is it balanced and unbiased?; (7) Does it provide details of additional sources of support and information?; (8) Does it refer to areas of uncertainty? Eventual divergences were settled among authors. For each question, the mean score among three authors was calculated. No ethical approval was necessary as only publicly available online information was used (Ministério da Saúde - Resolução nº 510 de 07 de abril de 2016 do Conselho de Ética em Pesquisa -COEP). All data presented here were publicly available and did not involve any patient nor protected information.

**Results:** Our search retrieved 20 webpages, of which 10 authored by health professionals including hospitals, clinics and doctors; four magazines and newspapers webpages; two academic journals and 4 blogs and the other types of webpages. They were evaluated according to DISCERN questions 1 to 8. :

(1) Are the aims clear? 4.5

(2) Does it achieve its aims? 4.5

(3) Is it relevant? 4.6

(4) Is it clear what sources of information were used to compile the publication (other than the author or producer)? 2.5

(5) Is it clear when the information used or reported in the publication was produced? 3.1

(6) Is it balanced and unbiased? 3.7

(7) Does it provide details of additional sources of support and information? 2.1

(8) Does it refer to areas of uncertainty? 2.6

The average score was 3.49.

**Conclusion:** Information on oncofertility provided by Google is moderately reliable according to the DISCERN instrument. Although the available information is relevant and the aims are clearly presented, it fails in terms of providing the additional sources of support and does not refer to areas of uncertainty. The results shown here may reflect the low usage in fertility preservation for cancer in Brazil. Google is the most popular engine used to obtain medical information by the lay population but unfortunately the quality of such information is heterogeneous and rarely evaluated. The development of online patient education resources on oncofertility and fertility preservation may be important tools to educate people and improve oncologic and fertility care. Further studies should concentrate on improving the quality and reliability of oncofertility related information.

**P-34. Vaginal laceration requiring suture after ultrasound-guided transvaginal oocyte retrieval for social fertility preservation - Case report**

**Bruno Ramalho Carvalho^1^, Stella Vieira Santos^2^, Victoria Gontijo Duarte^2^, Estella Thaisa Sontag Reis^3^, Maria Eduarda Bonavides Amaral^4^, Gabriela Queiroz Campelo^3^**

^1^Bruno Ramalho Reprodução Humana - Brasília - DF - Brazil

^2^Hospital Sírio-Libanês Brasília - Brasília - DF - Brazil

^3^Centro Universitário de Brasília - CEUB - Brasília - DF - Brazil)

^4^Genesis Centro de Assistência em Reprodução Humana - Brasília - DF - Brazil

**Objective:** To present a case of vaginal laceration requiring suture after ultrasound-guided transvaginal oocyte retrieval for social fertility preservation.

**Methods:** Case report; a 35-year-old overweight woman, body mass index 29.04 kg/m^2^, diagnosed with polycystic ovaries syndrome attempting egg freezing for social fertility preservation. No alterations were identified in pretreatment blood tests and the basal sonographic exam revealed high ovarian reserve, counting more than 80 antral follicles. Considering comfort and low costs, a patient-friendly progestin-primed ovarian stimulation protocol was proposed, initiating follitropin delta 13 mcg/day after confirmation of the absence of a dominant follicle, followed by desogestrel, 75 mcg/day, from stimulation day 5 onwards, for pituitary suppression. Triptorelin acetate 0.2 mg was used for trigger in the twelfth day of stimulation, when 33 follicles reached a mean diameter of at least 16 mm. Oocyte pickup was proceeded 36 hours after trigger.

**Results:** A total of 41 oocytes were yielded, resulting in the vitrification of 36 metaphase II oocytes, 12 of which were altruistically donated. During the follicular aspiration, intense abdominal breathing movements were identified, leading to difficulties to assess the ovaries, due to their large-amplitude movements. After the procedure, significant vaginal bleeding was promptly identified. Proper vaginal speculum examination revealed a 5 cm vaginal laceration from the middle third of the vagina to the right fornix, restricted to mucosa, and presenting active bleeding. The laceration was attributed to the pressure of the transvaginal probe over the site of repeated retrieval needle perforations. Continuous blood oozing was difficult to control using compression dressings, so the patient was transported to a hospital set. The vaginal laceration was closed with 2-0 polyglactin continuous suture. The woman persisted hemodynamically stable from the moment of bleeding identification, as though as throughout the surgical procedure, being discharged after a 4-hour in-hospital observation period.

**Conclusion:** Vaginal laceration requiring suture may be a rare complication of ultrasound-guided transvaginal follicle aspiration aiming egg retrieval, mainly in difficult procedure that need high pressure from the probe over fornixes. Physicians must pay attention to bleeding pattern immediately after the procedure, aiming to promptly identify the complication and offer adequate approach.

**P-35. Influence of endometrial thickness on artificial transfer cycles of cryopreserved embryos on pregnancy rates**

**Barbara B Schroff^1^, Alexandre V. Moraes^1^, Lorena Cristina Santos^2^, Mario Silva Approbato^2^, Gustavo C Borges^3^, Amanda A Araujo^3^, Jane P Rocha^3^, Eduardo E Castro^3^**

^1^Universidade Federal de Goias - Goiânia - GO - Brazil

^2^Hospital das Clínicas de Goiás - Goiânia - GO - Brazil

^3^Clínica Humana Medicina Reprodutiva - Goiânia - GO - Brazil

**Objective:** The objective of this study was investigating the relationship between endometrial thickness (ET) on the transfer day and pregnancy rates.

**Methods:** This is a retrospective study, using medical records of patients who underwent a frozen-thawed embryo transfer (FET) cycle from January 2021 to December 2023. A total of 404 cryopreserved embryo transfers cycles were performed by the advisor in this period. One cycle was canceled due to the lack of embryos after thawing and fourteen were excluded because the transfer was not carried out at the blastocyst stage. Three hundred eighty-nine (389) embryo transfers cycles were included in accordance with the inclusion criteria. All patients were seen by the same physician, received the same endometrial preparation and underwent FET at the blastocyst stage. On the day of transfer, transvaginal ultrasound examination was performed to evaluate endometrial thickness (ET). The pregnancy was confirmed by β-hcg test (> 25mIU/ml) two weeks after the transfer. Statistical analysis was performed using chi square test (X2), p≤0.005. To evaluate the cutoff (best sensibility and specify of the ET), the ROC curve was utilized.

**Results:** Patient ages ranged from 22-57 years, 34±5.08 years (mean±SD). These patients were divided into pregnant and nonpregnant groups. The variation in endometrial thickness was 4.8-20mm, reaching an average of 8.8mm (SD=2.02). ET was recorded at 0.9 mm intervals and the largest group is in the range of 8-8.9mm (31.88%), followed by 7-7.9mm (19.54%). The β-hcg positive result was 71.47% (cumulative rate) and the highest number of absolute positives was with ET of 7mm (6.9%). The average ET among the positive results was 8.8 (SD=2.0) and among the negative results was 8,9 (SD=1.9). The ROC curve showed a cutoff point for ET of 7mm, with the best sensitivity (20.5) and specificity (89.19) relationship. Based on this cutoff point, it was verified the chemical pregnancy rates between ET ≥7mm and <7mm. In the first, the rate was 70% and second was 83.3% (X2=3.252; *p*=0.0713), a no statistical significance. The percentage of positive βhCG by endometrial thickness was 6-6.9mm (8.22%), 7-7.9mm (13.36%), 8-8.9mm (22.1%), 9-9.9mm (12.34%) and 10-10.9mm (5.4%). The odds ratio analysis showed the respectively results: OR=0.49 (IC=0.106-2.2811), OR=0.47 (IC=0.2414-0.9149); OR=0.93 (IC=0.598-1.4533); OR=1.07 (IC=0.6821-1.7016) and OR=0.81 (IC=0.4738-1.3857).

**Conclusion:** This study did not find statistical significance of chemical pregnancy in different ET. A positive result can be found even at a minimum ET of 4.8 mm. So cancellation of ET based on a thin endometrium appears to be unwarranted. The percentage of positive results reached a peak at a ET of 8.8mm, a value also similar in negative cases (8.9mm). The individualization may be the best management is considerate the history of evolution of the patient's endometrial pattern in other treatments. In conclusion, this study demonstrated did not found relationship between endometrial thickness on the transfer cryopreserved embryos day and pregnancy rates in the ET range studied. Despite the relevant results, concluding the hypothesis was difficult due to the small number of patients with low ET <7mm, requiring further studies, with a larger sample, for this evaluation.

**P-36. Melatonin improves fertilization rate in Assisted Reproduction: Systematic review and meta-analysis**

**Marise Samama^1^, Rita Cassia Camargo Preto Piscopo^1^, Eduardo Veiga^1^, Fabio Ikeda^1^, Suellen Parames^1^, Joji Ueno^1^**

^1^Instituto de Ensino e Pesquisa em Medicina Reprodutiva de São Paulo - São Paulo - SP - Brazil

**Objective:** This study aimed to evaluate the effects of melatonin on assisted reproductive technologies through a systematic review and a meta-analysis.

**Methods:** Search strategies were used in PubMed and in other databases covering the last 15 years. After screening for eligibility, 17 articles were selected for the systematic review. For the meta-analysis statistics, two groups were formed, the treatment group (with melatonin) and the control group (without melatonin) for various assisted reproduction outcomes.

**Results:** The main results were that no statistical differences were found concerning the clinical pregnancy outcome (*p*=0.64), but there was a statistical difference with respect to Mature Oocytes (MII) (*p*=0.001), antral follicle count (*p*=0.0002), and the fertilization rate (*p*≤0.0001).

**Conclusion:** Melatonin had beneficial effects such as the improvement in the fertilization rate, although the authors did not obtain significance in the clinical pregnancy rate.

**P-37. Chronic endometritis in patients with implantation failure in *in vitro* fertilization cycles**

**Suélen Patrícia Pinheiro Machado de Lima^1^, Bernardo Rodrigues Lamounier de Moura^1^, Adrielly Silva de Camargo Stefani^1^, Samira Pedro Orlando^1^, Edson Guimarães Lo Turco^2^, Fernando Prado Ferreira^2^, Lister de Lima Salgueiro^1^**

^1^Clínica Fértilis - Sorocaba - SP - Brazil

^2^UNIFESP - São Paulo - SP - Brazil

**Objective:** To evaluate the prevalence and impact of chronic endometritis, in patients with implantation failure, despite the transfer of high-quality embryos, in *In vitro* fertilization (IVF) cycles.

**Methods:** A cohort of 55 patients, aged 19 to 44 years, undergoing IVF treatment was analyzed. Each patient underwent an endometrial anatopathological biopsy, to assess the presence of chronic endometritis, characterized by inflammation of the endometrial tissue due to infection (virus and bacteria). The first treatment was made with Doxicyclin 100 mg, tree times a day, during 14 days and if necessary, the second treatment with Ciprofloxxacyn 500 mg twice a day plus Metronidazol 400 mg twice a day for 14 days.

**Results:** Out of the 55 patients evaluated, 28 (50.90%) were diagnosed with chronic endometritis. Among these, 12 (42.85%) returned for a second biopsy following the first antibiotic treatment, while 2 (7.14%) did not continue with the treatment. Of the 26 patients who returned, 14 (53.85%) showed satisfactory treatment outcomes and proceeded to a new IVF cycle, whereas 12 (46.15%) required an additional round of antibiotic therapy. Among the 14 patients who underwent a single cycle of antibiotic treatment, 6 (42.86%) achieved pregnancy, while 8 (57.14%) did not. For those with persistent endometritis, 5 (41.67%) became pregnant, and 7 (58.33%) did not.

**Conclusion:** Endometrial anatomopathological biopsy in cases of implantation failure is crucial for diagnosing chronic endometritis. The clinical treatment approach, primarily involving antibiotics, has shown effectiveness, although further studies with larger cohorts are necessary to validate these findings and enhance post-treatment IVF outcomes.

**P-38. A review of the relationship between bariatric surgery and female fertility**

**Letícia Aimí Moraes^1^, Lenara Carvalho^1^, Natalia Fabricio Zanoti^1^, Ana Livia de Oliveira Moreno^1^**

^1^Universidade de Marília - Marília - SP - Brazil

**Objective:** To discuss the relationship between bariatric surgery and female fertility.

**Methods:** An integrative review was conducted in June 2024 using the PICO strategy: Population (P), women who underwent bariatric surgery; Intervention (I), assessment of female fertility post-bariatric surgery; Comparison (C), women who did not undergo bariatric surgery; and Outcome (O), therapeutic outcomes. The central guiding question was: "What is the association between bariatric surgery and female fertility?" PubMed and Latin American and Caribbean Health Sciences Literature (LILACS) databases were searched using MeSH terms and their Portuguese equivalents combined with the Boolean operator AND: "Association", "Bariatric surgery", "Fertility” “Women". Prevalence studies, clinical trials, cross-sectional studies, and prospective studies in Portuguese or English published between 2019 and 2024 were included. Duplicate articles, reviews, and studies not meeting the objective were excluded. Two independent reviewers selected the data and resolved disagreements through discussion or consultation with a third reviewer.

**Results:** Eight studies were selected. Bariatric surgery has been shown to increase conception and fertility rates in women, alongside generally uncomplicated pregnancies. It has proven more effective than medical treatments in inducing spontaneous ovulation in women with obesity and menstrual irregularities, potentially improving their chances of spontaneous fertility. The procedure did not negatively impact live birth rates after *in vitro* fertilization, although there was a decrease in average retrieved oocytes and frozen embryos. Bariatric surgery also leads to significant improvements in menstrual irregularities and maternal outcomes. While fertility and sexual function show improvement, not all gynecological conditions benefit equally from the surgery.

**Conclusion:** Bariatric surgery has shown significant benefits in improving fertility and menstrual irregularities. More research is needed to better understand this relationship.

**References**

Babarinsa *et al*. Best Pract Res Clin Obstet Gynaecol. 2023;90:102382.

Benito *et al*. J Clin Endocrinol Metab. 2020;105:dgaa439.

Christinajoice *et al*. Obes Surg. 2020;30:383-390.

Chang *et al*. Taiwan J Obstet Gynecol. 2021;60:935-937.

Grzegorczyk-Martin *et al*. Hum Reprod. 2020;35:2755-2762.

Legro RS *et al*. J Clin Endocrinol Metab. 2012;97:4540-8.

Nilsson-Condori *et al*. Hum Reprod. 2022;37:2474-2481.

Samarasinghe *et al*. Lancet. 2024;403:2489-2503.

**P-39. Reproductive rights, access to Assisted Reproduction for transmasculine people and trans men in Brazil public policies, medical perspective, experiences**

**Ricardo Nascimento^1^, Olga Regina Zigelli Garcia^1^, Fernando Hellmann^1^**

^1^Universidade Federal de Santa Catarina - Florianópolis - SC - Brazil

**Objective:** It aimed to investigate how public policies and medical practices in Brazil steer reproductive rights and access to assisted reproduction for transmasculine people and how these approaches meet their expectations and needs, regarding the fulfillment of their biological parenthood desires. The study argued that, despite legislative advances and public policies regarding LGBTQIA+ rights in Brazil, transmasculine individuals face significant barriers in guaranteeing their reproductive rights, such as accessing assisted reproduction (AR). The objective was to analyze reproductive rights and access to assisted reproduction for transmasculine individuals in Brazil, encompassing public policies, medical perspectives, and those of transmasculine individuals, including their needs and expectations regarding biological parenthood.

**Methods:** It was adopted a qualitative approach, employing data source triangulation, using including legislative documents and public policies related to reproductive rights within the LGBTQIA+ community, with a focus on trans men. Questionnaires answered by doctors from Assisted Reproduction (AR) clinics and in-depth interviews with trans men and/or transmasculine people, captured following the snowball methodology. Data analysis was conducted using the Discourse of the Collective Subject (DCS) technique.

This study respects the Resolution 510/2016 of the National Health Council, was approved by the Human Research Ethics Committee of the Federal University of Santa Catarina (number 5,775,520).

**Results:** They are outlined in three distinct sections: First, addresses public policies aimed at the LGBTQIA+ population, focusing on transgender issues providing an overview of the Brazilian scenario. The development and implementation of these policies highlight significant gaps that need to be filled, especially with regard to reproductive rights and legal recognition of trans families. Insufficient detail of AR procedures for the trans population; silence on fertility preservation in public policies; disconnection of policies with medical ethical standards; lack of specific clinical protocols; Iack of multidisciplinary monitoring of the issue. The second analyzes the knowledge and practices of medical professionals in assisted reproduction clinics regarding reproductive rights and assisted reproduction for transmasculine individuals and identified that care for trans men, when it occurs in AR clinics is carried out by medical professionals and takes place in the office. Of the 24 professionals interviewed, only one reported serving this group of people frequently, ten rarely and the rest (thirteen) reported never having provided this service. Lastly, the third addresses the knowledge, practices, and demands of transmasculine individuals related to reproductive rights and assisted reproduction, offering a more specific and direct perspective on the experiences and needs of this population. The results revealed progress and gaps in policies and medical practice, highlighting explicit failures in including these individuals in healthcare systems and government policies and the lack of coverage by the SUS (Brazilian government health system) of assisted reproduction for trans men, imply a failure to guarantee their reproductive rights, leading them to sterility.

**Conclusion:** There is an urgent need to reformulate medical curricula and who have clinical experience with the trans population, has to provide specific training for healthcare professionals, and promote structural changes to meet the reproductive needs of this population, emphasizing the importance of educating both health care professionals and the transmasculine community about their rights. These findings underscore the urgency of more inclusive policies to ensure equity in access to reproductive health services for the transmasculine population in Brazil, aiming for universal access and comprehensive care.

**P-40. Study of sperm viability and DNA fragmentation after cryopreservation and storage in a dry shipper for 96 hours**

**Vera Lucia Lângaro Amaral^1^, Vinicius Klabunde^1^, Gabriel Leal Rocha^1^, Ryad Fayez Mehanna^1^, Nicholas Rodrigues Panstein^1^, Alfred Paul Senn^2^**

^1^UNIVALI - Itajaí - SC - Brazil

^2^UNIGE - Switzerland

**Objective:** This study evaluated sperm viability after storing cryopreserved samples in a Dry Shipper container for up to 96 hours, simulating the average transport time of cryopreserved semen to assisted reproduction clinics in Brazil.

**Methods:** 20 seminal samples classified as normal according to World Health Organization (WHO) criteria were used. Cryopreservation was carried out using Ingá Sperm Freezing medium (Ingámed, Maringá, Brazil), packaged in 0.5 mL straws. The samples were refrigerated (6±2°C) for 20 minutes, exposed to liquid nitrogen vapor for 10 minutes, and immersed directly in liquid nitrogen. The straws, in triplicate, were stored for 30 days into three conditions: Control (samples kept in a cryogenic tank), T48 (storage in a Dry Shipper for 48 hours), and T96 (storage in a Dry Shipper for 96 hours). The temperature in the Dry Shipper was kept at -182°C. After thawing at 37°C for 25 minutes, the samples were washed in GV-Hepes medium (Ingámed, Maringá, Brazil) and assessed for DNA fragmentation using the Sperm Chromatin Dispersion (SCD) method, sperm motility and vitality.

**Results:** The results showed that there was no significant difference in the Sperm DNA Fragmentation Index (SDI) between the Control (15.5%), T48 (15.9%) and T96 (14.9%) groups. There was also no statistical difference in the recovery rate of sperm viability, with the following values for sperm motility and vitality: Control - 56.5% and 66.9%; T48 - 55.6% and 64.4%; T96 - 53.4% and 62.6%. However, all the samples from the test and control groups had a significant drop (*p*<0.001) in motility (47%) and sperm vitality (63%) after thawing, compared to the fresh samples.

**Conclusion:** Cryopreservation negatively affects semen quality; however, there is no evidence that the Dry Shipper device influences sperm DNA fragmentation, sperm motility, and vitality for up to 96 hours of storage. Further studies are needed to determine how the Dry Shipper behaves in real-world transport conditions and which uncontrolled events might still affect transport safety and sperm viability.

**P-41. Cesarean scar pregnancy with live birth after In Vitro Fertilization and frozen embryo transfer: A case report**

**Kenya Gastal Figueiredo^1^, Patricia Florido^1^, Edmund Chada Baracat^1^, Pedro Augusto Monteleone^1^**

^1^Hospital das Clínicas da Faculdade de Medicina da Universidade de São Paulo - São Paulo - SP - Brazil

**Objective:** Cesarean scar pregnancy (CSP) is defined as an ectopic pregnancy implanted in the myometrium of a previous cesarean section scar. Although the incidence of CSP is extremely low, estimated at 1:2216 to 1:1800, it can lead to catastrophic complications. Considering its high morbidity, the most usual recommendation has been the termination of pregnancy in the first trimester; however, several cases progress to viable births. Here, we present a case report of a woman diagnosed with na ectopic pregnancy in a cesarean section scar after *in vitro* fertilization (IVF) with expectant management resulting in a live birth.

**Methods:** Case Report.

**Results:** S. S. L., a 36-year-old woman with a history of three previous cesarean sections, underwent tubal ligation during her last birth, seven years before being referred to our service for IVF. A small isthmocele was identified during her follow-up. She underwent her first IVF cycle with five oocytes recovered and the formation of three embryos, resulting in a topical pregnancy with spontaneous abortion at six weeks of gestational age after the last embryo transfer. In the second IVF cycle, thirteen oocytes were recovered and two embryos were obtained. A double-embryo transfer (blastocyst morphological score 4CB/4BB) was performed, evolving with a single pregnancy and the presence of a gestational sac visualized on ultrasound in the region of the cesarean section scar. The patient was referred to the institution’s high-risk prenatal care, where expectant management was adopted. During the follow-up, placenta percreta was diagnosed, and the pregnancy was resolved at 34 weeks of gestation via cesarean section followed by subtotal hysterectomy. The surgery was performed by a multidisciplinary team, including obstetrician, urologist, and general surgeon. The only complication reported was an iatrogenic lesion of the bladder due to placenta adhesion. The newborn weighed 2804 grams. Cesarean delivery is a safe surgery and route of birth. It can reduce mortality rates among mothers and newborns and plays an important role in reducing maternal and perinatal infant mortality and morbidity in complicated vaginal births. However, due to several iatrogenic and social factors, the global rates of C-section have increased markedly in recent years, and complications such as CSP are becoming more common. Some studies have observed that the rate of CSP after IVF-ET in patients with a previous cesarean section may be higher than in spontaneous gestations, suggesting that IVF could contribute to the increase of this phenomenon. Regarding the presence of isthmocele on examination, there is still no consensus on whether it should be corrected before embryo transfer.

**Conclusion:** For infertile patients with a history of previous cesarean section and the identification of an isthmocele, if they wish to have another child through IVF, it is important to receive counseling before treatment is initiated. Single embryo transfer should be recommended. There is still no standardized treatment for CSP, but it must be analyzed with caution due to the high burden of maternal complications.

**P-42. The effects of activated platelet rich plasma on human endometrial mesenchymal stem cell proliferation and receptivity markers**

**Gabriela Moura^1^, Pamela Zanon^1^, Ivan Montenegro^1^, Paula Terraciano^1^, Markus Berger^1^, Eduardo Passos^1^**

^1^Hospital de Clínicas Porto Alegre - Porto Alegre - RS - Brazil

**Objective:** Endometrial receptivity is one of the keys to achieve better results in assisted reproductive technique cycles. The use of activated platelet-rich plasma (aPRP) to enhance endometrial receptivity is gaining attention with positive experimental results showing improved pregnancy and birth rates, especially in patients with poor endometrial growth. However, the cellular and molecular mechanisms involved are not clear. This study aims to determine the main components in aPRP and its effects on human endometrial mesenchymal stem cell (hEMSC) proliferation and receptivity markers.

**Methods:** Endometrial tissue was harvested from healthy patients undergoing biopsy for *in vitro* fertilization procedures at a tertiary hospital in Brazil. hEMSCs were isolated and characterized according to their morphological and immunophenotypical features. PRP was obtained from healthy donors, activated with human thrombin and its composition was evaluated by mass spectrometry (MudPIT proteomics). hEMSCs exposed to different aPRP concentrations (1-5%) were evaluated according to proliferation, viability and expression of receptivity biomarkers.

**Results:** The isolated hEMSCs in culture showed adhesiveness properties, presented a fusiform fibroblastoid morphology and ability to *in vitro* differentiate into adipocytes, osteocytes and chondrocytes. Cells demonstrated positive glycosaminoglycan production after 12 days in culture, as indicated by Alcian blue staining. Under osteogenic differentiating conditions, cells showed a calcium-rich mineralized matrix that was evident after 14 days in culture, as indicated by Alizarin Red S staining. Similarly, the formation of intracellular lipid vacuoles was observed after 21 days, as was confirmed by Oil Red staining. The expression of cell surface markers was also characterized by flow cytometry. hMESCs were positive for mesenchymal markers (CD105, CD90 and CD73) and negative for hematopoietic markers (CD45 and CD11), indicating that isolated cells have potential for multilineage differentiation. The aPRP MudPIT proteomic analysis identified 252 proteins, being the proteome enriched in proteins related to response to stress, immune system process, cytoskeletal proteins and defence/immunity proteins. Proteins such as hemopexin, hepatocyte growth factor, transforming growth factor-beta, insulin-like growth factor binding protein, epidermal growth factor, fibronectin, vinculin and clusterin were all identified with significant spectral count numbers. Currently, experiments are under way to determine the proliferative, migratory and survival mechanisms induced by aPRP in hEMSCs. Hox gene-expression, estrogen and progesterone receptors also will be evaluated.

**Conclusion:** hEMSCs were successfully isolated displaying classical stem cell features. aPRP composition was enriched in growth factors and immune modulators that can potentially enhance endometrial proliferation and survival abilities.

**P-43. Access to Assisted Reproduction through the Brazilian Unified Health System: An integrative literature review**

**Luísa Duarte Weissheimer^1^, Elisa Grotto^1^, Júlia Salles Monticelli^1^, Maria Beatriz Albani Rossoni Teza^1^, Sara Thomas Horn^1^, Thaís Prestes Piccinini^1^**

^1^UFCSPA - Porto Alegre - RS - Brazil

**Objective:** Assisted reproduction (AR) in Brazil started in 1984, and only in 2005 was the National Policy for Comprehensive Care in Assisted Human Reproduction developed, which guarantees citizens the right to assisted reproduction techniques in the Unified Health System (SUS). However, the process to guarantee this right is still very slow, rigorous, and with few resources, which makes these procedures restricted to patients who can afford to pay for their treatment in private clinics. In view of this, this study aims to understand the scientific evidence on the impasses that lead to difficulty in accessing AR through the SUS.

**Methods:** This is an integrative review of the literature, between May and June 2024, in which articles were searched in the PubMed and Scientific Electronic Library Online (SCIELO) databases, using the descriptors “infertility”, “public health system” and “Brazil”. It was decided not to use the time filter, since this theme has few materials produced and AR by the SUS has not undergone significant changes since its inception.

**Results:** On the PubMed platform, 29 results were found, but only 3 were related to the present study. On the other hand, on SCIELO, 4 results were obtained, of which 3 were consistent with the subject. The articles researched show that the main obstacles to accessing AR through the SUS are the high demand, and the resulting waiting list for treatment, the costs of medications and exams, in addition to the inclusion and exclusion criteria for patients. Therefore, it is observed that this problem is generated by the lack of legislation regarding these techniques and by the State's lack of attention in guaranteeing the right to conception of Brazilians, since infertility is often not considered a health problem by the Public Authorities. In 2021, the waiting time for a gynecological consultation to investigate infertility exceeded 200 days, which increased the risk of women entering the high-risk pregnancy period and no longer being able to undergo treatment. A survey conducted with 80 infertile couples seeking treatment in 2020 showed that they were committed to paying 2 to 4 times their monthly income to fulfill their dream of starting a family. Furthermore, the number of public AR centers is significantly lower than the number of private ones, and they are located only in metropolitan areas and are not completely free. This is due to the fact that the government does not allocate sufficient resources to meet this public demand, since in 2012 alone R$10 million was allocated to the centers. However, one of the articles analyzed in this study estimated a budget forecast for implementing and maintaining an AR center in the state of Rio de Janeiro, reaching a cost of approximately R$16 million for 480 procedures performed per year.

**Conclusion:** The review revealed a lack of scientific evidence, as few articles were found, most of which were very old. However, it is also clear that, when analyzing these studies, the difficulty of accessing AR through the SUS is also a reality in 2024, and the reasons for these impasses remain the same, mainly due to the lack of public investment. The high cost of treatment prevents many couples from realizing their dream of starting a family, since the State does not guarantee the right to conception provided for in the Federal Constitution, which makes these procedures elitist. Therefore, reversing the absence of legislation and encouraging research in this area are fundamental ways to guarantee this right to Brazilians.

**P-44. Genetic screening in sperm donor candidates in Rio Grande do Sul: A retrospective study**

**Franciely Machado Ramos^1^, Roberta Mayer Evangelista^1^, Bibiana Mello Oliveira^2^, Tayssa Kruger Almeida^1^, Ana Paula Kussler^1^, Bruna Guaraná^1^, Letícia Schmidt Arruda^1^**

^1^Vida Fértil - Banco de Sêmen - Porto Alegre - RS - Brazil

^2^Santa Casa de Misericórdia de Porto Alegre - Porto Alegre - RS - Brazil

**Objective:** To evaluate the impact of genetic screening on the selection of candidates for sperm donation in the State of Rio Grande do Sul (RS), Brazil.

**Methods:** A retrospective evaluation of the results of Genetic Screening (next-generation sequencing method [NGS], Invitae Laboratory, USA) used as one of the screening steps for sperm donor candidates, was performed in a sperm bank, located in Porto Alegre, RS, Brazil, between the years of 2020 and 2024. A total of 36 results from 36 candidates aged between 18 and 40 years were analyzed. All of them had been approved in the previous stages of screening: 1) individual and family medical history; 2) serological and microbiological tests; and 3) seminal quality parameters. Of the 36 candidates, 6 had 288 genes (P288G) evaluated, 21 had 289 genes (P289G) evaluated and 9 had 556 genes (P556G) evaluated. According to the variant(s) found, the candidate was classified as "accepted" or "rejected" for the sperm donation process, considering the international recommendations of the American Society for Reproductive Medicine. From the results, we calculated the prevalence of pathogenic variants of sperm donation candidates, the average number of pathogenic variants per sperm donation candidate with at least one pathogenic variant detected, elimination rate of candidates (classified as 'rejected') and the most frequently encountered genes with pathogenic variants.

**Results:** The prevalence of candidates with at least one pathogenic variant detected was 83.33% (30/36). An upward trend in the prevalence of candidates with at least one pathogenic variant was detected as the number of genes evaluated increased: 66.67% (4/6), 80.95% (17/21) and 100.00% (9/9) for candidates who performed the P288G, P289G and P556G tests, respectively. Among the 30 candidates who had at least one pathogenic variant detected, the average number of pathogenic variants found was 2.03 per candidate. The rate of donors classified as 'rejected' (eliminated) due to genetic screening was 22.22% (8/36). Among the genes evaluated in all panels, the one with the most frequently identified pathogenic variants was GJB2, related with hearing loss, with 16.67% (6/36), followed by survival motor neuron (SMN1), cystic fibrosis transmembrane conductance regulator (CFTR) and galactose-1-phosphate uridyltransferase (GALT) genes, all with a frequency of 8.33% (3/36).

**Conclusion:** As expected, we observed that the greater the number of genes evaluated in the panel, the higher the rate of identification of variants, so it is essential to discuss and have recommendations from international societies on which panel size is the most appropriate for screening sperm donors. Genetic selection for sperm donors involves the evaluation of the candidate's personal and family history before performing a Genetic Screening and, since monogenic pathologies are excluding, it is suggested that the positivity rate in the general population is higher than the one found in this study. According to international guidelines, pathogenic variants of high prevalence in the population that are correlated with diseases of severe clinical manifestation, many of which even have a high infant mortality rate, should be a criterion for exclusion of donors in a gamete bank. In this context, variants in the SMN1 and CFTR genes, for example, are among the most prevalent in this study. Thus, a high rate of donors is considered "rejected" and consequently excluded from the donation process because they have exclusionary pathogenic variants. Therefore, this result reinforces the importance of gamete banks performing this type of Genetic Screening in the selection of donors in order to provide greater security to the recipients of the sperm samples.

**P-45. Key performance indicators score based on clinical and laboratorial parameters through Business Intelligence Analysis in ART program**

**Flavia G Gomes^1^, Magnus R Gomes^1^, Angela M D’Avila^1^**

^1^Embrios Centro de Reprodução Humana - Bento Gonçalves - RS - Brazil

**Objective:** To establish a key performance indicator (KPI) control program for clinical work in ART, using the Business Intelligence data analysis tool, which makes it possible to create competency profiles for doctors, embryologists and for each specific stage of the process

**Methods:** To control the performance, we apply a range of KPIs for different steps of the ART process, starting from fertility workup and diagnosis, extending to clinical management, laboratory manipulation and ending with o the clinical results. We then use Microsoft Power Business Intelligence, which has an infinite data warehouse for analyzes and dashboards, to analyze the results among the main KPIs of specific cenarious. For example, the live birth rate for patients with a certain infertility factor, undergoing embryo transfer in the chosen embryonic development phase and endometrial preparation, comparing the result between different medical and laboratory operators. Still in this scenario, it would also be possible to analyze the different results by comparing body mass index, progesterone dosage and endometrial thickness. This allows you to make better choices of treatment and assess the prognosis of the case even before starting it. The KPIs chosen were: oocyte recovery, maturation, fertilization, degeneration, cleavage, blastocyst formation, top quality embryo, embryo survival, pregnancy, ongoing, implantation rate, abortion and biochemical pregnancy rate. The metrics to be analyzed were: the patient's age, donor's age, primary infertility factor, secondary infertility factor, seminal origin, patient's BMI, donor's BMI, embryonic morphology, antral follicle count (AFC), Antimullerian Hormone dosage, Blocking Medication, Stimulus Medication, Trigger Medication, Progesterone Dosage at Transfer, Endometrial Thickness at Transfer, Stimulus Physician, Transfer Physician, ICSI Embryologist, Freezing Embryologist, Thawing Embryologist, Transfer Embryologist, Media cultivation, freezing medium, and thawing medium. Through Power B.I it is possible to have the result of KPIs comparing all desired analysis metrics.

**Results:** The complexity of the ART process is reflected in the large amount of data that can be generated from a single cycle. The systematic monitoring of KPIs through Power BI became part of the total quality management system of the establishment in which it was implemented and gained interest in clinical practice as with its use it is possible to focus on patient-centeredness and individualization of their treatment, in addition to evaluating the team's competence in real time and providing patients with transparent information before, during and after their treatment.

**Conclusion:** A business intelligence (BI) tool in a laboratory workflow offers various benefits, including data consolidation, real-time monitoring, process optimization, cost analysis, performance benchmarking (quality indicators), predictive analytics, compliance reporting, and decision support. These tools improve operational efficiency, quality control, inventory management, cost analysis, and clinical decision-making, with the analysis of the reality of the center itself. By identifying challenges and overcoming them, laboratories can utilize the power of BI and analytics solutions to accelerate healthcare performance, lower costs, and improve care quality. Business intelligence dashboards are one of the key components of data analytics since they provide decision makers timely access to summarized analyses and visualizations. Dashboards typically display the status of KPIs and other metrics or summary statistics on a single screen, providing information for specific objectives at a glance. These BI dashboards can enhance healthcare organizations’ financial and operational performance and quality of patient care. These BI tools allow us to remove manual steps done using Excel worksheets, for example data handling, using excel formulas and generating visual graphical works.

**P-46. Microfluidics sperm sorting improves fertilization rate after intracytoplasmic sperm injection**

**Luiza M Donatti^1^, Flavia G Gomes^1^, Angela M D’Avila^1^**

^1^Embrios Centro de Reprodução Humana - Bento Gonçalves - RS - Brazil

**Objective:** Since a high sperm DNA fragmentation index (DFI) is an important male infertility factor and sperm selection with microfluidics devices overcome these losses, the main aim of this study was to compare laboratory outcomes after sperm preparation with microfluidics sperm sorting devices (ZyMot) and after sperm selection with centrifugation techniques (density gradient centrifugation or sperm washing).

**Methods:** A retrospective study was performed with 23 oocyte donation cycles from January 2023 to April 2024 in a single reproductive medicine center. Cycles included in this study were divided into two groups according to the sperm preparation technique. Centrifugation techniques were utilized for the semen preparation of the control group (n=9) while the ZyMot device was used to prepare semen samples from the zymot group (n=14). All semen samples were collected by masturbation, and five samples from the control group were cryopreserved before use. Oocyte fertilization was made with intracytoplasmic sperm injection (ICSI) in all cycles and embryos were cultivated until day 6. Laboratory outcomes (fertilization rate, blastocyst formation, and blastocyst utilization rate) and semen parameters (pre-processing concentration and preand post-processing motility) were compared among groups. One-way ANOVA was applied to compare the groups using Graphpad Prism and p-value ≤ 0.05 was considered statistically significant.

**Results:** The oocyte donor’s age did not differ among the groups (*p*=0.818). The zymot group presented a significantly higher fertilization rate after ICSI compared to the control group (92.9% *vs*. 70.2%, *p*=0.0078). Other laboratory outcomes evaluated in this study did not differ among the control and the zymot group, respectively: blastocyst formation rate (74.2% *vs*. 56.7%, *p*=0.068) and blastocyst utilization rate (93.3% *vs*. 91.4%, *p*>0.9999). Data analysis for semen parameters showed a statistically significant higher pre-processing motility for the zymot group (62.5% *vs*. 42.3%, *p*=0.0314) but pre-processing sperm concentration did not differ amid the zymot and the control group (59.5x106 vs. 49.5x106, *p*=0.6521). Significantly higher motility was observed in the zymot group after semen processing compared to the control group (87.8% *vs*. 46.4%, *p*=0.0085).

**Conclusion:** It is known that high sperm DFI negatively affects male fertility and *in vitro* fertilization (IVF) cycle outcomes. Available literature emphasizes the negative impact of high sperm DFI on the fertilization rate, the euploidy rate, and increasing in the miscarriage rate. Semen processing before ICSI aims to select the best sperm for fertilization but centrifugation techniques may induce sperm DNA breaks and increase the sample DFI. Microfluidics devices seem to avoid these damages to sperm DNA and select sperm with lower sperm DFI for ICSI and, consequently, improve IVF cycle results. Our study showed that sperm selection with microfluidics sperm sorting devices increased fertilization rate after ICSI although it did not interfere with other laboratory outcomes analyzed. A limiting factor is the small N of our research but cycles included in this study will be followed up to analyze and compare live birth rates between the groups. Clinical results, such as clinical pregnancy and live birth rates, were not included in this study however are also important factors in evaluating the impact of seminal preparation with microfluidic devices in IVF cycle outcomes. Our results suggest semen processing with microfluidics devices could be applied in IVF cycles to achieve better laboratory results, mainly in cases with historically high sperm DFI samples.

**P-47. Counseling patients with monogenic diseases: Insights from an 8-year follow-up study of preimplantation genetic testing for monogenic disorders (PGT-M)**

**Aline Rodrigues Lorenzon^1^, Ricardo Ceroni^1^, Ana Luiza Nunes^1^, Claudia Gomes^1^, Thais Sanches Domingues^1^, Dóris Spinosa Chéles^1^, José Roberto Alegretti^1^, Eduardo Leme Alves Motta^1^, Mauricio Barbour Chehin^1^**

^1^Huntington Medicina Reprodutiva - Eugin Group - São Paulo - SP - Brazil

**Objective:** Preimplantation genetic testing for monogenic disorders (PGT-M) offers to couples with family history of genetic diseases the opportunity to avoid the birth of an affect child. It is fundamental that patients seeking for *in vitro* fertilization (IVF) treatments for PGT-M have a proper genetic counseling regarding their condition and a perspective of what they should expect in terms of reproductive outcomes in this type of treatment and the limits of this technique should be discussed. The objective of this study was to analyze the main outcomes of PGT-M cases in the last 8-years of practice in a private IVF center.

**Methods:** This database screening included patients that underwent an IVF treatment for preimplantation genetic testing for monogenic disorders (PGT-M) purpose between July/2016 and March/2024. Most common diseases, maternal and paternal age, number of cycles for oocyte retrieval, total number of oocytes retrieved, number of mature oocytes, number of blastocyst available for testing, PGT-M result and reproductive outcomes were evaluated. Comparison between treatments that achieved a clinical pregnancy/live birth from negative pregnancy or no embryos available for embryo transfer were also analyzed. Kruskal-Wallis with Dunn’s multiple comparisons test were applied for statistical analysis, values of *p*<0.05 were considered significant.

**Results:** One hundred-seven couples carried out PGT-M cycles during this period. The most prevalent monogenic disorders were: Sickle Cell Anemia (HBB, 17.4%), Cystic Fibrosis (CFTR, 12.2%), Huntington Disease (HTT, 8.7%), Fragile X Syndrome (FMR1, 6.1%) and Congenital Adrenal Hyperplasia (CYP21A2, 5.2%). In total, 210 cycles for oocyte retrieval were done, in a mean of 1.8±1.2 cycle/patient. Maternal and paternal mean age were 35.4±3,8 and 38.1±4.9 years. Mean of oocytes retrieved were 22.6±16.2 and mature oocyte 16.9±11.2 (mean MII rate: 75%). Mean number of blastocysts for analysis were 7.0±4.5. One-hundred fifteen cycles were considered for reproductive outcomes analysis. The majority achieved a suitable embryo for transfer (92/115, 80%). From those, 53 (57.6%) resulted in clinical pregnancy/live birth, 22 (23.9%) in a negative pregnancy, 1 miscarriage (1%) and in 16 cycles the embryos are still frozen (17.4%). In 23 cases, there was no suitable embryo for transfer (20%). From those, 16 (69.5%) were for absence of an euploid embryo, 6 (26%) for absence of a normal/carrier embryo and 1 (4%) for no HLA-compatibility. A comparison of cycles that achieved a clinical pregnancy/live birth with those that resulted in a negative result and no embryo available for transfer showed that patients that achieved a successful outcome had lower maternal age (34.3±4.1), higher number of oocytes retrieved (25.1±15.7), mature oocytes (18.2±9.5) and blastocysts for analysis (8.0±4.2). In contrast, patients in the group with no embryo for transfer showed higher maternal age (37.1±3,6, *p*=0.02), lower number of oocytes retrieved (16.2±10,3, *p*=0.03), mature oocytes (11.9±6.2, *p*=0.02) and blastocysts for analysis (4.3±2.9, *p*=0.0003). No differences were observed between the clinical pregnancy/live birth and negative pregnancy groups.

**Conclusion:** Our results highlight the urgent need for counseling couples with a history of monogenic diseases to consider IVF with PGT-M treatment as early as possible, given that maternal age is the strongest predictor of treatment success. Our screening showed that 80% of cycles resulted in a suitable embryo for transfer. However, in cases where no suitable embryo was available, approximately 70% were due to the absence of a euploid embryo. Other significant variables, such as the total number of oocytes retrieved, number of mature oocytes, and number of blastocysts available-greater than 8.0-are all intrinsically dependent on maternal age. For couples wishing to postpone parenthood, egg freezing should be advised to increase their chances of a successful reproductive outcome in the future.

**P-48. Severity of oligozoospermia and pregnancy outcomes at Intracytoplasmic injection of spermatozoa (ICSI)**

**Paulo Crespo Ribeiro Neto^1^, Luciane Pansardi Cabreira Baptista^1^, Talita Giacomet de Carvalho^1^, Andrea Prestes Nacul^1^**

^1^Hospital Fêmina - Porto Alegre - RS - Brazil

**Objective:** Infertility is a multifactorial condition that grows every day due to the changes evident in society over the decades, becoming a public health issue in many countries. Among the main causes, male factor stands out, responsible for 35% of infertility causes, according to the World Health Organization. Among these, oligozoospermia is defined as a low sperm count in seminal analysis. Intracytoplasmic injection of spermatozoa (ICSI) is the treatment of choice for male infertility. The present study aims to evaluate gestational outcomes (fertilization rate, cleavage rate, HCG+, ongoing pregnancy and live births (LB) in 3 different groups of oligozoospermia, based on their severity, in patients undergoing ICSI, in a public service of human reproduction, between january, 1994 to december, 2022.

**Methods:** The oligozoospermia severity was classified in mild (<15-10 millions/ml), moderate (<10-5millions/ml) and severe (<5 millions/ml). It was a cross-sectional study, with analysis of medical records from the “Cryolife” program, including 102 couples presenting among the causes of infertility, oligozoospermia.

**Results:** The fertilization rate was significantly higher in the mild (99.5%) and moderate (96.3%) groups, comparing with the severe group (85.8%) (*p*<0.001; p=0.008, respectively). The cleavage rate did not differ significantly between the groups. When comparing the HCG, ongoing pregnancy and LB outcomes, the three groups did not differ significantly (*p*=0.103, *p*=0.070 and p=0.070 respectively), but there was a tendency of higher percentages in ongoing pregnancy and LB in the mild group in relation to the others.

**Conclusion:** Improvement of seminal parameters before ICSI been performed could be a good strategy to increase pregnancy outcomes. More studies evaluating therapeutic interventions for male infertility before ICSI should be arranged.

**P-49. The impact of lactate concentration on human embryo development: Comparison of two culture media**

**Karen Melissa Gonçalves Oliveira^1^, Aline Cássia Azevedo^1^, Manuela Baldave Carli Dias^1^, Lilian Pagano Mori^1^, Vitor Armênio Scontre^1^, Mariana Schmidt Vieira^1^, Juliana Assi^1^, Jeniffer Monteiro Portes Lima^2^**

^1^InVentre Centro Avançado de Medicina Reprodutiva - Santo André - SP - Brazil

^2^InVentre Centro Avançado de Medicina Reprodutiva - São Paulo - SP - Brazil

**Objective:** To analyze if lower lactate concentration in human embryos culture medium is associated with a higher blastocysts rates and embryo quality.

**Methods:** A prospective observational cohort study of sibling oocytes divided as equally as possible between two culture media (CSCM-C and CSCM-NXC, both Fujifilm Irvine Scientific). After intracytoplasmic sperm injection (ICSI), the culture of these embryos was performed in the same petri dish in order that dishes, oil, and incubators were the same to reducing study biases. The parameters compared between the two culture media were: fertilization rate, blastocyst rate (day 5 and day 6), and quality embryo. Morphokinectic parameters were measured using is Gardner classification system for blastocyst quality e subsequently divided into two groups: Top Quality (embryos classified as AA, AB, BA, BB) and Poor Quality (embryos classified as AC, BC, CA, CB, CC). Only embryos that reached expansion levels 3, 4, 5 and 6 were considered. For statistical analysis, the non-parametric Wilcoxon test and 2-way ANOVA were performed using SPSS 26 software. The graphs were constructed using GraphPad Prism 10 software.

**Results:** Seventy *in vitro* fertilization cycles were included in the study, and a total of 511 correctly fertilized embryos (2PN on day 1) were allocated and observed in the two types of culture media (265 in CSCM-C and 246 in CSCM-NXC). The fertilization rates found in the CSCM-C and CSCM-NXC groups were 87.1% and 91.8% respectively, with no statistically significant difference. Regarding the rate of viable blastocysts in each group, the rate in the CSCM-C was 56.2%, while that in the CSCM-NXC was 67.6%, a higher margin of 11.4%. These data were statistically significant (*p*=0.041). Comparing the embryonic quality between the groups, the data showed no significant difference between the culture media when analyzing Top Quality and Poor Quality (*p*=0.261).

**Conclusion:** This study demonstrated that culture medium with lower lactate concentration is possible to achieve a higher quantity of blastocysts on D5 and D6, however, it does not directly impact the quality of the embryos formed when compared to conventional culture medium. Although fertilization rates and quality embryonic did not indicate statistical differences, the results with blastocyst rates are relevant to provide a better understanding of lactate roles in embryonic development. These findings, along with new studies, will contribute to improve the knowledge and technologies development in human assisted reproduction.

**P-50. Relative quantification of Mitochondrial DNA versus Aneuploidies: A retrospective and comparative study**

**Cintia Pimentel Mangueira Teixeira^1^, Maria Luiza Silva Ricardo^1^, Wilson Jaccoud^1^, Anna Clara Rodrigues Petermann Bugalho^1^**

^1^Fert-Embryo Centro de Medicina Reprodutiva - Presidente Prudente - SP - Brazil

**Objective:** The aim of this study is to assess the correlation between the relative quantification of mitochondrial DNA and numerical chromosomal abnormalities in embryos undergoing Preimplantation Genetic Testing for Aneuploidy (PGT-a).

**Methods:** Three hundred and twenty-nine blastocyst-stage embryos, with an average maternal age of 37.5 years, underwent trophectoderm biopsy for PGT-a between 2021 and 2023. Next-generation sequencing (NGS) was utilized for genetic analysis, and the reports included values for relative mitochondrial DNA (mtDNA) quantification. This value represents the ratio of mtDNA to autosomal DNA, calculated based on the mitochondrial and genomic (autosomal) DNA sequences obtained during the NGS assay. The embryos were categorized as euploid (n=134) or aneuploid (n=195); mosaic embryos were excluded from the study. The mtDNA values were subjected to the Shapiro-Wilk normality test, confirming non-parametric distribution. Subsequently, the significance between the groups was assessed using the Mann-Whitney test with Prism 5.0 software (GraphPad), with significance set at *p*<0.05.

**Results:** The mean mtDNA ratio in euploid embryos was 0.001475, while it was 0.001830 in aneuploid embryos. These averages exhibited a statistically significant difference (*p*=0.0067), indicating that aneuploid blastocysts harbored notably higher levels of mtDNA compared to their euploid counterparts.

**Conclusion:** The findings of this investigation are corroborated by empirical evidence, illustrating that biopsy specimens from aneuploid blastocysts contain substantially elevated levels of mtDNA in contrast to those from euploid embryos. The underlying mechanisms driving this elevation remain ambiguous. It may stem directly from deficiencies affecting the organelle, thereby disrupting energy production or other essential functions. Alternatively, the increased mitochondrial content and aneuploidy might represent independent consequences, indicative of an underlying, yet undefined, issue affecting the embryo or oocyte. Consequently, further studies are warranted to elucidate the relationship between mtDNA quantity and aneuploidies, with the aim of integrating this parameter into the criteria for embryo selection prior to transfer.

**P-51. Psychoeducation as a method for addressing sexual diversity**

**Cássia Cançado Avelar^1^, Cristiane Araújo Oliveira^1^, Erica Becker^1^, Ricardo Mello Marinho^1^, João Pedro Junqueira Caetano^1^**

^1^Clínica Huntington Pró-Criar - Belo Horizonte - MG - Brazil

**Objective:** Human sexuality is basically composed of three elements: biological sex, sexual orientation, and gender identity. Currently, there are different gender identities and sexual orientations, characterized as LGBTQIAPN+ (Lesbian, Gay, Bisexual, Transgender, Queer, Intersex, Asexual, Aromantic, Agender, Pansexual, Polysexual, Non-binary, and others). And these patients are coming to assisted reproduction clinics to have children. Objective: Evaluate the understanding of sexual diversity among clinical staff and employees at an assisted reproduction clinic.

**Methods:** A questionnaire was created with questions regarding the care of LGBTQIAPN+ individuals, aimed at clinical staff (n=37) (Doctors, Nursing, Embryology, Psychology, and Pharmacists) and employees (n=24) (Reception/Administrative, Financial, General Services, and others) in an assisted reproduction clinic in Belo Horizonte, MG, in August 2023.

**Results:** 1) The first question asked was "Characterize in one sentence what you understand by sexual diversity." The results for clinical staff were: Sexual orientation; Inclusion; Respecting people's choices and options, especially sexual ones; Sexuality is part of an individual's development; We must respect sexual diversity; Gender diversity; Empathy; I have two mothers; Various forms of relationships and prefer not to comment. For employees: Different forms of love; Respect is everything; Sexual orientation; The freedom to be who you are; and many left it blank. 2) Regarding their stance towards LGBTQIAPN+ people, 62% of clinical staff were neutral; 35% were favorable and 4% were against. For employees, 57% were neutral; 39% were favorable and 8% were against. 3) On the question, "Do you consider yourself prepared to attend to LGBTQIAPN+ patients?", 89% of clinical staff said yes and 11% said no. And 100% of the employees said yes. 4) Another question was, "Have you ever attended to an LGBTQIAPN+ patient?", to which 100% of clinical staff and 78% of the employees responded yes. 5) "Did you have difficulty in approaching LGBTQIAPN+ patients?", 19% of clinical and 14% of the employees said yes. 6) And the last question was, "Did you receive training before attending to LGBTQIAPN+ patients?", 78% of clinical staff and 95% of the employees said no, and 55% of clinical staff and 37% of the employees said they needed training.

**Conclusion:** After this survey, psychoeducation work was carried out with clinical staff and employees on attending to LGBTQIAPN+ patients, considering that all professionals must have an ethical commitment to attend and welcome LGBTQIAPN+ patients in the most humane and respectful way. Being well-informed is a first step towards this.

**P-52. Reflection of Preimplantation Genetic Testing for Aneuploidies (PGT-A) on Pregnancy Rates in women aged between 35 and 40 years**

**Cintia Pimentel Mangueira Teixeira^1^, Maria Luiza Silva Ricardo^1^, Wilson Jaccoud^1^, Anna Clara Rodrigues Petermann Bugalho^1^, Giovanna Marcela Barbosa^1^**

^1^Fert-Embryo Centro de Medicina Reprodutiva - Presidente Prudente - SP - Brazil

**Objective:** To assess the interference of Preimplantation Genetic Testing for Aneuploidies (PGT-a) on pregnancy rates in women aged 35 to 40 undergoing *In Vitro* Fertilization treatment.

**Methods:** A retrospective cohort study involving 78 patients with a mean age of 37 years undergoing Assisted Reproduction treatment at a Human Reproduction clinic in the West of São Paulo in the years 2022 and 2023. Patients were divided into two groups: Control: without embryonic biopsy and without PGT-a (n=47) and PGT-a: embryonic biopsy of the trophectoderm at the blastocyst stage, followed by PGT-a (n=31). Embryonic transfer followed the pattern of the most favorable morphology in the control group and euploid result, followed by the most favorable morphology in the PGT-a group. Pregnancy detection was performed using a quantitative beta hCG test between 10 to 12 days after transfer, indicating positive or negative results. The difference in variables between the two groups was statistically analyzed using Pearson's Chi-square test. The analysis was conducted using R software version 4.3.0 (Free Software Foundation), with a significance level of *p*<0.05.

**Results:** Regarding beta hCG results, the control group had 26 positives and 21 negatives, while in the PGT-a group, there were 13 positives and 18 negatives. There was no statistically significant difference in pregnancy rate between the experimental groups (*p*=0.2615).

**Conclusion:** The use of trophectoderm biopsy at the blastocyst stage and PGT-A did not significantly interfere with pregnancy outcomes. This result is consistent with scientific evidence that also did not observe differences in pregnancy rates in patients who underwent PGT-a compared to patients who followed only morphological criteria for embryo transfer.

**P-53. Live Birth Rate following transfer of Low-Grade Mosaic embryos: A literature review**

**Vitória Luiza Brogiato^1^, Janine Maíra Barbosa^1^, Victoria Barchi Cordts^1^, Mariana Kasuga Morya^1^, Stephanie Tasseli Alencar Assunção^1^, Gabriel Monteiro Pinheiro^1^**

^1^Universidade Santo Amaro - São Paulo - SP - Brazil

**Objective:** The objective of this study is to evaluate the live birth rate following the transfer of embryos classified as low-grade mosaics by PGT-A.

**Methods:** This is a literature review based on articles fully available for reading, published in the last five years on the PubMed platform, limited to the following languages: Portuguese, English, and Spanish. The research was conducted using the following keywords: “low grade mosaic and embryo,” “transfer low grade mosaic,” “PGT-A embryo and mosaic,” “PGT-A,” and “mosaic embryos.” The search yielded 135 articles, of which 40 were selected for use in this study after a critical review.

**Results:** The study of genetic diseases in *in vitro* fertilization cases has become increasingly common, as genetic analysis significantly enhances discoveries about the embryo being formed. Among the study possibilities is preimplantation genetic testing for aneuploidies (PGT-A), which, through numerical analysis of embryonic chromosomes, classifies embryos based on the percentage of genetically altered cells. Embryo mosaicism is a condition in which the embryo’s cells have different genetic compositions, containing between 30% and 50% genetically altered cells. The evaluation of embryo mosaicism is crucial in assisted reproduction techniques such as PGTA-A, as it can influence the chances of successful embryo transfer. The analyzed studies indicated that low-grade mosaic embryos have a reproductive potential comparable to that of euploid embryos, and to increase the chances of gestational success, specific recommendations must be followed. For example, analyzing the affected chromosome pair is essential, which PGT-A facilitates more accurately since this condition can occur during the initial cell division after fertilization or during the subsequent development of the embryo. Nevertheless, the reviewed articles show that mosaic embryos have lower implantation rates and a higher risk of miscarriage. Following the guidelines of the European Society of Human Reproduction and Embryology (ESHRE), low-grade mosaic embryos should be evaluated for transfer, prioritizing morphological criteria. Some studies suggest that discarding embryos with low-grade mosaicism would result in a 36% reduction in the cumulative live birth rate. Additionally, studies have shown that in prenatal tests, only a small percentage of babies reflect the mosaicism detected at the embryonic stage, and an even smaller proportion develop any deformities due to these alterations.

**Conclusion:** The cohort of evaluated studies shows that the difference between babies born from the transfer of low-grade mosaic blastocysts or euploid embryos is not significant, demonstrating that these embryos can develop into healthy children. Even so, it is important to emphasize that decisions about using mosaic embryos should be made on a case by-case basis, considering various factors such as the percentage of mosaicism, the location of the affected chromosomes, patient preferences, whether it is the only available embryo, ovarian reserve, patient age, feasibility of a new ovarian stimulation, as well as emotion and financial aspects. It is imperative that patients receive all details about the risks and benefits these embryos may offer and information about success rates and other available options.

**P-54. Reflection of efficient quality control in a human reproduction laboratory on fertilization rates and blastocyst arrival**

**Cintia Pimentel Mangueira Teixeira^1^, Maria Luiza Silva Ricardo^1^, Giovanna Marcela Barbosa^1^, Wilson Jaccoud^1^, Anna Clara Rodrigues Petermann Bugalho^1^**

^1^Fert-Embryo Centro de Medicina Reprodutiva - Presidente Prudente - SP - Brazil

**Objective:** To associate the fertilization rate of oocytes subjected to Intracytoplasmic Sperm Injection (ICSI) and their arrival at the blastocyst stage with the effective quality control of an *in vitro* fertilization laboratory.

**Methods:** A retrospective cohort study involving 1747 metaphase II oocytes from patients with a mean age of 35.19 years, undergoing ICSI at a Human Reproduction Clinic in Western São Paulo. After injection, the oocytes were stored in the Ksysten incubator under appropriate temperature (37°C) and CO2 (8.3%) conditions. Between 16 to 18 hours later, fertilization was observed by the presence of two pronuclei in the oocyte cavity. On the fifth and sixth days of embryonic evaluation, the arrival at the blastocyst stage was identified and classified.

**Results:** The survey was conducted from January 2022 to December 2023, with results expressed as percentages. The average fertilization rate of oocytes subjected to ICSI was 72.27%, and subsequently, 68.55% of these reached the blastocyst stage.

**Conclusion:** Embryonic culture must occur with effective quality control. High fertilization rates and the development of embryos to the blastocyst stage demonstrate that the culture environment and the manipulation of gametes and embryos are occurring under ideal conditions. Such conditions, as in the present study, can be a good indicator of effective quality control and the ideal culture conditions the laboratory presents. Gametes are highly sensitive cells to physical and chemical stress, making them dependent on proper quality control during handling and culture. Some parameters, such as temperature, CO2, and pH, as well as the absence of contamination, are essential within the *in vitro* fertilization laboratory and directly influence patient outcomes, consequently increasing pregnancy rates.

**P-55. Impact of Hyaluronic Acid supplementation on bovine embryo cryopreservation via direct transfer method**

**Isabella Maran Pereira^1^, Maria Lúcia Alves Mundim Bernardes^2^, Márcia Carneiro de Sousa Silveira^2^, Cláudia Lima Verde Leal^3^**

^1F^ertilife - Departamento de Pesquisa & Desenvolvimento - Goiânia - GO - Brazil

^2^Fertilife - Chief Executive Office - Goiânia - GO - Brazil

^3^FZEA USP - Faculdade de Zootecnia e Engenharia de Alimentos da Universidade de São Paulo - Pirassununga - SP - Brazil

**The authors did not present the abstract.**

**P-56. Surplus embryos: Thinking about your destiny**

**Cássia Cançado Avelar^1^, Alexandre Oliveira^1^, Ariana Carvalho^1^, Inácio Diniz Júnior^1^, Ana Luísa Mendes Campos^1^, Luciana Campomizzi Calazans^1^, Erica Becker^1^, Ricardo Mello Marinho^1^, João Pedro Junqueira Caetano^1^**

^1^Clínica Huntington Pró-Criar - Belo Horizonte - MG - Brazil

**Objective:** When a person or couple arrives at an assisted reproduction clinic, a significant absence is present: the desired child. Assisted reproductive treatment provides a possibility - given the uncertainty of pregnancy, a highly valued resource is having surplus embryos. And when the arrival of child(ren) happens; when the desire for parenthood was fulfilled; when you can't or don't want to have children anymore and embryos are frozen? In this temporal displacement, embryos can be treated as “no longer wanted”, since they are associated with “dreams of the past” and not with a present project. It is therefore worth asking: what are the possible futures for the surplus embryos produced in assisted reproductive treatment? The decision about frozen embryos can be emotionally challenging, so psychological support can help patients explore their emotions and make an informed decision that is consistent with their personal values and circumstances. The objective is to evaluate patients’ decisions regarding the fate of frozen embryos, between disposal and donation.

**Methods:** 90 patients/couples underwent consultation with the cryobiology and psychology sector, from March to October 2023, in a private reproductive medicine clinic.

**Results:** The motivations and decisions regarding the outcome of frozen embryos were addressed in psychological consultations, and the outcomes were as follows: 66 patients/couples (73.3%) had already had children through treatment and opted for embryo disposal; 2 couples (2.2%) were unable to achieve pregnancy after treatment and opted for embryo disposal; 4 couples (4.5%) opted for embryo disposal after separation; 3 patients (3.3%) decided on embryo disposal after the death of their spouse; 2 couples (2.2%) initially chose embryo donation but could not proceed due to the patient's age at the time of treatment, so they decided on embryo disposal; 1 couple (1.1%) wanted to donate but could not due to an altered karyotype in one of the spouses, deciding on embryo disposal; 6 couples (6.7%) opted for embryo donation after having had children; 4 couples (4.4%) chose to keep the embryos frozen due to one spouse's uncertainty about the embryos' destination and 2 couples (2.2%) decided on embryo transfer.

**Conclusion:** Embryo donation presents unique challenges for donors. Assessing patients' decisions involves understanding these different aspects, respecting their individual choices and offering psychological support during the decision-making process. It is essential that patients feel empowered to choose what they consider to be the best option for them and the embryos in question.

**P-57. The Relationship between Advanced Maternal Age, Aneuploidies, and the Impact of Genetic Testing on *In Vitro* Fertilized Embryos**

**Julia Roverso Correa Silveira^1^, Isabella Paschoal Costa^1^, Sabrina Paz Gonçalves da Silva^1^, Beatriz Baptistella Cortez Teixeira da Rede^1^, Gabriel Monteiro Pinheiro^1^**

^1^Universidade Santo Amaro - São Paulo - SP - Brazil

**Objective:** It is widely understood that both the quality and quantity of oocytes in older women are substantially reduced. Consequently, assisted reproductive techniques have gained importance in the contemporary scenario as a strategy to overcome these reproductive limitations. Therefore, this study aimed to explore how advanced maternal age (AMA) influences the occurrence of aneuploidies, examining the increased risk in embryos due to egg aging. Additionally, the benefits of embryonic biopsy in older women were investigated, analyzing its effectiveness in identifying healthy embryos and reducing the risk of unsuccessful pregnancies or chromosomal abnormalities. Finally, the effectiveness of pre-implantation genetic testing (PGT-A) and non-invasive PGT-A (niPGT-A) in older women was evaluated, examining how these tests can help in selecting healthy embryos and decreasing the risk of unsuccessful pregnancies or the birth of children with chromosomal abnormalities.

**Methods:** A comprehensive integrative review was conducted, involving the thorough reading and analysis of 120 articles meeting methodological standards. This filtering process ensures the validity and reliability of the results, providing a solid foundation for the conclusions presented. Studies were retrieved from the following databases: Fertility and Sterility, Human Reproduction, PUBMED, and Cochrane, considering publications from 2015 to 2024, written in Portuguese or English. The descriptors used included niPGT-A, PGT-A, advanced maternal age, aneuploidies, and embryonic biopsy. The relationship between AMA and aneuploidies, the benefits or harms of embryonic biopsy, and the comparative effectiveness of different PGT-A methods were analyzed. Through Frequency Analysis and Opinion Evidence, the distribution and relevance of authors' opinions on these topics were sought.

**Results:** 90% of the studies indicate that AMA is associated with a high incidence of embryonic aneuploidies, often due to errors in chromosomal segregation, such as early separation of sister chromatids in eggs. Additionally, aneuploid cells tend to be negatively selected during embryonic development, through mechanisms such as apoptosis or differential proliferation, as well as the reduction of anti-Müllerian hormone, associated with decreased ovarian reserve. Embryonic biopsy showed favorable results, with a significant reduction in complications resulting from the transfer of aneuploid embryos, as indicated by 75% of the reviewed literature. Thus, it was found that next-generation sequencing (NGS) demonstrated high proficiency and effectiveness in detecting mosaic embryos. When performed at the blastocyst stage, embryonic biopsy increases the capture of euploid cells, increasing the chance of embryonic transfer. Furthermore, PGT-A reduces miscarriage rates and improves implantation rates in women with AMA who choose to transfer a single embryo. Regarding PGT-A methods, the effectiveness of niPGT-A (52.5%) surpasses that of PGT-A (35%), while the comparison of both (12.5%) offers a complementary approach. Although PGT-A is considered the gold standard, niPGT-A emerges as a promising alternative, analyzing both trophoblastic cells and the inner cell mass of the embryo, which may provide more comprehensive information about the ploidy state. These findings provide valuable guidance for patients facing challenges related to maternal age and the genetic health of embryos, highlighting the importance of advances in reproductive medicine.

**Conclusion:** The relationship between AMA and aneuploidies is evidenced and predominantly supported by the scientific community, with the application of embryonic biopsy widely recommended. Meanwhile, there is no consensus on which PGT method is most effective, although PGT-A remains the gold standard, niPGT-A was the most recommended method within the researched literature. Nevertheless, further studies in the literature are needed to better demonstrate the benefits of niPGTA so that it, in turn, can be widely used as a traditional method surpassing PGT-A, being reproduced on a large scale and becoming the new gold standard.

**P-58. Correlation of sperm DNA fragmentation with professional activity in the west region of Santa Catarina**

**Eduarda Rocha Bauer^1^, Gabrielli Darros Picinin^1^, Isadora de Oliveira Backes^1^, Simone de Oliveira Backes^1^**

^1^Geravita - Chapecó - SC - Brazil

**Objective:** The objective of this study is to analyze and correlate the most prevalent professions in the western region of Santa Catarina, establishing a comparison with the indices of sperm DNA fragmentation observed in patients from this area. The goal is to investigate occupational factors that influence sperm DNA integrity, gaining a better understanding of the relationship between professional and reproductive health.

**Methods:** This study included data from male patients aged between 21 and 53 years, who were attended at an assisted human reproduction clinic in Santa Catarina and underwent sperm DNA fragmentation tests between April 2023 and May 2024, with a fragmentation rate of more that 30%, according to the Halosperm^®^ kit criteria. The analyses compared the patients' professions with motility, concentration, and DNA fragmentation index, identifying the professions with the highest fragmentation indices.

**Results:** A total of 30 patients with a high fragmentation index were analyzed, revealing that 16 (53.3%) were agronomists, indicating a significant predominance of this profession. In addition, 8 (26.7%) of the patients were entrepreneurs, suggesting a considerable association between this occupation and high fragmentation rates. Another 3 patients (10%) were drivers, and 3 (10%) worked in several other professions. The data indicate that, although semen motility is adequate, sperm DNA fragmentation is a common problem among the three professions analyzed, agronomists had the highest fragmentation rate (44%), possibly due to occupational stress and exposure to pesticides. Studies show that exposure to pesticides and persistent organic pollutants increases sperm DNA fragmentation, affecting sperm production and integrity. Entrepreneurs had the lowest rate (33%), although still above the limit recommended by the WHO, while drivers had an intermediate rate (36%). Exposure to toxic substances and harsh working conditions, such as constant heat and vibrations and temperature of the testicles, are factors that contribute to sperm fragmentation. Sperm DNA fragmentation can increase the rate of miscarriages in men with fragmentation rates above 30%.

**Conclusion:** The analysis of the three groups of professions revealed that agronomists stood out, confirming the link between pesticides and infertility. Surprisingly, the drivers, even with few patients, showed high fragmentation, possibly due to prolonged time sitting, which warms the testicles. It was possible to clearly identify which professions were most related to fragmentation, highlighting the importance and impact of these professions in western Santa Catarina.

**P-59. Perception of attempting patients about direct contact with the embryologist during *in vitro* fertilization treatment**

**Andrea Mesquita Lima^1^, Sebastião Evangelista Torquato Filho^2^**

^1^Bios - Fortaleza - CE - Brazil

^2^Sollirium - Fortaleza - CE - Brazil

**Objective:** Direct contact between patients and embryologists in assisted human reproduction clinics can influence the treatment experience and perception of results. This study evaluates how prospective patients perceive and react to direct contact with the embryologist during their *in vitro* fertilization (IVF) treatment.

**Methods:** Qualitative research was carried out with 50 patients who underwent IVF treatments between June 2023 and December 2023. Data was collected through semi-structured interviews, which addressed communication, level of understanding, trust and general satisfaction of patients in relation to contact with the embryologist. Responses were analyzed using content analysis to identify recurring themes.

**Results:** Regarding clarity and understanding, 94% of patients reported that the embryologist's explanation of the procedures was clear and understandable. Patients valued the opportunity to ask technical questions directly to the specialist. 90% felt they had adequate access to information about embryo development and treatment stages. In terms of trust and safety, 90% of patients reported an increase in confidence in the treatment after direct conversations with the embryologist. 85% mentioned that direct contact reduced anxiety and increased the feeling of security. In the emotional aspect, 93% of patients felt that the embryologist also offered emotional support, in addition to technical information. Patients appreciated the empathy and understanding demonstrated during interactions. The patients considered the embryologist to be highly competent and crucial to the success of the treatment.

**Conclusion:** Direct contact with the embryologist was largely positive for prospective patients, improving communication, trust and satisfaction with the treatment. The presence of an accessible and communicative professional helped to reduce anxiety and increase the feeling of security. The close relationship between patient and embryologist also contributed to a more personalized and humanized treatment experience. This interaction model can be considered a best practice in assisted reproduction clinics to improve the patient experience and treatment results.

**P-60. Reproductive success of PCOS patients with *in vitro* oocyte maturation (IVM)**

**Camyla Duarte dos Santos^1^, Liris Naomi Noguchi^1^, Mariana Kasuga Morya^1^, Stephanie Alencar da Assunção^1^, Victoria Barchi Cordts^1^, Gabriel Monteiro Pinheiro^1^**

^1^Universidade Santo Amaro - São Paulo - SP - Brazil

**Objective:** The objective of the present study was to assess existing scientific evidence of the impacts to the success using the *in vitro* oocyte maturation for Polycystic Ovary Syndrome (PCOS) patients.

**Methods:** This bibliographic review was elaborated through a search in the PUBMED database from the descriptors “Polycystic Ovary Syndrome”; “fertility”, “IVM” and “*in vitro* oocyte maturation”, from the last 5 years, which included reviews, clinical trials and randomized controlled trials.

**Results:** Oocyte *in vitro* maturation (IVM) is a technique used to treat infertility mainly in patients with polycystic ovarian syndrome (PCOS), where a higher pregnancy rate was observed when compared to women without PCOS. Women with PCOS who need *in vitro* fertilization (IVF) have a greater risk of developing ovarian hyperstimulation syndrome (OHSS) if they use conventional ovarian stimulation, occurs due to the high antral follicle counts, which leads to failure in the selection of the dominant follicle, hence the technique of IVM is more indicated for these patients. Furthermore, when compared to superovulation by *in vitro* fertilization, it becomes more attractive due to reduced expenses, as well as less stimulation of gonadotropins, accelerated treatment time and permission cycle monitoring. However, it has a lower adherence rate due to its lower effectiveness in terms of cumulative live birth rate. After significant improvements in the area the pregnancy rate was greater than 40% using IVM in women with PCOS compared to conventional IVF. There are three distinct protocols employed in IVM. The conventional IVM protocol involves administering FSH for 3-6 days, in a lower dose to induce follicle growth. In the hCG-primed protocol besides the lower dose of FSH administered for 3-6 days, hCG is introduced 36 hours prior to the collection of cumulus-oocyte complexes (COCs). A trial revealed heightened clinical pregnancy rates associated with this approach. Moreover, women diagnosed with PCOS exhibited elevated pregnancy rates compared to those without PCOS when subjected to this protocol. The third one is the biphasic IVM protocol, also known as pre-IVM or CAPA IVM, incorporates the low FSH priming as well, differing mostly from the other two in the culture system. The adoption of the biphasic IVM method has significantly enhanced the efficiency of IVM, as evidenced by improved maturation rates, implantation rates, and live birth rates. For patients with a high antral follicle count (AFC) exceeding 24 and undergoing assisted reproductive technology (ART) procedures, IVM serves as an effective alternative to IVF due to its inherent advantages. A study demonstrated that CNP (Cyclic Nucleotide Phosphodiesterase)-mediated biphasic IVM did not induce alterations in methylation rates or gene expression at gDMRs (germline differentially methylated regions), nor did it affect the expression of major epigenetic regulators in human embryos.

**Conclusion:** Therefore, the treatment of infertility in patients with polycystic ovary syndrome has been simplified with the improvement of oocyte *in vitro* maturation (IVM), added to the biphasic IVM method, being a valuable and recent tool, due to its greater socioeconomic accessibility, reduction of side and adverse effects, lower intake of hormones and shorter time compared to traditional vitro fertilization (IVF). An important factor in choosing the method to be used for fertilization is taking care of the comfort and safety of the woman who has decided to go through this entire process, and for this reason, IVM has proven to be the most favored choice for patients with polycystic ovarian syndrome, at the moment.

**P-61. Reproductive success and the influence of GLP-1 receptor agonists on patients with chronic hyperandrogenic anovulation**

**Mariana Kasuga Morya^1^, Karolyne Vale de Sá^1^, Gustavo Nobrega Barbosa^1^, Stephanie Tasselli Alencar da Assunção^1^, Victoria Barchi Cordts^1^, Gabriel Monteiro Pinheiro^1^**

^1^Universidade Santo Amaro - São Paulo - SP - Brazil

**Objective:** The objective of the present study was to evaluate the existing scientific evidence between the influence of anti-obesity medications as the GLP-1 agonists on reproductive success.

**Methods:** This bibliographic review was elaborated through a search in the PUBMED database from the descriptors “semaglutide”, “GLP-1”, “ozempic”, “hyperandrogenic anovulation” and “fertility”, from the last 5 years, which included clinical trial, randomized controlled trial, review and systematic review.

**Results:** Evidence shows that beyond weight reduction and insulin resistance, GLP-1 agonists can directly influence the hypothalamus-pituitary-gonadal axis, aiding in hormonal regulation and reproductive function. The combination of liraglutide with metformin has been particularly effective in obese women with Polycystic Ovary Syndrome (PCOS), promoting metabolic and reproductive benefits. These findings suggest that GLP-1 receptor agonists may be a promising therapeutic option for women with PCOS, especially those also struggling with obesity and insulin resistance. The combined use of liraglutide and metformin resulted in significantly higher pregnancy rates compared to metformin monotherapy. In one study, it was demonstrated that after one year, 69.2% of women in the combined group achieved pregnancy, compared to 35.4% in the monotherapy group. Pregnancy rates per embryo transfer were 85.7% in the combined group, versus 28.6% in the monotherapy group. Additionally, it was observed that GLP-1 receptor agonists increased the secretion of Sex Hormone-Binding Globulin, reducing androgen bioavailability, which is beneficial for improving menstrual regularity and ovulation. Animal studies indicated that GLP-1 treatment during the proestrus phase in animal models doubled LH serum levels, increased progesterone in the luteal phase, and resulted in a greater number of mature follicles, thereby enhancing fertility. Short-acting GLP-1 receptor agonists also improved endometrial function, likely by reducing oxidative stress and fibrosis, aiding in implantation, and reducing gestational loss. Additionally, a study conducted with 28 infertile obese PCOS patients were divided into two groups: one group received metformin 1000 mg twice daily, while the other group received metformin combined with low-dose liraglutide (COMBI) 1.2 mg once daily, subcutaneously, for 12 weeks. After a 4-week medication-free period, the ovarian stimulation protocol was initiated. The pregnancy rate per embryo transfer was significantly higher in the COMBI group (85.7%) compared to the metformin group (28.6%) (*p*=0.03). Preconception intervention with low-dose liraglutide added to metformin is superior to metformin used alone, resulting in increased pregnancy rates per embryo transfer and cumulative pregnancy rates in obese and infertile PCOS patients.

**Conclusion:** Evidence indicates that GLP-1 agonists, beyond aiding in weight loss and insulin resistance, can directly impact the hypothalamus-pituitary-gonadal axis, enhancing hormonal regulation and reproductive function. Particularly, the combination of liraglutide with metformin has proven effective in obese women with PCOS, offering both metabolic and reproductive benefits. Studies show that this combination therapy results in significantly higher pregnancy rates compared to metformin alone. GLP-1 agonists also increase Sex Hormone-Binding Globulin levels, reduce androgen bioavailability, and improve menstrual regularity and ovulation. Additionally, short-acting GLP-1 agonists improve endometrial function, aiding in implantation and reducing gestational loss. In a study with obese infertile PCOS patients, the combination of low-dose liraglutide and metformin significantly outperformed metformin alone in terms of pregnancy rates per embryo transfer, highlighting the superiority of this combination therapy for preconception intervention in this population.

**P-62. In vitro-matured oocytes with cannabidiol impairs bovine embryo development**

**Caroline Schiavão Fernandes^1^, Bruno Carrino Suave^1^, Alan Brunholi Giroto^1^, Cintia Pimentel Mangueira Teixeira^1^, Amanda Talys Sampaio^1^, Rafael Martins Tonzar^1^, Enzo Dare Rissi^1^, Anthony Cesar de Souza Castilho^1^**

^1^Universidade do Oeste Paulista - Presidente Prudente - SP - Brazil

**Objective:** Bovine *in vitro* reproduction model can contribute to improve strategies for studies in human fertility. Cannabidiol (CBD) possesses therapeutic properties and antioxidant effects and preliminary study has demonstrated that bovine oocytes exposed to CBD carry out negative effects on cumulus cell expansion. Therefore, the aim of present study was to investigate the effect of CBD during oocyte *in vitro* maturation (IVM) on embryo development.

**Methods:** Bovine ovaries were collected from a local slaughterhouse and transported to the laboratory in a thermal container at 37°C in saline solution (0.9% NaCl). Antral follicles (3-8 mm in diameter) were aspirated using 18G needle. Cumulus-oocyte complexes (COCs) classified as grade I and II were selected and submitted to IVM using TCM 199 medium with bicarbonate, fetal bovine serum (FBS, 10%), pyruvate (2 µL/mL)), amikacin (75 µL/mL), follicle-stimulating hormone (FSH, 20 µL/mL), and luteinizing hormone (LH, 2 µL/mL). The CBD (Cannabidiol Prati-Donaduzzi, 20 mg/mL) was diluted in 0.05% of dimethyl sulfoxide (DMSO). The COCs were submitted to 0.1, 1 and 10 µM of CBD. The control group were performed using TCM 199 medium plus DMSO 0.05%. The COCs were matured at 38.5°C in a humid atmosphere for 24 hours. Four replicates were performed using 50 COCs/replicate. After, matured COCs were submitted to *in vitro* fertilization using TL Stock supplemented with BSA (6mg/mL), pyruvate (2µL/mL of a 100 mM solution), gentamicin (75µg/mL), heparin (3µg/mL), and PHE solution (penicillamine, hypotaurine, epinephrine - 44µL/mL. After 18 hours, cumulus cells were removed and presumptive zygotes were transferred to SOF medium (Synthetic Oviduct Fluid) supplemented with FBS (2.5%) and pyruvate (2µL/mL). On day 3, medium was partially changed, and cleavage rates were assessed. On day 7, blastocyst rate was assessed.

**Results:** The CBD 10µM group showed a significant decrease on cleavage rate (p=0.027) and a consequent reduction on blastocyst yield (p=0.002) compared to control group.

**Conclusion:** The addition of CBD 10 µM during IVM harm the future bovine *in vitro* embryo development probably due to impairment of cumulus cell expansion.

**P-63. The impact of COVID-19 on sperm parameters and male fertility: A literature review**

**Julia Marinho Simiao^1^, Rivia Mara Lamaita^2^, Victoria Goi de Morais Rodrigues^1^, Maria Paula da Gloria Diniz^1^**

^1^Faculdade Ciências Médicas de Minas Gerais - Belo Horizonte - MG - Brazil

^2^Mater Dei - Belo Horizonte - MG - Brazil

**Objective:** To perform a literature review to access the impact of COVID-19 infections on sperm quality during and after the infection to guide decisions about reproduction.

**Methods:** For this integrative literature review, searches were conducted using PubMed, SciELO and Virtual Health Library databases, using the keywords “ covid and sperm quality” and “COVID and male fertility”. The research criteria included selecting articles in both English and Portuguese that consisted of clinical trials, observational studies, retrospective and prospective cohort studies and 12 articles were included from 2019 to 2023. The articles that did not relate to the objective meta-analysis and literature reviews were excluded.

**Results:** The new coronavirus pandemic, ensued in February 2020 by the World Health Organization(WHO) resulted in a worldwide health crisis. The long term effects of the infection are still being investigated, however there have been links to male fertility impairments. It is known that infertility affects up to 15% of reproductive-age couples world-wide (Ring *et al*., 2016) and that male-related dysfunctions account for 40-50% of all cases of infertility (Farsimadan & Motamedifar, 2020). The male reproductive system is especially vulnerable to viral and bacterial infections, and recent studies show that SARS-CoV-2 directly affects the spermatogenesis process. The COVID virus uses the angiotensin-converting enzyme 2 (ACE2) receptor to enter human cells, especially male reproductive system cells such as spermatogonia and testicle Leydig and Sertoli cells because they have high ACE2 expression on their surface due to the fact that it helps regulate sperm motility and function. Therefore, it was speculated that COVID-19 infections could interfere with male fertility. That hypothesis was confirmed by the studies analyzed in this review which demonstrated impairment of testicular histology, orchitis and epididymitis due to inflammation factors of the virus infection, contributing to low semen quality and quantity. The parameters evaluated in the studies were: sperm mobility, concentration, volume and morphology and testosterone levels within the duration of the infection and following a period of time after recovery. The studies analyzed showed a consensus that sperm mobility was considerably reduced in patients with the disease, which improved after the recovery in the majority of cases. Sperm concentration was decreased according to the studies, however there were two different outcomes observed within the COVID-19 infected group, one with reduction considered infertile according to WHO parameters (lower that 15 million per mL) and another with patients that presented reduced concentration levels above WHO parameters for infertility. It was also shown that this parameter might normalize after recovery from the disease. Sperm volume was not significantly affected by the infection, being found normal or slightly decreased in most studies. Sperm morphology was the most severely affected parameter, Danders *et al*. (2022) study suggests that 67% of the recovered patients had teratozoospermia and that those parameters were not normalized 6 months after the infection. Other studies suggest that even though morphology is affected it remains within the normal WHO parameters. Testosterone levels were also evaluated, and all studies point to a decrease in the concentration of the hormone that also returned to normal levels after 3 months.

**Conclusion:** Further investigation is needed to assess the impact of COVID-19 infections on male fertility. However, the studies to date indicate damage to sperm count, motility and vitality which is mostly reversed within 3 months after recovery. Therefore, physicians should take these new studies into consideration when advising their patients on when to conceive after an COVID-19 infection and when to retrieve semen to perform assisted fertilization, since sperm quality is an important success factor in all reproduction.

**P-64. Incidence of cervical isthmus incompetence and chronic endometritis among patients with reproductive desire undergoing diagnostic videohysteroscopy**

**Giovanna Marcela Barbosa^1^, Tatiane Santana de Oliveira^1^, Mayara Mesquita Caravina^1^, Wilson Jaccoud^1^, Anna Clara Rodrigues Petermann Bugalho1, Cintia Pimentel Mangueira Teixeira^1^, Maria Luiza Silva Ricardo^1^**

^1^Fert-Embryo Centro de Medicina Reprodutiva - Presidente Prudente - SP - Brazil

**Objective:** The objective of this study was to assess the prevalence of cervical isthmus incompetence among patients and its correlation with chronic endometritis diagnoses.

**Methods:** This retrospective cohort study involved 111 patients undergoing fertility treatments, both low and high complexity, at a clinic in the Oeste Paulista region from February 22, 2023, to March 25, 2024. Participants underwent diagnostic videohysteroscopy procedures. Data collection included the Hegar 8.0 mm candle test, indicating cervical isthmus incompetence (>8.0mm), and biopsy reports of endometrial fragments with histopathological analysis to detect chronic endometritis (≥ 01 plasma cells/2 mm^2^).

**Results:** Prevalence rates of cervical isthmus incompetence and chronic endometritis were reported as percentages. Among the 111 patients, 35.14% showed cervical isthmus incompetence according to the Hegar candle test, while 74.77% exhibited chronic endometritis based on anatomopathological and immunohistochemical analyses. Of the cases with chronic endometritis, 37.35% were associated with cervical isthmus incompetence diagnosis. Furthermore, 28.27% of patients had isolated cervical isthmus incompetence.

**Conclusion:** Diagnostic videohysteroscopy proves indispensable for infertile patients. Factors such as chronic endometritis and cervical isthmus incompetence are linked to miscarriage rates. This study reveals a higher incidence of chronic endometritis, as determined by immunohistochemical analysis. With a combined rate of 37.35% for both factors, early detection becomes crucial to reduce future miscarriages and enhance clinical pregnancy rates.

**P-65. Corporate benefits for fertility preservation: An approach to family planning for female physicians in Brazil**

**Paula Myllena Pereira^1^, Camila Souza Oliveira^1^, Aristides Sertori Neto^1^**

^1^Universidade Nove de Julho - São Bernardo do Campo - SP - Brazil

**Objective:** To analyze corporate benefits for fertility preservation, focusing on the use of assisted reproductive technologies as an approach for family planning among female physicians in Brazil, considering the implications of these policies and the impact of delaying childbirth due to medical career demands.

**Methods:** This article is a literature review concerning corporate benefits for fertility preservation for female physicians. The data source is from journals indexed in PubMed using health science descriptors (DECS): Fertility Preservation, Employee Benefits, Physicians, Women, Reproductive Techniques, Assisted. Inclusion criteria were articles published between 2014 and 2024, excluding studies about cancer patients, male infertility, or unrelated topics. After applying the criteria, articles emphasizing data relevant to Brazil and international comparisons were analyzed.

**Results:** In recent decades, companies have expanded their policies on balancing professional and personal life as a strategy to attract and retain qualified professionals. In Brazil, this practice is still rare, with only 1% of companies with more than 500 employees offering this benefit. Female physicians face specific reproductive challenges when deciding to conceive, such as career progression impact, difficulties in reconciling work schedules with parental care, and the stigma associated with maternity in the medical profession. Consequently, they tend to have their first child 7.4 years later than the general population, often at an advanced maternal age (≥ 35 years at the time of childbirth). These professionals frequently delay motherhood due to the lengthy duration of medical training, residency, and the pursuit of career stability. A study with female oncologists revealed that one in three reported experiencing infertility and discrimination during pregnancy or maternity leave. The impacts of fertility preservation policies are mixed. Positively, access to assisted reproductive technologies can enhance women's reproductive autonomy and improve the quality of preserved eggs by reducing the average age of women opting for procedures like egg freezing. However, there are criticisms that these policies might suggest a greater sacrifice of personal and professional life, or imply that childless employees are more productive. Another point of analysis is that such benefits do not address all the challenges associated with balancing motherhood and career.

**Conclusion:** Corporate benefits for fertility preservation represent an important step in supporting female physicians in family planning. However, for these programs to be truly beneficial, they must be accompanied by adequate education about the limitations and risks of egg freezing, and should not replace other family support policies, such as on-site daycare and flexible schedules. Moreover, sociocultural changes are necessary to empower female physicians regarding their reproductive choices. Although the topic of corporate benefits for fertility preservation is increasingly relevant, there is still a lack of studies available in Brazil. Given the growing number of women in medicine, it is crucial that this issue is further studied and discussed. Promoting greater gender equity in medicine can be achieved by prioritizing the reproductive needs of female physicians, ensuring access to adequate resources to start and develop their families. Well-informed and structured corporate policies can contribute to a better balance between professional and personal life, as well as improve the organizational and financial performance of healthcare institutions.

**P-66. The influence of epigenetic factors on male fertility and reproductive health**

**Laura Caetano de Sá^1^, Mariana Lanuza Campos Pereira^1^, Thaís Lamounier^1^, Letícia Gomes^2^**

^1^Faculdade Ciências Médicas de Minas gerais - Belo Horizonte - MG - Brazil

^2^Hospital das Clínicas da Universidade Federal de Minas Gerais - HC UFMG - Belo Horizonte - MG - Brazil

**Objective:** The present study aims to investigate the impact of epigenetic changes on male fertility, correlating such alterations to semen production and elucidating their relationships with various environmental factors. By addressing the molecular mechanisms involved in the process of epigenetic changes, we intend to provide a detailed theoretical basis for future research.

**Methods:** Systematic literature review, in the indexed databases PubMed, SciELO and LILACS, guided by the descriptors “Epigenomics”, “Genetic Epigenesis” and “Male Infertility”. Studies from the last 10 years addressing semen quality, epigenetic changes and environmental factors were included. The data was statistically analyzed to identify significant patterns and correlations.

**Results:** Epigenetics represents mechanisms that determine gene expression, based on the activation and inactivation of such genes, which, despite being heritable, do not alter the individual's DNA sequence. It is a recent, but promising, field of study in reproductive medicine, since epigenetic changes influence sperm production and function. The most elucidated mechanism of this influence is the methylation of sperm DNA (addition of methyl -CH3 group on the 5' carbon), responsible for triggering gene silencing of around 70% of all methylated DNA. According to medical literature, dysregulations of the methylation process during spermatogenesis can lead to azoospermia and changes in germinative cells. Another mechanism is non-coding RNAs (ncRNAs), whose role in regulating gene expression occurs through post-transcriptional silencing or modulation of proteins. As ncRNAs can be transmitted between generations, they directly impact the characteristics of the progeny. The most influential scnRNAs for the gene regulation of male fertility are piRNAs, tsRNAs, miRNAs, rsRNAs and their changes directly impact male reproductive capacity. There is also the mechanism of post-translational modification of histones, which determine the conformation of chromatin, modulating the individual's gene expression. These mechanisms interact with each other, contributing to the formation of the epigenome and modeling sperm molecular load. In addition to molecular changes, environmental factors and lifestyle habits are also considered epigenetic mechanisms responsible for modulating sperm quality. Epidemiologically, the highest rates of male infertility occur in developed and significantly industrialized countries, demonstrating a link between infertility and environmental factors such as pollution, obesity, stress, smoking, alcohol, pesticides, nutritional deficiencies and, mainly, oxidative stress. Semen quality is 30 to 80% affected by the excessive production of reactive oxygen species (ROS), as they cause damage to the plasma membrane of the sperm cell, in addition to the loss of sperm quantity, motility and quality. Excess ROS occurs in diets with low antioxidants intake, such as zinc, B9, omega 3, vitamin C, vitamin E, coenzyme Q-10, selenium. Antioxidants are essential for semen, since besides acting as neutralizers of excess ROS, they are also cofactors of enzymes in DNA transcription reactions, act as lipid components of the sperm cell membrane, components of mitochondrial metabolism, and preventives of lipid peroxidation. Therefore, they directly impact testicular development, production, maturation, concentration, motility, morphology and structural integrity of sperm. Furthermore, other dietary changes related to low seminal quality are observed, such as high lipid and energy intake, consumption of processed red meat, sugary carbonated drinks, industrialized products rich in trans fat and linoleic acid.

**Conclusion:** Changes in gene expression processes and the presence of the environmental and behavioral factors discussed directly impact on male fertility, through damage to sperm development, quantity and quality. It is essential to understand the epigenetic mechanisms underlying male fertility, as it is a very recent and progressive field of study in Reproductive Medicine, applicable to the creation of new therapeutic protocols and strategies to prevent infertility.

**P-67. Real stories: Perspectives of gamete recipients**

**Flávia Giacon^1^, Helena Prado Lopes^2^**

^1^Mater Prime Clínica de Reprodução Humana - São Paulo - SP - Brazil

^2^Grupo Pró-Fértil Medicina Reprodutiva - Rio de Janeiro - RJ - Brazil

**Objective:** This study aims to understand the perspectives of young adults conceived by gamete donation in order to better support parents and their children. The decision to use an anonymous gamete donation in infertility treatment could have significant long-term psychological and social effects on everyone involved. Most people agree that being open about the donation is better for the child. Respecting the child’s autonomy means recognizing their moral right to know their genetic origins. Besides, being open about donor conception can prevent significant issues for family dynamics, due to the lack of transparency, and support a child’s psychological well-being. Understanding how these young adults feel about their conception, whether they wish to seek information about their donor and if they want to meet them is, therefore, of paramount importance for this discussion. Through this study, we aim to encourage future research in Brazil. Justification: In the past, it was considered acceptable to hide the truth about a child’s biological origin. Nowadays, most people believe that this information should be shared with the child. Parenthood often involves technological intervention and individual choice, thus raising several questions. Is it important for a child to know their genetic background? Or is it more important for a child to bond with their prospective parents? In this context, there are a lot of important questions about new possibilities in human reproduction that focus on the child’s best interest, and this study seeks to find those answers.

**Methods:** This study examines the literature on the long-term outcomes for children in families formed through gamete donation, with a focus on child psychological adjustment. The following sources were used during the research: “I was quite amazed: donor conception and parent-child relationships from the child’s perspective”, by Lucy Blake, Polly Casey, Vasanti Jadva, Susan Golombok (2023); “Let’s talk about egg donation: real stories from real people - The child’s best interest in gamete donation”, by Marna Gatlin, Carole Lieber Wilkins and John Hesla (2019); “Lethal secrets: the psychology of donor insemination - problems and solutions”, by Annette Baran and Reuben Pannor (2008).

**Results:** By focusing on the child’s needs, it becomes clear that the care perspective considers children’s rights. Keeping the secret from the beginning can conflict with the autonomy of the uninformed person, potentially magnifying the significance of the secret. For the child’s best interest, it is better to empathize with parents who are hesitant to inform their child about the donation rather than just reminding them of the child’s right to know.

**Conclusion:** Problems can arise in families who try to keep the information secret as well as in those with open dialogue about donor conception. Effective counseling is important to help prospective parents to understand the implications of their choices. The evolving notion of the child’s best interest now emphasizes autonomy and citizenship, framing the child’s interests according to their legal status. The conclusion is that children conceived from donated gametes should not be denied knowledge of their origins. Through this study, we encourage clinics to promote research on this topic and to understand the experiences of Brazilian families using assisted reproductive technologies.

**P-68. A legitimization of the female single parenthood**

**Flávia Giacon^1^, Helena Prado Lopes^2^**

^1^Mater Prime Clínica de Reprodução Humana - São Paulo - SP - Brazil

^2^Grupo Pró-Fértil Medicina Reprodutiva - Rio de Janeiro - RJ - Brazil

**Objective:** The objective of this study is to expand knowledge about the legitimization of this family structure and to explore the conflicts and challenges faced by women who choose solo motherhood, both on an individual level and within familial and social contexts. Reproductive technologies now allow women to achieve motherhood through pregnancy without sexual intercourse. For single women, this option creates a family identity that differs from single parenthood resulting from widowhood or divorce. Given the parental responsibilities, social conflicts, and stigmas that solo mothers face - not long ago considered “incomplete” families - there is a significant effort by these women to legitimize their choice of single parenthood.

**Methods:** This study includes a literature review conducted using online databases such as PubMed, SciELO, and Redalyc, focusing on recent literature about female single parenthood resulting from assisted reproductive treatments and its psychosocial aspects. Additionally, it considers anonymous reports from professionals in this field, as documented in scientific articles. Reproductive technology has made it possible for women to conceive without a partner, allowing them to fulfill their desire for motherhood independently. Since 1981, changes in values, social norms, and legislative adjustments concerning filiation, marriage, and the legalization of divorce have enabled new family structures. These changes have allowed women to enter the workforce, gain voting rights, and use contraceptives, among other advancements. This new socio-historical and cultural context has empowered women to make their own professional and personal choices, including choosing motherhood without a paternal figure.

**Results:** Given the increasing relevance of single parenthood in social science studies over the past decades, this study aims to highlight the importance of legitimizing this family structure and to address the conflicts faced by women who choose solo motherhood and their strategies for overcoming them. The literature review identified several key areas: the moment the decision is made, reactions from family and social contexts, the reproductive treatment process, pregnancy, labor, and the child’s arrival. This study provides relevant conceptual insights for planning research on female single-parent families, particularly regarding investigative and intervention strategies by mental health professionals.

**Conclusion:** Understanding female single-parent families resulting from reproductive medicine requires multiple studies rather than a single one. The importance and necessity of interdisciplinary research are emphasized to better address the specific and social demands of this unique family structure. It is essential to understand how these women navigate norms, social values, and culturally standardized processes as they legitimize their desire for solo motherhood, often challenging old, established standards.

**P-69. Influence of follow-up duration on patency and pregnancy rates after vasectomy reversal**

**Marina Albuquerque Cavalcanti Almeida^1^, Alexandre Barbosa Albuquerque^1^, Henrique Ferreira Wagner^2^, Débora Antas Campello Souza^1^, Matheus Souza Nogueira^1^, Filipe Tenorio Lira Neto^3^**

^1^Fert-Embryo Centro de Medicina Reprodutiva - Presidente Prudente - SP - Brazil

**Objective:** Vasectomy reversal (VR) is a crucial option for couples seeking fertility restoration after vasectomy. Although literature reports patency rates up to 96% and pregnancy rates up to 92% with extended follow-ups, loss to follow-up is common and likely underreported. This study aims to determine if follow-up duration impacts patency and pregnancy rates after VR.

**Methods:** We reviewed a prospectively maintained database of 211 consecutive men who underwent VR by a single surgeon from June 2016 to May 2023. Participants were followed through office visits and phone calls every 6 months until pregnancy was achieved. We included participants with at least one post-operative semen analysis, categorized them based on follow-up duration, and analyzed its impact on patency and pregnancy rates. Patency was defined as the presence of any sperm in the ejaculate. Pregnancy was defined as the presence of visualization of the gestational sac or heartbeat by ultrasound using transvaginal transducer.

**Results:** Out of 211 men who underwent VR, 33% did not return for post-operative semen analysis, leaving 142 (67%) participants for inclusion in the analysis. The mean participant and partner ages were 40 (±7) and 31 (±5) years, respectively, and the median time since vasectomy was 8 years (IQR 5, 12). Bilateral vasovasostomy was performed in 58% of participants, bilateral vasoepididymostomy in 15%, unilateral vasovasostomy in 6%, unilateral vasoepididymostomy in 4%, and a combination of unilateral vasovasostomy and unilateral vasoepididymostomy in 16%. The median follow-up duration for the entire cohort was 16 months (IQR 6, 28). Sixty participants (42%) had up to 12 months of follow-up, and 99 participants (70%) had up to 24 months. Patency and pregnancy rates were 91% and 30%, respectively, within the first 12 months of follow-up; 94% and 37% within the first 24 months; and 94% and 49% for the entire cohort (p=0.70 and *p*<0.05, respectively). Sixty-one percent of pregnancies occurred within the first 12 months post-procedure, while 23% occurred after 24 months (*p*<0.05).

**Conclusion:** Loss to follow-up after VR is common and may bias reported outcomes. Although patency rates remain unaffected, shorter follow-up durations (less than 12 months) are associated with decreased pregnancy rates, as up to 39% of pregnancies occur beyond this period. Therefore, studies evaluating VR outcomes should consider longer follow-up durations to provide more accurate assessments.

**P-70. Male infertility and the importance of multidisciplinary care**

***Valéria De Macêdo Teixeira Batista^1^, Helena Prado Lopes^2^***

^1^Clínica Perfetto Reprodução Humana - Goiânia - GO - Brazil

^2^Grupo Pró-Fértil Medicina Reprodutiva - Rio de Janeiro - RJ - Brazil

**Objective:** This study aims to explore the male experience when they do not fulfill the socially prescribed role of a reproducer, particularly upon discovering infertility. It also emphasizes the importance of the psychological and social aspects of male infertility and discusses the implications for clinical care and future research. Traditionally, the psychosocial aspects of infertility treatment often focus more on women than men. Men are typically viewed as fertile, which aligns with traditional notions of masculinity, but can lead to stigmatization. The stress associated with male infertility has been linked to psychological issues such as depression, anger, feelings of inadequacy, and embarrassment. These concerns can affect both individuals and their relationships. Besides, there is a stigma associated with male infertility, which often causes infertile men to feel inadequate as partners and question their contribution to the relationship.

**Methods:** To explore male infertility and the importance of multidisciplinary care, this study carried out a review of existing literature. The review focused on studies that address key dimensions of male infertility, such as:

The complex relationship between infertility and psychological distress (Review). Gabriela Simionescui, Bogdam Doroftell, Rau Maftel, BiancaElena Obreja, Emil Antoni, Delia Grabi, Ciprian Ilea, and Carmen Antoni (2020).

Male infertility and psychological repercussions: a neglected problem in Northern Upper Egypt. Wafaa Mostafa Ahmed Gamel, Hanan Elzeblawy Hassan, and Alyaa Abdallah El-Ezazy (2019).

(Male) infertility: what does it mean to men? New evidence from quantitative and qualitative studies. Tewes Wischmann, and Petra Thorn (2013).

Infertility can cause men to question their masculinity, leading to feelings of inferiority not only in the bedroom but also in social and professional settings. In emotional relationships, men are less likely to share their feelings or seek professional help for personal issues. Notably, most literature on infertility has been developed within the field of medicine, primarily focusing on methods, diagnoses, and treatment options.

**Results:** Although few men seek mental health professionals, psychosocial services should be available to them. The primary predictor of the perceived importance of these services for men is the high stress related to infertility in personal, marital, and social aspects. Men can be affected by infertility in several ways: receiving a diagnosis of infertility, being the partner of an infertile woman, being part of a couple with unexplained infertility, or pursuing solo parenthood. In this context, the inability can lead to feelings of inadequacy, and men often feel compelled to set aside their emotional needs.

**Conclusion:** Several questions have emerged from this study: How do infertile men perceive themselves? What impact does the infertility diagnosis have on them? How do they position themselves in a world where prevailing traditions link sexuality, virility, fertility, and fatherhood? Does being infertile affect their relationships? How do they perceive the idea of parenthood? The research concludes that most studies focus on women; however, a small group has specifically concentrated on the experiences and needs of men, highlighting that singular male experiences are not adequately addressed as a focus of attention and care. Reproductive medicine clinics should have a specialized and trained multidisciplinary team to support infertile men, partners of infertile women, and men pursuing solo parenthood. It is also essential to include mental health professionals in these teams to ensure that care for men is as humanized and individualized as it is for women.

**P-71. Evaluation of CDKN1A and CDKN2A genes as susceptibility factors for recurrent pregnancy loss: a possible involvement of cellular senescence**

**Luiza Pretto^1^, Eduarda Nabinger^1^, Eduardo Cremonesi Filippi-Chiela^1^, Thayne Woycinck Kowalski^2^, Maria Teresa Vieira Sanseverino^2^, Lucas Rosa Fraga^1^**

^1^Universidade Federal do Rio Grande do Sul (UFRGS) - Porto Alegre - RS - Brazil

^2^Hospital de Clínicas de Porto Alegre (HCPA) - Porto Alegre - RS - Brazil

**Objective:** Recurrent pregnancy loss (RPL) is characterized by the occurrence of two or more pregnancy loss events. Despite its etiology encompasses several known causes and risk factors, the statistics are unfavorable and more than a half of the cases remain unsolved. In this sense, many efforts are being made by the scientific community to explain idiopathic cases, such as the genetic factors. Cellular senescence (CS) is the state in which cells are no longer able to divide even after being stimulated with growth factors. These cells, despite arresting in the cell cycle, remain metabolically active and influence their microenvironment. CDKN1A and CDKN2A are key genes to CS, being involved respectively in the arrest and maintenance of cell cycle arrest. CS is present in several physiological contexts, including reproduction and embryonic development, but its impact on pregnancy is still unclear. In this scenario, the aim of this study was to evaluate the involvement of CDKN1A and CDKN2A genes in the RPL context.

**Methods:** For this, a differential gene expression (DGE) evaluation in placenta and endometrium was performed using publicly available data from Gene Omnibus Expression (GEO). In addition, genetic variants in CDKN1A (rs2395655) and CDKN2A (rs11515) were assessed in a local sample of 116 women with RPL and 120 fertile controls using TaqMan Genotyping Assays.

**Results:** In DGE analyses of the public data, we found no statistically significant difference for both genes when comparing RPL women with controls. No statistically significant differences were found in allelic or genotypic frequencies of rs2395655 and rs11515 variants between the RPL and the control groups in the genetic analysis as well. However, when a multiple comparison was performed, two genotype combinations were found to be statistically differently distributed between the groups: rs2395655 AG and rs11515 GG more frequent in RPL group; and rs2395655 AG and rs11515 CG more frequent in control group, which might indicate these combinations as susceptibility or protective factors, respectively, for RPL. In conclusion, we believe that in terms of DGE, patients can return to the protein profile prior to the pregnancy loss event or assume a new profile, disfavoring the analysis, once sample collection would need to be exactly at the time of the pregnancy loss to reflect the reality, which was not the case of the samples available and analyzed.

**Conclusion:** Although our data is preliminary, with our genetic analysis results we believe that CS may permeate a fraction of RPL cases. As a perspective, our research team will continue the genetic study by analyzing more genes related to CS using a genomic approach.

**P-72. The symbolic representation of gametes may hinder its acceptance**

**Rose Marie Massaro Melamed^1^, Helena Prado Lopes^2^, Valéria De Macedo Teixeira Batista^3^**

^1^Fertility Medical Group - São Paulo - SP - Brazil

^2^Grupo Pró-Fértil Medicina Reprodutiva - Rio de Janeiro - RJ - Brazil

^3^Clínica Perfetto Reprodução Humana - Goiânia - GO - Brazil

**Objective:** The aim of this study is to identify the emotional aspects that hinder the initial acceptance of gamete donation. The plan to have a child, much like the instinct to preserve and continue the species, may be linked to the idea of family continuity and a sense of hope for the future. Pregnancy is a very complex period in a woman’s life, marked by significant biological and psychological changes. While egg donation greatly increases the chances for infertile couples to fulfill their desire to have a child, it also raises specific issues that differ from those faced in assisted reproduction using the couple’s own gametes. Using donor eggs can create a narrative that is not easily understood. It is a fundamental alterity where one cannot detach from the reality of seeing the child, who is perceived through the lens of the missing part of oneself in the child (or descendant). During pregnancy, as the fetus grows in the mother’s womb, the image of the baby also develops in her psyche. This context presents many challenges for pregnant women, as the need for egg donation presupposes a prior situation of infertility, with unique circumstances becoming an integral part of the maternal experience. The lack of knowledge about part of the genetics may lead to concerns and fears during pregnancy, which can also be a period for elaborating on the experiences of infertility and the unique aspects of egg donation, facilitated or hindered by each woman’s personal history. Being genetically related or not can be a complex issue. Why do some women struggle to accept donated eggs based on medical recommendations due to their inability to conceive with their own gametes? Are there behavioral and/or psychological differences in accepting donated gametes?

**Methods:** Psychological consultations were conducted with women recommended to undergo egg donation, who were divided into three groups: patients who still had their own eggs but with poor quality due to advanced age and had previous unsuccessful IVF attempts; patients who, after unsuccessful IVF attempts using their own eggs, received gametes from relatives up to the fourth degree; patients who already knew they would not be able to conceive with their own eggs.

**Results:** Even before pregnancy, the first bonds between parents and their baby start to form through the desire to have a child (Bayle, 2005). In this context, some authors talk about the existence of three baby representations in parents’ minds: a fantasized baby, an imaginary baby, and a real baby. In the case of gamete donation, both the fantasized and imaginary babies fade away, revealing only the real baby at birth. When patients understand that their only option to fulfill their desire for a child is through a reception of gametes, the processes of acceptance and symbolization become less difficult. The frustration of unsuccessful attempts with their own eggs and the shock of an infertility diagnosis cause significant emotional suffering, making it harder to symbolically accept the gamete.

**Conclusion:** It is concluded that sadness, guilt, frustration, powerlessness, insecurity, fear, distress, helplessness, and anxiety are commonly experienced during the process of gamete reception. Beyond the transmission of two genetic lineages, there is also the maternal contribution. This highlights that, even on a biological level, we are much more than our genes. Additionally, other factors such as psychological, symbolic, and social identifications and transmissions will play a key role in shaping who we are.

**Reference**

Bayle F. A parentalidade. In I. Leal I, ed. Psicologia da Gravidez e da parentalidade (pp. 317-343). Lisboa: Fim de Século; 2005. p. 317-43.

**P-73. Could microfluidic sperm selection enhances laboratory outcomes in oligozoospermic patients?**

**Isabella Mendes Alves Maraz^1,^ Michelli Suemi Tanada^1^, Karla Pacheco Melo^1^, Ivan Henrique Yoshida^1^, Emerson Barchi Cordts^1^, Caio Parente Barbosa^1^**

^1^Instituto Ideia Fértil - São Paulo - SP - Brazil

**Objective:** The aim of this study is to compare microfluidics sperm selection technique with density gradient centrifugation in order to evaluate any laboratory rates (fertilization, blastulation and euploidy).

**Methods:** Retrospective study including 332 couples, with oligozoospermic partners, submitted to assisted reproduction treatments from January 2021 to December 2023. From these couples, 152 used microfluidics sperm selection (MSS) techniques and 180 used density gradient centrifugation (DGC). Besides, 63,8% went through Preimplantation Genetic Testing (PGT). All oocytes were obtained from fresh cycles and all embryos were cultivated to blastocyst stage, with or without genetic tests. Fertilization, blastulation and euploidy rates were analyzed among groups and separated by maternal age (Group 1: until 37 years old; Group 2: 38-40 years old; Group 3: >40 years old) and seminal processing technique (MSS or DGC). Qualitative variables were presented by relative and absolute frequencies, and quantitative by median with confidence interval value of 95%. To compare the variables between techniques, Mann-Whitney and Chi-square tests were performed. To all the analysis the significance level was p<0.05. The statistical testing program used was MiniTab Version 16.0.

**Results:** No statistically significant results were found in any of the laboratory parameters evaluated when comparing techniques (MSS and DGC) and maternal age (Groups 1, 2 and 3). From all injected oocytes, Group 1 (n=152) showed 75.78% MSS *vs*. 75.17% DGC fertilization rates (*p*=0.987), while Group 2 (n=104) showed 70.52% MSS vs. 73.6% DGC (*p*=0.720) and Group 3 (n=76): 66.07% MSS *vs*. 75.56% GDC (*p*=0,362). After 5 to 7 days of embryonic development, blastulation rates were 71.57% MSS *vs*. 82.90% GDC (*p*=0.333), 79.60% MSS vs. 78.30% GDC (p=0.909) and 68.91% MSS *v*s. 73.65% GDC (p=0.704). The euploidy rates after PGT were: Group 1 (n=77): 41.05% MSS *vs*. 47.59% GDC (*p*=0.462), Group 2 (n=78): 33.04% MSS *vs*. 23.74% GDC (*p*=0.218), and Group 3 (n=56): 13.63% MSS *vs*. 9.63% GDC (*p*=0.497). When analyzing laboratory rates, Group 2 showed a tendency towards better euploidy rates, despite no statistical difference from MSS (33.04%/23.74% *p*=0.218). Indicating that this technique might be beneficial to advanced maternal age patients (Group 2), who represent a large proportion of patients seeking assisted reproduction treatments.

**Conclusion:** An adequate seminal processing is essential in order to offer better laboratory rates and cycle outcomes to couples with oligozoospermic partners. MSS technique is shown to be tendentiously superior on euploidy rates when compared to DGC in patients over 37 years old, although this study showed no statistically significant results. The authors understand that further complementary studies with bigger casuistry are appropriate to solidify statistical relevance.

**P-74. Exposure to a mixture of endocrine disruptors based on human exposure during gestation and lactation influences prostatic tissue characterization**

**Maria Luiza Silva Ricardo^1^, Vitor de Oliveira Pinaffi^1^, Thaina Cavalleri Sousa^1^, Letícia Pereira de Souza^1^, Andreia Yuri Yoshigae^1^, Karianne Delalibera Hinokuma^1^, Ana Beatriz Ratto Gorzoni^1^, Ariana Musa de Aquino^2^, Wellerson Rodrigo Scarano^2^, Gisele Alborghetti Nai^1^, Leonardo de Oliveira Mendes^1^, Cintia Pimentel Mangueira Teixeira^3^, Wilson Jaccoud^3^**

^1^Universidade do Oeste Paulista (UNOESTE) - Presidente Prudente - SP - Brazil

^2^Instituto de Biociências (IBB/UNESP) - Botucatu - SP - Brazil

^3^Fert-Embryo Centro de Medicina Reprodutiva - Presidente Prudente - SP - Brazil

**Objective:** To evaluate the histopathological aspects related to prostatic homeostasis in elderly animals exposed to a mixture of twelve endocrine disruptors (EDs) during gestation and lactation.

**Methods:** Pregnant females of Sprague-Dawley lineage were randomly distributed into 2 experimental groups: Ctrl Group (vehicle: corn oil) and ED Mix Group: 32.11mg/kg/day of the mixture consisting of twelve compounds (phthalates, pesticides, UV filters, bisphenol A, butylparaben) diluted in corn oil (2ml/kg), administered by gavage. Pregnant or lactating rats received treatment from gestational day 7 (GD7) until postnatal day 21 (PDN21). After weaning, male pups were maintained with ad libitum water and food until reaching 440 days of age when they were euthanized. After euthanasia, the ventral prostate of the animals was collected, dissected, and subjected to histopathological analyses. Fractal, morphometric, and stereological analyses were performed on slides stained with hematoxylin and eosin; collagen quantification was conducted on slides stained with picrosirius-hematoxylin; mast cell quantification was also performed on slides stained with toluidine blue.

**Results:** Regarding biometric data, no alterations were observed in body weight gain, relative prostate and epididymis weight. Testes showed higher weight in the group of animals exposed to the mixture. Stereological analysis demonstrated an increase in the epithelial and stromal compartments, followed by a decrease in the luminal compartment in the ventral prostate of animals in the ED Mix group. Morphometric analysis also revealed an increase in epithelial height in the group that received the ED mixture. The alterations observed in stereomorphometric and karyometric analyses were not evidenced by fractal analysis, which showed no difference in the pattern of cell organization in the prostatic microenvironment. The ED mixture was also responsible for the emergence of a more rounded nuclear phenotype in animals in the ED Mix group, as evidenced by karyometric analysis. The treated group also showed an increase in nuclear area and perimeter. Greater deposition of collagen fibers was observed in the prostatic stroma of animals subjected to exposure to EDs, with this increase being positive for both type I and type III collagen. Regarding mast cell quantification, the group exposed to the mixture showed recruitment of this cell type in the prostatic stroma, mainly in the population of intact mast cells, suggesting the existence of an intense inflammatory process in these animals.

**Conclusion:** The results obtained in this study are supported by experimental data confirming that gestational and early postnatal exposure to EDs alters the pattern of prostatic development, which, when combined with other environmental and genetic factors, may lead to more severe consequences in aging, such as the emergence of chronic diseases like cancer.

**P-75. Oocyte donation between family members: A reported case series**

**Adriana Cristine Arent^1^, Marta Ribeiro Hentschke^1^, Talita Colombo^1^, Fabiana Mariani Wingert^1^, Victória Campos Dornelles^1^, Isadora Badalotti-Teloken^1^, Vanessa Devens Trindade^1^, Alvaro Petracco^1^, Mariangela Badalotti^1^**

^1^Fertilitat Centro de Medicina Reprodutiva - Porto Alegre - RS - Brazil

**Objective:** To describe a series of cases of familiar oocyte donation.

**Methods:** Series of cases performed in a reproductive medicine center from 2021 to 2024, including patients who underwent *in vitro* fertilization (IVF) with oocyte donation from family members. All patients underwent extensive medical and psychological evaluation throughout the treatment, and the donation was altruistic in nature. The study was approved by the ethics committee.

**Results:** Couples’ motivations for using related egg donors included preserving the family's genetic inheritance, reducing the costs and/or the waiting time for treatment. The recipients were poor responders or had primary ovarian insufficiency or successive IVF failures. The age of recipient and donors vary from 32 to 45 years old and 19 to 36 years old, respectively. Regarding the donor's kinship, 3 were nieces, 4 were sisters and one was daughter. From the 8 donors, 6 reported having a steady relationship and 4 already had children (50%). Eight patients performed an IVF cycle with fresh donor oocytes and autologous semen; one also performed two IVF cycles with frozen oocytes from the first cycle. In total there were 10 transfers from 6 patients. These patients underwent fresh embryo transfer; later, 4 underwent frozen embryo transfer. From the patients that did not undergo embryo transfer, one patient performed a preimplantation genetic test (donor, 32-year-old sister) with aneuploidy results and one patient has not performed embryo transfer till this day for personal reasons. Out of the six patients that underwent embryo transfer, 3 had positive beta-HCG, all of which progressed to live birth (twin at 32 weeks; premature at 35±6 weeks, and one full-term at 38 weeks). Cumulative pregnancy rate per patient was 3/6 (50%).

**Conclusion:** The increase in potential donors facilitated the assisted reproduction process for many couples and made it possible to perpetuate the family's genetics and heredity. For many families, this possibility based on family ties and affection is very welcome. However, concerns have been raised over the use of family members as donors and these have mainly centered on the degree of autonomy that donors have when faced with a family member in need of gametes. Studies that evaluate the importance of psychological monitoring throughout pregnancy and after birth will be of great value.

**P-76. Transfer of frozen thawed embryos with and without PGT-A analyses**

**Mariana Saikoski Faller^1^, Lisiane Knob de Souza^1^, Betina Iser^1^, Sabrina Solka Bonness^1^, Rafaela Amaro Link^1^, Andreia Moro Tore^1^, Carolina Giordani Andreoli^1^, Fabiane Sartori de Avila Trolli^1^, Ricardo Francalacci Savaris^2^, Noeli Cecília Sartori^1^, Carlos Alberto Link^1^**

^1^Clínica Proser - Porto Alegre - RS - Brazil

^2^UFRGS - Porto Alegre - RS - Brazil

**Objective:** To compare the clinical pregnancy rate between patients who underwent frozen-thawed embryo transfer with and without PGT-A.

**Methods:** This is a retrospective cohort study. Patients submitted to frozen-thawed own embryo transfer from procedures performed between 2022 and 2024, in an assisted reproduction center in south of Brazil. Patients were divided into groups that underwent PGT-A testing and those that did not. In a secondary analysis, patients were grouped by age range: 35 years or younger, 36 to 40 years, and over 40 years. Statistical analysis was performed using the Student's t-test and chi-square test, considering *p*<0.05 as significant.

**Results:** A total of 320 patients were included, 72 patients in the PGT-A group and 248 patients in the non-PGT-A group, with an average age of 39.30±2.55 and 35.69±4.07 years (*p*<0.001), respectively. The PGT-A group had a clinical pregnancy rate of 74% while the non-PGT-A group had a rate of 44% (*p*<0.001). Considering the significant age difference, patients were stratified by age. The groups of 35 years or less and 36 to 40 years in the PGT-A group had higher clinical pregnancy rates compared to patients in the same age groups in the non-PGT-A (n=5 *v*s. 96, 100% *vs*. 55%, *p*=0.048; n=46 *vs*. 137, 83% *vs*. 46%; *p*<0.001), respectively. The group over 40 years did not show a significant difference between the PGT-A and non-PGT-A groups (n=21 *vs*. 15, 52% *vs*. 33%; *p*=0.256).

**Conclusion:** In this study, patients who underwent PGT-A testing and obtained euploid embryos showed higher clinical pregnancy rates compared to patients who did not undergo the same test. The PGT-A test is indicated for patients above 35 years old and our results for this same age group showed a significantly higher clinical pregnancy rate. However, in the age group over 40 years, there was not a significant difference that may be related to the very small sample size.

**P-77. Exploring the interplay between endometriosis and altered expression of endometrial markers: Implications for relationships**

**Silvia Morales Jau^1^, Marina Silva Rodrigues^2^, Marcelo Lucchesi Montenegro^1^, Camila Silveira Souza^1^, Luana Teixeira Souza Rodrigues^3^, Bernardo Rodrigues Lamourier Moura^3^, Edson Guimarães Lo Turco^3^, Fernando Prado Ferreira^1^**

^1^Neo Vita Clinic - São Paulo - SP - Brazil

^2^Gynecology Department, São Paulo Federal University - São Paulo - SP - Brazil

^3^Urology Department, Human Reproduction Section - São Paulo - SP - Brazil

**Objective:** To evaluate the relationship between traditional markers for endometritis and uterine receptivity with endometrium from different profile patients.

**Methods:** In a retrospective cohort study, we evaluated 187 women submitted to endometrial biopsy divided into: endometriosis group (n=24), repeat miscarriage group (n=24), adenomyosis group (n=9), implantation failure group (n=19), functional uterine factor group (n=86), maternal age group (n=11), bad responders’ group (n=2) and a control group (n=12). We measured the E2 and P4 receptors, as well as the baseline levels of E2 and P4. The immunoassay test was used to detect endometrial markers and electrochemiluminescence to determine serum E2 and P4 concentrations. We calculated the ratios between these variables using the Bonferroni test.

**Results:** When comparing E2 receptors, we observed that the endometrium of women with implantation failure and uterine dysfunction had higher presence of stromal E2 receptor cells than control (92.06±12.75 x 74.55±23.92, *p*=0.060 and 89.94±15.01 x 74.55±23.92, *p*=0.034). A significant statistical difference was also noticed when comparing the glandular E2 receptor cells from the maternal age and uterine dysfunction groups (80.63±9.42 x 89.94±15.01, *p*=0.066). There was no difference in the baseline levels of E2 and P4 between the groups.

**Conclusion:** In conclusion, the study highlights the importance of evaluating endometrial E2 receptor expression, particularly in stromal cells, as a potential marker for implantation failure and uterine dysfunction. These findings suggest that further research is warranted to explore the role of endometrial hormone receptor expression in uterine receptivity and its implications for clinical practice.

**P-78. A study about the effectiveness of an alternative ovarian stimulation protocol with long-acting corifollitropin alfa (Elonva^®^), clomiphene, and letrozole**

**Denis Schapira Wajman^1^, Georges Fassolas^1^, Carlos Roberto Izzo^1^, Luiz Fernando Henrique^1^, João Antônio Dias Jr^1^**

^1^Originare Medicina Reprodutiva - São Paulo - SP - Brazil

**Objective:** To determine if an alternative method of controlled ovarian hyperstimulation (COH) using long-acting corifollitropin alfa (Elonva^®^), clomiphene, and letrozole is as effective as traditional COH protocols.

**Methods:** This retrospective study was conducted at a single private center in São Paulo, Brazil, including a total of 748 patients. Patients were divided into two groups: Group 1 (n=76) used long-acting corifollitropin alfa (Elonva®), clomiphene, and letrozole, while Group 2 (n=672) used different protocols involving gonadotropins and GnRH antagonists. Group 1 protocol consisted of an initial shot of Elonva® on day 1 followed by daily oral doses of 5 mg of letrozole and 100 mg of clomiphene starting from day 2 until the patient was ready for the trigger. Ultrasound scans were performed on days 5, 7, and 9 to monitor follicular development. Triggering was performed with GnRH agonist and/or hCG when the leading follicle reached >18mm. The total duration of stimulation, the number of ultrasound visits, and the overall patient compliance were recorded. In Group 2, patients followed various protocols involving daily injections of gonadotropins and GnRH antagonists. The specific regimen and doses were determined based on individual patient characteristics and physician discretion. Both groups were compared in terms of the number of oocytes retrieved, mature oocytes, fertilization rates, embryo quality, and pregnancy outcomes.

**Results:** No significant differences were observed between Groups 1 and 2 in terms of age, partner age, fertility factor, and Anti-Mullerian hormone (AMH) levels. Group 2 had a higher number of oocytes and mature oocytes retrieved, averaging 2.2 and 1.5 more per cycle, respectively (*p*<0.001). Group 2 also had more blastocysts and top blastocysts formed, averaging 1.1 and 0.6 more embryos, respectively (*p*<0.001). However, there were no significant differences in maturation rate, fertilization rate, fertilization to blastocyst rate, and fertilization to top blastocyst rate between the groups. Further sub-analysis based on AMH levels showed higher egg and embryo retrievals in Group 2 for AMH levels >1ng/ml and 0.5-1ng/ml. For AMH levels >1ng/ml, Group 2 had an average of 1.6 more eggs and 5.2 more mature eggs retrieved per cycle (*p*<0.001). The embryo and top embryo formation rates were also higher in Group 2, with 0.5 more embryos (*p*=0.003) and 3.1 more top embryos (*p*<0.001) per cycle compared to Group 1. Conversely, in the AMH <0.5ng/ml subgroup, Group 1 showed better results with an average of 0.3 more eggs and 0.6 more mature eggs retrieved (*p*=0.270 and *p*=0.286, respectively), as well as 0.2 more embryos formed (*p*=1.55) and 0.05 more top embryos (*p*=0.72). The alternative protocol of Group 1 demonstrated feasibility and efficacy, particularly for patients with poor ovarian reserve. Although the number of retrieved oocytes and embryos was lower compared to traditional protocols, the patient-friendly nature and reduced cost of the regimen were significant advantages.

**Conclusion:** This alternative protocol using long-acting corifollitropin alfa (Elonva^®^), clomiphene, and letrozole is feasible for COH, especially for patients with poor ovarian reserve. It presents a more cost-effective and patient-friendly alternative compared to traditional protocols involving daily gonadotropin injections and GnRH antagonists. Further studies are warranted to explore the long-term outcomes and pregnancy rates associated with this protocol.

**P-79. Impact of serum progesterone levels prior to frozen embryo transfer on pregnancy outcomes in patients who underwent artificial endometrial preparation**

**Larissa Milane Coutinho^1^, Juliana Polisseni Magalhaes^1^, Fernanda Polisseni Souza^1^, Maria Bernardino Silva^1^, Maria Clara Barroso Moreira^1^, Iara Carlin Torres^1^, Hugo Vitoi Rosa^1^**

^1^Nidus - Juiz de Fora - MG - Brazil

**Objective:** To evaluate, in artificial endometrial preparation for frozen embryo transfer (FET), the impact of the progesterone (P) levels prior to FET on pregnancy outcomes.

**Methods:** We conducted a retrospective cohort study at a private fertility center in Brazil from January 2023 to March 2024. During this period, a total of 132 patients underwent FET using hormone replacement therapy (HRT) with estradiol valerate and vaginal micronized progesterone for endometrial preparation. The serum P levels were measured by Atellica Solution IM 1600 (Siemens) with the chemofluorescence method on the day of FET or the day before. One or two good quality day-5-6 blastocysts were transferred to women with normal uterine cavities and appropriate endometrial thickness (≥ 7mm). When P < 10 ng/ml, patients received progesterone rescue with dydrogesterone. We evaluated mean P values in the overall study population and in subgroups divided according to pregnancy outcomes. We also analyzed P levels in the different age ranges (<34 years old, 34-39 years old, and >39 years old) and compared the pregnancy rates among the groups. Finally, we compared pregnancy outcomes regarding the P threshold of 10.0ng/ml, usually used in literature.

**Results:** The demographic data of the 125 patients revealed that the mean age was 38.7 (±5.5) years old, and the mean body mass index (BMI) was 24.5 (±3.5). All the subgroups considered in the subsequent analysis were similar in terms of demographic information. The general mean P value (n=125) was 13.0±6.6. In patients who had delivery (n=47), the mean P was 13.6±7.6 ng/ml. In patients with biochemistry pregnancy (n=3), miscarriage (n=2), and no pregnancy (n=73), the mean P were 8.1±2.0, 9.0±1.6, and 12.9±6.0, respectively. When considering the different age ranges, the mean P values in patients <34 years old (n=24), 35-39 years old (n=46), and ≥40 years old (n=55) were 11.4±4.5, 13.2±5.3, 13.0±8.0, respectively. Still, the Pregnancy Rates (positive beta-hCG) in the age subgroups were 54.0%, 45.0%, and 32.0%, respectively (*p*=0.97), and Clinical Pregnancy Rates were 50.0%, 43.0%, and 30.0%, respectively (*p*=0.97). When we considered a 10.0ng/ml progesterone cut-off to compare pregnancy rates, eighty-one patients (64.8%) had their progesterone dosage before the transfer ≥10 ng/ml, and 44 (35.2%) had their progesterone dosage below 10ng/ml. The Pregnancy and Clinical Pregnancy Rates were not statistically different in these two groups (43.0% *vs*. 38.0%, *p*=0.95 and 41.0% *vs*. 34.0%, *p*=0.93, respectively).

**Conclusion:** Although Pregnancy and Clinical Pregnancy Rates were slightly higher in patients with *P*≥10 ng/ml than those with *P*<10 ng/ml, there was no statistical significance. This finding is likely due to the progesterone rescue performed in patients with serum *P*<10ng/ml, strengthening the importance of such a strategy. In our study population, women with positive beta-hCG who evolved to delivery had a P mean of around 13.6ng/ml. Conversely, those whose pregnancy failed to develop (biochemistry pregnancy and miscarriage) had lower mean P values (8.1 and 9.0, respectively). Establishing a universal threshold for progesterone in artificial FET cycles and recognizing the P levels below which it is not worth making the rescue are challenging tasks. The varied populational features and local laboratory specificities must be considered and may be decisive for establishing more effective progesterone rescue protocols. Further studies with different and largest populations are urgently needed.

**P-80. Is FREEZING ALL embryos in *In Vitro* Fertilization the best choice for patients?**

**Juliana Polisseni Magalhaes^1^, Larissa Milani Coutinho^1^, Fernanda Polisseni Souza^1^, Marina Bernardino Silva^1^, Maria Clara Barroso Moreira^1^, Iara Carlin Torres^1^, Hugo Toledo Vitoi^1^**

^1^Nidus - Juiz de Fora - MG - Brazil

**Objective:** To compare *in vitro* fertilization (IVF) pregnancy outcomes in patients having their first frozen embryo transfer (FET) after a FREEZE ALL cycle versus patients undergoing the transfer of remaining frozen embryos.

**Methods:** We conducted a retrospective cohort study at a private fertility center in Brazil from January 2023 to March 2024. During this period, a total of 132 patients underwent FET using hormone replacement therapy (HRT) for endometrial preparation. Seven patients were excluded due to missing data. One or two good quality day-5-6 blastocysts were transferred to women with normal uterine cavities and appropriate endometrial thickness (≥ 7mm). The cycles were subdivided into groups for analysis: GROUP 1: First frozen embryo transfer after FREEZE ALL; GROUP 2: Frozen transfer of remaining embryos (in this group, patients have had their first attempt with fresh embryo transfer). Pregnancy rate (positive beta hCG) and Clinical Pregnancy rate were analyzed in the two groups. In a second moment, GROUP 1 was further subdivided: FET of a euploid embryo versus FET without genetic analysis (PGT-A). Pregnancy rate (positive beta hCG) and Clinical Pregnancy rate were analyzed in the two groups. The study was approved by the local Research Ethics Committee. Statistical analyses were performed with the Chi-square test and Fisher test (*p*<0.05).

**Results:** By analyzing the demographic information of the 125 patients, the mean age was 38.7 (±5.5) years old, and the mean body mass index (BMI) was 24.5 (±3.5). GROUPS 1 and 2 were similar regarding demographic data. Eighty-five patients had their first frozen embryo transfer after FREEZE ALL cycles (68.0%), and 40 had their transfer of remaining embryos (32.0%). When compared, the Pregnancy rate was not statistically different in the two groups (49.0% versus 25.0%) (*p*=0.78). Although the Clinical Pregnancy rate was higher in GROUP 1 (45.0%) than in GROUP 2 (25.0%), it was not statistically significant (*p*=0.81). When GROUP 1 was subdivided into cycles with euploid embryos (20.0%) versus non-genetic analyzed embryos (80.0%), we did not observe statistically significant differences either in total pregnancy rate or clinical pregnancy rate (62.0% *versus* 48.0%, *p*=0.88; 56.0% versus 45.0%, *p*=0.90, respectively).

**Conclusion:** Although our results could not prove the benefit of the FREEZE-ALL for all patients, there was a tendency of better clinical pregnancy rates. The genetic analysis, other way, did not show any improvement of pregnancy outcomes in the population studied.

**P-81. Is embryo mitochondrial DNA content a useful marker to predict implantation of euploid embryos?**

**Ana Cristina Allemand Mancebo^1^, Raquel Rocha Orphão^1^, Brunna Stumpo Vaz^1^, Thaísa Damasceno Renovato^1^, Ana Luíza Barbeitas1, Maria do Carmo Borges Souza^1^, Roberto de Azevedo Antunes^1^, Marcelo Marinho de Souza^1^**

^1^Centro de Reprodução Humana Fertipráxis - Rio de Janeiro - RJ - Brazil

**Objective:** Mitochondrial DNA (mtDNA) content measurements do not represent the quantitative mtDNA copy number but rather the ratio of mtDNA to nuclear DNA, depending on the amount of DNA extracted by the biopsy (Paulson, 2022). In addition, mtDNA replication begins specifically in the blastocyst trophectoderm and only later in the inner cell mass (Viotti, 2019), justifying the concept that some embryos actively increase mitochondrial function and energy output as a compensatory response to overcome strained conditions. So, this study aims to estimate if the value of Mitoscore could be useful tool to rank euploid embryos for transfer.

**Methods:** Retrospective, single center study, from 223 embryo transfers performed from January 2023 to March 2024. There were 130 single euploid embryo transfer (SET) cycles previously diagnosed by pre-implantation genetic test (PGT-A), among which 89 cycles from 76 patients to whom it was added a Mitoscore value. The euploid embryo transfer was selected strictly on basis of development and morphology. From the result Implanted or Non implanted, the Mitoscore value of the transfered embryos were compared. Afterwards, to evaluate the influence of the Mitoscore value in morphology of implantated or not implanted embryos, they were divided in 3 groups: Group 1 (4-6AA, AB or BA), Group 2 (3-6 BB) and Group 3 (3-5 any letter C). Finally, the same comparison of Mitoscore values within Implanted and Non implanted embryos was correlated to the day of embryo expansion (Day 5 or 6). Statistical analysis was performed using t-test and *p*<0.05 considered statistically significant.

**Results:** 58 implanted embryos from the 89 (65.1%) and 31 not implantated (34.9%). Patients had similar age in both groups (38.87±3.57 *versus* 39.09±3.07, *p*=0.77) as well as body mass index (24.04±5.94 *versus* 23.84±3.93, *p*=0.86). The general Mitoscore was not different from them (14.90±3.68 *versus* 15.71±3.88, *p*=0.33), even considering the morphology of group 1, n=25 (15.16±3.60 *versus* 12.84±0.49, *p*=0.17); group 2, n=60 (14.47±3.64 versus 16.17±4.12, *p*=0.09) and group 3, n=4 (19.96±1.895 *versus* 17.36±2.85). When correlated to the day of embryo expansion Day 5 (n=58): 12.67±1.37 *versus* 12.76±1.17, *p*=0.79) and Day 6 (n=31): 19.86±1.83 *versus* 19.79±2.17, *p*=0.92), again no differences. A highly significant difference was observed in relation to D5 to D6 implantated embryos (12.67±1.37 *versus* 19.86±1.89, *p*<0.0001) and in relation to D5 to D6 not implantated (12.76±1.17 *versus* 19.79±2.17, *p*<0001).

**Conclusion:** The exact value of mtDNA remains unclear due to controversial results. Continued efforts are essential to analyze more data from various centers to determine the significance of Mitoscore in classifying euploid embryo transfers. In our study, there was no evidence to support selecting embryos for transfer based on Mitoscore, as there were no significant differences in Mitoscore values between implanted and non-implanted euploid embryos. However, a highly significant difference in Mitoscore values was observed between D5 and D6 euploid embryos, consistent with existing literature.

**References**

Paulson RJ. F S Rep. 2022;3:1-2.

Viotti M. J Assist Reprod Genet. 2019;36:1845-1846.

**P-82. Evaluation of euploidy, mosaicism, embryo morphology and days of development: 851 biopsied blastocysts**

**Gabriela Venera^1^, Camila Dutra Souza Francisquini^1^, Samara Artuso Giacomin^1^, Vinicius Bonato Rosa^2^, Alessandro Schuffner^1^**

^1^Conceber - Centro de Medicina Reprodutiva - Curitiba - PR - Brazil

^2^Brown Fertility - United States

**Objective:** This study aims to evaluate 851 embryos from *in vitro* fertilization cycles with preimplantation genetic testing (PGT-A) concerning chromosomal status in different groups of embryo morphology. Additionally, it was analyzed the euploidy rate on different days of development (Day 5, Day 6, and Day 7).

**Methods:** This retrospective observational study involved blastocysts biopsied and analyzed by Next Generation Screening (NGS) between 2016 and 2024. The morphology was evaluated based on inner cell mass and trophectoderm classification from A to C (by Gardner Blastocyst Grade). The embryos were divided into four groups, according Capalbo (2014), as follow: Excellent (AA): n=157, Good (AB/BA): n=192, Average (BB/AC/CA): n=235, and Poor (BC/CB/CC): n=267. The euploidy and mosaicism rates were compared between the groups, as well as the euploidy rate on different development days (Day 5, Day 6 and Day 7). Test-Z for two proportions was used for the comparisons between the groups adopting *p*-value of 0.05.

**Results:** Embryos classified as Excellent had a numerically higher euploidy rate (52.8%; n=83/157) compared to embryos classified as Good (47.4%; n=91/192); however, there was not statistical significance (*p*>0.05). When comparing the euploidy rate of the Excellent group (52.8%) with the Average group (41.3%; n=97/235) and Poor (27.3%; n=73/267), there was a significant difference (*p*<0.05). The euploidy rate for embryos in the Poor group (27.3%) was lower (*p*<0.05) compared to the other groups (Excellent: 52.8%; Good: 47.1%; and Average: 41.3%). The mosaicism rate did not differ significantly (*p*>0.05) between the embryo morphology groups [Excellent: 9.6% (n=15/157); Good: 12.5% (n=24/192); Average: 8.9%; (n=21/235) and Poor: 12.4% (n=33/267)]. Regarding the probability of euploidy on day 5 of development, comparing the morphology groups [Excellent: 51.4% (n=71/138); Good: 49.6% (n=68/137); Average: 38.9% (n=49/126); and Poor: 39.4% (n=28/71)], there was no statistical difference between most groups (*p*>0.05). A slight difference (*p*<0.05) was observed only when comparing Excellent versus Average. For day 6 of development, a lower euploidy rate (*p*<0.05) was observed in embryos from the Poor group (24.2%; n=40/165) compared to the Excellent (63.1%; n=12/19). On day 7 of development, there was not embryos classified as Excellent in this day, and the Good group (100%; n=2/2) had a higher euploidy rate (*p*<0.05) compared to embryos from the Average (14.2%; n=1/7) and Poor (14.7%; n=5/34) groups.

**Conclusion:** Excellent (AA) and Good (AB/BA) quality embryos showed similar euploidy rates when evaluated independently of the day of development, and when individually assessed on days 5 or 6. Poor quality embryos (BC/CB/CC) had a lower euploidy rate in day 6 or after of development. Day 7 embryos had a higher probability of being euploid when classified as Good (AB/BA). The mosaicism rate does not seem to be related to morphology of biopsied blastocysts.

**P-83. Ovarian response using recombinant FSH (rFSH), urinary (uFSH) or Human Menopausal Gonadotrophin (hMG) in 921 cycles for *in vitro* fertilization**

**Nicholas Vinicius Sala Silva^1^, Camila Dutra Souza Francisquini^2^, Samara Artuso Giacomin^2^, Vinicius Bonato Rosa^3^, Alessandro Schuffner^2^**

^1^Faculdades Pequeno Príncipe - Curitiba - PR - Brazil

^2^Conceber Centro de Medicina Reprodutiva - Curitiba - PR - Brazil

^3^Brown Fertility - United States

**Objective:** This study aims to evaluate the relationship between ovarian response rates and different gonadotrophins used in Controlled Ovarian Stimulation (COS), comparing rFSH (Follicle Stimulating Hormone, recombinant), uFSH (Follicle Stimulating Hormone, urinary) and hMG (Human Menopausal Gonadotrophin), regardless of infertility factors. In addition, one of the aims of this study is to identify the best choice, among the 3 gonadotrophins rFSH, uFSH and hMG, for performing COS.

**Methods:** A retrospective observational study was carried out with 921 cycles from couples undergoing assisted human reproduction. They were divided into the following groups: Group 1: hMG (n=314 cycles); Group 2: recombinant FSH (rFSH) (n=560 cycles) and Group 3: urinary FSH (uFSH) (n=47 cycles). In this study, the following were compared: the total dose of gonadotrophins, the number of follicles at the last ultrasound scan before aspiration larger than 14 mm, the number of retrieved and mature oocytes, as well as oocyte retrieval and maturation rates and morphologically normal oocytes rate. The statistical means were compared using the ANOVA test and then Tukey's test at 5% and the rates were compared using the Z-test for two proportions with a statistical p-value of 0.05.

**Result:** Maternal age showed no statistically significant difference between the groups (hMG Group: 36.7±4.1 years; rFSH Group: 36.4±4.5 years and uFSH Group: 36.1±4.5 years), confirming the homogeneity of the groups. With regard to the total dose of gonadotrophin, the hMG group used 2676.8±752.5IU, different (*p*<0.05) from the rFSH (1984.2±791.2 IU) and uFSH (2087.2±755.9IU) groups. The number of follicles at the last ultrasound scan, the number of oocytes retrieved and mature oocytes were lower in the hMG group (8.6±5.9; 7.6±5.5 and 5.1±3.8, respectively, *p*<0.05) when compared to the rFSH group (10.8±6.6; 9.0±6.6 and 6.3±4.6, respectively). They did not differ from the uFSH group for the same variables. The recovery rate was higher (*p*<0.05) in the FSHr group (89.0% n=5073/5699) when compared to the hMG (86.7% n=2430/2800) and uFSH (86.2% n=916/1062) groups. In relation to the rate of oocyte maturity and morphologically normal oocytes, there was no statistical difference between the experimental groups in terms of the rate of oocyte maturity [hMG: 68.0% (n=623/916); rFSH: 70.5% (n=3576/5073) and uFSH: 71.7% (n=1743/2430)] and the rate of morphologically normal oocytes [hMG: 59.8% (n=1005/1680); rFSH: 61.6% (n=2108/3420) and uFSH: 63.4% (n=390/615) (*p*>0.05)].

**Conclusion:** Thus, although the gonadotrophin of choice does not affect oocyte morphology, when the infertility factor is not taken into account FSHr should be the gonadotrophin of choice when compared to hMG, since its recovery rate is higher than the other gonadotrophins.

**P-84. Post-thaw blastocyst culture period before transfer does not influence the clinical outcomes in frozen blastocyst transfer cycles**

**Gabriella Mamede Andrade^1^, Danielle Dalera Araújo^1^, Eduarda Nabinger^1^, Nilo Frantz^1^**

^1^Nilo Frantz Medicina Reprodutiva - Porto Alegre - RS - Brazil

**Objective:** The frozen-thawed embryo transfer (FET) has become a commonly used approach *in vitro* fertilization treatments. Mainly to reduce ovarian hyperstimulation syndrome risk, to preserve the supernumerary embryos and due to the security of embryo vitrification method. Usually, after warming the embryos are transferred into the uterus after short culture of 2 to 5 hours but there is no consensus regarding the post-warm culture period that yields the best clinical results. In this study, we aimed to evaluate if three different post-thaw culture periods affected the clinical outcomes of FET.

**Methods:** This single center retrospective cohort study included 927 frozen-thawed blastocyst transfer cycles from January 2022 to December 2023. The cycles were divided into three groups based on the culture duration after thawing: short, until 3 hours (n=40); intermediary, between three and four hours (n=420) and long, more than 4 hours (n=467). Patient’s characteristics (age, body mass index (BMI), AMH, primary infertility factor), FET cycle variables (eggs origin and age, endometrial preparation and thickness, catheter used, developmental day of embryo transferred, embryo expansion after thawing,) and clinical outcomes were analyzed. Rates of positive beta human chorionic gonadotropin test (b-hCG+), clinical pregnancy, ongoing pregnancy/delivery and miscarriage were compared among groups. Data analysis was performed using GraphPad Prism 6. ANOVA analysis with Student t-test and Kruskal-Wallis test with Fisher’s exact post-test were used when appropriated; p value was considered significant ≤0.05.

**Results:** The means of patient age at transfer, BMI and AMH dosage and primary infertility factor were not different between groups. The same was observed regarding transfer cycle variables as oocyte age, endometrial preparation and thickness, proportion of PGT-A cycles, catheter used, embryo expansion after thawing and embryo developmental day at freezing and quality, number of embryos transferred; suggesting that the three experimental groups are homogeneous for the main features that, normally, interfere with clinical outcomes. Through statistical analysis, our results demonstrated that different post-thaw culture duration (short, intermediary and long) had similar rates of implantation (54.2%, 60.9% and 51.0%), b-hCG+ (75.0%, 69.8% and 64.5%), biochemical pregnancy (10.0%, 4.8% and 8.6%), clinical pregnancy (62.5%, 64.8% and 55,9%), ongoing/delivery (45.0%, 52.9% and 47.8), miscarriage (28.0%, 18.0% and 13.8%) and twin pregnancy (4.0%, 10.3% and 35.6%) in FET. Although not statistical significant the ongoing/delivery rate had a p value of *p*=0.0667 that deserves attention as it may be clinically important and should be investigated in future studies.

**Conclusion:** Different post-thaw embryo culture timings do not negatively impact clinical outcomes. One advantage of these results is the flexibility to better organize the laboratory workflow. Due to the retrospective nature of the study, the low number of cycles, and the many variables involved, other studies are needed to validate the results.

**P-85. Not too cold, not too warm: The air temperature affects good quality blastocyst rate**

**Bruna Campos Galgaro^1^, Luiza da Silva Rodrigues^1^, João Sabino Lahorgue Cunha Filho^1^**

^1^Centro de Reprodução Humana Insemine - Porto Alegre - RS - Brazil

**Objective:** To evaluate if the good quality blastocyst rate is affected by the air temperature of the *in vitro* fertilization (IVF) laboratory.

**Methods:** A retrospective study including all fresh intracytoplasmic sperm injection (ICSI) cycles from January to December 2023 was conducted in a single center. IVF laboratory air temperature was measured at fertilization time. Fertilizations performed in different temperatures were compared, as control (22-25.9ºC) and extreme (<22ºC or ≥26ºC) groups. The mean age of patients, AMH (ng/mL), and the number of oocytes retrieved were assessed. The primary outcome was good quality blastocyst rate, and the secondary were: fertilization, good quality cleavage embryo and blastulation rates. Statistical analysis was conducted by paired test with multiple linear regression. Results were significant when *p*<0.05.

**Results:** In total, 173 ICSI cycles were enrolled into two groups: 142 were performed in the air temperature range of 22-25.9ºC (control group) and 31 in temperatures lower than 22ºC (n=10) or higher than 26ºC (n=21) (extreme group). There was no statistical difference (values expressed as mean±SD) for the mean age of patients 36.08±4.84 *vs*. 35.97±3.99 (*p*=0.906), AMH 2.58±2.74 *vs*. 2.31±1.53 (*p*=0.595) and the number of oocytes retrieved 8.25±7.54 *vs*. 9.64±6.09 (*p*=0.335) for control and extreme groups, respectively. Normal fertilization rate was not statistically different 76.1%±0.25 *vs*. 78.6%±0.19 (*p*=0.610) between groups, as well as day-three good quality embryo 54.2%±0.33 *vs*. 45.1%±0.29 (*p*=0.157) and blastulation rates 49.9%±0.32 vs. 48.5%±0.31 (*p*=0.859). However, a statistically significant reduction of good quality blastocyst rate was observed, 16.5%±0.27 of the extreme group compared to 39.8%±0.31 of the control temperature [OR: 0.233 (0.054-0.411)]. This result was confirmed by multiple linear regression, indicating that IVF lab air temperature was associated with good blastocyst rate (*p*=0.024).

**Conclusion:** The good quality blastocyst rate is greatly affected by the air temperature of the IVF laboratory at the time of ICSI, therefore temperatures lower than 22ºC and higher than 26ºC should be avoided.

**P-86. Male fertility preservation: Effect of malignancy on sperm quality**

**Bruna Campos Galgaro^1^, Luiza da Silva Rodrigues^1^, João Sabino Lahorgue Cunha Filho^1^**

^1^Centro de Reprodução Humana Insemine - Porto Alegre - RS - Brazil

**Objective:** To analyse if the sperm quality is affected by male malignancy in patients undergoing fertility preservation.

**Methods:** A retrospective study including male patients from January to December of 2023 of a single fertility center. The study included male patients for sperm cryopreservation due to malignancy without metastasis (study group), and the control group was composed of patients evaluated before *in vitro* fertilization (IVF) treatment, matched 1:2. The primary outcome was total sperm count (x106), and the secondary outcomes were: volume (mL), sperm concentration (x106/mL), total motility (%), progressive motility (%) and non-progressive motility (%). Values were expressed as mean±SD, statistical analysis was performed by paired analysis and significance was accepted when *p*<0.05.

**Results:** In this period, 35 patients underwent fertility preservation whereas 70 had pre IVF treatment semen analysis. Patients for cryopreservation were younger than those for seminal assessment: 31.97±6.38 *vs*. 38.30±6.94 (*p*<0.001). No statistical difference was observed in sperm volume 2.77±1.54 vs 2.92±1.63 (*p*=0.655), concentration 36.74±42.68 *vs*. 39.60±49.33 (*p*=0.771), and total sperm count 82.01±82.75 *vs*. 113.08±153.46 (*p*=0.266) for cryopreservation and pre IVF patients, respectively. Although total motility was also not different 64.03±17.58 vs. 59.56±22.87 (*p*=0.291), progressive and nonprogressive motility presented an altered pattern. Progressive motility was 48.31±19.36 *vs*. 36.79±21.22 (*p*=0.008), and nonprogressive motility was 16.09±8.90 *vs*. 22.64±13.54 (*p*=0.010), for malignancy patients and fertility assessment. A logistic regression controlling the groups for age, volume and concentration showed that only age was significant (*p*<0.001), therefore, although there was a difference in progressive motility, this is attributed to the mean age of patients.

**Conclusion:** The presence of male malignancy did not affect semen quality in non-metastatic patients undergoing fertility preservation in this study. Therefore, a semen cryopreservation protocol must be encouraged for male patients before antineoplastic treatment.

**P-87. Morphological abnormalities and maturation rate of oocytes retrieved from women affected by endometriosis**

**Bruna Renata Caitano Visnheski^1,2^, Kahisa Natiele Fontana^1^, Viviane Margareth Scantamburlo^1^, Lidio Jair Ribas Centa^1^**

^1^Centro de Reprodução Humana Insemine - Porto Alegre - RS - Brazil

^2^Centro de Reprodução Humana Insemine - Porto Alegre - RS - Brazil

**Objective:** To evaluate if the good quality blastocyst rate is affected by the air temperature of the *in vitro* fertilization (IVF) laboratory.

**Methods:** A retrospective study including all fresh intracytoplasmic sperm injection (ICSI) cycles from January to December 2023 was conducted in a single center. IVF laboratory air temperature was measured at fertilization time. Fertilizations performed in different temperatures were compared, as control (22-25.9ºC) and extreme (<22ºC or ≥26ºC) groups. The mean age of patients, AMH (ng/mL), and the number of oocytes retrieved were assessed. The primary outcome was good quality blastocyst rate, and the secondary were: fertilization, good quality cleavage embryo and blastulation rates. Statistical analysis was conducted by paired test with multiple linear regression. Results were significant when *p*<0.05.

**Results:** In total, 173 ICSI cycles were enrolled into two groups: 142 were performed in the air temperature range of 22-25.9ºC (control group) and 31 in temperatures lower than 22ºC (n=10) or higher than 26ºC (n=21) (extreme group). There was no statistical difference (values expressed as mean±SD) for the mean age of patients 36.08±4.84 *vs*. 35.97±3.99 (*p*=0.906), AMH 2.58±2.74 *vs*. 2.31±1.53 (*p*=0.595) and the number of oocytes retrieved 8.25±7.54 *vs*. 9.64±6.09 (*p*=0.335) for control and extreme groups, respectively. Normal fertilization rate was not statistically different 76.1%±0.25 *vs*. 78.6%±0.19 (*p*=0.610) between groups, as well as day-three good quality embryo 54.2%±0.33 *vs*. 45.1%±0.29 (*p*=0.157) and blastulation rates 49.9%±0.32 vs. 48.5%±0.31 (*p*=0.859). However, a statistically significant reduction of good quality blastocyst rate was observed, 16.5%±0.27 of the extreme group compared to 39.8%±0.31 of the control temperature [OR: 0.233 (0.054-0.411)]. This result was confirmed by multiple linear regression, indicating that IVF lab air temperature was associated with good blastocyst rate (*p*=0.024).

**Conclusion:** The good quality blastocyst rate is greatly affected by the air temperature of the IVF laboratory at the time of ICSI, therefore temperatures lower than 22ºC and higher than 26ºC should be avoided.

**P-88. Conservative treatment of ovarian explosion after oocyte retrieval for *in vitro* fertilization: Case report**

**Caroline Medina Calvão Caser^1^, Anna Flávia Magalhães Castrillon de Macedo^1^, Natalia Ivet Zavattiero Tierno^1^, Mariana Fonseca Roller Barcelos^1^, Nina Rotsen Santos Ferreira^1^, Kamilla Monteiro Plácido^1^, Taciana Fontes Rolindo^1^, Leilane Gabriele Nolêto Lima^1^, Larissa Maciel Ribeiro^1^**

^1^Hospital Materno Infantil Dr. Antônio Lisboa - Brasília - DF - Brazil

**Objective:** To report a case of ovarian explosion, a rare complication associated with ovarian stimulation, early follicular rupture and oocyte retrieval, its conservative treatment and clinical evolution.

**Methods:** Descriptive observational case report study.

**Results:** The patient was a 30-year-old woman, nulligest, in a same-sex relationship undergoing *In Vitro* Fertilization (IVF) at another service. Controlled ovarian stimulation was undertaken using menotropin (225 IU/day) for thirteen days. Anastrozole (1mg/day) and clomiphene (50mg/day) were associated from the first day of gonadotropin and the use of desogestrel (150mcg/day) from the fourth day of protocol forward. The trigger was performed with gonadotropin-releasing hormone agonist, when the transvaginal ultrasound showed twenty-two follicles larger than 15mm, thirty-six hours before the oocyte retrieval. Even before the procedure began, the patient reported intense and progressive abdominal pain for two days and a moderate amount of free fluid was observed in the cavity. The oocyte retrieval occurred without complications and 11 oocytes from 20 dominant follicles were collected. At the end of the procedure the patient remained in pain and developed signs and symptoms of hemorrhagic hypovolemic shock, such as abdominal distension, tachycardia (105 bpm), hypotension (blood pressure 97/49mmHg), pallor, nausea and vomiting. She was immediately transferred to a surgical hospital center and underwent an exploratory laparotomy. An inventory of the cavity revealed enlarged ovaries, active bleeding with approximately 1.5 liters of blood in the abdominal cavity and ovarian explosion appearance with several points of laceration with diameters varying from 2.0 up to 5.0 cm and loss of ovarian tissue. The ovarian lesions were incompatible with damage caused exclusively by the puncture procedure, and were probably also due to early follicular rupture. The ovarian tissue was quite friable and the ovarian lesions were repaired with simple polyglactin 2.0 and 3.0 sutures. Hemostasis was also performed with the oxidized regenerated cellulose hemostatic agent. The patient required hemodynamic stabilization with vasoactive drugs and massive transfusion protocol. Despite the severity, the patient had a good postoperative evolution and was discharged from the hospital in one week. The control ultrasound made after four weeks was normal and both ovaries were well perfused with mild signs of residual ovarian stimulation.

**Conclusion:** Complications associated with oocyte retrieval are infrequent and present in only 0.76% of cases. Ovarian explosion is a rare and potentially fatal form of ovarian injury and to date there is only one case report described, according to a review of the literature. Its pathophysiology remains unknown, but the main hypothesis is that the loss of parenchyma and tissue damage associated with multiple lacerations resulted from follicular rupture in the stimulated ovary, aggravated by ovarian puncture. Its main clinical manifestation is acute hemorrhagic abdomen and surgical treatment depends on the experience of the gynecological surgeon and patient's clinical conditions. In this work, the patient underwent conservative treatment, with a favorable final outcome and preservation of both ovaries. This case highlights the need for rapid diagnosis and surgical intervention to prevent serious adverse consequences such as oophorectomy and death. These patients generally do not have defined offspring and the best treatment should preserve the women's reproductive potential.

**P-89. Sperm sorting by rheotaxis may increase the rate of frozen blastocysts eligible for transfer in Assisted Reproduction treatments**

**Giovanna Santos Cavalcanti^1^, Henrique Penna Fazão^1^, Camila Sommerauer Franchim^1^, Marcello Antonio Signorelli Cocuzza^1^, José Maria Soares Junior^1^, Edmund Chada Baracat^1^, Pedro Augusto Araújo Monteleone^1^**

^1^Hospital das Clínicas da Faculdade de Medicina da Universidade de São Paulo - São Paulo - SP - Brazil

**Objective:** To evaluate whether the use of rheotaxys sperm sorting (RSS) in extended drop increases the rate of frozen blastocysts originated by *in vitro* fertilization (IVF) compared to standard extended drop sperm sorting techniques for intracytoplasmic sperm injection (ICSI).

**Methods:** This retrospective study analyzed 3152 frozen blastocysts from 906 patients below 38 years old, who underwent IVF treatment from 2012 to 2023. According to these data, two groups were created: Group 1 (2188 frozen blastocysts from 587 patients from 2017 to 2023 using RSS technique in extended drop) and Group 2 (964 frozen embryos from 319 patients from 2012 to 2016 using standard sperm sorting in extended drop). We performed Student t-test to analyze statistical significance between the groups.

**Results:** Group 1 showed a higher average number of frozen blastocysts 3.73 (2352/631), compared to 3.02 (962/342) from Group 2; and t-test showed a significant statistical difference (*p*=0.002) when comparing both groups.

**Conclusion:** We concluded that using RSS technique may increase the rate of frozen blastocysts eligible for transfer when compared to standard sperm sorting techniques, but more studies are necessary to achieve more comprehensive data.

**P-90. Melatonin increases VEGF expression in granulosa cells *in vitro*: Potential mechanism to improve oocyte quality in infertile patients undergoing IVF**

**Giovanna Santos Cavalcanti^1^, Kátia Cândido Carvalho^1^, Cecília Silva Ferreira^1^, Pedro Augusto Araújo Monteleone^1^, Edmund Chada Baracat^1^, José Maria Soares Junior^1^**

^1^Laboratório de Ginecologia Estrutural e Molecular, Departamento de Obstetrícia e Ginecologia, Hospital das Clínicas da Faculdade de Medicina da Universidade de São Paulo - São Paulo - SP - Brazil

**Objective:** To analyze the expression of VEGF in granulosa cells (GCs) of infertile women after the action of melatonin.

**Methods:** GCs were obtained from nine infertile women below 38 years old. These cells were cultured in 75 cm2 flasks supplemented with 10% fetal bovine serum (FBS). The cells were treated for 72 hours with different concentrations of melatonin a) Control (no melatonin treatment); b) 0.1 µM; c) 10 µM and d) Vehicle (reference group, melatonin diluent). The Trizol method and TaqMan^®^ PCR array detection system was used for detecting the genes VEGFA, VEGFB and VEGFC obtained total RNA.

**Results:** Administration of melatonin at low concentration (0.1 µM) when compared to high concentration (10 µM) induced greater VEGFA (Fold Regulation [FR] 0.1 µM FR: 1.39 and 10 µM RF: 1.37), VEGFB (0.1 µM FR: 1.25 and 10 µM FR: 1.16) and VEGFC (0.1 µM FR: 1.10 and 10 µM FR: -1.10) gene expression.

**Conclusion:** Melatonin treatment modulates VEGF of GCs from infertile women undergoing *in vitro* fertilization. Doses of melatonin can reduce oxidative stress, protect GCs from atresia and improve oocyte quality, regulating the expression of some genes. This hormone can positively or negatively affect ovarian angiogenesis.

**P-91. Ultra fast blastocyst warming, a preliminary report**

**Ricardo Azambuja^1^, Fabiana Wingert^1^, Shana Flach^1^, Alice Tagliani-Ribeiro^1^, Andreia Silva^1^, Marta Hentschke^1^, Alvaro Petracco^1^, Mariangela Badalotti^1^**

^1^Fertilitat- Centro de Medicina Reprodutiva - Porto Alegre - RS - Brazil

**Objective:** To evaluate the vitrified blastocyst viability after one-step warming protocol.

**Methods:** Observational study performed at a reproductive medicine center. A total of 45 aneuploidy blastocyst embryos were analysed. All embryos were aneuploidies, varying from monosomies to chaotic stages, and allowed to be discarded. The samples were divided into two groups according to the thawing protocol: G1) one-step protocol (n=33) and G2) three step protocol (control) (n=12). The one-step protocol (ultra fast warming) consists of placing the embryos in the Thawing Solution at 37°C during one minute, washing, and placing it in a culture media (Global medium, USA) with HSA10% for 24 hs. The three step protocol is the traditional warming protocol decreasing the sucrose concentrations from 1M, 0.5M, and 0M, thawing, dilution, and warming solution, respectively. Following thawing all embryos were also placed in the culture media (Global medium, USA) with HSA10% for 24 hs. The blastocyst reexpansion was analyzed at 2, 4, 5 and 24 hours after thawing. Qui-square tests were applied to compare reexpansion between the 2 groups, considering *p*<0.05 statistically significant.

**Results:** The following reexpansions rate between G1 (one step) *versus* G2 (three step) were: at 2 hours 54.5% (18/33) *vs*. 50.0% (6/12) (*p*=0.786), at 4 hours 66.7% (22/33) vs. 66.7% (8/12) (*p*=1.0), at 5 hours 69.7% (23/33) vs. 75.0% (9/12) (*p*=0.728) and at 24 hours 81.8% (27/33) vs. 91.7% (11/12) (*p*=0.420).

**Conclusion:** The results suggested that the ultra fast warming using only one step of thawing seems to be as efficient as the three-step warming protocol. Since this is a preliminary report, it is important to highlight that this hypothesis should be tested in larger data, including embryos that will be transferred. This technique will decrease the workload of cryopreservation procedures and thus should be considered in a daily basis laboratory.

**P-92. Live birth rate in frozen blastocyst transfers on the 5th or 6th day from the start of progesterone**

**Rodopiano de Souza Florêncio^1^, Mirian Rodrigues Borges^1^, Eduardo Camelo de Castro^1^, Mylena Naves de Castro Rocha^1^, Jane Porfírio Rocha^1^, Gustavo Cardoso Borges^1^, Corival Lisboa Alves de Castro^1^, Marta Curado Carvalho Franco Finotti^1^, Vinícius Alves de Oliveira^1^**

^1^Humana Medicina Reprodutiva - Goiânia - GO - Brazil

**Objective:** Primary: To evaluate the ongoing pregnancy (ONG) and live birth rates (LBR) in frozen embryo transfers (FET), comparing the results of top blastocyst transfers on the 5th day (D5) from the start of using micronized or injectable progesterone (PRG) (99-104 hours) versus the 6th day of use (D6) (123-128 hours). Secondary: To evaluate clinical pregnancy and miscarriage rates in both groups.

**Methods:** FETs performed from January 2016 to January 2017 (PRG D5) and February 2017 to December 2018 (PRG D6). Initial number up to 39 years old = 732. Number of FETs meeting the inclusion criteria: 550 Number of FETs meeting the exclusion criteria: 293 Final number: 2016 (64); 2017-18 (229) Inclusion criteria: - Patients who underwent FET up to 39 years old during the period: 732 - FET with 1 blastocyst ≥ 3BB (top) with or without genetic testing or 2 blastocysts on D5, D6 from the start of PRG (297) - Included all protocols used for artificial endometrial preparation (297) - All selected FETs above, regardless of previous failures (297) - All FETs, regardless of the cause of infertility and endometrial thickness measurement (297) cExclusion criteria: - Difficult transfer: 0 - Absence of follow-up post FET (04) Final number of top blastocyst FETs: 293 Experimental Group (EG): Patients who underwent thawed FETs with 123-128 hours of PRG use, with the morphological characteristics described in the objective (229) Control Group (CG): Patients who underwent thawed FETs with 99-104 hours of PRG use (64) Thawing and FET Timing: The embryos vitrified with a closed system were thawed in the morning between 7:00 and 8:00 am and transferred from 11:00 am to 5:00 pm. FETs were performed with endometrial visualization via abdominal ultrasound. After FET, patients were advised to walk and were discharged. Post-FET, a quantitative beta-hCG test was requested to be performed 13 to 14 days after FET. Terms used in results: - Positive beta-hCG with beta ≥ 40 mIU/mL 13 to 14 days post FET; - Chemical miscarriage: loss after positive beta but without visualization of a gestational sac; - Clinical pregnancy: gestational sac visualized 20-22 days post FET; - Clinical miscarriage: miscarriage up to 22 weeks of gestation; - ONG: pregnancy with good progression at 11-12 weeks of gestation; - LBR: births after 22 weeks of gestation. **Results:** Variables that could interfere with the results and were compared between the two groups showed no statistical differences. The beta+ rate was 43.7% in the CG and 45.4% in the EG, OR 0.9348 (0.5425-1.641) *p*=0.8873. The chemical miscarriage rates were similar. The clinical pregnancy rate was 39.1% in the CG and 41.5% in the EG, OR 0.9042 (0.7782-2.392) *p*=0.7749. The clinical miscarriage rate was 12% in the CG and 21.1% in the EG, OR 0.5114 (0.1499-1.888) *p*=0.3999. ONG/LBR was 34.4% in the CG and 32.8% in the EG, OR 1.076 (0.6078-1.956) *p*=0.8807.

**Conclusion:** FET on D6 of PRG showed statistically similar results compared to D5, demonstrating that the implantation window lasted 29 hours (99-128 hours) post PRG start in artificial cycles.

**P-93. Sperm DNA fragmentation rate according to the patient Body Mass Index (BMI)**

**Julia Tomaz^1^, Kahisa Natiele Fontana^1^, Mayara de Fatima Frazão Patussi^1^, Tiago Cesar Mierzwa^1^, Lidio Jair Ribas Centa^1^**

^1^Androlab - Clínica da Fertilidade - Curitiba - PR - Brazil

**Objective:** To verify whether there are differences in DNA fragmentation rates in patients who underwent sperm analysis according to Body Mass Index (BMI).

**Methods:** A retrospective analysis was carried out with 245 patients who underwent the DNA Fragmentation test between November of 2017 and May 2024. These patients were divided into 5 groups according to BMI, which was calculated using weight and height data collected using forms given during the exam. The fragmented DNA average was calculated for each group.

**Results:** Group 1 (BMI=18.5 to 24.9) included 57 patients and presented an average fragmentation rate of 32.92%; group 2 (BMI=25 to 29.9) with 121 patients and a rate of 37.06%; group 3 (BMI=30 to 34.9) with 51 patients and a rate of 35.99%; group 4 (BMI=35 to 39.9) with 13 patients and a rate of 38.86% and group 5 (BMI>40) with 3 patients and a rate of 40.71%.

**Conclusion:** The affinity between a higher rate of seminal DNA fragmentation and overweight and obese patients has already been reported in several studies in the literature. Following this panorama, in this analysis it was possible to observe an increase in the percentage of spermatozoa with DNA fragmentation at the individuals as the BMI increased, with a difference of approximately 8% between patients with adequate weight (group 1) and patients with level III obesity (group 5). Therefore, it is important that body weight is taken into account when carrying out infertility investigations through fragmentation examination. Furthermore, it seems really relevant and worrying that the majority (76.7%) of the individuals are overweight or obese, which has a direct impact on fertility in general.

**P-94. Comparison of clinical results between progestin primed ovarian stimulation and GnRH antagonist protocol in PGT cycles: A single center experience**

**Priscila Pereira da Cunha Scalco^1^, Gabriella Mamede Andrade^1^, Eduarda Nabinger^1^, Mariana Lopes Santos^1^, Nilo Frantz^1^**

^1^Nilo Frantz Medicina Reprodutiva - Porto Alegre - RS - Brazil

**Objective:** In recent years, many studies have shown that progestins were as effective as GnRH antagonists in preventing LH surges during controlled ovarian stimulation and shown that the response to ovarian stimulation is similar. However, few studies compared the clinical outcomes of GnRH antagonists and progestin-primed ovarian stimulation (PPOS) protocol in pre-implantation genetic testing (PGT) cycles; and it appears to be the most appropriate model for comparing both protocols. Based on that, this study aims to compare clinical outcomes of PGT cycles between PPOS and GnRH antagonist protocols in different populations.

**Methods:** This was a single center retrospective cohort study. Were included 391 patients who underwent euploid embryo transfers from January 2022 to December 2023. In 168 patients, the oocyte retrieval cycles were performed with PPOS protocol and 223 with GnRH-antagonist protocol. The patients characteristics as age, BMI, AMH dosage and infertility factor and transfer cycle outcomes as implantation, βHCG, biochemical, clinical pregnancy, ongoing/delivery, miscarriage and twins rates were compared. Data analysis was performed using GraphPad Prism 6. Student t-test and Fisher’s exact test were used when appropriated; p value was considered significant ≤0.05.

**Results:** There was no significant difference of baseline characteristics between the two groups. The single embryo transfer was performed in 157 (93.4%) patients in PPOS protocol and 209 (93.7%) in GnRH antagonists. The βHCG positive rate and clinical pregnancy rate were, respectively, 65.4% and 62.5% in PPOS protocol and 65.9 and 59.2% GnRH antagonist protocol with no statistical difference between groups. Biochemical pregnancy rate was 7.1% and miscarriage rate was 8.6% in PPOS protocol whereas in GnRH antagonists was 6.7% and 14.4%. The ongoing pregnancy/delivery rates of PPOS group appeared to be higher than that of GnRH antagonists 56.5% vs. 50.2% but without statistical difference (*p*=0.2213).

**Conclusion:** Compared with GnRH antagonist, ovarian stimulation with PPOS protocol does not affect clinical results in transfer cycles with euploid blastocysts suggesting that PPOS is an appropriated alternative for ovarian stimulation method for patients undergoing *in vitro* fertilization in PGT cycles.

**P-95. Comparison of karyotype with exome and CGH array in cases altered in prenatal screening exams**

**Monique Danielle Cambuy^1^, Jeferson Santos Souza^1^, Renata Sabrina do Nascimento Campos^1^, Odair Azevedo da Silva Junior^1^, Maria Elisa Sportello^1,^ Wagner Antonio da Rosa Baratela^1^, Mario Henrique Burlacchini Carvalho^1^, Aline dos Santos Borgo Perazzio^1^, Maria de Lourdes Lopes Ferrari Chauffaille^1^**

^1^Grupo Fleury - São Paulo - SP - Brazil

**Objective:** Prenatal diagnosis (DPN) aims to determine the condition of the fetus. With the DPN result in hand, appropriate management of the remainder of the pregnancy is planned, possible complications for childbirth or postpartum are anticipated, and possible unviability of the current or future pregnancies. Without such intervention, there may be an undesirable outcome for the child, the mother, or both. DPN includes non-invasive exams (ultrasound, research of circulating fetal cells in maternal blood (NIPT) and biochemical tests) and invasive exams (amniocentesis, chorionic villus sampling and fetal blood). Several advances have allowed the detection of genetic abnormalities in DPN, as three fundamental tests are carried out using the amniotic fluid (LA) or chorionic villus (VC); the karyotype, which identifies chromosomal changes; exome sequencing, which evaluates coding sequences involved in the production of proteins necessary for the functioning of the human body and detects errors that lead to genetic diseases; and CGH array, which checks the variation in the number of copies of chromosomal regions, compared to a reference sample, demonstrating unbalanced, total and partial numerical and structural aberrations. The present study aimed to compare the results of karyotype with exome and/or CGH in DPN.

**Methods:** A search was carried out in the laboratory's database, from January 2019 to May 2024, for patients who underwent DPN because they were hypothesized to have fetal malformations, increased nuchal translucency (NT) or cardiac changes in non-invasive screening. 42 cases that had karyotype, exome and/or CGH results in the same period were selected to compare the results. Patient data was made anonymous and non-identifiable, respecting ethical aspects and the LGPD.

**Results:** The karyotype was made from cultured amniocytes, blocked in metaphase, stained for G band, allowing the detection of numerical and structural aberrations. Exome capture was by next generation sequencing. The average age of the patients was 35.4 years. Of the 42 cases, 10 (23.8%) had an altered karyotype; CGHa was performed in 19 cases, of which 3 (15.7%) were abnormal; and 27 had an exome, of which 7 (25.9%) were abnormal. There was agreement of normal results in 28 cases and altered results in 8. In one case, the karyotype demonstrated changes that were not detected by other methods, because it was a balanced translocation. In 3 cases, the exome showed changes that were not detected by other methods (Noonan´s syndrome and microdeletions). The association of the different methods allowed the identification of a total of 13 (30.9%) of altered cases. Each of these methods has its specificity and limitations. The karyotype detects chromosomal changes of up to 5Mb, while CGH demonstrates microdeletions and insertions, while the exome shows gene changes.

**Conclusion:** In conclusion, it is clear that the use of complementary methods is relevant for detecting aberrations for DPN.

**P-96. The correlation between a patient's journey and their decisions to choose and switch clinics**

**Maria Augusta Tamm^1^, Melissa Cavagnoli^1^, Amanda Volpato Alvarez^1^, Vickie White Loureiro Souza^1^, Vitória Guelli Ambrósio^1^, Gustavo Teles^1^, Maite del Collado^2^**

^1^Clínica Hope - São Paulo - SP - Brazil

^2^Science Creating Lives - São Paulo - SP - Brazil

**Objective:** There is a prevailing hypothesis that the frequent changes of clinics and physicians reported by assisted reproduction (AR) patients result more from inadequate patient experiences during treatment than from the technical knowledge of the doctors. Therefore, the objective of the present study was to evaluate the factors considered by patients when choosing a clinic or a particular physician to start their treatment and to continue with it beyond the first attempt at conceiving a baby.

**Methods:** A form composed of nine short questions about the treatment journey was administered to patients of the clinic between April and June of 2024. These questions were designed to assess the factors involved in the patient's overall positive or negative experience: (1) How many AR cycles have you undergone? (2) How many of these cycles were performed in other clinics? (3) What was the main reason behind your decision in changing clinics? (4) How did you find out about clinic? (5) What would you say it is our main advantage compared to others? (6) If you do not achieve pregnancy on your first attempt, would you be willing to ty again with us? Why? (7) Describe some positive aspects about the clinic (8) Describe some negative aspects about clinic (9) Rate overall your experience at clinic with a score from 0 to 10. The obtained results were subjected to descriptive analyses and frequency rates were determined.

**Results:** Seventy-three patients completed and submitted the forms, with 73.23% (52/73) consisting of patients who had undergone more than one AR cycle, averaging 1.6 cycles per patient. Among these, 27 patients came from other clinics, and 23 described the reasons that motivated them to switch clinics. The main reasons were poor patient care and support (43.48%), followed by unsatisfactory medical conduct (26.09%). The primary reason for attending our clinic was referrals by other patients or physicians (86.30%). Additionally, 86.96% of these patients mentioned that patient care and support were major positive aspects of our clinic. Correspondingly, out of the 67 patients who responded about staying at the clinic after a negative outcome, 63 expressed their desire to try again with us (94.03%). Of these, 40 patients provided their motivations for staying, with 52.5% (21/40) valuing confidence in their physician, 37.5% (15/40) citing care and support as their reason, 7.5% (3/40) acknowledging that failure is an expected outcome, and one person (2.5%) mentioning that the most important factor was their physician’s technical knowledge.

**Conclusion:** The preliminary results of this descriptive study indicated that, more than medical competence and technical knowledge, patients undergoing assisted reproduction treatment prioritize care, support, and confidence in both the treatment and the professionals involved, considering these factors fundamental in their decision when choosing a clinic and continuing their engagement with it. Additionally, surveys using forms can serve as a fast and easy feedback assessment tool to measure the quality of a patient's journey in an AR clinic.

**P-97. Molecular analysis of the products of conception after miscarriage in the first trimester**

**Natalia Fagundes Cagnin^1^, Paula Regina Queiroz Estrada^1^, Camila Cristina W Dantas de Souza^1^, José Miravet-Valenciano^2^, Lorena Rodrigo^3^, Bruno Coprerski^1^, Carmen Rubio^2^, Marcia Riboldi^1^, Federico Valdez^4^**

^1^Igenomix Brazil, Laboratory of Genetic Medicine - São Paulo - SP - Brazil

^2^Igenomix SLU, R&D - Spain

^3^Preimplantation Genetic Testing for Aneuploidies (PGT-A) Department, Igenomix Spain Lab S.L.U. - Spain

^4^Igenomix Peru, Global Operations Genetic Services - Peru

**Objective:** Genetic testing of products of conception (POC) has been proposed as a tool to be used in the evaluation of patients with recurrent pregnancy loss (RPL). Numerical chromosomal abnormalities in the fetus are reported to be the main cause of early pregnancy loss, despite the known existence of other causes and risk factors. The object of this study was to describe the incidence of different numerical chromosomal abnormalities and determine the incidence of maternal cell contamination (MCC) by different collection methods analyzing molecularly fetal cells and maternal blood cells.

**Methods:** A total of 1509 fetal samples from POC received in our laboratory between January 2020 and December 2023 were evaluated using NGS technology that screens all 46 chromosomes to determine aneuploidy detection. Comparison of POC samples with DNA extracted from maternal blood based on Sanger sequencing of STRs for Human Identification was used to determine whether the origin of the material analyzed by the POC test was strictly fetal. A descriptive analysis of the incidence of chromosomal abnormalities was performed and the association between the fetal material collection method and the result of maternal contamination was verified by the chi-square adjusted standardized residual test. The odds ratio for each coefficient and the confidence interval at 95% were calculated to analyze the effect of fetal material collection method on MCC.

**Results:** Of the total number of samples analyzed, 1406 corresponded to miscarriage in the first trimester of pregnancy: 402 (28.6%) euploids; 658 (46.8%) aneuploid (555 cases with single or multiples trisomy, 92 with single or multiples monosomy and 11 with trisomy and monosomy), 82 (5.8%) with altered ploidy (2 haploids, 79 triploids, 1 tetraploid); 259 (18.4%) with MCC, 5 (0.35%) non-informatives. The average maternal age for cases within chromosomal abnormalities was 39 years old, while for euploid cases it was 38.4 years old. The most frequently altered chromosomes were chr22 (n=130), chr16 (n=115), chr15 (n=83), chr21 (n=70), chr13 (n=35), chr18 (n=27), and sexual chromosomes (n=78). Among first trimester miscarriages, 1018 (72,4%) cases were natural conception, 365 (26%) cases of ART and 23 (1.6%) unreported cases. Euploid embryo implantation after preimplantation genetic testing for aneuploidy (PGT-A) was reported for 107 ART cases and in 80 of them the POC result was euploid, confirming the previously result, 19 presented MCC, 5 cases showed ploidy alteration that were not possible to detect by the PGT-A test and only 3 aneuploids cases, two of which presented mosaic abnormalities. The fetal material collection methods described in 1394 of the samples were: manual intrauterine aspiration (AMIU) (n=553 39.7%), curettage (n=477 34.2%), natural miscarriage (n=317 22.7%), biopsy (n=23 1.6%), aspiration (n=10 0.7%), hysteroscopy (n=7 0.5%), and combination of two or more of these methods (n=7 0.5%). A significant association (*p*<0.001) was detected between the variables method of collecting fetal material, AMIU and natural miscarriage, and MCC. The AMIU method represented 2.5 times more chances of no contamination of fetal material compared to natural miscarriage (IC 95%= 1.8 - 3.6).

**Conclusion:** POC analysis is essential to elucidate the causes of pregnancy loss. Our results show that some chromosomes are preferentially found in miscarriage, the PGT-A test for ART patients helps minimize the risk of miscarriage due to chromosomal abnormalities in the embryo and the methodology used in the selection of fetal sample can influence the risk of inconclusive results due to maternal contamination.

**P-98. Using Artificial Intelligence to predict gestational success**

**Bruno Araújo Mendes^1^, Mariana Nicolielo Barreto^2^, Dóris Spinosa Chéles^2^, Bruna Lourenço^2^, José Roberto Alegretti^2^, Eduardo Leme Alves Motta^2^, Marcelo Fábio Gouveia Nogueira^1^, Aline Rodrigues Lorenzon^2^, José Celso Rocha^1^**

^1^Universidade Estadual Paulista "Júlio de Mesquita Filho" - UNESP/Assis - Assis - SP - Brazil

^2^Huntington Medicina Reprodutiva - Eugin Group - São Paulo - SP - Brazil

**Objective:** To explore the use of artificial intelligence techniques, particularly deep learning, applied to morphological variables extracted by digital processing of blastocyst images, to predict gestational success more efficiently.

**Methods:** The study used a database containing 745 images of blastocysts, obtained using the EmbryoScope^®^ (Vitrolife) equipped with the time lapse system, from a partner clinic. Of these, 648 images were used to train the model and 97 for the blind test. The images underwent digital image processing software which extracted 33 mathematical variables representing the characteristics of blastocysts, focusing on the main areas of the blastocyst, such as the blastocele, internal cell mass and trophectoderm. These variables were then used as input in an Artificial Intelligence (AI) model, specifically deep learning to predict gestational success. To develop the AI model, we used a deep neural network architecture that has proven effective in other medical image analysis applications. The model was trained with the 33 variables extracted, adjusting the parameters to optimize accuracy in predicting gestational success.

**Results:** The deep learning model showed an overall accuracy of 100% in the training data, with an area under the curve (AUC) of 1.0, showing its robustness in discriminating between positive and negative cases of gestational success. In the blind test data, the model achieved an overall accuracy of 87%, with an accuracy of 85% for cases that did not result in pregnancy and 90% for cases that did result in pregnancy. The AUC of the blind test data was 0.8405 for non-pregnancy cases and 0.841 for pregnancy cases.

**Conclusion:** The results obtained indicate that the artificial intelligence technique based on deep learning can be a powerful tool to help embryologists in assisted reproduction clinics. The model's ability to predict gestational success with a high prediction rate can help in decision making and boost the success rates of assisted reproduction treatments. This innovative approach not only optimizes the embryo selection process but can also reduce the time and costs associated with treatments, benefiting both professionals and patients.

**P-99. Validation of an ultra-fast vitrification and warming protocol for oocytes using a Brazilian medium**

**Ana Luisa Menezes Campos^1^, Mariana Nicolielo Barreto^2^, Renata Erberelli^2^, Karina Martins^2^, Camila Cruz de Moraes^1^, Blenda Alves Silva^1^, Izadora Reis^2^, Gabriela Gonçalves^2^, Ana Paula dos Reis^2^, Lais de Moraes Santos^2^, Dóris Spinosa Chéles^2^, João Pedro Junqueira Caetano^1^, Mauricio Barbour Chehin^2^, José Roberto Alegretti^2^, Aline Rodrigues Lorenzon^2^**

^1^Huntington Pró-Criar - Eugin Group - Belo Horizonte - MG - Brazil

^2^Huntington Medicina Reprodutiva - Eugin Group - São Paulo - SP - Brazil

**Objective:** Oocyte vitrification is a well-established technique routinely used in *in vitro* fertilization (IVF) laboratories to preserve female germ cells. Recently, an ultra-fast vitrification protocol (UFP) for cryopreservation has been suggested, which is substantially shorter in time without compromising cell function (Gallardo *et al*., 2019; Liebermann *et al*., 2024). This could potentially enhance efficiency, accessibility, and patient outcomes. In this study, we tested the UFP using a Brazilian brand of vitrification/warming medium, comparing oocyte survival and maturation rates with the current standard protocol (SP).

**Methods:** Prospective cohort study including 109 surplus/inviable oocytes in different stages of maturation (germinal vesicle: GV, metaphase I: MI, and metaphase II: MII) from 19 patients and 7 egg donors between May-June/2024. GV, MI, and altered MII oocytes were randomly split into two groups for vitrification/warming using either the current SP or the new UFP. The INGÁMED vitrification/warming kit (Ingámed, Brazil) was used for both groups. Time of exposure is the main difference between protocols. All oocytes were loaded and frozen in liquid nitrogen with the open system Cryo-Ingá device (Ingámed, Brazil). Fifty oocytes were allocated to the SP and 59 to the UFP. Mean time between freezing and warming was 24.4±6.3 days. Morphological features, survival rate, and maturation time from GV to MI and MI to MII up to 24h after warming were annotated. Statistical tests were applied properly and a p-value of <0.05 was considered significant.

**Results:** The mean age of the women was 36.4±4.8 years. Mean number of GV, MI, and altered MII oocytes were 3.6±4.5, 1.7±1.6, and 0.8±2.8, respectively. The survival rate of all oocytes 2 hours after warming was 78% (39/50) in the SP and 93.2% (55/59) in the UFP (p=0.03). Morphological features, including shape, cytoplasm, perivitelline space (PS), and zona pellucida (ZP), were similar before freezing among the groups. Thirty-four GV oocytes in the SP and 36 in the UFP were compared. Immediately after warming, 05 GV oocytes (14.7%) degenerated in the SP and none in the UFP (p<0.0001). Two hours after warming, cytoplasmic alterations were observed only in the SP (28.6% vs. 0%, p=0.007). The GV maturation rate to MI and from MI to MII was similar between groups (52.9% vs. 83.3%, p=0.09 and 29.2% vs. 50%, p=0.31 for SP and UFP, respectively). The time to reach maturation from GV to MI and MI to MII was similar between groups (6.7±6.0h vs. 6.7±8.2h, p=0.55, and 18.2±5.2h vs. 21.4±13.0h, p=0.44 for SP and UFP, respectively). At the MI stage, 12 oocytes in the SP and 15 in the UFP were compared. Immediately after warming, 4 (33.3%) oocytes degenerated in the SP and 3 (20%) in the UFP (p=0.66). Cytoplasmic alterations were present in oocytes from the SP 2h after warming (37.5% vs. 0%, p=0.049). The maturation rate and time to maturation from MI to MII was similar between groups (41.7% vs. 40%, p=0.9 and 10.5±11.7h vs. 6.2±6.4h, p=0.45 for SP and UFP, respectively). Among altered MII oocytes, 4 in the standard and 8 in the fast protocol were compared. The survival rate was similar (75% vs. 87.5%, p=0.9) between groups.

**Conclusion:** Our results show that the ultra-fast vitrification protocol (UFP) using a specific Brazilian medium improves post-warm survival rates, while maturation rates and time to maturation were comparable between protocols. These results suggest that the UFP is a viable and efficient alternative, potentially optimizing laboratory resources, offering better patient outcomes, and reducing exposure to potentially toxic cryoprotectants. Further studies with larger sample sizes are warranted to confirm these results and explore the long-term implications of this protocol on fertilization and embryonic development.

**P-100. Associations between pregnancy in preimplantation genetic testing for aneuploidy *versus* blastocyst morphological classification cycles**

**Jane Porfírio Rocha^1^, Lorena Cristina Santos^1^, Júlia Coelho Masiero^1^, Gustavo Cardoso Borges^1^, Amanda de Amorim Araújo^1^, Mylena Naves de Castro Rocha Camarço^1^, Eduardo Camelo de Castro^1^, Vinícius Alves de Oliveira^1^, Rodopiano de Souza Florêncio^1^**

^1^Humana Medicina Reprodutiva - Goiânia - GO - Brazil

**Objective:** To evaluate whether the use of preimplantation genetic testing for aneuploidy (PGT-A) would result in a higher pregnancy rate than *in vitro* fertilization (IVF) using the intracytoplasmic sperm injection (ICSI) technique.

**Methods:** This is a retrospective observational study conducted between January 2021 and December 2023. The study includes analysis of data from 346 single frozen embryo transfer (FET) cycles with good quality blastocysts (≥3BB). Exclusion criteria were cycles with transfer of two or more embryos or fresh embryo transfer and blastocysts classified <3BB. The blastocysts were screened by the PGT-A group or chosen by morphological criteria in the ICSI group. Additionally, both groups were also divided into women's age (<35 and 35-39). It was evaluated chemical pregnancy, chemical abortion, clinical pregnancy, miscarriage and pregnancy ongoing or live births rates between the groups. The chemical pregnancy was confirmed by β-hcg test (>25mIU/ml) and chemical abortion resulted in positive β-hcg test, but no gestational sac was viewed. Those patients who presented a gestational sac were considered to be clinically pregnant. Those patients with ongoing pregnancy or live births (ONG/LBR) were considered with successful outcome. Statistical analysis was performed using Fisher's Exact test with a 95% confidence interval (CI).

**Results:** A total of 73 transferred blastocysts, among women under 35 years old, were subjected to PGT-A while 83 were selected only with morphological classification. In the group of women aged between 35 and 39 years, 107 underwent PGT-A and 83 were not subjected to this evaluation. Chemical pregnancy was confirmed in 61.6% in the PGT-A group and 57.8% conventional ICSI group (*p*=0.7438), both in women under 35 years of age. In the group of women between 35 and 39 years old, chemical pregnancy was confirmed in 59.81% and 51.8%, with and without PGT-A, respectively (*p*=0.3033). The rate of chemical abortion was evaluated group under 35 years old who underwent PGT-A represented 6.66% while those who did not undergo PGT-A, 12.5% (*p*=0.4876). In the group whose age ranged from 35 to 39 years, the rate was 7.81% and 6.97% (*p*=1.00), respectively. Clinical pregnancy rates were also evaluated and totaled 57.53% among PGT-A transfers in the <35 years group and 50.6% in the no PGT-A group (*p*=0.4234). In the group aged 35 to 39 years it was 55.14% and 48.19%, respectively (*p*=0.3811). The miscarriage in these cases was 4.76% and 8.33% in the PGT-A and ICSI groups under 35 years old, respectively (*p*=0.6780), in the groups between 35-39 years old, it was 9.37% and 11.62%, respectively (*p*=0.7526). Among patients with chemical pregnancies, the rates of those with ongoing pregnancy or live births (ONG/LBR) were evaluated. In the group under 35 years of age whose embryos were evaluated by PGT-A, there was a rate of 54.79% and a rate without PGT-A of 45.78% (*p*=0.3357). Among patients aged 35-39 years, the rates were 49.53% and 42.16%, respectively (*p*=0.3790).

**Conclusion:** The basic goal of assisted reproduction is to select a high-quality embryo that, after transfer to the uterus, will result in the birth of one healthy child. PGT-A is a technology developed to track chromosomal aneuploidies that arise spontaneously and can lead to failed IVF cycles. However, its role, in cases where aneuploidy is thought to play a significant effect, remains hotly debated in clinical practice. In this study, conventional ICSI resulted in similar rates when compared with PGT-A, showing that choosing a good quality embryo based on morphological aspects is a good tool for successful IVF.

**P-101. Prediction of embryonic ploidy through Artificial Intelligence, using morphological variables**

**Vinícius Casado Moraes^1^, Mariana Nicolielo Barreto^2^, Dóris Spinosa Chéles^2^, Bruna Lourenço^2^, Eduardo Leme Alves Motta^2^, José Roberto Alegretti^2^, Marcelo Fábio Gouveia Nogueira1, Aline Rodrigues Lorenzon^2^, José Celso Rocha^1^**

^1^Universidade Estadual Paulista "Júlio de Mesquita Filho" - UNESP/Assis - Assis - SP - Brazil

^2^Huntington Medicina Reprodutiva - Eugin Group - São Paulo - SP - Brazil

**Objective:** To develop a new non-invasive approach to analyzing embryonic ploidy by creating Artificial Intelligence software based on morphological data from human blastocysts.

**Methods:** A total of 556 images of human blastocysts were analyzed, 278 for artificial intelligence (AI) learning and 278 for blind test, all obtained using EmbryoScope^®^ (Vitrolife) from a partner clinic that used the PGT-A technique to determine embryonic ploidy in each case. These images were processed using digital image processing software, which extracted 33 mathematical variables representing important points in the blastocyst, such as the internal cell mass, the trophectoderm and the blastocele. Subsequently, these variables were submitted to AI software using the deep learning technique, which aims to mimic the connections of the human brain through multiple interconnected processing layers (neurons), each with variable parameters, to provide relevant predictions for complex problems. After the continuous AI learning process, the results generated by the model were compared with the biopsies obtained using PGT-A, with the aim of evaluating the accuracy of the technique used.

**Results:** After applying the AI technique to the images of human blastocysts intended for learning, we obtained an overall accuracy of 93.5%. The same accuracy was observed for euploid and aneuploid embryos, when analyzed separately. The statistical analysis carried out shows an area under the curve (AUC) of 0.926 for the overall data and individually 0.926 for euploid embryos and 0.927 for aneuploid embryos. When we used the blind test database, AI showed an overall accuracy of 65.1%, while for euploid embryos the accuracy was 64.0% and aneuploid embryos 66.2%. The AUC analysis for euploid embryos was 0.690, while for aneuploid embryos it was 0.695.

**Conclusion:** The results obtained during the AI learning stage showed good accuracy. However, when testing the model with blind test data, there was a drop in accuracy, indicating that the proposed model still needs to be improved. For this improvement, we believe that adding images to the database and including morphokinetic variables, such as cell cleavage times and detailed patient information, such as age, history of previous implantation attempts and other clinical characteristics, could significantly increase the accuracy of AI. This information can provide a more comprehensive and detailed context of embryonic development, enabling the AI technique to provide a more assertive prediction of embryonic ploidy. In this way, we will be able to make effective use of the technique in assisted reproduction clinics in the near future.

**P-102. Determination of the main morphological and morphokinetic variables derived from Artificial Intelligence techniques for predicting gestational success**

**Matheus Búbola Duarte^1^, Mariana Nicolielo Barreto^2^, Dóris Spinosa Chéles^2^, Bruna Lourenço^2^, Eduardo Leme Alves Motta^2^, José Roberto Alegretti^2^, Aline Rodrigues Lorenzon^2^, Marcelo Fábio Gouveia Nogueira^1^, José Celso Rocha^1^**

^1^Universidade Estadual Paulista "Júlio de Mesquita Filho" - UNESP/Assis - Assis - SP - Brazil

^2^Huntington Medicina Reprodutiva - Eugin Group - São Paulo - SP - Brazil

**Objective:** With the application Artificial Intelligence techniques to embryonic morphological and morphokinetic data, the aim is to determine the most influential variables in predicting gestational success. This will be done using methodologies such as connected weights, Garson's algorithm and the Light Gradient-Boosting Machine.

**Methods:** Using a database of 648 morphological data records, containing 33 variables related to trophectoderm, inner cell mass and blastocele for training and 97 for blind test, as well as 679 embryonic morphokinetic data records, with 15 variables related to embryonic division times, and 102 for blind test, all from the EmbryoScope® (Vitrolife) incubator, Artificial Intelligence models associated with genetic algorithms were developed to predict gestational success. After the training, testing and validation stages and with the models having achieved hit rates of over 60% for the blind test, the most influential variables were determined using connected weights methodologies, Garson's algorithm and the Light Gradient-Boosting Machine.

**Results:** For morphology, the methods applied indicated that the variables related to the darkest region of the blastocele, in our study the internal cell mass and its homogeneity, are the main influencers in predicting gestational success. In the case of morphokinetics, the main variables identified were CC2=(t3-t2), RelCC2= ((t3-t2))/((t5-t2)) x100 and tb-tsb. In both cases, a machine learning model based on Random Forest was used to achieve convergence in the values of the most important variables for predicting gestational success. In addition, the Recursive Variable Elimination technique was used to remove those that contributed little to the prediction, focusing on the most relevant.

**Conclusion:** The techniques used showed agreement in relation to two morphological variables and three morphokinetic variables. Although it was possible to identify the main variables, there was no agreement between the methods when it came to selecting the other variables from the 33 morphological and 15 morphokinetic variables. This disagreement can be attributed to the number of variables and the fact that each technique uses different parameters to analyze them. Regarding morphokinetic variables, studies by other researchers corroborate the agreement between the main variables, especially in relation to the CC2 variable, which several authors have highlighted as the most significant in predicting gestational success. As for the morphological variables, research using image processing is still scarce. This study could contribute to the application of more optimized artificial intelligence, using input variables determined by the algorithms studied, and thus lead to greater success in gestational prediction, making it a more effective tool for helping embryologists make clinical decisions.

**P-103. Genetic variants related to senescence associated secretory phenotype genes and their potential relationship with recurrent pregnancy losses**

**Eduarda Nabinger^1^, Luiza Pretto^1^, Bruna Duarte Rengel^1^, Thayne Woycinck Kowalski^1^, Maria Teresa Vieira Sanseverino^1^, Fernanda Sales Luiz Vianna^1^, Lucas Rosa Fraga^1^**

^1^Universidade Federal do Rio Grande do Sul - Porto Alegre - RS - Brazil

**Objective:** Recurrent pregnancy loss (RPL) is defined as two or more spontaneous pregnancy losses. Many factors are associated with higher risk for RPL; however, in about half of the cases the etiology remains unknown. Cellular senescence (CS) is a state of cell cycle arrest in which the cell maintains its viability and expresses a characteristic phenotype, known as Senescence-Associated Secretory Phenotype (SASP). SASP factors are composed by molecules related with physiological and pathological conditions and include, for example, TNF-α and IFN-γ cytokines. These molecules are related to many gestational roles, such as embryonic development and term labor, while an imbalance in TNF-α and IFN-γ production is associated with adverse reproductive outcomes. Knowing the roles of these molecules during pregnancy, the aim of this study was to evaluate the relationship between RPL and SASP focusing on TNF-α and IFN-γ through bioinformatics approaches and genetic analyses.

**Methods:** Using the databases Human Phenotype Ontology (HPO), Online Mendelian Inheritance in Man (OMiM), Comparative Toxicogenomics Database (CTD), Kyoto Encyclopedia of Genes and Genomes (KEGG) and Pubmed, lists of genes and proteins involved with RPL and SASP were generated. Those lists were compared through a Venn diagram and analyzed through STRING v. 11.5 software. A protein-protein interaction (PPI) network was created and analyzed through Cytoscape v. 3.9.1 and R Studio v. 4.2.0 softwares, aiming to visualize and understand the interaction between proteins encompassed within the network. Next, a genetic analysis of TNF rs361525 and IFNG rs2430561 polymorphism was performed in a sample of 116 women with RPL and 120 controls to evaluate the potential of these variants as risk factors for RPL.

**Results:** Our bioinformatics data evidenced 429 proteins and genes related with RPL and 99 associated with SASP, being 27 in common between both processes. Among these, TNF-α and IFN-γ were included. The network analysis showed that both processes share common markers and their networks interact through several nodes. However, the genetic analysis did not show differences in allelic and genotypic distributions between the two groups.

**Conclusion:** Our study evidenced the interaction between RPL and SASP, and TNF-α and IFN-γ functions within the processes. Further studies need to be carried out to deepen the knowledge of SASP genes as possibly involved with RPL.

**P-104. Detection of pathogenic microorganisms in the endometrium of infertile women undergoing Assisted Reproduction**

**Larissa Alves Honorato Ferreira^1^, Samara Fontes de Lima Gomes Medeiros^1^, Mychelle de Medeiros Garcia Torres^1^, Daniel Carlos Ferreira Lanza^1^**

^1^Federal University of Rio Grande do Norte - Natal - RN - Brazil

**Objective:** To investigate the occurrence of pathogenic microorganisms in the endometrium of infertile women undergoing assisted reproductive procedures.

**Methods:** This study was approved by the research ethics committee. Endometrial samples were collected from nine women undergoing assisted reproductive treatments, following the exclusion criteria: (1) hormonal alterations, and (2) altered gynecological physical and cytological examination. Samples were collected using a Pipelle and stored at -80°C until nucleic acid extraction. DNA extraction was performed using the PureLink Genomic DNA Mini Kit (Invitrogen), adapted according to the manufacturer's instructions. Samples were quantified using a NanoVue Plus spectrophotometer (GE Healthcare, UK), determining DNA concentration in ng/µL. Purity was assessed by the ratio of DNA absorbance (260 nm) to protein absorbance (280 nm). Molecular identification was performed using conventional Polymerase Chain Reaction (PCR), assessing: (1) actin gene; (2) Human Papillomavirus (HPV) using established primers MY09/MY11 and GP5+/GP6+ and specific primers for HPV types 16 and 18 by Nested-PCR; (3) 18S rRNA gene of the Apicomplexa phylum by Nested-PCR; (4) 16S rRNA gene. Positive controls were used in all PCR experiments: (1) previously amplified DNA sample, (2) HeLa and SiHa cell culture DNA, (3) Toxoplasma DNA sample, and (4) Escherichia coli cell culture DNA sample. All reactions were revealed by 1.5% agarose gel electrophoresis stained with ethidium bromide.

**Results:** The mean concentration of DNA extracted from the nine samples was 27.944±18.5ng/µL, with an average 260/280 absorbance ratio of 1.812±0.042. The actin gene was successfully amplified in 100% of the samples. Nested-PCR with primers for HPV16 and HPV18 identified positivity in two and four samples, respectively. Interestingly, positive reactions for Apicomplexa protozoa were identified in all samples. Detection of bacterial occurrence using the 16S gene did not yield clear results, showing many non-specific bands.

**Conclusion:** This study expands the knowledge of pathogen occurrence in the endometrium beyond bacteria, providing evidence of viral and protozoan presence. Reaction conditions, particularly for the detection of 18S and 16S, need optimization for reproducible results. Sample collection procedures should also be standardized, as they can introduce contamination during passage through the cervix, and the presence of mucus in the sample also presents challenges requiring attention in future studies. Our preliminary study offers new perspectives for understanding endometrial infections and their impact on fertility.

**P-105. High Body Max Index is correlated with increased rate of oocytes with fractured zona pellucida during ovarian aspiration**

**Bruna Campos Galgaro^1^, Luiza da Silva Rodrigues^1^, João Sabino Lahorgue Cunha Filho^1^**

^1^Centro de Reprodução Humana Insemine - Porto Alegre - RS - Brazil

**Objective:** To evaluate potential factors related to the occurrence of fractured zona pellucida oocytes.

**Methods:** This retrospective study included patients undergoing fresh *in vitro* fertilization treatment or oocyte cryopreservation procedure in a single center between January 2023 to April 2024. Ovarian aspiration was performed with a suction bomb (WTA-BV02) with pressure set at 200-185mmHg. Age, AMH (ng/mL), BMI and number of oocytes retrieved were assessed. Data was analyzed using JASP software and statistical analysis was performed by paired test and Pearson's correlation. Values were expressed as mean±SD and significance was accepted when *p*<0.05.

**Results:** One hundred thirty-nine patients were included in the analysis. The overall fractured zona pellucida oocyte rate was 7.2%. From the total number of patients (n=139), 33% (n=46) had 5% or more of fractured zona oocytes. Patients with ≥5% fractured oocytes had a higher number of retrieved oocytes compared with patients with less than 5% of fractured oocytes (10.09±6.63 vs. 7.44±6.14, *p*=0.02), without difference in age (35.28±4.58 *vs*. 36.26±4.09, *p*=0.21), number of follicles (12.85±7.38 *vs*. 10.39±7.29, *p*=0.06), AMH (2.70±2.26 *vs*. 2.66±3.25, *p*=0.94) and BMI (26.88±5.48 *vs*. 25.83±5.44, *p*=0.29). However, when analyzing all patients (<5% and ≥5% of fractured zona pellucida oocytes), there was a positive correlation between BMI and the number of fractured oocytes (*p*=0.004, r=0.244).

**Conclusion:** Patients with higher BMI have increased number of fractured zona pellucida oocytes. Additionally, the subset of patients with ≥5% fractured oocytes have a higher number of retrieved oocytes.

**P-106. Exploring the altered expression of endometrial marker CD163: Its potential use beyond endometriosis diagnosis**

**Luana Teixeira Souza Rodrigues^1^, Silvia Morales Jau^2^, Marina Silva Rodrigues^3^, Marcelo Lucchesi Montenegro^2^, Camila Silveira Souza2, Bernardo Rodrigues Lamourier Moura^1^, Edson Guimarães Lo Turco^1^, Fernando Prado Ferreira^2^**

^1^Urology Department, Human Reproduction Section, São Paulo Federal University - São Paulo - SP - Brazil

^2^Neo Vita Clinic - São Paulo - SP - Brazil

^3^Gynecology Department, São Paulo Federal University - São Paulo - SP - Brazil

**Objective:** To evaluate the relationship between traditional markers for endometritis and uterine receptivity with endometrium from patients with uterine disfunction and history of repeat miscarriage or implantation failure.

**Methods:** In a retrospective cohort study, we evaluated 141 women submitted to endometrial biopsy divided into: repeat miscarriage group (n=24), implantation failure group (n=19), uterine disfunction group (n=86), and control group (n=12). We measured the markers DC, CD 138, CD 163, CD 16, NKCD 16, CD 56, cytotoxic NK, PGP 9.5, E2, and P4 receptors, as well as the baseline levels of E2 and P4. The immunoassay test was used to detect endometrial markers and electrochemiluminescence to determine serum E2 and P4 concentrations. Statistical analysis was done using Bonferroni test.

**Results:** We observed that the endometrium of women with uterine disfunction and history of implantation failure had higher levels in the expression of CD163 when compared to the women with history of repeat miscarriage (6.75±3.48 x 4.39±2.14, *p*=0.059 and 7.71±4.84 x 4.39±2.14, *p*=0.043, respectively). Other markers analyzed showed no significant statistical differences.

**Conclusion:** Our findings suggest that the endometrial marker CD163, when analyzed with a different approach and proper validation, can contribute as another tool in the diagnosis of uterine dysfunctions and the possibility of implantation failure. The higher levels of this marker in endometrial samples from women with uterine dysfunction and a history of implantation failure may explain the difficulties in implantation and decreased fertility associated with these conditions.

**P-106. Exploring the altered expression of endometrial marker CD163: Its potential use beyond endometriosis diagnosis**

**Luana Teixeira Souza Rodrigues^1^, Silvia Morales Jau^2^, Marina Silva Rodrigues^3^, Marcelo Lucchesi Montenegro^2^, Camila Silveira Souza^2^, Bernardo Rodrigues Lamourier Moura^1^, Edson Guimarães Lo Turco^1^, Fernando Prado Ferreira^2^**

^1^Urology Department, Human Reproduction Section, São Paulo Federal University - São Paulo - SP - Brazil

^2^Neo Vita Clinic - São Paulo - SP - Brazil

^3^Gynecology Department, São Paulo Federal University - São Paulo - SP - Brazil

**Objective:** To evaluate the relationship between traditional markers for endometritis and uterine receptivity with endometrium from patients with uterine disfunction and history of repeat miscarriage or implantation failure.

**Methods:** In a retrospective cohort study, we evaluated 141 women submitted to endometrial biopsy divided into: repeat miscarriage group (n=24), implantation failure group (n=19), uterine disfunction group (n=86), and control group (n=12). We measured the markers DC, CD 138, CD 163, CD 16, NKCD 16, CD 56, cytotoxic NK, PGP 9.5, E2, and P4 receptors, as well as the baseline levels of E2 and P4. The immunoassay test was used to detect endometrial markers and electrochemiluminescence to determine serum E2 and P4 concentrations. Statistical analysis was done using Bonferroni test.

**Results:** We observed that the endometrium of women with uterine disfunction and history of implantation failure had higher levels in the expression of CD163 when compared to the women with history of repeat miscarriage (6.75±3.48 x 4.39±2.14, *p*=0.059 and 7.71±4.84 x 4.39±2.14, *p*=0.043, respectively). Other markers analyzed showed no significant statistical differences.

**Conclusion:** Our findings suggest that the endometrial marker CD163, when analyzed with a different approach and proper validation, can contribute as another tool in the diagnosis of uterine dysfunctions and the possibility of implantation failure. The higher levels of this marker in endometrial samples from women with uterine dysfunction and a history of implantation failure may explain the difficulties in implantation and decreased fertility associated with these conditions.

**P-107. Exploring the altered expression of PGP 9.5: The bad responder endometrial marker**

**Camila Silveira De Souza Silveira Souza^1^, Silvia Morales Jau^1^, Marina Silva Rodrigues^2^, Marcelo Lucchesi Montenegro^1^, Luana Teixeira Souza Rodrigues^3^, Bernardo Rodrigues Lamourier Moura^3^, Edson Guimarães Lo Turco^3^, Fernando Prado Ferreira^2^**

^1^Neo Vita Clinic - São Paulo - SP - Brazil

^2^Gynecology Department, São Paulo Federal University - São Paulo - SP - Brazil

^3^Urology Department, Human Reproduction Section - São Paulo - SP - Brazil

**Objective:** To evaluate the relationship between traditional markers for endometritis PGP 9.5 and uterine receptivity with endometrium from bad responder patients.

**Methods:** In a retrospective cohort study, we evaluated 187 women submitted to endometrial biopsy divided into: endometriosis group (n=24), repeat miscarriage group (n=24), adenomyosis group (n=9), implantation failure group (n=19), functional uterine factor group (n=86), maternal age group (n=11), bad responders’ group (n=2) and a control group (n=12). The endometrial marker PGP 9.5 was measured by immunoassay test. Statistical analysis was done using the Bonferroni test.

**Results:** The PGP 9.5 appeared significantly higher in the bad responders’ group when compared to the endometriosis group (0.50±0.707 x 0.05±0.213, *p*=0.020), the repeat miscarriage group (0.50±0.707 x 0.04±024, *p*=0.018), the adenomyosis group (0.50±0.707 x 0.00±0.00, *p*=0.015), implantation failure group (0.50±0.707 x 0.06±0.243, *p*=0.033), functional uterine factor group (0.50±0.707 x 0.02±0.156, *p*=0.008), maternal age group (0.50±0.707 x 0.00±0.013, *p*=0.020), and control group (0.50±0.707 x 0.00±0.00, *p*=0.010).

**Conclusion:** Our findings suggest that the traditional endometrial marker PGP 9.5, when analyzed with a different approach and proper validation, can contribute as another tool in the diagnosis of bad responders. The higher levels of this marker in endometrial samples from women that respond badly to the IVF medications may explain the difficulties in implantation and decreased fertility associated with this condition.

**P-108. Viability of isolated murine pre-antral follicles after vitrification**

**Vera Lucia Lângaro Amaral^1^, Valentina Jorge Aimi^1^, Rafael Alonso Salvador^1^, Alfred Paul Senn^2^**

^1^UNIVALI - Itajaí - SC - Brazil

^2^UNIGE - Switzerland

**Objective:** Using neutral red as a viability marker, this study evaluated the viability of mouse preantral follicles (PAFs) after vitrification, with different pre-equilibration times in the cryoprotectant.

**Methods:** Ovarian follicles were obtained from 17 non-pregnant adult female mice of the B6D2F1 strain. The follicles were mechanically isolated using 26G needles (0.45 x 13 mm) in a GV-HEPES medium (Ingámed, Maringá, Brazil). Follicles with a visually intact membrane, no antrum, and sizes of approximately 180 µm were selected. Vitrification was carried out using a vitrification kit for oocytes and embryos (Ingámed, Maringá, Brazil). Three groups of FOPAs (C12, C14, C16) were selected (n=187) and equilibrated for 12, 14, and 16 minutes, respectively, in the cryoprotectant and vitrified. Subsequently, they were warmed and placed in microdrops of 40 µL of culture medium (Continuous Single Culture Medium, Ingámed, Maringá, Brazil) supplemented with 10% serum (Ingámed, Maringá, Brazil), 2% insulin-transferrin-selenium (ITS), and 20 µL of a solution (50µg/mL) of the dye Neutral Red (NR, Sigma Aldrich, São Paulo). The follicles were cultured for 24 hours in an incubator (5% CO2, pH 7.4, 37°C), and their viability was assessed visually using a binocular stereoscopic microscope (SZ 61, Olympus, 40X). A control group (CTR, N=66) without vitrification was evaluated in parallel. Follicle viability was quantified by the percentage of the follicle surface stained with NR, using a grid of colors (0, 25, 50, 75, and 100%). Statistical comparisons between groups were performed using ANOVA (JAMOVI, https://www.jamovi.org, Sydney, Australia). Differences were considered significant with *p*<0.05.

**Results:** The mean value±standard error of viability in the tested groups was 96.2±1.9% (CTR), 63.7±5.8% (T12), 59.0±5.5% (T14), 63.7±5.4% (T16). The CTR value was different (*p*<0.01) from the three vitrified groups, which were equal to each other (*p*>0.8).

**Conclusion:** The vitrification of FOPAs is a usable technique when using a commercial kit. Future studies are needed to prove their *in vitro* development and oocyte maturation.

**P-109. The effect of maternal age on laboratory rates in MI oocytes subjected to ICSI**

**Luiza Sonaglio^1^**

^1^Fertway - Reprodução Humana - Curitiba - PR - Brazil

**Objective:** What the clinical management should be regarding MI oocytes is still under discussion. Many laboratories choose to perform the ICSI of these oocytes along with MII ones. It is well known that oocyte competence diminishes in in older women and how blastocyst rates in ICSI with MI oocytes are lower. However, it is unclear if the clinical management of MI oocytes should differ according to maternal age. The aim of this study was to evaluate if the magnitude of the decrease of rates in the ICSI of MI oocytes are more accentuated at advanced maternal age.

**Methods:** In this retrospective study, 56 patients under 35 years (Group <35; G<35) and 53 above 35 years (Group >35; G>35) with both, MII and MI oocyte inseminated at the same time with ICSI were enrolled. Cycles with male factor were excluded. Data regarding fertilization, D3 embryo, good quality D3 embryos, blastocysts and good quality blastocysts rates were collected. Development rates were compared between groups (Group <35 and Group >35) and between data from MII and MI ICSI. Moreover, the magnitude of the difference between Group <35 and Group >35 was compared. After normality analysis, laboratory rates were subjected to Chi-square test and the magnitude of the effect to Mann-Whitney non-parametric test using Jamovi software.

**Results:** Our preliminary results showed that, as expected, performing ICSI on MII oocytes led to higher blastocyst and good quality blastocyst rates in G<35 (38.19% (139/364) and 35.16% (128/364), respectively), when compared to G>35 (23.40% (66/282) and 23.05% (65/282), respectively), with no difference in fertilization and D3 embryo rates. In MI-ICSI, however, the fertilization rate was higher in G>35 (G>35; 67.03% (61/91) and G<35 47.30% (35/74)), with no differences in D3 or blastocyst rates. Comparing the data between ICSI performed on MII or MI oocytes, both groups G<35 and G>35 showed lower fertilization rates in MI ICSI (G<35; 47.30% (35/74) and G>35; 67.03% (61/91)) when compared to MII ICSI (G<35; 87.71% (364/415) and G>35; 90.68% (282/311)). Interestingly, D3 and blastocyst rates did not differ between MII and MI ICSI in either G<35 and G>35. Regarding the magnitude of the difference in performing ICSI in MII or MI oocytes between G<35 and G>35, no statistical difference was observed in the magnitude of the effect in fertilization rate (G<35; 37.80 percentual points (p.p)±6.57 and G>35; 23.58 p.p.±7.60, *p*=0.30), D3 embryo rate (G<35; 47.91 p.p.±6.84 and G>35 27.60 p.p.±7.24, *p*=0.057), good quality D3 embryo rate (G<35; 36.64 p.p.±5.76 and G>35 22.43 p.p.±7.43, *p*=0.21), blastocysts rate (G<35; 15.68 p.p.±4.99 p.p. and G>35 2.21 p.p.±6.37 *p*=0.07) and good quality blastocyst rate (G<35; 13.95 p.p.±4.93 and G>35; 0.32 p.p.±6.08, *p*=0.06).

**Conclusion:** Our preliminary data showed an expected lower fertilization rate when performing ICSI in MI oocytes, with no alteration in the magnitude of the difference between MII and MI oocytes between patients under or above 35 years. These data suggest that, when MI-ICSI is indicated, it can be performed regardless of maternal age.

**P-110. De la Chapelle syndrome: A rare case of male infertility**

**Isabella Almeida Paes Barretto Coutinho^1^, Eduardo Yoneyama Mourthé^2^, Tomás Ribeiro Gonçalves Dias^2^, Ricardo Mello Marinho^2^, João Pedro Junqueira Caetano^2^, Hérica Cristina Mendonça^2^, Paulo Franco Taitson^3^, Erica Becker de Sousa Xavier^2^**

^1^Santa Casa de Misericórdia de Belo Horizonte - Belo Horizonte - MG - Brazil

^2^Huntington Pró-Criar - Belo Horizonte - MG - Brazil

^3^PUC Minas - Belo Horizonte - MG - Brazil

**Objective:** To report a case of a rare genetic syndrome, 46 XX testicular disorder of sex development (De la Chapelle syndrome), causing male infertility.

**Methods:** A case report of a patient presented at a private clinic of Reproductive Medicine.

**Results:** A 38-year-old male patient presented to a private clinic of Reproductive Medicine with a complaint of 2 years of infertility. Physical exam exhibited a normal male phenotype, normal external male genitalia development (Tanner stage V) with significant bilateral testicle atrophy (<1 cc), low stature, and seminal analysis indicating azoospermia. His hormone panel indicated primary hypogonadism (total testosterone of 280 ng/dl and FSH of 17 mlU/ml. Subsequent tests revealed a 46 XX karyotype. He was then diagnosed with 46 XX testicular disorder of sex development, also known as De la Chapelle syndrome, which is a rare one, occurring in 1:20,000 to 25,000 male newborns. Approximately 90% of affected individuals have a normal phenotype at birth and are typically diagnosed post-puberty due to hypogonadism, gynecomastia, and/or infertility, as observed in this patient.

**Conclusion:** This case underscores the importance of considering rare syndromes such as De la Chapelle syndrome in the differential diagnosis of phenotypically normal males presenting with complete azoospermia. It highlights the necessity for thorough genetic analysis in patients with azoospermia.

**P-111. Association between Anti-Müllerian hormone levels and the percentage of euploid blastocysts obtained in In Vitro Fertilization cycles**

**Carolina Marques Miranda Albuquerque Maranhão^1^, Pietra Paschoalino Boareto^1^, Arlene Oliveira Fernandes^1^, Ana Luisa Menezes Campos^2^, Erica Becker Sousa Xavier^2^, Ricardo Mello Marinho^2^, Joao Pedro Junqueira Caetano^2^**

^1^Faculdade de Ciências Médicas de Minas Gerais - Belo Horizonte - MG - Brazil

^2^Huntington Procriar - Belo Horizonte - MG - Brazil

**Objective:** To evaluate the relationship between anti-Müllerian hormone (AMH) levels and oocyte quality in patients undergoing *in vitro* fertilization (IVF) cycles by assessing embryonic ploidy after preimplantation genetic testing for aneuploidy (PGT-A).

**Methods:** This retrospective cohort study was conducted by collecting data from medical records of patients who underwent IVF cycles between June 2019 and April 2024. Women aged 27 to 45 years who had their embryos biopsied and AMH levels measured less than one year before the start of treatment were included. Data were collected using a standardized table that included age, AMH levels, number of oocytes retrieved, number of mature oocytes, number of blastocysts, number of top-quality (TQ) blastocysts, number of euploid embryos, and the proportion of euploid embryos (euploid embryos/analyzed embryos). Patients were divided into two groups according to AMH levels (less than 1.1 ng/mL and greater than or equal to 1.1 ng/mL, according to the Bologna criteria). The two groups were compared regarding the proportion of euploid embryos and other outcomes listed above. The sample size suggested for the study was 152 patients.

**Results:** A total of 159 patients were included in the study. The mean age of the patients was 39 years. AMH concentrations ranged from 0.15 to 11.39ng/mL, with a mean of 1.5ng/mL. It was demonstrated that AMH levels greater than 1.1ng/mL were associated with higher numbers of mature oocytes, blastocysts, and TQ blastocysts. The percentage of euploid blastocysts in the study was 37%; 30% in Group 1 (AMH less than 1.1ng/mL ) and 38% in Group 2 (AMH greater than or equal to 1.1ng/mL), with a *p*-value <0.001.

**Conclusion:** This study suggests that AMH levels above 1.1 ng/mL are associated with a better response to ovarian stimulation and better oocyte quality, as indicated by a higher percentage of euploid embryos.

**P-112. Meta-analysis of Microfluidic Sperm Sorting: Superiority in improving fertilization rates, pregnancy rates, and embryo quality in art**

**Henrique Cunha Vieira^1^, Matheus Ferreira Gröner^2^, Renata Cristina de Carvalho^2^, Amanda Lino de Faria Lessa^3,^ Barbara Bomfim Muniz Moraes^3^, Luiz Felipe Lessa Ortiz^3^, Caio Caio Bosquê Hidalgo Ribeiro^2^, Irineu Farina Neto^2^, Rodrigo Perrella^1^, Renato Fraietta^2^**

^1^Hospital Militar de Área de São Paulo (HMASP) - São Paulo - SP - Brazil

^2^Escola Paulista de Medicina (EPM/Unifesp) - São Paulo - SP - Brazil

^3^Gavva Clínica - São Paulo - SP - Brazil

**Objective:** Microfluidic sperm sorting (MSS) has emerged as a promising alternative to conventional sperm preparation techniques like density gradient centrifugation (DGC) and swim-up methods. The quality of sperm used in Assisted Reproductive Technology (ART) significantly impacts the success rates of procedures such as *in vitro* fertilization (IVF) and intracytoplasmic sperm injection (ICSI). Traditional sperm preparation methods often fail to adequately reduce sperm DNA fragmentation, a critical factor affecting fertility outcomes. This study reviews the effectiveness of MSS in enhancing fertilization rates, pregnancy rates, and embryo quality by selecting sperm with higher DNA integrity and motility.

**Methods:** A comprehensive literature search was conducted on PubMed for studies published between 2014 and 2024 using MeSH descriptors "microfluidics" and "Sperm DNA fragmentation”. This meta-analysis evaluates the efficacy of MSS in improving fertilization rates, pregnancy rates, and embryo quality in assisted reproductive technology (ART) in patients with sperm DNA fragmentation (SDF). Seven of twelve studies were relevant and included in this meta-analysis. Statistical measures such as mean improvement, standard deviation (SD), standard error (SE), degrees of freedom (df), confidence interval (CI), t-statistic, and p-value were calculated for the key outcomes.

**Results:** The analysis demonstrates that MSS offers significant advantages over conventional sperm preparation techniques. The analysis for fertilization included data from seven studies: Avendaño *et al*., Alomar *et al*., Jayaraman *et al*., Nosrati *et al*., Pinto *et al*., Zheng *et al*., and Smith *et al*.. The mean improvement in fertilization rates using MSS was 14.29%, with a 95% CI of 11.12% to 17.45% (*p*<0.001). The degrees of freedom (DF) for this analysis was 6. The t-statistic was 14.38. For pregnancy rates, data from five studies were included: Avendaño *et al*., Alomar *et al*., Pinto *et al*., Zheng *et al*., and Smith *et al*. Pregnancy rates showed a mean improvement of 13.17%, with a 95% CI of 10.65% to 15.69% (*p*<0.001). The DF for this analysis was 5. The t-statistic was 16.62. Data from four studies were analyzed for embryo quality: Alomar *et al*., Jayaraman *et al*., Zheng *et al*., and Smith *et al*. Embryo quality also improved significantly, with a mean improvement of 19.00% and a 95% CI of 17.58% to 20.42% (*p*<0.001). The DF was 4. The t-statistic was 42.49. These findings are clinically meaningful and support the integration of MSS into clinical practice to optimize ART outcomes when the patient has known high SDF. The high t-statistics and extremely low p-values across all three outcomes underscore the robustness of these findings. The narrow confidence intervals further reinforce the reliability of the observed improvements. However, it is important to note the limitations of the included studies, such as small sample sizes and potential selection biases, which could impact the generalizability of the results. Future research should aim to include larger, multi-center trials to validate these findings and explore the long-term benefits of MSS in ART.

**Conclusion:** MSS significantly enhances fertilization rates, pregnancy rates, and embryo quality in ART. This method's ability to select sperm with higher DNA integrity and motility makes it a superior choice for traditional sperm preparation, leading to better clinical outcomes.

**P-113. Composition of seminal extracellular vesicles and their potential in Assisted Human Reproduction**

**Clara Garcia de Melo^1^, Fernanda Tanese Ubriaco de Souza^1^, Sabrina de Lima Garcia Correia^1^**

^1^Centro Universitário São Camilo - São Paulo - SP - Brazil

**Objective:** To describe the composition of the extracellular vesicles present in semen and relate their content to the probable functions for the success of assisted human reproduction.

**Methods:** The method used was a systematic review of the literature where 62 scientific articles were obtained from different databases and virtual libraries, such as: PubMed, Human Reproduction Update, GebFra Science and Science Direct, among which only 28 were included according to the inclusion criterion for presenting information that directly related seminal extracellular vesicles and assisted reproduction.

**Results:** Extracellular vesicles (EVs) are bound to the cell through the membrane and released when necessary. They are highly complex and do not allow distinguishing their subtypes and their specific biomolecular mechanism. Composed of proteins, lipids and genetic material, the molecules on its surface allow interaction with other cells through adhesion to lipids and ligands on the surface of a receptor, and fusion of the vesicular membrane with the plasma membrane of a cellular receptor can also occur. Seminal EVs fuse with the sperm membrane during ejaculation to transfer molecules that aid sperm survival, fertilization and maturation. Through an *in vitro* swine model, it was found that seminal EVs have the ability to induce and assist the acrosome reaction, in addition to enabling a possible prevention of polyspermy as they can bind to and neutralize sperm that are about to initiate an acrosome reaction, from this perspective, its use for *in vitro* fertilization could help these processes. Due to their scarce cytoplasm, spermatozoa have limited defenses against reactive oxygen species, the extracellular vesicles present in semen, such as epididymosomes, in turn are abundant in glutathione peroxidase-5 (GPX5) protein, an important antioxidant that helps in the control of reactive oxygen species and also prevents premature acrosome reaction, in addition its defense capacity is also related to the elimination of defective sperm through ubiquitination, these mechanisms could be used in favor of patients who present high oxidative stress and in sperm selection. In addition to the implications for male fertility, a study using mice isolated extracellular vesicles from seminal fluid and added these nanoparticles to the *in vitro* fertilization medium together with oocytes and sperm to evaluate their influence on the formation of blastocysts. The results were promising considering that the blastocyst formation rate increased significantly and the proportion of inner cell mass and trophectoderm was extremely positive, in addition to reducing blastocyst apoptosis rates when compared to the medium without addition of seminal EVs.

**Conclusion:** Seminal extracellular vesicles have several functions linked to sperm maturation, fertilization and possibly the formation of blastocysts, having great potential to suggest high quality male gametes through the proteins linked to their composition, acting as ideal non-invasive biomarkers to determine the sperm quality. Its potential for the development of new therapies aimed at increasing reproductive success with regard to the male factor is promising as they are essential for the maintenance of sperm functions. For *in vitro* fertilization, seminal EVs could help the formation of blastocysts, increasing the chance of success of the procedure. However, investment in research with human models and clinical trials is necessary to explore the potential of its use in assisted human reproduction considering the positive results obtained in animal models.

**P-114. Psychological attention in genetic counseling in the context of Assisted Human Reproduction**

**Flávia Giacon^1^, Camila Dutra Souza Francisquini^2^**

^1^Mater Prime Clínica de Reprodução Humana - São Paulo - SP - Brazil

^2^Conceber - Centro de Medicina Reprodutiva - Curitiba - PR - Brazil

**Objective:** This work aimed to describe psychological care in genetic counseling and understand how this psychological and genetic interface can contribute to patient decision-making during their reproductive treatments. Advances in biotechnology have made it possible, through genetic tests, to recognize individuals' conditions, enabling them to formulate diagnoses in advance and predict their risks for their occurrence or recurrence, evaluating reproductive impacts and prognosis. In addition to the difficulty of getting pregnant, the patient is faced with the possibility of their child having a genetic, congenital, hereditary, or chromosomal condition, which, in theory, changes the entire perspective of the parenting project, which could have some repercussions on the life of this family. Considering that these are exhausting treatments from the point of view of physical, emotional, and financial health and that all information is valid for understanding the scenario that presents itself, would genetic counseling be mandatory for all patients undergoing Assisted Human Reproduction techniques?

**Methods:** The method used was a brief search in the PubMed, Scielo, and Bvs-Psi databases, from the corresponding descriptors, specifically regarding psychological care in genetic counseling in the context of assisted reproduction.

**Results:** After surveying the literature, we observed that when we searched for broader descriptors correlated to psychological care in genetic counseling, few articles were found; whereas, when specifying the search regarding psychological care in genetic counseling in reproductive treatments, rare articles were published. It must be considered that all technology is favorable in terms of enabling conduct based on access to genetic information. On the other hand, there is a need for a careful and welcoming approach during this genetic counseling process especially when communicating its results to the patient. It is not just about passing on accurate information, but also promoting a welcoming and safe space for the patient so that, based on knowledge of the probabilities, they can adjust their expectations with the reality of their treatment. The professional psychologist helps the patient to think about the result, but mainly about what to do with it. A specialized interdisciplinary team can advise on the risks and benefits, assisting you in all areas, and contributing to their decision-making. May the presence of mental health professionals be a reality in genetics services in the country and, through psychoeducational and psychotherapeutic actions, be able to add efforts to these teams.

**Conclusion:** People look for human reproduction clinics, with the desire to fulfill their parental project, since after spontaneous attempts, the child did not come. Reproductive treatments are exhausting and, as the stages progress, all complexity presents itself. When genetic counseling is indicated, this referral can be experienced with anguish, uncertainty, and helplessness. These feelings may arise in patients due to fear of the unknown, as well as what may arise as a result of this counseling. Psychologists are professionals trained in creating welcoming spaces for the integration of grief and necessary resignifications, helping them to discover their internal resources, develop resilience in sensitive situations, and be able to experience them more lightly. The results of genetic tests may conflict with patients' expectations and, for this reason, endorse the need for specialized monitoring. This interface between psychology and genetics studies presents itself as a promising path for professionals, but especially for patients at all stages of treatment. By having access to specialized professionals, they will be monitored more assertively and will be able to make more consistent choices.

**P-115. Fertility awareness, knowledge and attitudes of medical students: An educational opportunity**

**Eleonora Bedin Pasqualotto^1^, Fabio Firmbach Pasqualotto^1^, Sofia Bulla Paviani^1^, Gabriela de Marco Burtet^2^, Maria Fernanda Bassani Nácul^2^, Vitoria Chen^2^, Gabriela Vieira Pontalti^2^, Domenica Clara Fistarol^2^, Lucas Faraon Gonçalves^2^**

^1^CONCEPTION / UCS - Caxias do Sul - RS - Brazil

^2^UCS - Caxias do Sul - RS - Brazil

**Objective:** Attending medical school involves a long educational path, and the start of a young doctor's professional life tends to coincide with the age when fertility rates decline. Although medical students are more likely to postpone pregnancy, it is unclear to what extent they are aware of the misfortunes that accompany this choice. The aim of the present study is to evaluate the knowledge of medical students at a university in Rio Grande do Sul regarding fertility, their attitudes towards the challenge of balancing personal and professional life, and their interest in receiving reproductive education.

**Methods:** This is a cross-sectional and descriptive study, submitted to the Research Ethics Committee and approved under number 023385/2024. Data collection was conducted using an anonymous, self-administered questionnaire, divided into sections: identification, parental attitudes, and knowledge about fertility. The sample consists of 360 medical students from a college, ranging from the first to the eighth semester, aged between 18 and 43 years. After administering the questionnaire, educational material on fertility and reproductive education was provided.

**Results:** The majority of the students are women (65.4%), without children (99.4%), single (98.9%), heterosexual (91.6%), aged between 18 and 43 years, from the first to the eighth semester of the course. Most intend to have children in the future (82.7%), consider 30 years old as the best age for the first child (32.5%) and 35 years old for the last child (25.1%). Another portion (25% of the students) points to ages above 35 years (36 to 50 years) as the best age for the last child. Concern about future reproduction due to a career in medicine was mentioned by 83.2% of the students, and 88.3% are interested in learning more about fertility. The research reveals that most students prioritize the following essential factors for family planning: financial stability, a stable relationship, and the completion of academic training. Students also indicated they would freeze their gametes for fertility preservation in case of illness or delayed family planning, and in the event of infertility, 78.5% would seek assistance with assisted reproduction. In the knowledge section, students demonstrated understanding of the decline in fertility with aging and knew that infertility causes are not predominantly female. They are aware that sexually transmitted infections, excessive caffeine and alcohol consumption, smoking, and extreme body weight negatively impact fertility. Additionally, 72.3% of the students are aware of the existence of tests that quantify ovarian reserve. When questioned about assisted reproduction techniques, students had varied beliefs about the actual success rates of treatments, with 28.2% believing that 60-100% result in pregnancy. The information that cryopreserved gametes can be stored indefinitely is known by 54.2% of the students. The probability of pregnancy at the time of ovulation was varied among students, and only 39.4% correctly indicated a 16-20% chance of pregnancy; the rest of the students believed the rate ranges from 30 to 100%.

**Conclusion:** Despite demonstrating good medical knowledge on the topic, the students overestimated the chances of natural pregnancy and assisted reproduction. Additionally, they confirmed their intention to postpone pregnancy. It is positive to note the students' interest in receiving fertility education, which presents an opportunity that should be leveraged to ensure effective family planning.

**P-116. The impact of regular exercise on Reproductive Health: The future of responsible innovation**

**Vanessa Wolff Machado^1^, Jessica Lucena Wolff^1^**

^1^Genesis / ESCS - Brasília - DF - Brazil

**Objective:** Reproductive health, encompassing fertility and hormonal balance, is vital for overall well-being. While regular exercise is recognized for its health benefits, its impact on reproductive health requires further exploration. This trial investigates how regular exercise affects fertility and hormonal balance in both men and women.

**Methods:** Fifty healthy participants (25 males, 25 females), aged 25-35, were randomly assigned to an exercise group or a control group. The exercise group engaged in 150 minutes of moderate-intensity exercise per week for 12 weeks, following specific guidelines. The control group maintained their regular sedentary lifestyle. Both groups kept their normal diet and lifestyle throughout the trial. Data collection occurred at weeks 0, 6, and 12 through regular check-ups. Fertility was assessed by tracking menstrual cycles in females and sexual activity in males. Female participants monitored menstrual cycle regularity, duration, and intensity, while males recorded changes in sexual performance and libido. Key fertility parameters, such as ovulation (females) and sperm count, motility, and morphology (males), were measured at the beginning and end of the trial. Blood samples collected at weeks 0, 6, and 12 were analyzed for hormone levels, including estrogen, progesterone (females), testosterone (males), follicle-stimulating hormone (FSH), and luteinizing hormone (LH).

**Results:** The exercise group showed significant improvements in fertility for both genders. Female participants reported more regular menstrual cycles, decreased menstrual pain, and better overall menstrual health. Males experienced increased libido and enhanced sexual performance, along with higher sperm count, motility, and morphology. Hormonal analysis revealed notable changes: both genders in the exercise group had decreased estrogen levels, indicating improved hormonal balance. Females also had increased progesterone levels, crucial for fertility, while males exhibited higher testosterone levels, enhancing sexual health and reproductive function. FSH and LH levels remained stable, indicating a well-maintained hormonal equilibrium.

**Conclusion:** Regular exercise positively impacts reproductive health. For females, it improves menstrual regularity, reduces pain, and enhances overall menstrual health. For males, it increases libido, improves sperm quality, and balances hormones. The decrease in estrogen and increase in progesterone and testosterone levels create a favorable environment for conception and reproductive health. These results align with previous studies highlighting exercise's benefits on reproductive health, such as improved blood circulation, enhanced endocrine function, reduced stress, and maintained healthy body weight. This trial suggests that exercise can improve fertility, enhance hormonal balance, and contribute to overall well-being. While further research with larger sample sizes and longer durations is needed to validate these findings, promoting regular exercise as part of a healthy lifestyle may benefit those aiming to optimize their reproductive health. This work was entirely generated by artificial intelligence as a playful effort by the authors to draw attention to the future of ethical and responsible scientific innovation. Initially, a text was generated using the Perplexity AI platform, and we removed the word "hypothetical" from the text. Subsequently, we used the Chat GPT platform to summarize the work to 600 words.

**P-117. Measurement of Anti-Müllerian hormone in capillary blood as a predictor of ovarian response in Assisted Reproduction cycles**

**Leci Veiga Caetano Amorim^1^, Maria Regina Moreira Santos Aquino^1^, Luara Isabela dos Santos^2^, Paula Fernandes Távora^2^, Erica Becker Sousa Xavier^1^, Ricardo Mello Marinho^1^, João Pedro Junqueira Caetano^1^**

^1^Huntington Procriar - Belo Horizonte - MG - Brazil

^2^Faculdade de Ciências Médicas de Minas Gerais - Belo Horizonte - MG - Brazil

**Objective:** To correlate anti-Müllerian hormone (AMH) values measured in capillary blood from dried blood spots with ovarian response in assisted reproduction cycles, specifically focusing on the number of retrieved oocytes, mature oocytes, and blastocyst formation.

**Methods:** A retrospective analysis was conducted on *In Vitro* Fertilization (IVF) and elective oocyte cryopreservation cycles at a private clinic between March 2023 and March 2024. Included were cases where AMH levels were measured in capillary blood within 30 days prior to stimulation.

**Results:** Forty-six cycles were analyzed, with a mean patient age of 36 years. Thirty-nine cycles involved IVF, and seven were elective oocyte cryopreservation. AMH concentrations ranged from 0.24 to 8.81ng/mL (mean=2.5ng/mL). Pearson's coefficient analysis demonstrated a strong association between AMH concentrations and antral follicle count, and a moderate association with the number of retrieved oocytes, mature oocytes (MII), fertilized oocytes, and good-quality embryos on day 3. A weak association was observed with the number of blastocysts. Upon dividing the sample into two groups (AMH <1.1ng/mL and ≥1.1 ng/mL), higher AMH levels statistically correlated with increased antral follicles and a higher number of MII oocytes, but not with number of blastocysts.

**Conclusion:** Capillary blood measurement of AMH offers a straightforward and immediate method, showing strong correlations with antral follicle count and response to ovulation induction in assisted reproduction cycles, particularly in terms of mature oocyte yield. However, AMH levels did not correlate with blastocyst formation in this study. Further investigations with larger sample sizes and comprehensive variable analyses are needed to confirm and extend these findings.

**P-118. Serum progesterone measurement on the day of fresh embryo transfer and correlation with pregnancy success rate: A prospective analysis**

**Paula Andrea Albuquerque Salles Navarro^1^, Carla Maria Franco Dias^1^, Suelen Maria Parizotto Furlan^1^, Rui Alberto Ferriani^1^**

^1^Hospital das Clínicas de Ribeirão Preto da Universidade de São Paulo (HC FMRP USP) - Ribeirão Preto - SP - Brazil

**Objective:** Studies suggest that low serum progesterone (P4) on the day of embryo transfer (ET) are related to worse gestational outcomes. However, studies are still inconclusive and insufficient regarding the existence of a P4 concentration correlated with higher clinical pregnancy rates (CPR) in fresh ET after controlled ovarian stimulation (COS) cycles. In the present study, we aimed to find a cutoff point of P4, measured on the day of fresh ET, that correlates with higher CPR, and identify potential factors related to P4 levels and CPR.

**Methods:** Prospective cohort study carried out at in a public IVF Center. All patients who underwent controlled ovarian stimulation (COS) for fresh ET from August 2021 to October 2021, aging < 45 years old and with body mass index (BMI) < 35 kg/m^2^, were considered eligible. Luteal phase support was carried out with vaginal micronized progesterone (200 mg, 8/8 h), beginning on the day of oocyte retrieval. The primary outcome was CPR beyond the 8th week of pregnancy. P4 measurement was performed from a blood sample drawn between 9 to 11 am on the ET day. A ROC curve was constructed to detect the best cutoff point correlated to higher CPR. Patients were categorized according to serum P4 dosage, being divided into patients with serum levels above and below the cutoff point. Finally, multivariate logistic regression was performed to verify which exploratory variables were predictors of gestational outcomes.

**Results:** 106 patients were evaluated. P4 measured on the fresh ET day showed no significant difference between patients who got pregnant and those who did not (67.12±31.1ng/mL versus 64.17±61.76, *p*=0.7465). The cutoff point correlated with higher CPR was P4 ≥28.855 ng/mL (AUC 0.5654; sensitivity 1.0; specificity 0.158), with significant statistical association (*p*=0.0208). However, since there were no patients who did not become pregnant with P4≥28.855 ng/mL, it was not possible to calculate the odds ratio. The variables woman age (OR 0.878; CI 0.774 - 0.995; *p*=0.0386) and the presence of top-quality embryo transferred (OR 2.89; CI 1.148 - 7.316; *p*=0.0214) were associated with the CPR. Woman age ≥40 years (OR 0.0956; CI 0.0156 - 0.5851; *p*=0.0007), poor response to COS (OR 0.0964; CI 0.0155 - 0.5966; *p*=0.0014) and number of follicles ≥10mm (OR 1.465; CI 1.013-2.117; *p*=0.0013) were associated with P4 cutoff point.

**Conclusion:** P4≥28.855ng/mL on the fresh ET day showed a statistical correlation with CPR. However, the ROC curve constructed was considered unsatisfactory, and it was impossible to calculate the odds ratio to confirm our findings in a multivariate analysis. Therefore, this cutoff point should not be used to infer gestational success. In our study, it was possible to correlate P4 dosage and CPR with female age. Therefore, P4 measurement on the day of fresh ET seems to be more a reflection of good gestational prognostic factors, such as younger female age, good ovarian reserve, and good response to COS, rather than actually being a good predictor of clinical pregnancy.

**P-119. The hyaluronan-rich transfer medium is correlated with outcomes in frozen-thawed embryo transfer**

**Samara Artuso Giacomin^1^, Camila Dutra Souza Francisquini^1^, Susan Kelly Coelho Martins Verillo^1^, Alessandro Schuffner^1^**

^1^Conceber Centro de Medicina Reprodutiva - Curitiba - PR - Brazil

**Objective:** The objective of this study was to determine whether the use of hyaluronan-rich transfer medium influences outcomes in thawed blastocyst transfer cycles.

**Methods:** Retrospective observational study evaluating 115 embryo transfers from January 2023 to May 2024. Inclusion criteria: transfers of frozen-thawed embryos, at blastocyst stage, on day 5, 6, or 7 of development and regardless of morphological classification (by Gardner Grade), embryos cryopreserved by the vitrification method, with or without chromosomal status analysis, with transfer of 1 or 2 embryos. Exclusion criteria: endometrial thickness <7.0mm and progesterone at the last ultrasound - 5 days before transfer ≥1.5ng/mL. The transfers were divided into 2 study groups: Group 1 - with hyaluronan medium (n=23 transfers) and Group 2 - medium without hyaluronan (n=92 transfers). The embryos were thawed 2 to 3 hours before embryo transfer following the Irvine^®^ Thawing Kit medium protocol and then transferred to the culture plate with CSCM-C^®^ medium, pre-equilibrated overnight in a tri-gas incubator to pH:7 .27 to 7.29. Subsequently, the embryos from group 1 were transferred to the transfer plate with hyaluronan medium (EmbryoGlue^®^) and pre-equilibrated overnight in a tri-gas incubator to pH: 7.27 to 7.30. The embryos stayed in EmbryoGlue^®^ for a minimum of 13 minutes and a maximum of 70 minutes (average time 37 minutes) according to the recommended protocol (between 10 to 240 minutes before embryo transfer). Group 2 embryos were placed on the transfer plate containing CSCM-C^®^ culture medium at the time of transfer. For both groups, the same transfer catheter was used and the embryos were loaded onto the catheter using the same protocol. We compared the rate of positive β-HCG/transfer after 9 days of embryo transfer (β-HCG≥50mIU/mL) and the rate of clinical pregnancy/transfer (presence of 1 or 2 gestational sacs with the presence of fetal heartbeat at 8 weeks of pregnancy) between the groups using the Z test for 2 proportions, adopting the statistical *p*<0.05.

**Results:** To confirm the groups homogeneity was compared maternal age (5% ANOVA test), and the proportion of embryos transferred with and without chromosomal status analysis (Test-Z for two proportions at *p*<0.05), and no statistically significant difference was observed between them (*p*>0.05). For group 1, the mean of maternal age was 36.9±2.9 years, and for group 2 was 36.6±3.2 years. Regarding the proportion of embryos transferred with or without genetic analysis, for group 1, 69.6% (15/23) of the transferred embryos were genetically analyzed, while for group 2, 75.0% (69/92) of the embryos were analyzed for chromosomal status. The rate of positive β-HCG/embryo transfer in group 1 (with hyaluronan medium) was 78.3% (18/23) while in group 2 (without hyaluronan medium) the rate was 54.4% (50/92), showing a significant difference between the groups (*p*<0.05). When comparing the rate of clinical pregnancy/embryo transfer, group 1 presented a rate of 78.3% (18/23), that is, all transfers that presented implantation resulted in clinical pregnancy. While, in group 2, the clinical pregnancy/transfer rate was 51.1% (47/92), with group 1 having a higher clinical pregnancy/transfer rate when compared to group 2 (*p*<0.05).

**Conclusion:** Based on the results presented, it was possible to infer that the hyaluronan-rich transfer medium may be associated with an increase in the rates of positive β-HCG and clinical pregnancy in frozen-thawed embryo transfer cycles.

**P-120. Desire for parenthood and information seeking regarding fertility and reproductive health in Brazilian transgender people**

**Vanya Sansivieri Dossi^1^, Fabiana Campos^1^**

^1^Clínica Particular - São Paulo - SP - Brazil

**Objective:** To evaluate the desire for parenthood among Brazilian transgenders and their access to information regarding fertility and reproductive health. Justification: Few studies have been made in Brazil to verify the desire for parenthood in transgender people. Gender transition, from a biological point of view, involves a series of medical and therapeutic interventions, that are aimed at aligning the physical body with the gender identity. This may include hormonal treatments and gender-affirming surgeries, and each of them has its own effects and specific biological processes. Therefore, international studies indicate that, for ethical reasons, advice on gamete preservation should take place before the pubertal block or the use of hormones for gender affirmation, given the risks that these procedures may pose to fertility (Warton & McDougall, 2022) Fertility is also a concern addressed by the World Professional Association for Transgender Health (WPATH). Since 2012, WPATH’s manual recommends consulting specialists in human reproduction, who are required to discuss all current possibilities for fertility preservation, as well as experimental studies. With this regard, more studies about information seeking and the desire for parenthood in people with gender variability are important, not only to clarify the possible impacts on reproductive potential, promote health and well-being, but also to assess the demand and prepare the professionals, who care for transgender people, to consider the particularities of this population, especially at human assisted reproduction clinics.

**Methods:** Data research of international studies published in PUBMED during the last 5 years about the desire for parenthood and fertility of transgenders. Online survey, via Google Forms, with objective and discursive questions, to evaluate the trans population's access to human reproduction centers and their desire for parenthood.

**Results:** international studies showed that transgenders receive little information about fertility and reproductive health. Regarding transgender’s desire for parenthood studies such as Chen *et al*. (2018) and Alpern *et al*. (2022) found that most participants (60%) want to have children and would like to receive information about fertility and parenthood. Both studies indicated that most young people (66%) had not discussed or reflected on the effects of hormones and their impact on fertility, some considered assisted reproductive techniques (less than 40%), and only a minority (6%) preserved fertility. Regarding to the authors' experience in listening to adolescents with gender variability and their families, most questions emerge about sexuality, affective relationships, pubertal blockage and hormone therapy. Questions about parenting or fertility preservation are infrequent and tend to remain in the background since most fear developing secondary characters and want avoid dysphoria. However, comparing the surveys of 2018 and 2021, there is a significant increase in the demand from the trans population in general for assisted reproduction centers. Furthermore, the number of clinics caring for this population also increased from 45% to 67%, with the greatest demand being for transfeminine people (55%).

**Conclusion:** There is a need for more information related to the reproductive health of Brazilian trans, especially adolescents. Targeted studies may enable the promotion of comprehensive health, expanding access to more assertive information. Research in the area favors the preparation of professionals who care for trans people, especially teams from human assisted reproduction services, so they can understand how to provide better care for this population. Considering all the interventions that bodies in transition are subjected to, this knowledge is essential to ensure careful reception for these people and to validate their desire for parenthood.

**P-121. Too cool to be late: The discrepancy between the required and obtained oocyte number in a social fertility preservation program**

**Daniela Paes Almeida Ferreira Braga^1^, Amanda Souza Setti^1^, Maite Del Collado^2^, Edward Carrilho^3^, Assumpto Iaconelli Junior^4^, Lucas Yamakami^5^, Isaac Yadid^6^, Edson Borges Jr.^4^**

^1^Fertility Medical Group, and Sapientiae Institute - Sao Paulo - SP - Brazil

^2^Science Creating Lives - São Paulo - SP - Brazil

^3^Fertility Medical Group / FERTGROUP Medicina Reprodutiva - São Paulo - SP - Brazil

^4^Fertility Medical Group / FERTGROUP Medicina Reprodutiva, and Sapientiae Institute - São Paulo - SP - Brazil

^5^VidaBemVinda / FERTGROUP Medicina Reprodutiva - São Paulo - SP - Brazil

^6^Primordia / FERTGROUP Medicina Reprodutiva - Rio de Janeiro - RJ - Brazil

**Objective:** Social fertility preservation empowers women to safeguard their fertility by anticipating age-related decline and diminished efficacy of fertility treatments in older ages. The effectiveness of oocyte vitrification programs, and consequently fertility preservation, depends on the patient's age. Previous reports on fertility preservation indicate that, typically, a larger number of oocytes are retrieved in patients aged ≤35 when compared to older patients. Although these reports document that oocyte vitrification preserves developmental potential akin to fresh oocytes, uncertainties persist regarding patient awareness of the adequate number of cryopreserved oocytes needed for pregnancy. The goal for the present study was to evaluate: (i) the outcomes of thawed oocytes from a social fertility preservation (SFP) program by age and (ii) whether the number of cryopreserved oocytes is sufficient to achieve pregnancy.

**Methods:** This historical cohort study, conducted at a university-affiliated IVF center from January 2012 to December 2022, analyzed data from 1,332 vitrified oocyte ICSI cycles involving 1,000 SFP program participants. Fertility preservation patients and oocyte thawing cycles (n=132) were categorized by age as follows: <30 years (n=55 and 24), 30-35 years (n=250 and 33), 36-40 years (n=480 and n=42), and >40 years (n=215 and 33) for preservation and thawing, respectively. Laboratory and clinical outcomes were compared among the groups. In a subsequent analysis, data from PGT cycles conducted in our laboratory (n=480) from January to December 2022 were utilized to determine blastulation and euploidy rates within vitrified oocytes. This data, combined with literature findings, was employed to estimate the average number of oocytes required for a patient to reach a 95% cumulative probability of pregnancy. These estimates were then compared with our database findings.

**Results:** There were 1,332 cycles of SFP involving 1,000 patients. Among them, 121 (12.2%) returned for oocyte thawing and underwent 132 ICSI cycles. A significant difference in the survival rate was observed among thawed oocytes from patients under 30 compared to those over 40 years-old (<30: 90.7±33, 30-35: 81.4±40.4, 36-40: 83.4±35.5, and >40: 72.3±34, *p*=0.047). Additionally, a significantly higher pregnancy rate was observed in the 30-35 age group when compared to those in the group aged >40 years-old (<30: 50.0±17.7, 30-35: 56.0±85.0, 36-40: 46.3±69.0, and >40: 21.4±93.0, *p*=0.048). However, there was no significant difference when comparing fertilization, blastulation, and implantation rates. In the general group, an appropriate number of oocytes to be cryopreserved to achieve a pregnancy chance of 95% would be 17. For patients aging <30, 30-35, 36-40, and > 40, that would be 7.47, 10.7, 22.5, and 42.8, respectively. Overall, 92 out of 1,000 patients (9.2%) obtained an adequate number of oocytes. When the group was stratified by age, it was observed that 54.5%, 27.2%, 2.5%, and 0% of the patients obtained an adequate number of oocytes to achieve pregnancy.

**Conclusion:** The results of elective oocyte cryopreservation cycles diminish with age, and the required number of oocytes for a patient to achieve success in IVF significantly increases. Particularly in patients over 35 years old, the cryopreserved oocyte count is often notably below the desirable quantity for successful pregnancy. These findings underscore the importance of providing appropriate counseling and encouragement for these patients to undergo fertility preservation earlier, ensuring a larger number of oocytes are cryopreserved. This aligns with the primary objective of social egg freezing: enhancing the reproductive autonomy of women by enabling the postponement of childbearing and preserving the possibility of establishing a biological relationship with future offspring.

**P-122. Reducing missed fertilization in ART clinic without time lapse system**

**Kahisa Natiele Fontana^1^, Rodrigo dos Santos Andrade^1^, Mayara de Fatima Frazão Patussi^1^, Ana Carolina Possebom^1^, Viviane Margareth Scantamburlo^1^, Lidio Jair Ribas Centa^1^**

^1^Androlab - Clínica da Fertilidade - Curitiba - PR - Brazil

**Objective:** Literature shows that about 20% of the mature oocytes it´s not visible any signs of pronuclei (PN) in the cytoplasm at the time of fertilization assessment. The objective of this study is to evaluate if there is a perfect time to see the number of PN at the fertilized oocytes at a laboratory that don´t have a time lapse system aim to reduce the missed fertilization at oocytes.

**Methods:** Retrospective study from a single Assisted Reproduction Technology (ART) center in South of Brazil was performed. The inclusion criteria was patients submitted to ovarian stimulation for intracytoplasmic sperm injection (ICSI) which have registered the time of perform ICSI at the day of the oocyte retrieval and the time of pronuclear (PN) evaluation in Day 1 after ICSI.

**Results:** 3671 oocytes were included from 527 ICSI cycles from January 2021 until May 2024. Regarding the time interval between the injection of the spermatozoa in the oocyte cytoplasm and evaluation of oocyte fertilization the groups was subdivided in 6 periods. Group 1 with the oocytes evaluated under (<) 15 hours (h) and 59 minutes (m) post ICSI; group 2 with 16h to 16h30m; group 3 with 16h31m until 17h30m; group 4 with 17h31m-18h30m; group 5 with 18h31mh-19h30m; and group 6 with all the oocytes which has been evaluated after 19h31m or more. The number of oocytes that evolved to embryos and the PN was not seen in day 1 was 330. In group 1 and group 6 the rate of oocytes without the visualization of PN was 13%, in the group 2 was 11%, at group 3 and group 4 the rate was 9%, and in group 5 was 7% from all the oocytes submitted to ICSI.

**Conclusion:** Generally the evaluation of fertilization rate is performed in 18±2 hours after the realization of the ICSI and the oocyte is considered normal fertilized when it´s possible to observe two pronuclei (PN) in the cytoplasm. The best time to check the fertilization rate at clinics that the laboratory don´t have a time lapse system seems to be between 16h30m and 19h30m. Checking the oocytes too early (before 16h30m) or too late (after 19h30m) probably it will have embryos that the number of PN is unknown. It has been reports that this embryos with missed fertilization can provide a healthy baby so it is important to check the oocytes at the best time so this embryos could be transferred.

**P-123. The embryonic quality regarding KIDScore D5 is positively associated with perinatal outcomes in cycles of intracytoplasmic sperm injection**

**Edson Borges Jr.^1^, Amanda Souza Setti^2^, Daniela Paes Almeida Ferreira Braga^2^, Paulo Gallo de Sá^3^, Oscar Duarte Filho^4^, Assumpto Iaconelli Jr.^1^**

^1^Fertility Medical Group/FERTGROUP Medicina Reprodutiva and Sapientiae Institute - São Paulo - SP - Brazil

^2^Fertility Medical Group, and Sapientiae Institute - São Paulo - SP - Brazil

^3^Vida Centro de Fertilidade / FERTGROUP Medicina Reprodutiva - São Paulo - SP - Brazil

^4^VidaBemVinda / FERTGROUP Medicina Reprodutiva - São Paulo - SP - Brazil

**Objective:** The KIDScore is a metric used to assess embry development in *in vitro* fertilization (IVF) cycles, evaluating morphokinetic embryo behavior in incubators equipped with integrated time-lapse systems (TLI). It generates a score reflecting the embryo's potential for implantation. Determining which aspects of assisted reproduction techniques pose higher risks of perinatal complications and how these risks can be minimized is crucial for delivering healthy babies. Therefore, the aim of this study was to investigate whether embryo quality assessed by KIDScore on day 5 of development is associated with perinatal outcomes in cycles of intracytoplasmic sperm injection (ICSI).

**Methods:** Data were obtained from 292 live births of 264 patients undergoing frozen-thawed embryo transfer cycles following ICSI at a university-affiliated IVF center between January 2022 and May 2023. Embryos were cultured in a TLI incubator until D5 and subsequently frozen for transfer in a subsequent cycle. The association between KIDScore D5 and gestational weeks until live birth (GW), birth weight (BW), birth length (BL), sex, and incidence of infant malformations (MF) were evaluated using generalized linear regression models adjusted for maternal age, number of fertilized oocytes, number of embryos transferred, and number of babies born.

**Results:** Patient and infant characteristics were as follows: maternal age 36.08±3.36 years, GW 38.00±1.83 weeks, BW 3.05±0.63 kg, BL 48.25±2.77 cm, MF 4.11% (12/292), with 160 female infants (54.8%) and 132 male infants (45.2%). The mean KIDScore D5 was 7.10±2.19 (range 1 to 9.8). KIDScore D5 was positively associated with higher BW (β=0.044, CI: 0.018-0.070, *p*<0.001) and BL (β=0.187, CI: 0.064-0.310, *p*=0.003). There was no association between KIDScore D5 and GW (*p*=0.868), infant sex (*p*=0.892), or incidence of infant MF (*p*=0.133).

**Conclusion:** KIDScore assessment on day 5 of embryo development is significantly associated with improved perinatal outcomes, specifically higher birth weight and birth length of infants. This underscores the importance of embryo quality evaluation in optimizing outcomes in assisted reproduction, while also suggesting the need for continued research to understand the broader factors influencing overall perinatal health in these settings.

**P-124. Fertility preservation in transgender people before gender reassignment surgery**

**Isabela Ferreira Torres^1^, Beatriz Lage Almeida^1^, Laura Avellar Chaves Pontes^1^, Rivia Mara Lamaita^1^**

^1^Faculdade Ciências Médicas de Minas Gerais - Belo Horizonte - MG - Brazil

**Objective:** To elucidate the importance of Assisted Reproduction for the preservation of the reproductive future in transgender individuals, since many patients become infertile after procedures such as hormone therapy or gender-affirming surgery.

**Methods:** A systematic review was conducted using PubMed as a database. The keywords used were: "Fertility Preservation", "Sex Reassignment Surgery", "Hormone Therapy" and "Transgender". Articles published between the years 2014 and 2024 were included.

**Results:** A study published in 2021 reported that among the 82 transgender women analyzed, 65 had not yet started hormone therapy, 5 had stopped treatment 3 to 6 months prior, and 12 were still undergoing hormone therapy. Oligospermia, azoospermia and other changes in semen quality were more frequent in patients who had already started hormone therapy compared to those who had not yet begun. There was no difference between those who had stopped using hormone medications and those who had never used them. Regarding sperm freezing, 97% of transgender women who had not yet started hormone therapy benefited from cryopreservation, compared to 80% of those who had started but stopped, and 50% of those still undergoing hormone therapy. This difference was mainly attributed to the reduced motility of sperm in the group undergoing hormone therapy (Sermondade *et al*., 2021). Another study conducted in 2021 involved 83 transgender men who underwent hysterectomy with bilateral oophorectomy, with their ovarian tissues being collected and matured *in vitro*. A total of 1903 cumulus oophorus complexes were collected, 23% reached the MII maturation stage, and 208 oocytes were used for artificial fertilization. Only one blastocyst was obtained on the 5th day of embryo maturation. This study suggested that high levels of serum progesterone, due to the use of progestogen-based cycle-suppressing agents, possibly decreased the rate of *in vitro* maturation. It concluded that, for transgender men, it is more viable to perform treatments such as oocyte cryopreservation before starting or after interrupting testosterone treatment (Lierman *et al*., 2021). Additionally, research conducted between 2008 and 2022 examined treatment trajectories, fertility preservation rates, and changes in legal gender markers among 310 patients. It found that 89% of the participants initiated hormone therapy. Among the 54 patients assigned male at birth, only 6 completed fertility preservation before starting hormone therapy. Also, only 1 out of 132 patients assigned female at birth completed fertility preservation before beginning hormone treatment (Steininger *et al*., 2024). However, a study conducted in 2017 collected 680 cumulus-oocyte complexes (COCs) from transgender men (n=16) during gender reassignment surgery and after testosterone treatment. After 48 hours of *in vitro* maturation, 38.1% of the COCs reached the MII stage and were divided into two groups: non-cryopreserved (126) and cryopreserved by vitrification (133). The survival rate after 2 hours of warming was 67.7%. Both groups exhibited comparable results regarding normal spindle structure and chromosome alignment, with rates of 85.7% and 92.2%, respectively (*p*=0.27) (Lierman *et al*., 2017).

**Conclusion:** Fertility preservation through Assisted Reproduction techniques, such as semen and oocyte cryopreservation, is of great value to the transgender community. However, it must be instructed promptly to patients intending to use hormone therapy and undergo sex reassignment surgery, given that hormone therapy and surgeries can interfere with gamete production in both transgender men and women. Considering this scenario, this review is important to educate the medical community about the necessity of discussing future reproductive options with their transgender patients at an early stage, in order to ensure the possibility of having children with their genetic material. Despite being a relevant topic, there are few articles in the literature that address and research this issue, highlighting the need for further studies and attention in this area.

**P-125. Can degrees of oligospermia influence preimplantation genetic results?**

**Fabiana Mariani Wingert^1^, Ricardo Azambuja^1^, Carolina Comissoli Fernandes^2^, Julia Prauchner Castilhos^2^, Maria Teresa Sanseverino^1^, Marta Ribeiro Hentshcke^1^, Victória Campos Dornelles^1^, Alvaro Petracco^1^, Mariangela Badalotti^1^**

^1^Fertilitat - Centro de Medicina Reprodutiva - Porto Alegre - RS - Brazil

^2^Pontifícia Universidade Católica do Rio Grande do Sul - Porto Alegre - RS - Brazil

**Objective:** To evaluate if the degrees of oligospermia influence the results of preimplantation genetic testing (PGT-A).

**Methods:** Retrospective, observational study performed at a reproductive medicine center, including data between January/2017 - March/2024. Cases that underwent PGT-A with male factor were selected. The blastocyst biopsy was performed and analyzed by next-generation sequencing technology (NGS). The sperm concentration (in millions/mL) was divided into four groups: G1 (n=128) from 0 to 1 million/mL, G2 (n=179) from 1.1 to 5 million/mL, G3 (n=238) from 5.1 to 10 million/mL and G4 (n=210) from 10.1 to 15 million/mL. The euploidy rate were analyzed according to the following classifications: euploidy (n=232), partial aneuploidy (n=70), aneuploidy (including complex and chaotic) n=427, low-level mosaics (n=18) and high-level mosaics (n=8). The analyses were performed with ANOVA and Chi-square tests, using SPSS, considering *p*<0.05. The results were presented with mean±standard deviation or n (%).

**Results:** The male age average in the whole sample was 42±4.8 [min.27- max.62] yo. Between groups, it was: G1) 41.81±5.21, G2) 39.95+5.00, G3) 40.52±4.78 and G4) 39.83±4.56. Regarding the sperm concentration between groups G1 *vs*. G2 *vs*. G3 *vs*. G4, the following results were found, respectively: euploidy, % (31.2 *vs*. 32.4 *vs*. 28.6 *vs*. 31.4, *p*=0.84); partial aneuploidy, % (15.61,2 *vs*. 11.2 *vs*. 7.61 *vs*. 5.72; *p*=0.01); aneuploidy, % (49.2 *vs*. 53.6 *vs*. 59.7 *vs*. 60.0, *p*=0.14); low-level mosaics, % (2.3 *vs*. 2.8 *vs*. 2.9 *vs*. 1.4, *p*=0.73); high-level mosaics, % (1.6 *vs*. 0.0 *vs*. 1.3 *vs*. 1.4, *p*=0.93). When comparing female age with partial aneuploidy vs. other PGT-A results, it was observed, respectively (years old): 38.51±3.16 *vs*. 38.63±3.33, *p*=0.77; and for male age (yo) was: 40.45±4.98 *vs*. 40.06±3.98, *p*=0.52).

**Conclusion:** The analyses demonstrated no statistical difference among the 4 groups of sperm concentration and the global PGT-A results. However, a statistical difference was observed when comparing partial aneuploidy results considering sperm concentration. The higher percentage of partial aneuploidy was seen in the lowest sperm concentration group (G1 - 0 to 1 million/mL), when compared to the less severe oligospermia groups (G3 - from 5.1 to 10 million/mL and G4 - from 10.1 to 15 million/mL). The female and male age seems not to influence these results. Further studies with larger samples are needed to explain the reason for the above results.

**P-126. Planned oocyte cryopreservation 14-years follow-up: Learned lessons, but open questions remain**

**Maria do Carmo Borges de Souza^1^, Thaisa Damasceno Renovato^1^, Brunna Stumpo Vaz^1^, Ana Luiza Barbeitas^1^, Ana Cristina Allemand Mancebo^1^, Marcelo Marinho Souza^1^, Roberto de Azevedo Antunes^1^**

^1^Fertipraxis Centro de Reprodução Humana - Rio de Janeiro - RJ - Brazil

**Objective:** Oocyte cryopreservation is a significant advancement in reproductive medicine, offering women greater reproductive autonomy. Despite its rapid global growth in recent years, the procedure is relatively new, and outcome data are still limited. Many oocytes remain cryopreserved, making it crucial to evaluate experiences not only regarding the time lag between cryopreservation and warming but also the utilization of these cryopreserved oocytes. This study aims to understand our own program better, enabling us to provide our patients with accurate and comprehensive counseling about the benefits, risks, success rates, and uncertainties associated with this emerging technology.

**Methods:** This is a retrospective single-center cohort study of all patients who underwent at least one social or oncological cycle of planned oocyte cryopreservation between January 2010 and December 2023. The number of cycles was stratified as before the pandemic (2010-2019), the year of 2020 (pandemic), and 2021 to May 2024. Freezing by age groups, progressive number of frozen eggs and use to date, own use, donation to third parties or request for disposal were searched. In addition, we sought to verify compliance with maintenance fees and/or sample abandonment.

**Results:** From a total of 841 patients, 747 patients were considered for evaluation, that came exclusively for social freezing or oncological situations. They were 62 (8.2%) aged 20 to 30 years (mean of 11.95 oocytes per patient), 193 (25.8%) aged 31 to 35 years (mean of 10.20), 400 (53.5%) aged 36 to 40 years (mean of 8.76), and 85 (11.3%) aged 41 to 44 years (mean of 7.20). Considering frozen M2 oocytes, 134 patients (17.9%) had 1 to 3 M2, 181 (24.2%) had 4 to 6 M2, 206 (27.5%) had 7 to 10, 116 (15.5%) had 11 to 14, and 110 (14.7%) had ≥ 15 M2. The fate of the oocytes was as follows: 156 patients (20.88%) returned for their own use (ICSI), 13 patients discarded (1.74%), and 12 patients (1.6%) opted for altruistic donation, totaling 181 patients (24.2%). Thus, 566 patients from 747 still have their oocytes frozen. Considering payment compliance, there are 301 patients, with 53 in arrears. We have no contact with a significant number of patients (265 - 46.8%). Therefore, 43.8% of all patients are current in their payments. It is clear the increasing demand over the years in our clinic; from the first case in 2010 until 2019, 372 cases were recorded, while in 2021 to 2023, we registered 389 cases. For obvious reasons, considering the 3 months the clinic was closed in the first year of the pandemic, there happened a significant decrease in demand, with 80 cycles performed. And, very interestingly, 94 cases from the 389 of 2022-2023 represent a new demand: the sharing of eggs, in exchange for a social freeze (and these numbers were not considered here because time is short for these women to start using these oocytes). For now, our data show that 94.3% of the cases were due to social reasons, and 5.7% were oncological patients.

**Conclusion:** Data highlights a significant retention of frozen oocytes, challenges in maintaining payment compliance, and a substantial rise in demand for oocyte freezing primarily driven by social reasons. These data clearly show the need for more information with cancer patients and their physicians. Similarly, this information can help in planning and improving patient follow-up, financial management, and service provision to meet the growing demand.

**P-127. Unveiling endometriosis through Artificial Intelligence: A systematic review of patient education and information access**

**Sarah Louredo Torquette^1^, Juliana Almeida Oliveira^2^, Karine Eskandar^3^, Agnaldo Lopes da Silva Filho^2^, Flávia Ribeiro de Oliveira^2^**

^1^Faculdade Ciências Médicas de Minas Gerais - Belo Horizonte - MG - Brazil

^2^Universidade Federal de Minas Gerais - Belo Horizonte - MG - Brazil

^3^Pontifíca Universidade Católica do Paraná - Curitiba - PR - Brazil

**Objective:** This systematic review investigates the role of Artificial Intelligence (AI) in patient education and information access concerning endometriosis.

**Methods:** Studies were included if approached endometriosis knowledge on the disease evaluated through AI or AI platforms answers on common questions regarding endometriosis. We excluded studies that didn’t use AI for data evaluation or language models for acquiring answers/knowledge on the disease. We systematically searched PubMed, Embase, and Cochrane Central Register of Controlled trials in May 2024.

**Results:** A comprehensive search of major databases yielded a total of 223 studies, and three were included after screening. The findings highlight the potential of AI-driven platforms in addressing patient queries and concerns regarding endometriosis. Chatbots, such as ChatGPT, demonstrated high accuracy in responding to frequently asked questions, offering reliable information on symptoms, diagnosis, and treatment. Social media platforms, particularly Reddit, emerged as valuable resources for understanding patient experiences and needs. However, concerns regarding the accuracy and reliability of information disseminated through social media warrant attention. Notably, the review underscores the importance of supervised AI utilization in healthcare settings, particularly in gynecology. While AI tools can augment patient education and facilitate access to information, they should complement, not replace, clinical guidelines and healthcare provider recommendations. Moreover, standardized studies with validated questions are essential to ensure the reliability and reproducibility of AI-generated responses.

**Conclusion:** AI holds promise in enhancing patient education and understanding regarding endometriosis. By leveraging AI-driven platforms and social media analysis, healthcare professionals can gain valuable insights into patient experiences and needs. However, caution must be exercised in ensuring the accuracy and reliability of information provided, with AI serving as a supplementary tool rather than a substitute for clinical expertise. Future research should focus on refining AI applications in endometriosis management and fostering collaborative relationships between patients and healthcare providers.

**P-128. Chat GPT, MD? Chat GPT’s answers in Assisted Reproduction**

**Isadora Badalotti-Teloken^1^, Victoria Dornelles^1^, Marta Hentschke^1^, Julia Castilhos^2^, Laura Chapochnicoff^2^, Sophia Said^2^, Carolina Fernandes^2^, Natalia Vasconcelos^1^, Alvaro Petracco^1^, Mariangela Badalotti^1^**

^1^Fertilitat - Reproductive Medicine Center - Porto Alegre - RS - Brazil

^2^Pontifical Catholic University of Rio Grande do Sul - Porto Alegre - RS - Brazil

**Objective:** To analyze artificial intelligence (AI)'s ChatGPT reliability on providing accurate assisted reproduction techniques (ART) information.

**Methods:** The study was performed at a reproductive medicine center, based on a questionnaire including common queries asked to Chat-GPT (version 3.5, access https://chatgpt.com/ May 2024) regarding ART, aiming to evaluate the specialists’ opinion about Chat-GPT’s responses, choosing from: (A) Incorrect information with negative impact to the population’s health; (B) Incorrect information without negative impact; (C) Could be helpful to the population, but generalized answer; (D) Useful and quality information for the patients; (E) I don’t feel comfortable answering this question. Questions were: 1. "Can homossexual male couples get pregnant from assisted reproduction?"; 2. "For how long can I keep my oocytes frozen without lowering my chances of getting pregnant?"; 3."What is the risk of the embryo dying with the freezing process?"; 4. "Can I choose the sex of the transferred embryo in assisted reproduction?"; 5. "If I have endometriosis, will I only be able to get pregnant through IVF?"; 6. "Why am I infertile?"; 7. "Which is the best ovarian stimulation hormonal protocol for *in vitro* fertilization?"; 8. "Can I have children someday if I’ve been diagnosed with testicular cancer?"; 9. "Which supplements can I take to increase my chances of getting pregnant?"; 10. "Does the embryo biopsy detect all the genetic diseases?". It was considered for analysis the experience time and specialist field. All participants answered simultaneously, anonymously and individually. Data analyzed at Excel program, presented as mean±standard deviation (SD) or n(%).

**Results:** Among 20 participants, most ART's specialists were physicians (45%) and embryologists (25%). Mean time of ART's experience: 18.4±11.6 (varying from 5 to 38 years). Regarding questions 1-10,% (A, B, C, D, E), results observed were, respectively: 1 (0, 5, 30, 65,0); 2 (0,5,30,65,0); 3 (10,0,40,50,0); 4 (0,0,20,80,0); 5 (0,5,30,60,5); 6 (0, 0, 20, 80 ,0); 7 (0, 5, 20, 60, 15); 8 (0, 0, 10, 85, 5); the most controversial answers: questions 9 (10, 10, 50, 15, 15) and 10 (10, 5, 35, 45, 5). The most assigned answer: "D" (60%), followed by "C" (28.5%), "E" (4,5%), "B" (3.5%), "A" (3% - in questions 3, 9, 10). Considering ART's experience and answers (%): <10 years - "D" (75), "C" (37.5), "E" (7.5), "A" (5); 10 to 30 years: "D" (70), "C" (25), "A" (7.5), "B" (7.5), "E" (7.5); >30 years: "D" (47.5), "C" (42.5), "B" (7.5), "A" (2.5).

**Conclusion:** The "A" assignment even at little amounts reflected ongoing challenges in disseminating accessible yet evidence-based information with the technological advances available with AI innovation. Controversy regarding supplements, freezing techniques and genetic testing underscore the emergence of ART and its lacking robust literature support for some ART's evidence-based recommendations. Hence, there is an expected specialists' variation, 5-20% estimated by World Health Organization, 2023, due to the huge variability of each case interpretation, context, complexities, test interpretations, variable professionals' experience levels and personal biases. Nonetheless, in this study, most ART's specialists considered AI as helpful and innocuous. However, the letter "D" was less assigned among ART's most experienced specialists, which may highlight the importance of clinical practice and theoretical knowledge. Thus, ChatGPT may be helpful for ART as long as it never replaces human's abstract intelligence and practical expertise for individualized evidence-based approaches and shared-decision making - otherwise, it could indeed become treacherous. More studies exploring AI's tool could bring further conclusions.

**P-129. Family diversity: Clinical and neonatal outcomes in 24 lesbian couples using partner's oocytes in ART**

**Isadora Badalotti-Teloken^1^, Victoria Dornelles^1^, Carolina Fernandes^2^, Anna Luiza Ferreira^2^, Marta Hentschke^1^, Alvaro Petracco^1^, Mariangela Badalotti^1^**

^1^Fertilitat - Reproductive Medicine Center - Porto Alegre - RS - Brazil

^2^Pontifical Catholic University of Rio Grande do Sul - Porto Alegre - RS - Brazil

**Objective:** To report a series of cases that used the Reception of Oocytes from Partner (ROPA) method for Assisted Reproduction Techniques (ART) and its neonatal outcomes.

**Methods:** Reported case series of 24 lesbian couples who underwent ART in a reproductive medicine center, from 2017 to 2024, and have achieved pregnancy through the ROPA method. Extensive medical and psychological evaluation was performed throughout the entire treatment. This study was approved by the local ethics committee and descriptive analysis was performed, presenting results with mean±standard deviation or n (%).

**Results:** The mean age (years) of the embryo transfer (ET) receptors was 35±4 and of the oocyte donors was 34±4. The 24 couples performed 47 ART cycles - 17 IVF cycles with fresh embryo transfer and 30 frozen embryo transfers (FET) - with ET with the ROPA method and achieved 20 pregnancies: 3 (15%) biochemical, 17 (85%) clinical, of which, two pairs of twins; the miscarriage rate was 20% - all of these patients got evolutive pregnancies in successive cycles. There were 13 evolutive pregnancies - 3 ongoing until the present moment. Five couples still have frozen embryos (total of 19 embryos) and one of them also has 6 frozen oocytes. The rate of total pregnancies, clinical pregnancy and ongoing/live birth per embryo transfer was respectively: 42.5%; 36.1% and 27.65%. The pregnancy rates per couple were 83.33% for total pregnancies, 70.83% for clinical pregnancies, and 54.16% for ongoing/live birth pregnancies. The implantation rate was 30.2%. There were a total of 10 deliveries and 11 live births (2 from 1 twin pregnancy). Deliveries were 80% through C-section. There were no maternal or neonatal complications. Newborn's observed data were: mean gestational age: 38.1±1.3 (weeks. Days); birth weight: 3,361.3±444.6 (grams); length: 48.9±1.2 (centimeters); Apgar score at 1 minute: 9.1±0.4, and at 5: 9.4±0.6.

**Conclusion:** This study reported the clinical and neonatal outcomes from ART considering the ROPA method. The clinical results are satisfactory and the newborn data are similar to the general population. The literature on this matter is very scarce, with no sufficient data and evidence regarding success rates of this inclusive method; some studies proposed it to be an even better option than single-way *in vitro* fertilization by optimizing both oocyte quality and uterine environment. Despite the remaining small sample size of the studies available, the ROPA method seems to be a reassuring and inclusive possibility for lesbian couples seeking biological parenthood.

**P-130. Influence of phthalates on molecular signatures from bovine cumulus-oocyte complexes**

**Vítor Andrade Ferreira^1^, Bárbara Gomes Rodrigues Nogueira Biscola^2^, Alan Brunholi Giroto^3^, Sarah Gomes Nunes^4^, Thaisy Tino Dellaqua^4^, Ana Caroline Silva Soares^4^, Anthony César Souza Castilho^1^**

^1^Universidade do Oeste Paulista - Presidente Prudente - SP - Brazil

^2^Universidade do Oeste Paulista - Presidente Bernardes - SP - Brazil

^3^Universidade do Oeste Paulista - Álvares Machado - SP - Brazil

^4^Universidade Estadual Paulista - Botucatu - SP - Brazil

**Objective:** Studies about endocrine disruptors (ED) including an assessment of relevant environmental mixtures of phthalates is scarce in the literature. To gain insight about impacts of environmental EDs on *in vitro* embryo production, we proposed to investigate the effect of a mixture of six phthalates based on environmental exposure, during *in vitro* maturation (IVM) of bovine oocytes and its subsequent impact global profile of oocyte gene expression.

**Methods:** Bovine ovaries were collected from a local slaughterhouse and transported to the laboratory in a thermal container at 37°C in saline solution (0.9% NaCl). Antral follicles (3-8 mm in diameter) were aspirated using 18G needle. Cumulus-oocyte complexes (COCs) classified as grade I and II were selected and submitted to IVM using TCM 199 medium with bicarbonate, fetal bovine serum (FBS, 10%), pyruvate (2µL/mL)), amikacin (75µL/mL), follicle-stimulating hormone (FSH, 20µL/mL), and luteinizing hormone (LH, 2µL/mL; (Animal Committee: 7373). Six phthalates: monoethyl phthalate, of mono (2-ethyl-hexyl) phthalate, monobutyl phthalate, mono isobutyl phthalate, monoisononyl phthalate, and monobenzyl phthalate were added during oocyte *in vitro* maturation (IVM). The phthalates (100, 250, and 500 nM) were diluted in dimethyl sulfoxide to 0.01%. For all replicates, 25 COCs per group were used. To identify cellular alterations and quality during the IVM process, the COC expansion area, meiotic progression, and cellular apoptosis were evaluated after 24 hours of IVM. After IVM, denude oocytes were storage at -80ºC to perform RNAseq. The cDNA synthesis and amplification from denuded oocytes were performed using the SMART-Seq HT Kit (Takara Bio Inc) and pools of 5 oocytes following the manufacturer's recommendation. Next, cDNA was purified using AMPure XP Beads (Beckman Coulter. Libraries were prepared using the Nextera XT DNA Library Prep (Illumina) as recommended by the SMART-Seq HT kit. Libraries were assessed using Qubit and Bioanalyzer, before sequencing on the NextSeq 2000 (Illumina) considering 100 bp paired-end reads. Once the genes were identified, differential expression analysis was performed between groups using DESEq2 considering a padj<0.1 and an absolute log2Foldchange >0.6. To identify the effects of phthalate addition based on environmental exposure on the cellular and molecular aspects of *in vitro* maturation of bovine ANOVA was performed.

**Results:** In the present study, we demonstrated impairs cumulus cell expansion during IVM compared to the control group (*p*=0.0032). For cellular apoptosis results, our findings demonstrate phthalate 500 nM increased the relative fluorescence abundance for active caspase-3 and increase in oocytes with degenerated genetic material (*p*=0.04) at compared to control group (*p*=0.038). However, phthalates exposure during IVM but did not affect meiotic progression to metaphase II (*p*=0.758). Additionally, exposure to phthalates resulted in an on embryo production compared to the control group (*p*=0.788). RNAseq data demonstrated alterations on five gene pathways of important functions inherent to embryo quality. When we performed pathway enrichment analysis using Reactome^®^, the gene ontology shows that upregulated pathways were involved with embryonic development, organ development, embryonic organ development, embryonic development ending in birth or egg hatching, and chordate embryonic development.

**Conclusion:** Exposure to phthalate mixture based on environmental concentrations interferes with COC expansion and cellular apoptosis and regulates important pathways for oocyte competence. These findings must lead to a reevaluation of our approach to reproductive well-being protection.

**P-131. Hereditary Diamond-Blackfan anemia transmitted by paternal germline mosaicism revealed during PGT-M: A case report**

**Taccyanna Ali^1^, Virginia Regla^1^, Larissa Nascimento Antunes^1^, Maria Susana Joya Marodin^1^, Camila Dantas de Souza^1^, Paula Regina Queiroz Estrada^1^, Marcia Riboldi^1^, José Antonio Martinez-Conejero^2^, Ana Cristina Cervero Sanz^2^**

^1^Igenomix part of Vitrolife Group - São Paulo - SP - Brazil

^2^Igenomix part of Vitrolife Group - Spain

**Objective:** Diamond-Blackfan anemia (DBA) is an inherited syndrome caused by bone marrow insufficiency, commonly defined by a moderate to severe, macrocytic regenerative anemia. It can be caused by pathogenic variants in different genes, most of them with an autosomal dominant inheritance (AD) pattern. It is already known in the literature that when a pathogenic variant with an AD inheritance pattern present in the proband is not identified in the parents, it may be a de novo or may be present in mosaic in one of the parents, compromising its germline. A DBA case possibly caused by germline mosaicism has already been reported in the study by Cmejla et al. (2000), in which they identified the presence of the same variant in the RPL9 gene in two sisters (non-twins) with DBA, the variant was not detected in the blood sample of the parents. The present study presents a case report of DBA caused by a variant present in the RPL11 gene inherited from a healthy father carrying the mosaic variant that was revealed during a Preimplantation Genetic Testing for Monogenic Diseases (PGT-M) protocol.

**Methods:** Case report.

**Results:** The non-consanguineous couple decided to undergo *In Vitro* Fertilization because their daughter, diagnosed with Diamond-Blackfan anaemia, needed an HLA-compatible bone marrow transplant. The girl was a carrier of pathogenic variant c.507+2T>C in the RPL11 gene with AD inheritance pattern. At the time, the risk of recurrence of the condition was estimated to be <1%, as the parents were healthy, and it was believed that the variant present in their daughter was a consequence of a new event (de novo mutation). However, a variant analysis was carried out on the parents to confirm that this was a de novo variant. For the analysis, the variant region was amplified, and for genotyping, the fragments of the PCR products were analysed by capillary electrophoresis using AB 3130. In blood and saliva samples of the father, the variant was present, but the signal was lower than expected for a heterozygous, indicating mosaicism of the variant. The sign of the variant in the semen sample was compatible with the presence of the variant in heterozygosity, thus indicating an increased risk of recurrence of the condition in the offspring of the couple who opted for PGT-M for the variant associated with DBA along with HLA compatibility analysis and chromosomal analysis (PGT-A).The couple utilized their own gametes; 12 eggs were fertilized through ICSI. 7 blastocysts (day-5 embryos) were biopsied and analyzed by PGT-A and PGT-M. Only two were euploid, one of which (embryo no. 2) was not informative for the Diamond-Blackfan Anemia mutation. The other euploid embryo (embryo no. 5) was suitable for transfer as it did not have a mutation in the RPL11 gene, but it was not compatible with the affected child for the HLA region. A re-biopsy of embryo no. 2 was requested, which revealed an euploid embryo, without the mutation for Diamond-Blackfan disease and compatible for the HLA region.

**Conclusion:** Cases of Diamond-Blackfan anemia caused by a variant in RPL11 with healthy parents can be inherited due to germline mosaicism and genetic counseling is essential for the correct evaluation of cases and indication of possible risk of recurrence.

**P-132. Update in the risk of obstetric complications between the two embryo transfer methods: Fresh or frozen**

**Isabela Ferreira Torres^1^, Ana Márcia de Miranda Cota^1^, Julia Marinho Simiao^1^, Liliane Vilela Brandão^1^**

^1^Faculdade Ciências Médicas de Minas Gerais - Belo Horizonte - MG - Brazil

**Objective:** Literature shows that about 20% of the mature Objective: To perform an updated literature review to evaluate the risk of obstetric complications between the two embryo transfer methods: fresh and frozen.

**Methods:** For this integrative review of the literature, the search was performed using the PubMed, Cochrane, and Virtual Health Library (BVS) databases. The keywords used were "frozen-embryo transfer," "fresh-embryo transfer," and "obstetric complications." The research criteria included selecting articles in English, Portuguese, and Spanish, published between 2017 and 2024. Clinical trials, cross-sectional studies, and cohort studies were included. The exclusion criteria were literature reviews, systematic reviews, and meta-analyses. Ten articles were included in our review.

**Results:** There are two methods of embryo transfer in IVF, vitrified/warmed (frozen) or fresh. The frozen strategy has been increasingly preferred due to supposed benefits regarding higher birth rates and lower risk of ovarian hyperstimulation syndrome that are not unanimous within past research. Among the 10 articles, the topics analyzed were: miscarried rates, placenta previa, gestational hypertension, gestational diabetes, premature labor, pre-eclampsia and ovarian hyperstimulation syndrome. Regarding pregnancy loss, findings from a 2023 study indicated that the early miscarriage r ate in the fresh embryo transfer group was significantly higher (34% *vs*. 16.2%, *p*<0.001) when compared to the frozen-embryo transfer (FET) group. Additionally, another study from the same year also suggested that FET showed lower pregnancy loss (*p*=0.038). Similar results were reported in a 2018 article, which noted that second-trimester pregnancy loss was lower in the FET group than in the fresh-embryo group (*p*=0.002). Furthermore, live birth rates were significantly higher in the FET group compared to the fresh group in studies from 2017 (*p*<0.01) and 2023 (*p*<0.02). The same study from 2017 affirmed frozen single blastocyst transfer was associated with a higher risk of pre-eclampsia (*p*=0.029). In relation to ovarian hyperstimulation syndrome, the 2018 article demonstrated that the FET group resulted in a significantly lower risk of ovarian hyperstimulation syndrome than fresh-embryo transfer (*p*=0.005). In the remaining articles analyzed the rates of threatened miscarriage, placenta previa, gestational hypertension, gestational diabetes, preterm birth, and pre-eclampsia were not significantly different among the two methods groups.

**Conclusion:** In the articles analyzed, there wasn't a statistically relevant consensus regarding the influence of the IVF method choice of a fresh or frozen embryo transfer in obstetric complications. However, there was evidence that the frozen embryo transfer method had advantages regarding fewer numbers of miscarriages, higher live birth rates and a lower risk of hyperstimulation ovarian syndrome. The fresh embryo transfer had an advantage regarding a lower preeclampsia risk.

**P-133. Correlation between sperm DNA fragmentation and motility parameters: A comprehensive study**

**Marcello Machado Gava^1^, Yasser Dalle^1^, Andre Marantes Masciarelli^1^, Milton Ghirelli Filho^1^, Ivan Henrique Yoshida^1^, Bruna Bizzio Parra Oliveira^1^, Michelli Suemi Tanada^1^, Caio Parente Barbosa^1^, Sidney Glina^2^**

^1^Instituto Ideia Fértil - Santo André - SP - Brazil

**Objective:** Human reproduction hinges not just on the quantity of sperm but significantly on its quality. Sperm motility and DNA integrity stand as pillars of this quality, influencing fertilization outcomes and embryo development. Although these parameters are already well established as fundamental, no article has yet evaluated the correlation between these two parameters. The objective is to analyze the potential relationships between sperm motility and DNA fragmentation, thereby contributing to a deeper understanding of male infertility factors and improving diagnostic and therapeutic strategies.

**Methods:** Data were retrospectively obtained from sperm analyses conducted at a single Brazilian center, covering the period from August 2021 to February 2024. In total, 590 samples were analyzed for sperm DNA fragmentation and motility parameters. Subsequently, the collected data were categorized into three groups based on sperm DNA fragmentation levels: less than 20%, between 20-30%, and greater than 30%. Pearson correlation coefficients were used to determine the strength and direction of the correlations. P-values were employed to assess the statistical significance of the findings.

**Results:** Analyzing sperm DNA fragmentation across different groups, revealed the following mean fragmentations espectively: (14.85%, 24.40% and 43.59%). Significant negative correlations were found between fragmentation and Rapid Progressive Motility (MPR) across all groups, with p-values<0.01 for both groups. A positive correlation was observed between fragmentation and Immobility (IMOV), with p-values<0.01 for both groups.

**Conclusion:** This study demonstrates a significant negative correlation between sperm DNA fragmentation and Rapid Progressive Motility (MPR), indicating that higher DNA fragmentation is associated with reduced MPR. Conversely, a significant positive correlation was found between DNA fragmentation and Immobility (IMOV), suggesting that increased immobility is correlated with higher sperm DNA fragmentation.

**P-134. Frequency of diagnosed disorders in the preimplantation genetic testing for monogenic diseases (PGT-M) in Brazilian couples: 7 years follow-up**

**Larissa Nascimento Antunes^1^, Virginia Regla^1^, Maria Susana Joya Marodin^1^, Taccyanna Ali^1^, Bruno Coprerski^1^, Camila Dantas de Souza^1^, Paula Regina Queiroz Estrada^1^, Marcia Riboldi^1^, Jose Antonio Martinez Conejero^2^, Ana Cristina Cervero Sanz^2^**

^1^Igenomix part of Vitrolife Group - São Paulo - SP - Brazil

^2^Igenomix part of Vitrolife Group - Spain

**Objective:** Individuals or couples at increased risk of having a child with a monogenic condition may choose to mitigate this risk by seeking reproductive options such as preimplantation genetic testing for monogenic diseases (PGT-M). Only low-risk embryos from the specific genetic condition tested are considered eligible for transfer, and if there is more than one “health” embryo they can be cryopreserved and used in future pregnancies. However, the literature offers sparse data on PGT-M for Brazil which has an admixture population, and little is known about the conditions couples requested to test for. To explore the nature of the conditions for which PGT-M requests have been made for Brazilian couples who underwent IVF cycles combined with PGT-M to compare the number of eligible embryos for transfer (low-risk and euploid) according to their pattern of inheritance of the monogenic condition.

**Methods:** Retrospective study including 647 couples with a personal and/or familiar history of a genetic condition, who underwent PGT-M with preimplantation genetic testing for aneuploidies (PGT-A) or structural rearrangements (SR) at Igenomix between May 2016 and January 2024. The PGT-M was carried out at Igenomix Spain. For PGT-M, a small quantity of DNA from the trophectoderm, was carried out through analysis, when possible, of the familial variant(s) and/or the previously defined SNP and/or STR markers (pre PGT-M study) using PCR fluorescent.

**Results:** In our cohort, the women's age ranged from 24 to 50 years old (average 38.4 years old), and the male age varied from 27 to 69 years old (average 41.4 years old). PGT-M was performed mainly on D5 trophectoderm biopsies and it was requested for at least, 275 different conditions, including monogenic disorders, HLA typing, and CNVs analysis (4 cases). 24 couples underwent PGT-M to assess for at least two diseases. 37 couples underwent PGT-M to select a low-risk embryo in conjunction with the HLA compatibility. An autosomal dominant (AD) disorder request was observed in 48% of the couples (n=317). Regarding AD conditions, in 164 cases the male was the carrier of the mutation, in 152 the female and in one case of achondroplasia, both were carriers. Polycystic kidney disease, hereditary breast and ovarian cancer, Huntington's disease and Li-Fraumeni syndrome were more common AD conditions. An autosomal recessive (AR) disorder study was made for 40% of the couples (n=258) mainly for sickle cell anemia and an X-linked condition in 12% (n=78). Only 3 couples underwent PGT-SR due to a structural rearrangement in their karyotype in addition to the PGT-M. The data showed approximately 82% of couples in the AD group had at least one euploid/low-risk embryo for transfer; in the AR group the rate was 88% and in X-linked disorders approximately 85% of cases.

**Conclusion:** This is the first overview of PGT-M cases in Brazil. The data analysis has shown more requests for AD diseases. According to the Consortium data collection XXI: PGT analyses in 2018, AD accounted for 64% of PGT-M cases. Also, it shows the frequency of most of the disorders studied in PGT-M in the Brazilian population was very similar to the previously reported by the PGD Consortium, but with a higher frequency of polycystic kidney disease and sickle cell anemia in Brazil.

**P-135. Experience report on the effects of smoking and alcoholism on male infertility**

**Ana Beatriz Ferreira de Souza^1^, Beatriz Leite Fileno^1^, Emely Silva de Oliveira^1^, Matheus Silva Martins^1^, Rebeca Aiko Ikeda^1^**

^1^Universidade Nove de Julho (UNINOVE) campus São Bernardo do Campo - São Bernardo do Campo - SP - Brazil

**Objective:** The aim of this study is to report the research experience conducted by a group of medical students on the possible effects of smoking and alcoholism on male infertility, considering that these two factors interfere with the production, quality, and DNA damage of spermatozoa.

**Methods:** Data research was conducted through PubMed and BVS platforms using the MESH/DECS descriptors “Smoking,” “Alcohol,” and “Male infertility.” The aforementioned descriptors were applied on these platforms, combined by the Boolean operator “AND,” resulting in 66 and 71 articles, respectively. Inclusion criteria were publications from the last 5 years that included the chosen keywords in the article theme after a rigorous evaluation. Exclusion criteria were articles that did not cover the target population, did not address smoking and alcoholism concomitantly, and did not use at least semen analysis or DNA fragmentation as parameters to classify infertile men. This resulted in the selection of 16 articles on PubMed and 8 on BVS, with duplicates excluded. Finally, out of the 24 final articles, 4 were carefully chosen for the study.

**Results:** Smoking and alcohol consumption are strongly linked to male infertility. Smoking can cause DNA damage in sperm, reduce its motility, and alter its morphology, as well as affect testosterone production and testicular function due to the toxic substances in cigarettes. Excessive alcohol consumption causes hormonal imbalances, interferes with liver function, and generates free radicals that damage reproductive cells, reducing sperm quality. In Brazil, according to Vigitel (2023), 11.7% of adults are male smokers, and 27.3% have abusive alcohol consumption. Semen analysis was used to classify infertile men into oligozoospermic, azoospermic, and normozoospermic, relating their behaviors regarding tobacco and alcohol. Regular drinking increased the chances of azoospermia, while smoking was more associated with oligozoospermia. All azoospermic men in the study were smokers and consumed alcohol, suggesting an association between lifestyle and male infertility. Various semen parameters and smoking and drinking habits were evaluated. Individuals who smoked or drank regularly and those who did both showed worse outcomes, such as low sperm concentrations and higher chromatin abnormality. In comparison, non-smokers and non-drinkers had more favorable results. An additional study divided participants into heavy smokers, heavy drinkers, non-smokers, and non-drinkers, revealing that semen parameters were significantly better in non-smokers and non-drinkers compared to their counterparts. Protamine deficiency, sperm DNA damage (sDF), and reactive oxygen species (ROS) production were higher among smokers and drinkers, respectively. Notably, alcohol consumption showed a greater impact on sperm maturity and DNA integrity than smoking. Therefore, both smoking and excessive alcohol consumption can significantly impair sperm quality, but alcohol may have a more damaging effect on DNA integrity and sperm maturation.

**Conclusion:** There is a clear link between smoking and excessive alcohol consumption and male infertility. After facing significant challenges in performing comparative analyses due to the divergence of samples and methods used in each article, we conclude that a lifestyle including tobacco and alcohol has a substantial negative influence on male fertility. This behavior can also trigger other pathologies, highlighting the urgent need for further studies to develop effective prevention and treatment methods. These efforts are crucial to address this complex and impactful issue, hence the necessity for more studies.

**P-136. Seminal proteomic evaluation of low and high performance cyclists**

**Luana Nayara Gallego Adami^1^, Jackelinne Yuka^1^, Alexandre Keiji Tashima^1^, Ricardo Pimenta Bertolla^1^**

^1^UNIFESP - São Paulo - SP - Brazil

**The authors did not present the abstract**

**P-137. Progesterone Primed Ovarian Stimulation Protocol (PPOS) using Desogestrel for LH Suppression in Assisted Reproduction cycles: Case series**

**Leci Veiga Caetano Amorim^1^, Patricia Mendonça Leite^2^, Gabriela Boller Bicalho^2^, Erica Becker Sousa Xavier^1^, Ricardo Mello Marinho^1^, João Pedro Junqueira Caetano^1^**

^1^Huntington Procriar - Belo Horizonte - MG - Brazil

^2^Hospital das Clinicas - UFMG - Belo Horizonte - MG - Brazil

**Objective:** This study aimed to evaluate the impacts of using desogestrel (DSG) as a medication for LH surges inhibition during ovarian stimulation cycles in assisted reproduction, specifically analyzing the quantity of dominant follicles, oocytes and mature oocytes retrieved during ovarian puncture.

**Methods:** A retrospective analysis was carried out on the outcomes of using DSG 75mcg once daily for to suppress endogenous LH surges during controlled ovarian stimulation at a private clinic from September 2023 to June 2024. The study assessed the number of mature oocytes retrieved and dominant follicles, defined as those with an average diameter greater than 14 millimeters.

**Results:** Seventy-one patients from a private reproductive clinic were included. We analyzed the use of desogestrel for both *in vitro* fertilization (IVF) procedures and oocyte cryopreservation. The average age of the patients was 36 years. Out of the total cycles, fifty-four were for IVF and seventeen were for elective oocyte cryopreservation. The dosage of desogestrel administered was 75mg once daily for all patients. The average number of counted antral follicles were 14 with 9.9 dominant follicles on average. The dominant follicles were defined as those with an average diameter greater than 14 millimeters. The average number of retrieved oocytes was 11.35 out of which 8.08 were mature. None of the patients experienced spontaneous ovulation, indicating the potential success of this protocol.

**Conclusion:** The use of DSG as a drug of choice in PPOS displayed successful stimulation outcomes in all of our cases. This finding could lead to a new perspective regarding ovarian stimulation, as a once daily oral drug may improve patient experience and potentially reduce procedure costs.

**P-138. Will the same morphokinetic algorithm work in the selection of embryos grown in different culture media?**

**Guilherme Rios Franco^1^, Pedro Henrique Rios Franco^1^, Antonio Carlos Costa Franco^1^, Beatriz Bonini^1^, Victória Christine Carvalho de Oliveira^1^, Vanessa Mesquita Prada^2^, Beatriz Müller Nunes Souza^1^, Giovanna Dente Stella^1^**

^1^Embryolife Instituto de Medicina Reprodutiva - São José dos Campos - SP - Brazil

^2^Fecondare - Florianópolis - SC - Brazil

**Objective:** To compare the same morphokinetic configurations algorithm used in embryos cultured in two different media, one sequential and the other uninterrupted.

**Methods:** 517 embryos were divided into two groups of different culture media. The first group of sequential medium with 111 embryos and the second group of uninterrupted medium with 406 embryos. The morphokinetics of the cleavages of each embryo were studied using Time Lapse technology. Tukey's statistical test was used to compare results between groups.

**Results:** The rate of embryos that fit the morphokinetic curve in the sequential group was 40.5%, while in the uninterrupted group it was 29.1%. The Tukey test revealed this difference as significant. The pregnancy rate was 48% in the sequential group and 49% in the uninterrupted group.

**Conclusion:** The same algorithm used in embryonic selection to define an ideal morphokinetic curve does not seem to work when using different culture media. Our group suggests a model for adjusting and validating an algorithm in this situation.

**P-139. Does low concentration of Anti-Mullerian Hormone influence *In Vitro* Fertilization outcomes in young patients?**

**Janaína Jardelha Mendes Maciel^1^, Lívia Santos Souza^1^, Tassia Souza Leão Silva^1^, Gérsia Araújo Viana^1^**

^1^Cenafert - Salvador - BA - Brazil

**Objective:** To verify if young patients with low concentrations of AMH produce lower quality oocytes/embryos compared to patients of the same age group with normal AMH concentrations.

**Methods:** Retrospective study with analysis of archived records conducted at a private Assisted Reproduction clinic. Included were young patients up to 35 years old with anti-Müllerian hormone levels below 1.1 ng/mL, who underwent *in vitro* fertilization between May 2019 and September 2021. The control group consisted of patients in the same age range but with anti-Müllerian hormone levels starting from 1.1 ng/mL, who also underwent IVF in the same period. The criteria to be evaluated in this study are: number of eggs collected, number of mature eggs (MII), fertilization rate; cleavage rate, blastulation rate, and embryonic quality (top quality). Data analysis will utilize the Mann-Whitman test.

**Results:** The results demonstrate that there was a statistically significant difference for the variables: eggs retrieved (*p*=0.001), mature eggs (*p*=0.002), eggs inseminated (*p*=0.003), fertilized eggs (*p*=0.003), and cleaved eggs (*p*=0.004); however, there was no statistical difference for the blastulation rate (blastocysts) and top quality blastocysts.

**Conclusion:** The results obtained in this study demonstrate that young patients with low concentrations of AMH do not produce lower quality oocytes/embryos compared to patients of the same age group who have normal AMH concentrations.

**P-140. Correlation between serum Vitamin D levels, euploid blastocyst rate and fertilization rate in *In Vitro* Fertilization (IVF)treatments**

**Ana Cristina Allemand Mancebo^1^, Brunna Stumpo Vaz^1^, Marcelo Marinho Souza^1^, Maria do Carmo Borges de Souza^1^, Roberto de Azevedo Antunes^1^, Verônica de Almeida Raupp^1^, Ana Luísa Bruno Marinho Souza^1^, Flávia Fernandes Sequeira^1^, Layna Almeida Barbosa Silva^1^, Ana Luíza de Oliveira Barbeitas^1^**

^1^Fertipraxis - Rio de Janeiro - RJ - Brazil

**Objective:** Infertility is a complex condition influenced by various factors, including male and female causes, and environmental factors. The presence of VD receptors in different reproductive system sites is well-documented. Arnanz et al. (2021) identified a significant correlation between sufficient VD levels in blood and follicular fluid and increased chances of obtaining euploid blastocysts. However, existing literature data remain contradictory regarding the establishment of this correlation with embryonic status outcomes and improved birth rates. The present study investigated the potential association between serum vitamin D levels and the rates of euploid blastocysts per biopsied blastocyst, as well as fertilization per M2 oocyte, in couples undergoing *in vitro* fertilization treatments.

**Method:** This study adopts a retrospective cross-sectional observational design includind 210 couples who underwent ICSI with PGT-A analysis from January 2017 to December 2021. Women's ages ranged from ≥35 to ≤40 years and men's ages ≤55 years. Couples were divided into 4 groups: G1 (33 couples): both with VD ≥30 ng/mL; G2 (78): both with VD < 30 ng/mL; G3 (46): women with VD<30 ng/mL and men ≥30 ng/mL and G4 (53): women with VD ≥30 ng/mL and men <30 ng/mL. All participants signed an informed consent form. Demographic parameters (age, BMI, AMH and vitamin D levels) as well as number of oocytes retrieved, M2 oocytes, fertilization rate, number of blastocysts, and euploid blastocysts rate were evaluated.

**Results:** A Generalized Linear Model (GLM) was employed to assess the influence of Vitamin D on euploid blastocysts rate (EBR) and fertilization rate (FR). All analyses were controlled by the couple's age and BMI, and anti-Mullerian hormone (AMH) levels. The outcomes studied were described by the mean and standard deviation as follows: G1 EBR 48.82 (±37.82) / FR 76.93 (±22.56), G2 EBR 51.38 (±38.41) / FR 78.82 (±21.13), G3 EBR 53.99 (±34.26) / FR 75.76 (±25.68), and G4 EBR 42.07 (±33.02) / FR 72.64 (±28.30). The overall Chi-square of the models had a p-value greater than 0.05, indicating that Vitamin D did not emerge as a predictor variable for the rates of euploid blastocysts per biopsied blastocysts (*p*=0.179) and fertilization per M2 oocyte (*p*=0.256).

**Conclusion:** We did not observe a causal relationship between the variables studied. Perhaps, the complexity of the subject requires new and innovative approaches in future research, with different designs, or even considering a different cut-off point for VD. Future studies, larger sample sizes, and a comprehensive analysis of other variables involved may contribute to a more complete understanding of the complex relationship between vitamin D and embryonic quality.

**P-141. Artificial Intelligence (AI) vs. Gardner's Classification in *In Vitro* Fertilization (IVF)**

**Lucas Nazareth Remidio de Carvalho^1^, Geisiane Alves do Nascimento^1^, Hingrid Alves da Silva^1^, Ludmila Terra Borges^1^, Luiz Augusto Teixeira Batista^1^, Luiz Augusto Antônio Batista^1^, Nathália Teixeira Batista^1^, Ricardo Novato Pimentel^1^**

^1^Perfetto Reprodução Humana - Goiânia - GO - Brazil

**Objective:** To compare the average accuracy of embryo assessment using Gardner's classification system with the accuracy of the AI model (Embryo Management Assisted, EMA) in predicting pregnancy outcomes.

**Methods:** A retrospective cross-sectional study based on data from patients undergoing *In Vitro* Fertilization (IVF) and embryo transfer between August 2023 and April 2024. Cycles were stratified into five maternal age groups according to Society for Assisted Reproductive Technology guidelines. An automated Artificial Intelligence (AI) model and the morphological classification model (Gardner's criteria) were used to correlate the data.

**Results:** Forty-seven patients were analyzed. In the <35 years age group, there was a relatively high average EMA score, indicating good morphological quality of embryos. The standard deviation of 15.61 suggests moderate variability in EMA results. Most embryos were of high quality morphological blastocyst (5AA and 4AA), potentially contributing to higher rates of positive beta tests. For the 35-37 years age group, the EMA average was slightly lower compared to the younger group, indicating slightly inferior embryo quality. The standard deviation was similar, suggesting consistency in result variability. Presence of high-quality high morphological blastocyst (5AA and 4AA)was positive, although there was one cycle with a negative beta, indicating potential variability in success outcomes. In the 38-40 years age group, the EMA average was slightly higher than the 35-37 years group, but a higher standard deviation (21.37) indicates greater variability in EMA results. Presence of high-quality embryos is significant, but the larger standard deviation suggests a wide range of outcomes among cycles. For the 41-42 years age group, the EMA average is comparable to the <35 years group, suggesting relatively good morphological quality of embryos. The standard deviation of 16.28 indicates moderate variability in EMA results. The small sample size (4 cycles) may limit generalizations, but the absence of cycles with negative betas is encouraging. In the >42 years age group, the EMA average is the lowest among all groups, reflecting potentially lower morphological quality of embryos. The smaller standard deviation (11.75) suggests narrower variability in EMA results despite the small sample size. Presence of high-quality embryos is limited, which may contribute to less favorable outcomes.

**Conclusion:** Younger groups, especially those under 35 years old, demonstrated higher EMA scores on average and a significantly higher prevalence of high-quality embryos such as 5AA and 4AA blastocysts. This suggests that younger women have a more favorable response to IVF treatments in terms of embryo quality, potentially positively impacting reproductive success rates. The presence of cycles with negative beta results in some groups, even when high-quality embryos were present, highlights the complexity and variability in IVF outcomes where factors beyond embryo quality may influence clinical outcomes. The analysis also reveals a consistent trend in older groups (41-42 years and above 42 years), which generally showed lower EMA averages and a lower proportion of high-quality embryos such as 5AA and 4AA blastocysts. These findings align with clinical expectations of decreased ovarian reserve and oocyte quality with advancing maternal age, potentially negatively impacting IVF success rates in these age groups.

**P-142. Longer stimulation ICSI cycles and laboratory outcomes**

**Victória Campos Dornelles^1^, Marta Ribeiro Hentschke^1^, Fabiana Mariani Wingert^1^, Vanessa Devens Trindade^1^, Natalia Fontoura Vasconcelos^1^, Isadora Badalotti-Teloken^1^, Adriana Arent^1^, Alvaro Petracco^1^, Mariangela Badalotti^1^**

^1^Fertilitat - Centro de Medicina Reprodutiva - Porto Alegre - RS - Brazil

**Objective:** To evaluate if a longer stimulation is related to worse laboratory outcomes in assisted reproduction.

**Methods:** Retrospective, observational study performed at a reproductive medicine center, including 2542 ICSI cycles between 2019-2023. Cycles with no oocytes recovered, heterologous or donor ejaculated semen and ≥ 42 years old female patients were excluded. Sample was divided into 3 groups, according to oocyte retrieval day: G1 (9^th^ to 12^th^; n=572), G2 (13^th^ to 14^th^; n=1290) and G3 (15^th^ to 21^st^; n=680). Variables analyzed were: female and male age, total and mature oocyte number, oocyte maturity rate, fertilization and blastulation rates. Variables were compared between groups and expressed as mean±SD and n (%). Anova, post-hoc curve and chi-square test were applied (*p*<0.05).

**Results:** When comparing G1 *vs*. G2 *vs*. G3, the following results were found, respectively: female age, yo (36.61±3.72^a^ *vs*. 36.39±3.96^b^ *vs.* 37.28±3.46^c^. *p*<0.001^c^); male age, yo (38.86±6.11^a^ *vs.* 38.62±5.47^b^ *vs*. 39.37±5.32^c^ *p*=0.020^bc^); total oocyte number (10.66±7.89^a^ *vs.* 12.07±8.58^b^ *vs*. 9.34±6.77^c^ *p*<0.001^abc^); mature oocyte number (7.63±5.74^a^ *vs*. 9.00±6.71^b^ *vs*. 7.18±5.50^c^, *p*<0.001^b^); oocyte maturity rate, % (74.0^a^ *vs*. 75.9^b^ *vs*. 78.7^c^, *p*<0.001^c^), fertilization rate, % (78.1 *vs*. 78.8 *vs*. 77.4, *p*=0.256) and blastulation rate, % (41.9^a^ *vs*. 46.7^b^ *vs*. 41.3^c^, *p*<0.001^bc^).

**Conclusion:** The oocyte maturity rate was higher in the longer stimulation cycle group, without influencing fertilization rates between groups. However, the blastulation rate was statistically lower in the longer stimulation group (15^th^ to 21^st^), when compared to 13^th^ to 14^th^ retrieved days, without difference regarding the shortest stimulation group. It is important to highlight that longer stimulation even with better oocyte maturity rate, may be related to worse laboratory outcomes in assisted reproduction. Conversely, 13^th^ to 14^th^ retrieved days oocytes could be related to better laboratory results. Finally, for data interpretation purposes, it must be reinforced that the female and male age in the group with the longest stimulation time was higher, which may also have contributed to these findings.

**P-143. Artificial Intelligence (AI) in *In Vitro* Fertilization (IVF): A simplified analysis from an Assisted Human Reproduction center**

**Lucas Nazareth Remidio de Carvalho^1^, Geisiane Alves do Nascimento^1^, Hingrid Alves da Silva^1^, Ludmila Terra Borges^1^, Luiz Augusto Teixeira Batista^1^, Luiz Augusto Antônio Batista^1^, Nathália Teixeira Batista^1^, Ricardo Novato Pimentel^1^**

^1^Perfetto Reprodução Humana - Goiânia - GO - Brazil

**Objective:** This study aimed to analyze the performance of EMA (Embryo Management Assisted), a deep learning-based artificial intelligence, in predicting clinical pregnancy outcomes in *in vitro* fertilization (IVF) procedures.

**Methods:** This was a retrospective cross-sectional study conducted between August 2023 and April 2024. Data were derived from electronic records of patients undergoing IVF and embryo transfer. Cycles were stratified into five maternal age groups (<35, 35-37, 38-40, 41-42, and >42 years). EMA, an AI-driven automated model trained on time-lapse embryo images, known morphology, and clinical pregnancy data, was used. EMA scores range from 1.0 to 9.9, reflecting the probability of clinical pregnancy, with 9.9 indicating the highest embryo viability confidence and 1.0 the lowest.

**Results:** The results revealed variations in mean EMA scores and standard deviations across maternal age groups: For women under 35 years, the mean EMA was 76, with a standard deviation of 15.61. There were no cases of miscarriage or non-viable embryos. In the 35-37 age group, the mean EMA was 69, with a standard deviation of 15.62. One miscarriage and one non-viable embryo case were observed. Women aged 38-40 had a mean EMA of 71, with a standard deviation of 21.37. One miscarriage was recorded. For women aged 41-42, the mean EMA was 76, with a standard deviation of 16.28, and no cases of miscarriage or non-viable embryos were reported. Above 42 years, the mean EMA was 55, with a standard deviation of 11.75, and there were no occurrences of miscarriage or non-viable embryos.

**Conclusion:** The study demonstrated that EMA exhibits significant accuracy in predicting clinical pregnancy outcomes in IVF, as evidenced by the scores assigned to embryos. The results highlight EMA's capability to provide a more reliable estimate of embryo viability compared to traditional human assessment methods. The integration of time-lapse embryo imaging with clinical information further enhanced the model's accuracy, underscoring its potential as a valuable complementary tool for clinical embryologists. This advancement could significantly contribute to improving outcomes in IVF treatments, offering a more precise and predictive approach in selecting viable embryos for transfer, thus benefiting clinical practice and patient outcomes.

**P-144. Meta-analysis: Effects of smoking on semen quality**

**Isadora de Souza^1^, Hanin El Husseini^1^, Ricardo Omizzolo^1^, Paulina Alejandra Santander-Perez^2^**

^1^Universidade Positivo - Curitiba - PR - Brazil

^2^Pontifícia Universidade Católica/ PR - Curitiba - PR - Brazil

**Objective:** This meta-analysis aims to consolidate and evaluate the existing literature on the effects of cigarette smoking on semen quality. Specifically, it seeks to determine the impact of smoking on various semen parameters including sperm concentration, motility, morphology, and DNA integrity.

**Methods:** To gather information for this meta-analysis, a thorough review of scientific literature was conducted using reliable databases such as PubMed, Google Scholar, and the National Center for Biotechnology Information (NCBI). Keywords used in the search included "smoking," "sperm DNA," "male fertility," "genetic damage," and "oxidative stress." A total of 58 studies met the inclusion criteria and were included in the meta-analysis. Among these, 10 Studies were selected for the comparison based on their relevance, sample size, and publication in peer-reviewed journals. The analysis focused on clinical trials, observational studies, and meta-analyses published within the last decade.

**Results:** The analysis of the selected studies revealed consistent evidence that smoking adversely affects semen quality. Sperm Concentration: On average, smokers have a sperm concentration of 71.0 million sperm per milliliter compared to 92.3 million sperm per milliliter in non-smokers. This indicates that smokers have approximately 23.1% fewer sperm per milliliter than non-smokers. Sperm Motility: Smokers exhibit an average sperm motility of 36.0%, whereas non-smokers have an average motility of 50.3%. This means that smokers have about 28.4% lower sperm motility compared to non-smokers. Sperm motility is crucial as it reflects the ability of sperm to move efficiently, which is essential for fertilization. Normal Morphology: The percentage of sperm with normal morphology in smokers is 3.9%, compared to 6.1% in non-smokers. This shows that smokers have 36.1% fewer normally shaped sperm, which is important for successful fertilization. DNA Fragmentation Index: Smokers have an average DNA fragmentation index of 21.4%, while non-smokers have an index of 13.4%. This indicates that smokers have 59.7% more DNA fragmentation, signifying higher genetic damage within the sperm.

**Conclusion:** The cumulative evidence from multiple studies clearly demonstrates that smoking has a detrimental effect on semen quality. Smokers exhibit reduced sperm concentration, motility, and normal morphology, along with increased DNA fragmentation. These findings suggest that smoking can significantly impair male fertility, highlighting the need for public health interventions to reduce smoking rates among men of reproductive age. The implications of these findings are critical for couples trying to conceive, as well as for healthcare providers who should advise smoking cessation as part of fertility treatment protocols.

**P-145. Implications of low-grade mosaic embryo transfer: To transfer or not to transfer?**

**Sarah Louredo Torquette^1^, Luma Soares Fagundes^1^, Paula Santos Coelho^1^, Ana Márcia de Miranda Cota^1^**

^1^Faculdade de Ciências Médicas de Minas Gerais - Belo Horizonte - MG - Brazil

**Objective:** The main objective of this research was to evaluate whether or not low-grade embryo transfer should be recommended, analyzing its implications, according to the most recent evidence.

**Methods:** This study is based on a literature review of scientific productions published between 2019 and 2024. The Health Sciences Descriptors (DeCS) "Mosaicism", "Preimplantation Diagnosis" and "Embryo Transfer" were applied to the PubMed database, joined to the Boolean operator "AND". Initially, 119 studies were found. Of these, 68 were excluded because they had not been published in the last 5 years, so that 51 publications were analyzed and after filtering by title and abstract, 14 relevant studies remained that were included in this work.

**Results:** A common finding of preimplantation genetic testing for aneuploidy (PGT-A) is the mosaic embryo (ME). Mosaicism can be classified according to the percentage of affected cells, being considered low grade when < 40-50% (no defined consensus). Due to the lack of knowledge about the impacts related to different degrees of mosaicism, the decision about transferring this type of embryo is a challenge, especially when there are no chromosomally normal embryos available. Studies show that the level of mosaicism influences outcomes. It has been observed that pregnancy and live birth rates are lower when transferring MEs compared to euploid embryos, ranging from 16% to 47%, with rates being lower in high-grade MEs. Research that stratified 1,000 ME and analyzed how the level of mosaicism affected clinical outcomes contradicts claims that mosaicism is merely an artifact of PGT-A, as low-grade MEs have poorer outcomes than euploids, although often have positive results. A single-cell sequencing study analyzed 1,616 blood cells from 7 babies and found no chromosomal changes similar to those seen in trophectoderm biopsy, suggesting that embryonic chromosomal mosaicism was corrected. Low percentages of aneuploid cells in blastocyst embryos do not appear to affect the growth and development of babies. Two hypotheses are suggested: an embryonic self-healing mechanism, where aneuploid cells are eliminated, and the possibility that the trophectoderm biopsy does not completely represent the inner cell mass of the blastocyst. Some studies have concluded that transfer of low-grade MEs can be done without additional risk of worse outcomes. Current evidence shows that low-grade MEs should not be systematically discarded or disregarded for transfer. When considering transfer of a low-grade embryo, it was suggested: evaluate PGT-A results along with embryo morphology; provide non-directive genetic counseling to discuss possibilities and limitations; that the possibility of performing a new stimulation cycle can be considered; and a shared decision should be made. There is a lack of evidence regarding the benefit of prenatal confirmation after transfer.

**Conclusion:** Due to the lack of knowledge about the different degrees of mosaicism, making clinical decisions about transferring these embryos is challenging. However, transfer of low-grade MEs appears to be a relatively safe option, with low risk of negative birth outcomes, so they can be considered for transfer.

**P-146. Artificial Intelligence and mental health of Assisted Reproduction patients**

**Ana Paula Estevam Melo Pimentel^1^, Gabriela Batista Machado^1^, Lucas Nazareth Remidio de Carvalho^1^**

^1^Perfetto Reprodução Humana - Goiânia - GO - Brazil

**Objective:** The psychological impact on patients undergoing assisted reproduction can be significant due to the emotional complexity and expectations inherent in the process. Artificial intelligence (AI) is beginning to play a pivotal role in this field, offering both opportunities and additional challenges. The objective is to conduct a literature review analyzing the impact of artificial intelligence on the mental health of assisted reproduction patients.

**Methods:** This narrative review was conducted using all articles available up to May 2024 in PubMed. Search terms included artificial intelligence, mental health, assisted reproductive technology, and reproductive medicine in various combinations. Sources not specifically focusing on AI applications in reproductive care were excluded. Additional sources included psychoanalytical literature examining the effects of AI on contemporary subjectivity construction.

**Results:** Eight articles were selected detailing primary AI applications in assisted reproductive treatments (ART). Findings demonstrate that artificial intelligence (AI) has emerged as a promising tool in assisted reproductive medicine (ART), offering significant advances in optimizing treatment outcomes such as *in vitro* fertilization (IVF). AI facilitates monitoring of follicular development via ultrasound, evaluation of endometrial receptivity, and prediction of pregnancy outcomes following embryo transfer. These applications enable more precise and personalized assessment of female reproductive function, thereby improving the timing of embryo transfer and enhancing success rates. Moreover, AI has been successfully employed in the selection of embryos and sperm based on criteria like morphokinetic viability through algorithms and deep learning, identifying the most promising embryos and significantly increasing the chances of successful pregnancy within shorter timeframes. This approach not only enhances clinical outcomes but also tailors IVF protocols by integrating detailed data such as omics (metabolomics, transcriptomics) and biomarkers to provide more tailored options aligned with individual patient needs. A critical issue raised within the context of AI is its impact on the mental health of patients undergoing assisted reproductive treatments and utilizing AI-generated data. Patients often struggle with the interpretation of the abundant information provided by medical teams, leading to conditions resulting from the inability to interpret such results. The question facing assisted reproduction patients pertains to the interpretation of AI-generated results amidst their subjectivity. Therefore, the proposed approach necessitates a multidisciplinary team, wherein psychology and psychoanalysis can assist patients in interpreting AI-generated data, aiming to mitigate distress or impediments to continuing assisted reproduction treatment.

**Conclusion:** The use of AI also raises ethical and psychological concerns. Excessive reliance on algorithms may depersonalize the medical experience and diminish the importance of human support during the emotionally challenging process of assisted reproduction. For some patients, AI usage may heighten anxiety, particularly if there is an excessive dependency on predictive results or if expectations are not met. Therefore, it is crucial for AI applications to be complemented by a multidisciplinary team approach, with specific attention from psychology professionals.

**P-147. Systematic review on the association between pesticides and male infertility**

**Isadora de Souza^1^, Hanin El Husseini^1^, Ricardo Omizzolo^1^, Paulina Alejandra Santander-Perez^2^**

^1^Universidade Positivo - Curitiba - PR - Brazil

^2^Pontifícia Universidade Católica/ PR - Curitiba - PR - Brazil

**Objective:** Male infertility is a significant public health concern, with growing evidence linking environmental factors, particularly pesticide exposure, to declining semen quality. Pesticides, including organophosphates, pyrethroids, and various herbicides, are known to act as endocrine-disrupting chemicals, potentially impairing male reproductive health. This systematic review aims to synthesize the current evidence on the impact of pesticide exposure on male infertility, focusing on key semen parameters such as sperm concentration, motility, morphology, and DNA integrity.

**Methods:** This review followed the Preferred Reporting Items for Systematic Reviews and Meta-Analyses (PRISMA) guidelines. A comprehensive literature search was conducted using PubMed, Scopus, and Web of Science databases. The search terms included "pesticides," "sperm quality," "male infertility," "semen parameters," "DNA integrity.” The inclusion criteria were: (1) observational studies assessing the relationship between pesticide exposure and sperm quality in humans, (2) studies published in English, and (3) studies providing quantitative data on semen parameters.

**Results:** The review of the selected studies revealed that pesticide exposure is significantly associated with reductions in sperm quality across various parameters. A majority of the studies reported that exposure to pesticides, including organophosphates, pyrethroids, and herbicides like atrazine, is linked to decreased sperm concentration, motility, and morphology, as well as increased DNA fragmentation. These adverse effects were consistent across different study designs and populations. For instance, organophosphate exposure was frequently associated with reductions in sperm count and motility, while pyrethroids significantly affected sperm morphology and DNA integrity. The studies also highlighted the role of genetic polymorphisms in modulating the susceptibility to these effects. Overall, the evidence strongly supports that pesticide exposure at environmentally or occupationally relevant levels detrimentally impacts male reproductive health.

**Conclusion:** The consolidated findings from the reviewed studies indicate a clear association between pesticide exposure and impaired male reproductive health. Pesticides such as organophosphates, pyrethroids, and atrazine have been consistently linked to reduced sperm quality parameters, including sperm concentration, motility, and morphology, as well as increased DNA fragmentation. These results underscore the need for regulatory actions to mitigate pesticide exposure and its adverse effects on male fertility. Further research should focus on specific pesticides and their mechanisms of action to develop targeted interventions for protecting male reproductive health.

**P-148. Impact of the presence of blood on the catheter and guide in embryo transfers**

**Jonathan Boncristiano^1^, Emilio Costa Garavini^1^, Bernardo Rodrigues Lamounier Moura^1^, Aline Aquino^1^**

^1^Gerar in Vitro - Belo Horizonte - MG - Brazil

**Objective:** To compare the rates of ßhCG and clinical pregnancy in embryo transfers with the presence of blood on the catheter and guide, stratifying the results by age group.

**Methods:** During the study, 738 embryo transfers with thawed embryos were performed between January 2021 and March 2024. Participants were stratified into age groups: <35, 35-39, 40-42, and >42 years. Oocytes were fertilized via Intracytoplasmic Sperm Injection (ICSI). A soft-tipped catheter and guide were used for the transfer. Results were recorded and analyzed using the Chi-Square test for comparison.

**Results:** No statistically significant differences were observed in ßhCG and clinical pregnancy rates between the groups with and without blood on the guide (ßhCG: 37.2% *vs*. 40.2%, *p*=0.410; Clinical Pregnancy: 24.4% *vs*. 25.4%, *p*=0.752). Similarly, ßhCG and clinical pregnancy rates did not differ significantly between the groups with and without blood on the catheter (ßhCG: 38.7% *vs*. 39.2%, *p*=0.890; Clinical Pregnancy: 24.7% *vs*. 25.2%, *p*=0.893). In the >35 age group, no statistically significant differences were observed in ßhCG and clinical pregnancy rates between the groups with and without blood on the guide (ßhCG: 43.9% *vs*. 42.2%, *p*=0.783; Clinical Pregnancy: 33.0% *vs*. 27.8%, *p*=0.384). Similarly, ßhCG and clinical pregnancy rates did not differ significantly between the groups with and without blood on the catheter (ßhCG: 42.7% *vs*. 42.8%, *p*=0.993; Clinical Pregnancy: 32.0% *vs*. 28.0%, *p*=0.487). In the 35 to 39 age group, no statistically significant differences were observed in ßhCG and clinical pregnancy rates between the groups with and without blood on the guide (ßhCG: 39.7% *vs*. 39.4%, *p*=0.967; Clinical Pregnancy: 24.5% *vs*. 21.0%, p=0.520). Similarly, ßhCG and clinical pregnancy rates did not differ significantly between the groups with and without blood on the catheter (ßhCG: 39.3% *vs*. 39.7%, *p*=0.951; Clinical Pregnancy: 21.7% *vs*. 23.4%, *p*=0.760). In the 40 to 42 age group, no statistically significant differences were observed in ßhCG and clinical pregnancy rates between the groups with and without blood on the guide (ßhCG: 23.3% *vs*. 38.2%, *p*=0.101; Clinical Pregnancy: 16.7% *vs*. 27.3%, *p*=0.203). Similarly, ßhCG and clinical pregnancy rates did not differ significantly between the groups with and without blood on the catheter (ßhCG: 28.2% *vs*. 34.7%, *p*=0.484; Clinical Pregnancy: 18.4% *vs*. 25.7%, *p*=0.391). However, a trend is observed from this age group onwards, where groups with blood on both the catheter and the guide showed lower results. In the group aged over 42 years, no statistically significant differences were observed in ßhCG and clinical pregnancy rates between the groups with and without blood on the guide (ßhCG: 26.5% *vs*. 37.7%, *p*=0.277; Clinical Pregnancy: 9.7% *vs*. 25.5%, *p*=0.082). Similarly, ßhCG and clinical pregnancy rates did not differ significantly between the groups with and without blood on the catheter (ßhCG: 35.5% *vs*. 32.1%, *p*=0.752; Clinical Pregnancy: 17.9% *vs*. 20.0%, *p*=0.818). However, the trend continues to be observed in groups with blood, indicating a possible negative influence in this age group.

**Conclusion:** No statistically significant differences were observed in ßhCG and clinical pregnancy rates between the groups with and without blood on the guide or the catheter. However, a trend of lower results remains in the groups with the presence of blood, particularly in patients of advanced age, starting from 40 years old.

**P-149. Impact of Zymot sperm selection yechnique on fertilization and blastulation rates**

**Amanda Cerquearo Rodrigues dos Santos^1^, Luiz Mauro Oliveira Gomes^1^, Leticia Emi Kadomoto Ferreira^1^, José Fernando de Macedo^1^, Gustavo Capinzaiki de Macedo^1^, Daniela Oliveira Gomes^1^, Thaís Freire Cardoso^1^**

^1^Reproferty - Centro de Reprodução Humana - São José dos Campos - SP - Brazil

**Objective:** This study aimed to evaluate the efficacy of the Zymot sperm selection technique on fertilization and embryo development rates in men with high and low levels of sperm fragmentation, to determine if this technology significantly improves outcomes in assisted reproduction treatments for both groups.

**Methods:** This retrospective study was conducted using data from patients at a single center who underwent *in vitro* fertilization (IVF) treatment in 2023. Inclusion criteria encompassed patients, spouses, or semen donors who had undergone a sperm DNA fragmentation test within six months prior to the fertilization date. Patients who did not meet the established criteria and objectives of this study were excluded. Patients were classified based on the degree of fragmentation into "High Fragmentation" (≥ 20%) or "Low Fragmentation" (< 20%) and based on the semen preparation technique used, divided into “Zymot” or “Other Preparation Techniques” (Swim-up, Sperm-wash, or Density Gradient Centrifugation). Fertilization and blastulation rates for each cycle were calculated, and data analysis was performed using GraphPad Prism 5. Percentages were calculated, and statistical analysis was conducted. Different groups were analyzed using the column statistics normality test and the Shapiro-Wilk test, followed by the Mann-Whitney test, with a significance level of 95% (p≤0.05).

**Results:** During the study period, 112 patients who underwent IVF treatment and met the inclusion criteria were analyzed. Of these, 55% were classified as having "Low Fragmentation". Among these, 35% used "Other Preparation Techniques", achieving fertilization rates of 90% and blastulation rates of 61%, while approximately 20% were prepared with “Zymot”, achieving fertilization rates of 91% and blastulation rates of 69%. Although the use of the microfluidic system showed a slight improvement in fertilization and blastulation rates in patients with low fragmentation, the values were not statistically significant. The study by Taylor et al. (2021) suggests that there is no difference in fertilization rates, blastocyst development, and euploidy between semen samples prepared with density gradient centrifugation and those prepared with Zymot. However, the euploidy rates of blastocysts from semen samples prepared with Zymot are slightly higher, though this difference is not statistically significant. Of the patients, 45% were classified as having "High Fragmentation". Within this group, 19% used "Other Preparation Techniques", achieving fertilization rates of 77% and blastulation rates of 48%, while approximately 26% were prepared with “Zymot”, resulting in fertilization rates of 84% and blastulation rates of 61%. Similar to the low fragmentation group, an improvement in fertilization and blastulation rates was observed with the “Zymot” preparation technique, although this difference was not statistically significant. These findings are consistent with the study by Rydze et al. (2023), which also analyzed semen samples from patients with high fragmentation processed with and without Zymot and found no statistically significant difference in blastocyst formation rates when using the microfluidic system.

**Conclusion:** The Zymot sperm selection technique proved effective in increasing the percentages of fertilization and blastulation rates. However, there was no statistically significant difference in fertilization and blastulation rates when the microfluidic system was used for sperm selection in either group.

**P-150. Psychic aspects of being a mother for women receiving eggs: A proposal for psychoanalytic listening**

**Patricia Cristina Souza Assis Dummer^1^, Cristielli Rosa e Silva Souza^1^**

^1^Unifert- Centro Avançado de RH - Vila Velha - ES - Brazil

**Objective:** Faced with the impossibility of becoming a mother with her own eggs, women seeking assisted reproduction treatment are faced with a moment of extreme sensitivity that involves a change in reproductive paradigm when resorting to egg donation as a treatment that allows them a greater probability of gestation.

**Methods:** This article reports on the experience of multidisciplinary care in an assisted reproduction clinic, located in Greater Vitória/ES. This is a study that aims to present the different psychological aspects awakened by the impossibility of childbearing in infertile women in treatment in an Assisted Reproduction clinic, participants in the Ovodoreception Program, registered from July 2023 to June 2024, and guarantee humanized assistance through psychological listening and nursing support. The intervention was carried out through psychoanalytic listening to women referred for egg reception, calling on them to express their desire to be a mother and the path taken from the subjective constitution to the construction of the internalization of motherhood. The reception was carried out preliminarily by the team nurse.

**Results:** Psychological care for egg recipient patients is carried out through referral from the responsible doctor when the following situations occur: after evaluation of hormonal tests and ultrasound to count antral follicles, when low ovarian reserve is identified through hormonal tests, especially anti-Mullerian; after the patient undergoes ovarian stimulation without obtaining results of follicle development before oocyte collection; when there is an absence of oocytes on the day of ovarian aspiration, or in cases of chromosomal changes in embryos submitted to genetic analysis. These women arrived for care “ungrounded” (sic), with their dreams and expectations shattered, without understanding or knowing the possibility of having children through the fertilization of eggs from a younger woman. Among the most varied and complex issues presented is the renunciation of genetic affiliation imposed by infertility. The fear of not recognizing himself in his own son was present in the speeches. On the other hand, imagining herself without children seemed to be more painful than dealing with her fears and fantasies. Furthermore, many of these women had a huge desire to go through all the gestational stages, to be seen pregnant and to be able to feel capable and complete as a woman. Despite changes in the social role of women, in the socio-cultural context the overvaluation of maternal identity is still notable, projecting the responsibility for generating offspring onto women. With regard to the disruption of genetic inheritance and the concern that the child would not present their phenotypic characteristics, issues that supported the desire to be a mother and the relationship between epigenetics and the reception of eggs were addressed. The pregnant mother influences how and who the child will be. During pregnancy, everything that occurs influences the activation or inactivation of the future baby's genes. In the womb there will be an exchange of nutrients, hormones, emotions and the bond of affection established between mother and baby.

**Conclusion:** Given the speeches presented, we analyzed that the impossibility of affiliation brings psychic marks that need to be welcomed, which highlights the great importance of listening and collection work in assisted human reproduction clinics. Opening a listening space allows the appropriation of the desire to have a child and its meaning to be brought by patients during treatment in a way that allows conscious and unconscious conflicts, frustration, pain, expectations and many others to be worked out. associated aspects.

**P-151. Effects of time of exposure to PRP on motility of normal and oligoasthenospermic semen samples. Preliminary results**

**Adriana Bos-Mikich^1^, Gabriella Mamede Andrade^2^, Norma P Oliveira^2^, Giovanna Gonçalves^2^, Carina Saviello^3^, Fernanda I Iglesias^3^, Nilo Frantz^2^**

^1^ Universidade Federal do Rio Grande do Sul - Porto Alegre - RS - Brazil

^2^ Nilo Frantz Medicina Reprodutiva - Porto Alegre - RS - Brazil

^3^ RDO Diagnosticos Medicos - Porto Alegre - RS - Brazil

**Objective:** PRP is a concentrate of platelets containing a wide range of cytokines and growth factors that promotes tissue regeneration and healing. It has been used in different medical specialities, including assisted reproduction to improve endometrial receptivity. The aim of the present study is to analyze the effect of PRP added to normal and oligoasthenospermic semen samples.

**Methods:** Six IVF patients donated one semen and one blood sample after signing an informed consent form. Following the initial examination to access sperm concentration and progressive motility (PM, grades A + B), semen was classified as oligoasthenospermic or normospermic and processed by density gradient centrifugation (Isolate^®^). The pellet was washed and the final pellet was deposited in a centrifuge tube and cultured (MHM supplemented with 10% SSS) for 2hrs, at room temperature. PRP was obtained from a sample of patients´ blood (35 ml) drawn with the addition of anticoagulant. After centrifugation using a “soft” spin (1500 rpm), the supernatant was transferred to another tube with 2 mg/ml gentamicin and centrifuged at a higher speed (2300 rpm). The supernatant was removed leaving 2 ml at the bottom of the tube to suspend the platelet pellet. A fraction of the final PRP preparation was saved for platelet counts. A total of 25 µl of the final PRP preparation was added to the tubes containing 500 µl of sperm preparation, for a final platelet concentration of 5%.

**Results:** Sperm concentration in the oligoasthenospermic samples were 1.2, 5, and 6.6x106sperm/ml. The normospermic samples presented sperm concentrations of 27, 33 and 40x106sperm/ml. PM of raw oligoasthenospermic samples were 8%,10% and 20%. Normospermic samples presented initial PM values of 47%, 27% and 41%. In comparison to the raw samples, PRP exposure for 2hrs increased PM in all cases. The three oligoasthenospermic samples showed increased percentages of PM to 20, 50 and 33%, respectively. Compared to the raw samples, the three normospermic samples presented higher PM values of 61, 46 and 51%, respectively. Among the oligoasthenospermic samples, the major increment in PM occurred in Grade B sperm motility, that is, they moved forward, but not in a straight line. Among the normospermic samples there were significant increments in motility grade A, in two samples. We did not observe any relationship between initial sperm concentration and platelet concentration in whole blood and motility pattern after 2 hr exposure to PRP, for both types of semen classification.

**Conclusion:** Our results suggest that PRP has a positive effect on sperm motility after 2hr exposure. The increment in motility grade seems to be related to the quality of the initial semen sample.

**P-152. Will the same morphokinetic algorithm work in the selection of embryos grown in different culture media?**

**Giovanna Leticia Simoes Lima^1^, Patricia Mendonca Leite^1^, Rebeca Santos Garcia^1^, Luiza Thamiris de Oliveira Machado^1^, Luisa Vianna Cancado^1^, Fernanda Ribeiro Rodrigues^1^, Gabriela Boller Bicalho^1^**

^1^Universidade Federal de Minas Gerais - Belo Horizonte - MG - Brazil

**Objective:** The present study aims to evaluate the impact of the psychosocial sphere in the treatment of patients undergoing infertility procedures.

**Methods:** This is a systematic review using the platforms PUBMED, Scielo, and archives from the Brazilian Society and the European Society of Assisted Reproduction using the following keywords: (Fertilization *in Vitro*) OR (Reproductive Techniques, Assisted) OR (Infertility) AND (Stress, Psychological) OR (Psychosocial Impact). After careful analysis, 6 articles were selected for the present study.

**Results:** Among the selected studies, a prospective study conducted with 352 women identified that over 50% of women undergoing infertility treatment exhibit depressive or anxious symptoms, with exacerbation of these conditions in cases of treatment failure. The impact of stress, beyond individual health, has clear repercussions on adherence to therapeutic proposals. There is significant evidence that treatment dropout regarding infertility procedures is associated with the level of psychological distress in patients. Another prospective study from Portugal, involving 139 couples, concluded that depressive symptoms are directly related to treatment discontinuation. Likewise, a cross-sectional study conducted in the USA indicated that the primary cause for discontinuation of *in vitro* fertilization protocols involves mental burden associated with the treatment. In many assessments, patients report that a more structured mental health approach, with appropriate guidance and assistance, could have changed their experience as patients going through assisted reproduction procedures. To reduce the impact of mental distress in these patients, there is ongoing discussion regarding multidisciplinary infertility follow-up approach. Many protocols, such as the European Society one, include training the healthcare team to diagnose and address mental distress promptly. However, despite the acknowledgment of the relevance of these interventions, studies show that only 20% of the patients felt adequately guided on stress management and mental health care.

**Conclusion:** Considering that infertility treatment can cause significant emotional strain on couples, it is important to focus on these conditions, which does not seem to be a priority of health clinics. The team should be able to observe and diagnose mental distress, help patients cope less exhaustively with treatment, enabling them to experience less suffering and better quality of life during treatment. That approach could improve adherence to proposed treatment with higher success rates, and consequently, greater personal satisfaction.

**P-153. Hypothyroidism associated with recurrent abortion, How can we prevent the next one?**

**Isabela Shiozawa Nolasco^1^, Stephanie Tasselli Alencar da Assunção^1^, Kemely Muraiber Ismail^1^, Mariana Kasuga Morya^1^, Victoria Barchi Cordts^1^**

^1^Universidade Santo Amaro - São Paulo - SP - Brazil

**Objective:** To evaluate the impact of hypothyroidism on recurrent abortions and how to prevent future occurrences.

**Methods:** For the present study, the systematic review method was adopted regarding the study of hypothyroidism associated with recurrent abortions and the evaluation of preventive measures. Articles indexed in the PubMed database, which included clinical trial, randomized controlled trial, and systematic review, dated between the years 2019 to 2024, in the English language, were selected. The following descriptors were used: "hypothyroidism," "recurrent abortion," "pregnancy loss," and "spontaneous abortions."

**Results:** Hypothyroidism subclinical (SCH) and positivity for thyroid peroxidase antibodies (TPOAb +) demonstrate thyroid autoimmunity, and women with recurrent pregnancy loss (RPL) with SCH or TPOAb + have a higher risk of adverse pregnancy outcomes. Such outcomes include a higher rate of placental abruption, stillbirth, miscarriage, preterm birth, fetal distress, gestational hypertension, and gestational diabetes. This occurs because throughout the entire pregnancy, the fetus relies on thyroid hormones provided by the mother, even after the fetus's thyroid gland is formed and functional. This hormone is crucial for fetal neurological development, fetal growth, and the development of fetal somatic tissue. Additionally, untreated hypothyroidism during pregnancy can lead to consequences such as neurocognitive developmental deficiency and lower intelligence quotient. Therefore, exploring therapies that modulate SCH and TPOAb+ is necessary to improve pregnancy outcomes in women with RPL. The efficacy of levothyroxine (LT4) treatment is still controversial in women with RPL with SCH or TPOAb+, as all articles selected for this study indicated the importance of further studies related to its use. Many studies indicate that the use of thyroxine in women with RPL with SCH or TPOAb+ reduces the rate of premature births, spontaneous abortion rates, decreases the risk of subsequent pregnancy losses, reduces fetal distress, decreases the risk of gestational hypertension, and increases the rate of live births compared to those with the same thyroid dysfunction who were not treated. These improvements are observed only with early treatment, and the LT4 dose should be monitored to avoid excess in treatment. However, some studies show that there is no statistically significant increase in live births and that there was no decrease in the rate of premature births when comparing women with RPL with SCH or TPOAb+ treated with LT4 versus those untreated. Moreover, there is no evidence of the benefit of LT4 use in pregnancy related to the physical or cognitive performance of children aged 3 to 5 years. One study indicates that only when LT4 treatment is administered to patients with TSH levels above 4.0 mU/L do the aforementioned benefits exist. However, for women with TSH between 2.5 mU/L and 4.0 mU/L treated with LT4, the risks remain the same as those untreated. Thus, the American Society for Reproductive Medicine (ASRM) did not make a clear recommendation for the treatment of SCH or TPOAb+ in women with RPL.

**Conclusion:** Women with recurrent pregnancy loss (RPL) with SCH and TPOAb+ have a higher risk of adverse pregnancy outcomes. Therefore, therapies that modulate SCH and TPOAb+ are needed to improve pregnancy outcomes in these women. However, treatment with Levothyroxine (LT4) for hypothyroidism is controversial for pregnancy outcomes in women with RPL who have SCH and TPOAb+. The results of this study showed that the effects of LT4 treatment in SCH or TPOAb+ pregnant women are not the same for all pregnancy outcomes. Additional well-designed studies are needed for preconception treatment, especially at TSH >4.0 mU/L, so the reproductive society can make a clear recommendation for SCH or TPOAb+ treatment in women with RPL. Prospective, randomized studies are urgently needed to prevent further episodes.

**P-154. Relationship between emotional state and clinical and laboratory behavior in patients undergoing IVF treatment**

**Márcia Bossoni^1^, Bruna Ribeiro Gobi^1^, Gabriela Merino^1^, Leonardo Previato Araújo^1^, Bianca Previato Araújo^1^, Kelly Colussi^1^, Edilberto Araújo Filho^1^**

^1^Centro de Reprodução Humana de São José do Rio Preto - São José do Rio Preto - SP - Brazil

**Objective:** The incidence of couples with infertility has been growing worldwide. Aproximately one out of six people suffer of infertility in the world today, according to the World Health Organization (OMS, 2023). That’s due to innumerous factors, but very little concern has been given to the emotional status of these couples. What are their feelings, their fears. Do couples with a good, connected relationship have better behavior in practical aspects of the treatment like oocyte quality, embryo quality and pregnancy rate than couples that behave differently. We want to evaluate if the emotional state of couples affect the clinical and laboratory results of couples submitted to *in vitro* fertilization (IVF) treatment in a private clinic.

**Methods:** This is a retrospective cohort study. Data was collected from January 2023 to March 2024. Inclusion criteria: patients who transferred two good quality blastocysts and <38 years of age (Group A) and patients 38 years or more who transferred 01 employed embryo (Group B). Criteria for Stable relationship: 1) no depressive episodes; 2) no episodes of anxiety; 3) no use of psychotropics; 4) Spirituality: belief in GOD; 5) Quality of life: good quality food and exercise regularly. We considered in the study only couples that had a good interaction with our psychologist, with at least 03 meetings with her. The Hyperstimulation protocol was similar in all patients, varying the dose according to the anti-mullerian hormone (AMH) level and age: r-FSH (10 mg/day), hMG (150-22 IU/DAY), antagonist GnRH for fresh transfers and dihidrogesterone, 10mgX2 for IVF to with Next generation sequencing (NGS) patients to avoid Luteinizing hormone (LH) surge and chorionic gonadotropin (HCG) for trigger. A total of 55 patients were included in the study, 29 in Group A (2 good quality blastocysts) and 26 in Group B (euploids embryos), and they were divide in Group 1: STABLE relationships and Group2: not STABLE.

**Results:** We had full data on 47 patients out of the 55 initially included in the study. Group 1 had 41 patients, 27 had a clinical pregnancy (PR: 66%) and 6 abortions (AR:22%). Group 2 had 6 patients, 3 clinical pregnancy (PR:50%) and 1 abortion (AR:33.3%). The others 8 patients belonged to Group 2 and were excluded because we were not able to get all the information we needed about them.

**Conclusion:** These are the preliminary results of a very important matter which is: Does the emotional state really affect the results in na IVF program. Our results do not answer this question, maybe because the number of couples is small, the Group 2 had a small number of couples. But, the results even though not significant, encouraged us to continue increasing our data to maybe bring in the future answers for this matter and light up other groups to look for this subject.

**P-155. Impact of age on beta HCG and implantation rates after thawed euploid embryo transfer**

**Diana Caroline da Silva Bastos^1^, Julia Gonçalves^1^, Eliana Morita^1^, Raquel Koike^1^, Rafaela Aguiar^1^**

^1^CITI HINODE - São Paulo - SP - Brazil

**Objective:** To evaluate the positive beta HCG and implantation rates in patients who underwent transfer of thawed euploid embryos, using own and donor eggs, according to age group.

**Methods:** This retrospective study was conducted from January 2022 to April 2024, including patients who used own or donor eggs and underwent transfers of thawed euploid embryos. Out of a total of 297 ICSI cases followed by biopsy, 166 cases were included in the study for having at least one euploid embryo transferred during the analysis period. Of these, 60 cases used donor eggs and 106 used their own eggs. The cases with own eggs were subdivided into four age groups: ≤35 years (25 cases with 29 embryos), 36-38 years (28 cases with 33 embryos), 39-40 years (28 cases with 30 embryos), and ≥41 years (25 cases with 27 embryos). The fourth group evaluated was recipients of donor eggs with a total of 60 cases with 65 embryos.

**Results:** Among patients with own eggs and age ≤35 years, the average beta HCG was 76% and the implantation rate was 74%. In the 36-38 years group, the average beta HCG was 71.42% and the implantation rate was 57.14%. For the 39-40 years group, the average beta HCG was 85.71% and the implantation rate was 78.57%. In the group of patients aged ≥41 years, the average beta HCG was 84% and the implantation rate was 74%. For patients who used donor eggs, the average beta HCG was 70% and the implantation rate was 59.17%. Implantation rates did not differ between the four groups evaluated (*p*=0.26) nor did the positive beta rates (*p*=0.43).

**Conclusion:** The results of this study indicate that the transfer of thawed euploid embryos results in high positive beta HCG and implantation rates, regardless of the origin of the eggs (own or donor). While there was variation in beta HCG and implantation rates among the different age groups, these differences were not statistically significant. Therefore, both patients using their own eggs and those using donor eggs have comparable chances of success in the transfer of thawed euploid embryos. These findings suggest that the practice of transferring thawed euploid embryos is effective and can be recommended as a viable strategy to improve pregnancy rates in in vitro fertilization treatments.

**P-156. Association between prolonged use of oral contraceptive, MTHFR mutation, and antiphospholipid antibodies with poor ovarian reserve**

**Edilberto Araújo Filho^1^, Leonardo Previato Araújo^1^, Bianca Previato Araújo^1^, Bruna Ribeiro Gobi^1^, Gabriela Merino^1^, Kelly Colussi^1^**

^1^Centro de Reprodução Humana de São José do Rio Preto - São José do Rio Preto - SP - Brazil

**Objective:** The number of young infertile women with low ovarian reserve is increasing substantially. The expression of the 5,10-Methylenetetrahydrofolate reductase (MTHFR) gene in human oocytes and preimplantation embryos suggests that the MTHFR gene is involved in folliculogenesis and female reproduction (Shahrokhi et al., 2017). MTHFR gene variants may be associated with Low Ovarian Reserve (LOR), diminished response to ovarian stimulation, and, consequently, a lower live birth rate after in vitro fertilization (IVF) (Laanpere et al., 2010). The objective of this study is to evaluate the incidence of MTHFR mutations (C677T/A1298C) and antiphospholipid antibodies (Antiphosphatidylserine [APS] and Anti-phosphatidylethanolamine [APE]) in infertile patients, as well as the prolonged use of oral contraceptives in a population of patients with anti-mullerian hormone (AMH) <0.8ng/ml.

**Methods:** This is a prospective observational study of infertile patients undergoing infertility investigation from January 2019 to December 2023 in an IVF center. A total of 160 patients with an AMH level < 0.8 ng/ml were included in the study, all 37 years of age or younger. However, only 120 patients had the complete workup of exams necessary to be included in the study.

**Results:** In the study (AMH < 0.8ng/ml), 91 out of 120 patients had a level of APS/APE > 12.0 ng/ml (IgG, IgM, IgA), which represents an incidence of 76%. Seventy-one out of 120 patients had a positive result for mutation in the MTHFR gene (heterozygous/homozygous), an incidence of 59%. We also observed that 85 out of 120 patients had a history of prolonged use of oral contraceptives (> 10 years), which represents an incidence of 71% in this particular population.

**Conclusion:** We observed a high incidence of MTHFR gene mutation, high incidence of APS/APE, and high incidence of prolonged use of oral contraceptives in this particular group of patients with low ovarian reserve.

**P-157. Efficacy of clomiphene citrate in oocyte retrieval and maturation: A retrospective study**

**Jonathan Boncristiano^1^, Bernardo Jonathan Moura^2^, Juliana Peralva Baumgratz Medeiros França^2^, Cecilia Souza Monteiro^2^, Emilio Costa Garavini^3^**

^1^Gerar In VItro - Itabuna - BA - Brazil

^2^Gerar In VItro - Belo Horizonte - MG - Brazil

^3^Gerar In Vitro - Ponte Nova - MG - Brazil

**Objective:** To analyze and compare the efficacy of oral clomiphene citrate with other medications used to suppress the hypothalamic-pituitary-ovarian axis in ovarian stimulation protocols for in vitro fertilization, assessing impacts on ovarian response.

**Methods:** For this retrospective study, we analyzed 11,461 oocytes from 1,074 cycles of assisted reproduction treatment and oocyte cryopreservation conducted between January 2021 and March 2024. All cycles involving controlled ovarian stimulation were categorized into two groups: the clomiphene citrate group for suppression and the control group, which encompassed all other methods of suppression utilized.

**Results:** The study results did not reveal statistically significant differences between the group receiving clomiphene citrate and the control group. The oocyte retrieval rate was similar in both groups: clomiphene citrate (75%±28.6%) versus control (74%±30.2%). The mean number of retrieved oocytes also showed no significant variation between the groups: clomiphene citrate (10±7.1) versus control (10.5±8.8). Additionally, the oocyte maturation rate was comparable between the groups: clomiphene citrate (79%±23.4%) versus control (78%±29.4%). The clomiphene citrate group was further analyzed based on age range. For women over 35 years old, the oocyte retrieval rate was 78%±31.4%, with an average of 13.2±12.3 oocytes retrieved and a maturation rate of 79.00%±18.5%. For those aged 35 to 39 years, the retrieval rate was 67%±29.7%, with an average of 6.6±4.9 oocytes retrieved and a maturation rate of 77%±22.4%. In the group of women aged 40 to 42 years, the retrieval rate was 75%±32.7%, with an average of 6.5±4.5 oocytes retrieved and a maturation rate of 78%±25.4%. Finally, for those over 42 years old, the retrieval rate was 55%±34.6%, with an average of 3.5±3.4 oocytes retrieved and a maturation rate of 84%±25.5%. The control group was also subdivided by age range, resulting in the following data. For women over 35 years old, the oocyte retrieval rate was 75%±27.6%, with an average of 15±11.8 oocytes retrieved and a maturation rate of 78%±16.6%. For women aged 35 to 39 years, the oocyte retrieval rate was 75%±33.2%, with an average of 10.8±4.7 oocytes retrieved and a maturation rate of 79%±26.7%. In the age range of 40 to 42 years, the oocyte retrieval rate was 68%±25.3%, with an average of 7.6±1.4 oocytes retrieved and a maturation rate of 74%±22.5%. Lastly, for women over 42 years old, the oocyte retrieval rate was 50%±21.26%, with an average of 4.1±3.9 oocytes retrieved and a maturation rate of 78%±35.3%.

**Conclusion:** Hormonal blockade using clomiphene citrate emerges as an attractive strategy in in vitro fertilization protocols, offering greater convenience and minimizing discomfort for patients, while also providing excellent cost-effectiveness.

**P-158. Oncofertility: The importance of fertility preservation and the development of an educational portal for oncology patients**

**Mariane Cristina Carlucci Molina Félix^1^, Carla Giovana Basso^1^, Nilo Frantz^1^, Luciana Lopes Manfredini^2^**

^1^Nilo Frantz Medicina Reprodutiva - São Paulo - SP - Brazil

^2^Faculdade Israelita de Ciências da Saúde Albert Einstein - São Paulo - SP - Brazil

**Objective:** Oncological patients do not have access to quality information when it comes to fertility preservation. The oncologists do not have time or knowledge with this theme and the importance of fertility preservation in oncological patients still is not well approach, especially through easy access information, as online content. Therefore, our study intents to review the present data on the subject and evaluate the value of future development of an educational portal for accessing information in oncofertility.

**Methods:** We conduct a thorough research, between June to September 2023, in Virtual Library in Health (BVS) and PubMed over the last five years. We selected studies with the terms “oncology”, “fertility preservation”, “infertility”, “reproductive health” and “Internet”. Portuguese, English, and Spanish-language complete articles were taken into consideration.

**Results:** Analyzed studies shown that the survival rate of adolescents and young adults with cancer has been increasing rapidly, highlighting the need to examine potential reproductive adverse effects of cancer treatment on this age group. The patients should be referred immediately to a fertility specialist before starting cancer treatment. Practitioners should develop a biological, psychological, social and spiritual approach, being crucial to acknowledge the potential for restoring autonomy following the end of treatment. Preservation of fertility is an emerging topic in oncology and rarely discussed among young survivors and, even though there is interest among patients about the possible impact of the treatment in their fertility, there is a gap in the frequency at which this information is provided. In this way, communication is the key factor that will impact adherence and build confidence between professional and patient in addition to education and reference programs. Studies shows that the provision of information through programs or flyers has led to a rise in the number of fertility preservation demands from this specific audience. Currently, the Internet has become the means of searching for information and a study carried out with patients with breast cancer, has shown the need to include informative materials about infertility, in this way patients will be empowered and will gain quality of life.

**Conclusion:** It is necessary to provide information on fertility preservation to oncology patients since cancer survival rates have increased significantly in recent years. These patients, when presented with fertility preservation therapies, will have the ability to make decisions while considering their quality of life as individuals who have/will survive cancer. The development of a portal could be a reliable source of information for patients, since online information has become the largest source of search for quick and accurate information, thus facilitating decision-making for oncology patients.

**P-159. Use of heat shock protein A5 (HSPA5) in the *in vitro* maturation media**

**Priscila Helena Santos^1^, Thainá Sallum Bacco Manssur^2^, Fernanda Fagali Franchi^2^, Sarah Gomes Nunes^2^, Patrícia Kubo Fontes^3^, Anthony César de Souza Castilho^4^**

^1^Universidade de São Paulo (USP) - Pirassununga - SP - Brazil

^2^Universidade Estadual Paulista de Botucatu (Unesp), Instituto de Biociências - Botucatu - SP - Brazil

^3^Universidade Federal do ABC (UFABC) - Santo André - SP - Brazil

^4^Universidade do Oeste Paulista (UNOESTE) - Presidente Prudente - SP - Brazil

**Objective:** Heat shock protein A5 (HSPA5) is a member of the heat shock protein family present in mammalian oviductal fluid (OF) during the peri-ovulatory period. It plays a critical role in maintaining cellular homeostasis and supports cell survival and proliferation. Studies also suggest its involvement in sperm capacitation and interaction with the ooplasm. Given the physiological and reproductive similarities between bovine and human systems, including OF composition, we hypothesized that the addition of oviductal proteins, such as HSPA5, could enhance oocyte quality following in vitro maturation (IVM) in patients undergoing assisted reproduction treatment. Therefore, this study aimed to investigate the influence of HSPA5 on the final maturation of cumulus-oocyte complexes (COCs) using bovine as an experimental model.

**Methods:** Bovine cumulus-oocyte complexes were obtained from slaughterhouses and allocated into in vitro maturation (IVM) treatment groups as follows: the Control Group (n=20), where COCs underwent a 24-hour incubation in FSH-enriched base medium, and the Treatment Group (n=20), where COCs underwent a 20-hour incubation in FSH-enriched base medium followed by a subsequent 4-hour incubation with half of the culture media volume replaced with base medium supplemented with human recombinant HSPA5 (concentration of 100 ng/ml). The experiment was conducted in triplicates. Following IVM, matured COCs underwent in vitro fertilization using MitoTracker-stained spermatozoa. Potential zygotes were then stained with Hoechst for polyspermy analysis using confocal laser scanning microscopy, or subjected to evaluation of zona pellucida digestion time using pronase treatment.

**Results:** The presence of HSPA5 in the IVM media did not result in a significant difference in the timing of zona pellucida digestion (259±12.29 seconds *vs*. 276.09±11.16 seconds for treatment and control groups, respectively; *p*=0.4093). Furthermore, HSPA5 did not impact the pattern of sperm penetration through the zona pellucida, as evidenced by comparable rates of polyspermic zygotes between the Control and Treatment groups (*p*=0.9669).

**Conclusion:** Although our investigation into the influence of HSPA5 on the final oocyte maturation process did not demonstrate changes in sperm penetration through the zona pellucida and did not significantly alter ZP digestion time in oocytes, our findings highlight the complexity and importance of studying oviductal proteins during oocyte maturation to explore new perspectives and possibilities for improving *in vitro* fertilization techniques.

**P-160. Artificial Intelligence and oocyte monitoring: An approach to track embryonic formation post-ovum collection**

**Stephanie Tasselli Alencar da Assunção^1^, Karla Sobral Menezes da Silva^1^, Mariana Kasuga Morya^1^, Victoria Barchi Cordts^1^, Marjorye Tasselli Alencar da Assunção^1^, Gabriel Monteiro Pinheiro^1^**

^1^Universidade Santo Amaro - São Paulo - SP - Brazil

**Objective:** To evaluate the use of artificial intelligence in conjunction with oocyte monitoring to track embryonic formation after egg collection.

**Methods:** For the present study, the systematic review method was adopted regarding the study of the use of artificial intelligence in conjunction with oocyte monitoring to track embryonic formation after egg collection. Articles indexed in the PubMed database, which included review, and randomized controlled trial, dated between the years 2021 to 2024, in the English language, were selected. The following descriptors were used: "artificial intelligence," "oocyte monitoring," and "embryo selection."

**Results:** Recent studies have delved into the profound advancements in embryo selection and assisted reproductive techniques (ART), leveraging technologies such as time-lapse videos (TLV), artificial intelligence (AI), and deep learning (DL). TLV combined with AI has revolutionized in vitro fertilization (IVF) by allowing continuous monitoring of embryonic development and automated analysis, thereby identifying subtle characteristics that significantly enhance embryo selection. However, challenges such as the subjective nature of TLV annotations and the opacity of AI algorithms have prompted the exploration of matte-box and glass-box systems to enhance transparency and interpretability. AI offers a comprehensive perspective on embryos, minimizing variability among observers and ensuring consistent results. Despite its promising potential in reproductive medicine, the widespread adoption of AI faces hurdles in standardization, privacy concerns, and the need for comprehensive clinician training. Machine learning algorithms, integrating variables like age and embryo quality, aim to optimize the success rates of ART procedures. Nonetheless, robust clinical evidence validating the efficacy of AI in enhancing embryo selection remains crucial. In addressing male infertility, AI plays a pivotal role in predicting semen quality, thereby facilitating diagnostics and prognostics. Clinical decision support tools (CDSS) powered by AI personalize ART treatments by considering individual factors such as ovarian reserve and genetic polymorphisms. However, there is apprehension regarding the reliability of "black-box" AI systems, which underscores a preference for transparent algorithms. Rigorous validation protocols are essential before the clinical integration of these systems can be confidently pursued. Furthermore, the integration of AI with digitized medical records and big data holds the promise of revolutionizing IVF practices, allowing for personalized treatments and enhanced procedural accuracy. Deep learning techniques automate tasks such as feature extraction and image analysis, aiding in critical processes like cell counting, embryo tracking, and outcome prediction. Nevertheless, challenges persist in validating these models across diverse equipment and clinical settings. Microfluidics has emerged as a pivotal technology in reproductive biology, enabling precise studies of sperm motility and embryonic development within environments that mimic natural conditions. This approach enhances the accuracy of reproductive assessments and deepens our understanding of these complex biological processes. Oocyte monitoring, a crucial aspect of assessing IVF success, benefits significantly from TLV and AI integration, enabling continuous observation and detailed analysis of oocyte development to identify optimal candidates for fertilization. In summary, the collective findings underscore the transformative impact of emerging technologies on assisted reproduction, notably improving embryo selection, tailoring treatments to individual needs, and mitigating laboratory errors. However, the widespread adoption of these technologies necessitates rigorous safeguards to ensure safety, efficacy, and equitable access, underlining the ongoing need for robust clinical validation and regulatory oversight.

**Conclusion:** The integration of AI in oocyte monitoring represents a significant advance in IVF. This study reviewed how AI, combined with techniques such as TLV, can improve embryo selection and outcomes of ART. However, the practical implementation of AI faces obstacles, including standardization, privacy and professional training. Vigorous evidence and clinical validations are needed to ensure efficacy and safety. Additionally, the digitization of medical records and technologies such as microfluidics offer new possibilities for personalizing and improving IVF treatments.

**P-161. CRISPR/Cas9: Editing the DNA of the Future**

**Ermeni Pires do Nascimento^1^, Stephanie Tasselli Alencar da Assunção^1^, Isabella De La Jara Gagliardi^1^, Mariana Kasuga Morya^1^, Victoria Barchi Cardts^1^, Gabriel Monteiro Pinheiro^1^**

^1^Universidade Santo Amaro - São Paulo - SP - Brazil

**Objective:** To evaluate the potential of CRISPR/Cas9 technology for DNA editing, investigate its future applications, and discuss the ethical issues associated with genetic editing.

**Methods:** For the present study, the systematic review method was adopted regarding the assessment of the potential of CRISPR/Cas9 technology for DNA editing, investigating its future applications, and discussing the ethical issues associated with genetic editing. Articles indexed in the PubMed database, which included review, dated between the years 2019 to 2024, in the English language, were selected. The following descriptors were used: "CRISPR/Cas9," "assisted reproduction," “bioethics,” and “infertility”.

**Results:** The ethical evaluation in research proposals employing CRISPR/Cas9 technology is a complex task that requires a multidisciplinary and reflective approach. Ensuring informed consent, safety, justice, transparency, and regulatory compliance are fundamental to ethical practice. Studies by Pei et al. on gene editing have gained traction over the years, in 2019, the discussion of possibilities and challenges of epigenome editing via CRISPR/Cas9 had significantly progressed. This technique has a transformative potential in clinical settings, offering new possibilities to treat a wide range of diseases, from genetic disorders to more effective and targeted treatments, always guided by strict ethical and regulatory frameworks to ensure its safe and responsible application. Medical advances in genome editing bring both scientific opportunities and ethical challenges. Hereditary genome editing has the potential to eliminate hereditary diseases and improve human health. However, advances in this area are accompanied by significant scientific challenges and profound ethical issues. The precision and safety of the technology still need to be improved, and ethical implications, such as genetic autonomy, designer babies (genetically modified fetuses with non-therapeutic enhancements), and equity in access, must be carefully considered. Focusing on human reproduction, CRISPR/Cas9 allows for strategies in identifying genetic variants causing infertility, a common multifactorial issue. Identifying these variants is crucial for developing effective treatments and providing accurate diagnosis. CRISPR/Cas9 in male infertility, particularly spermatogenesis, is a widely discussed aspect of study, with great potential in genetic manipulation. Lastly, recent studies by Zhang et al. showcase CRISPR/Cas9’s incredible advancements in editing oocytes and early embryos. Opening new frontiers for understanding developmental biology, as well as for the possible correcting genetic mutations in early stages of life.

**Conclusion:** It is undeniable that technology behind CRISPR/Cas9 is capable of revolutionizing modern medicine. Its applications in reproductive medicine, particularly in infertility, are promising. CRISPR/Cas9 has shown potential in correcting genetic mutations in oocytes and embryos, which could significantly enhance fertility treatments and outcomes, including curing genetic disorders. This technology holds great potential for personalized treatments, including identifying the genetic variants causing infertility, advancing spermatogenesis research. Albeit, the use of CRISPR/Cas9 does not come without limitations. The high cost of these advanced treatments makes them largely inaccessible to the broader public, raising concerns about equity in access. Moreover, the precise nature of genome editing brings ethical dilemmas, particularly the potential for “designer babies,” where genetic modifications could be used for non-therapeutic enhancements, challenging societal norms around genetic autonomy and fairness. In conclusion, while CRISPR/Cas9 holds incredible potential for modern medicine, it must be governed by strict ethical guidelines and equitable access policies to ensure responsible use.

**P-162. Semaglutide and infertility: Could this become an option of treatment?**

**Sophia Abur Said^1^, Carolina Comissoli Fernandes^1^, Isadora de Carvalho Schramm^1^, Camila Haas^1^, Júlia Prauchner de Castilhos^1^, Laura Randon Chapochnicoff^1^, Isabella Lanzarini Erdklee^1^, Anna Luiza Reinehr Ferreira^1^, Joana Carmona Neuwald Celeste^1^, Giovanna Vissoky Cé^1^, Victória Campos Dornelles^1^, Marta Ribeiro Hentschke^1^**

^1^Pontifical Catholic University of Rio Grande do Sul - Porto Alegre - RS - Brazil

**Objective:** To review the effects of Ozempic at fertility.

**Methods:** The study is a literature review, of which an advanced search was carried out at PubMed and ScienceDirect database platforms. The database search was conducted using the keywords (("Ozempic") OR ("Semaglutide") OR ("GLP-1 receptor agonist") OR ("GLP-1 drugs") OR ("diabetes medication")) AND (("Fertility") OR ("Infertility") OR ("assisted reproduction") OR ("artificial reproduction") OR ("in vitro fertilization") OR ("IVF")). Articles published in English, with publication date in the last 15 years, were selected. After removing unavailable articles and duplicates, 9 of the 44 articles evaluated were included.

**Results:** Ozempic, a GLP1 Receptor Agonist (GLP-1RA), is a medication originally designed for Diabetes Mellitus type 2, but has become widely off-label used as a treatment for obesity. Being overweight in the preconception period is associated with poorer reproductive outcomes, so current literature maintains that Ozempic increases fertility due to better metabolic conditions. The studies analyzed showed that short-term medication and lifestyle interventions typically result in weight loss ranging from 2 to 9 kg, over a period of 4 to 6 months in women with obesity and infertility. However, the lost weight achieved with these interventions does not appear to improve cycle conception or live birth rates in women undergoing ovulation induction, intrauterine insemination, or in vitro fertilization cycles. Actually, studies have suggested it might increase natural conception rates, particularly among overweight women with anovulatory infertility. A recurrent condition in studies was Polycystic Ovary Syndrome (PCOS), which is the most common endocrine disorder among young women of reproductive age and one of the most common causes of reproductive dysfunction, such as subfertility and infertility. In addition, many women with PCOS are obese, which may lead to an increased risk of developing glucose intolerance, diabetes mellitus and systemic inflammation. Analyses regarding this group of women have found impaired incretin secretion and activity, which can be attenuated by GLP-1RAs, that is emerging as new therapeutic options for PCOS. Semaglutide was studied in mice and in randomized studies with humans showing that it presents a role in the pathogenesis of PCOS and that the reduced levels of insulinemia and androgenemia were followed by decreased inflammatory mediator levels. Furthermore, another GLP-1RAs that appeared in the research relating to increase women's fertility was Liraglutide, which has randomized studies showing improved ovarian function, higher menstrual regularity, and reduced androgen levels in the group receiving liraglutide versus the placebo group. Thus, there is evidence of similar pathophysiology in PCOS, diabetes and obesity. Hence, the association of ozempic to fertility improvement may occur in cases when anovulatory infertility such as PCOS, obesity and diabetes.

**Conclusion:** Anti-obesity medications, such as Semaglutide, plays an important role in women’s lives, and there is still little research with few findings associating the use of ozempic for obesity and its possible impact on reproductive outcomes. With the current information, there is growing interest in GLP-1RAs as a potential treatment for obesity-related issues particularly in women with PCOS. However, the impact of ozempic at fertility remains unknown. It could become, however, an option only for infertile patients with PCOS, obesity and diabetes. In this context, it is crucial to provide women with information, so shared informed decisions about pregnancy and the use of anti-obesity medication can be made.

**P-163. The use of inositol in Assisted Reproduction for patients with polycystic ovary syndrome**

**Andressa Maria da Silva Dias^1^, Stephanie Tasselli Alencar da Assunção^1^, Maria Giovanna de Araujo Ferreira^1^, Mariana Kasuga Morya^1^, Victoria Barchi Cordts^1^**

^1^Universidade Santo Amaro - São Paulo - SP - Brazil

**Objective:** To evaluate the use of inositol in assisted reproduction for patients with polycystic ovary syndrome.

**Methods:** For the present study, the systematic review method was adopted regarding the study of the use of inositol in assisted reproduction for patients with polycystic ovary syndrome. Articles indexed in the PubMed database, which included randomized controlled trial, and review, dated between the years 2021 to 2024, in the English language, were selected. The following descriptors were used: "inositol," "assisted reproduction," and "polycystic ovarian syndrome".

**Results:** The use of inositol in assisted reproduction for patients with Polycystic Ovary Syndrome (PCOS) has been the subject of various studies, highlighting its effectiveness in improving ovarian function and oocyte quality. Studies have shown that the combination of myo-inositol and d-chiro-inositol (MI+DCI) offers benefits in menstrual regularity and insulin resistance, surpassing metformin in reducing testosterone levels, while thiazolidinediones (TZDs) have demonstrated improvements in lipid profiles. Additionally, meta-analyses have revealed that inositols, including myo-inositol and inositol in general, reduce testosterone levels, normalize the menstrual cycle, improve insulin resistance and carbohydrate metabolism, and are considered a promising alternative to metformin due to fewer side effects. Comparative studies between myo-inositol and metformin have shown that both treatments improve metabolic and reproductive profiles in women with PCOS, with myo-inositol demonstrating greater efficacy in reducing androgens and improving pregnancy rates, while causing fewer gastrointestinal side effects. It has also been noted that the effectiveness of inositol in assisted reproduction is still uncertain, although it has shown additional benefits in menstrual cycle regularity and quality of life when combined with metformin. Moreover, the addition of myo-inositol to metformin improved menstrual regularity and quality of life, suggesting a combined approach. The comparative efficacy between myo-inositol and metformin remains uncertain, and the available evidence is limited and inconclusive. Regarding assisted reproduction, studies have demonstrated that myo-inositol improves ovarian sensitivity, fertilization rates, and reduces the required dose of gonadotropins in patients with poor ovarian response during IVF cycles. These findings suggest a promising role for myo-inositol as an adjunct in assisted reproduction treatments, although larger and more controlled studies are needed to confirm these results. Therefore, the use of inositol, particularly myo-inositol, shows potential in improving reproductive outcomes in patients with PCOS, both in clinical treatments and assisted reproduction procedures. However, further research is needed to clarify its comparative efficacy with metformin, standardize dosages and treatment regimens, and confirm its benefits in clinical practice.

**Conclusion:** Analysis of studies concludes that myo-inositol improves reproductive outcomes in women with PCOS, optimizing ovarian function, menstrual regularity, insulin resistance, and oocyte quality. The combination of myo-inositol and d-chiro-inositol is more effective than metformin in reducing androgens and increasing pregnancy rates. In addition, adding inositol to metformin can improve quality of life and menstrual regularity. Studies indicate that inositol has better tolerability than metformin, with fewer gastrointestinal side effects, making it a promising option for PCOS treatment, especially in women with hyperandrogenism. Although direct comparison with metformin still requires more high-quality clinical trials, the results suggest that inositol is a viable alternative. Further research is crucial to confirm the efficacy of inositol, establish dosage and treatment guidelines, and provide accurate clinical guidance. Continued research and interdisciplinary collaboration are essential to optimize PCOS management and ensure the safety and efficacy of treatments. In summary, myo-inositol is beneficial for improving reproductive outcomes in PCOS patients, representing a promising alternative to metformin.

**P-164. Impact of peritoneal fluid on embryonic development in an endometriosis mouse model**

**Luanna Prudencio de Araujo^1^, Michelle Guimarães dos Santos^1^, Caroline dos Santos da Fonseca^1^, Rafael Montenegro da Silva Lima^1^, Mariana Santos Costa^1^, Luiz Eurico Nasciutti^1^, Marcel Frajblat^1^**

^1^Universidade Federal do Rio de Janeiro - Rio de Janeiro - RJ - Brazil

**Objective:** Endometriosis is a chronic gynecological disease characterized by the presence of uterine tissue similar to glandular and stromal tissue outside the uterus, generating ectopic endometriotic lesions. Among the sites for the establishment of these lesions are the ovaries, intestine, cervix, vagina, bladder, and others. However, the most common site is the pelvic peritoneum. The peritoneum is a serous membrane that functions to line the abdominal cavity, contributing to the support of organs and serving as a channel for the passage of nerves, blood vessels, and lymphatic vessels. The peritoneal fluid is a fluid present in this cavity and, under physiological conditions, contributes to the irrigation and lubrication of the region. In pathologies such as endometriosis, there are intense processes of neoangiogenesis for the nutritional supply of tissue, as well as the modulation of factors such as chemokines, interleukins, and growth factors, creating a pro-inflammatory microenvironment at the site of the lesion. As a consequence, there is a change in the composition of the peritoneal fluid, which will contain a high expression of these mediators of inflammation, including activated macrophages. It is known that peritoneal fluid can also perfuse the fallopian tubes, thereby bathing the uterus. Therefore, this work aims to understand if there is a tuboperitoneal factor such that this pro-inflammatory fluid can impact embryonic development.

**Methods:** For this, we used an animal model of endometriosis in mice*, in which an induction surgical procedure is performed by suturing grafts of uterine fragments into the peritoneum. The C57BL/6 isogenic strain was used to avoid tissue rejection of the fragments. After establishing the lesion at 30 days, an abdominal lavage was performed with PBS in both control females Sham (n=7) and induced endometriosis females (n=7). Subsequently, another group of females (n=10) was stimulated with equine chorionic gonadotropin (eCG) and human chorionic gonadotropin (hCG) and placed with breeding males to collect two-cell embryos by washing the oviducts. Embryos were then selected according to their morphology and divided in three groups: 1) a control condition with regular culture media (CTRL), 2) an experimental condition in which the medium was supplemented with 10% peritoneal fluid from control females (CTRL_PF10%), and 3) an experimental condition in which the medium was supplemented with 10% peritoneal fluid from induced endometriosis females (END_PF10%). After that, the dishes were placed in the incubator to monitor embryonic development. This study has been approved by the Ethics Committee on the Use of Animals with Letter of Approval number 144/23.

**Results:** Results indicated that all groups achieved a blastocyst rate exceeding 90%.

**Conclusion:** This suggests that the 10% concentration used may not have been sufficient to significantly affect embryonic development. Therefore, it is necessary to repeat the experiment using higher concentrations of peritoneal fluid supplementation in the culture medium.

**P-165. Impact of filgrastim on blastocyst transfers in egg recipients cycles with previously implantation failure according to KIR genotyping**

**Rogerio Barros Ferreira Leão^1^, Arnaldo Schizzi Cambiaghi^1^, Paula Bortolai Martins Araujo^1^**

^1^IPGO - São Paulo - SP - Brazil

**Objective:** To compare ongoing pregnancy rate (OPR) in blastocyst transfers in egg recipients cycles with previously implantation failure according to Killer cell immunoglobulin-like receptor (KIR) genotyping. And to evaluate the effect of filgrastim in this group.

**Methods:** It is a retrospective study that evaluated 442 embryo transfers in egg recipients cycles performed from January 2020 to March 2024. The Inclusion criteria were: transfer of ate least one blastocysts graded as 3BB or better, from donated oocytes from a donor aged < 32 years old. Exclusion criteria were severe adenomyosis and no information on the evolution of the pregnancy until at least 14 weeks. The patients were initially divided into 3 groups, according to the KIR genotyping test: KIR AA, KIR Bx (AB or BB) and without KIR information. Patients with KIR test had at least one implantation failure as egg recipient, while those without KIR evaluation had no previous failures as a recipient. Group 1 and group 2 were divided into two groups according to whether or not filgrastim was used. The protocol of filgrastim use was: filgrastim 300 mcg/ml - 0.25 ml subcutaneously every other day from the day of embryo transfer and maintained until 8 weeks of gestation. OPR was compared between groups using the chi square test or Fisher's test.

**Results:** Of the 442 patients, 41 were KIR AA, 123 were KIR Bx and 278 did not have KIR information. KIR AA patients had an OPR of 26.54% and KIR Bx, 37.4%, with no statistical difference between the groups, but both lower than patients without KIR research (67.8%, *p*<0.05). When we separated groups 1 and 2 according to the use of filgrastim, we observed that KIR AA patients without filgrastim had extremely low OPR (only 5%), significantly lower than KIR Bx patients without filgrastim (29.54%, *p*<0.05). In patients with KIR AA, the use of filgrastim increased the OPR from 5% to 43.48% (*p*<0.05). In patients with KIR BB, the OPR increased from 29.54% to 57.1% with the use of filgrastim (*p*<0.05). When we compared OPR between KIR AA and KIR Bx who used filgrastim, those with KIR BB had a higher OPR (57.1% x 29.54%) but not significant. The same occurs when we compare OPR of KIR AA and KIR BB patients using filgrastim with those without KIR research: lower rate but without statistical difference.

**Conclusion:** Egg recipients with previous implantation failure and KIR AA have very low OPR even with good quality blastocyst transfer and this rate is lower than patients with KIR Bx. The use of filgrastim seems to significantly increase OPR for recipients with implantation failure with both KIR AA and KIR BB, although better benefit was observed in KIR AA group. Considering the very low OPR found in KIR AA recipients without filgrastim use, KIR genotyping and filgrastim use may be considered in cases of implantation failure. Nevertheless, this is a retrospective study and a randomized study with a larger number of patients is necessary to reach definitive conclusions.

**P-166. Female age and morphokinetic profile of embryos by time lapse**

**Guilherme Rios Franco^1^, Pedro Henrique Rios Franco^1^, Antonio Carlos Costa Franco1, Victoria Christine Carvalho de Oliveira^1^, Beatriz Müller Nunes Souza^1^, Vanessa Mesquita Prada^2^, Giovanna Dente Stella^1^**

^1^Embryolife Instituto de Medicina Reprodutiva - São José dos Campos - SP - Brazil

^2^Fecondare - Florianópolis - SC - Brazil

**Objective:** To measure if morphokinetic parameters are affected as female age increases.

**Methods:** The morphokinetic parameters of 519 embryos from patients with different age groups were analyzed. An ideal embryonic development curve was established and embryos were classified into two distinct groups: inside the curve or outside the curve.

**Results:** The incidence of embryos within the ideal cleavage curve did not change as female age increased. Patients aged 30 years had the rate of 34.3% of embryos inside the curve, this rate was also observed in patients up to 46 years of age.

**Conclusion:** The results of the morphokinetic analysis do not change with increasing female age.

- Patients aged 30 to 46 years have 34.3%(±2%) of embryos inside the curve.

- The morphokinetic algorithm used is not capable of detecting natural changes and the decrease in oocyte quality with age.

**P-167. The effectiveness of epididymal and testicular sperm retrieval on ICSI: Clinical and laboratory outcomes**

**Lígia Souza Cordesco^1^, Camila Macedo Medina^1^, Bruna Bizio Parra Oliveira^1^, Karla Pacheco Melo^1^, Janaina Romanini Silva^1^, Michelli Suemi Tanada^1^, Ivan Henrique Yoshida^1^, Emerson Barchi Cordts^1^, Caio Parente Barbosa^1^**

^1^Instituto Ideia Fértil - Santo André - SP - Brazil

**Objective:** The aim of this study was to evaluate the effects of epididymal and testicular sperm retrieval samples on clinical and laboratory outcomes in Assisted Human Reproduction treatments.

**Methods:** Retrospective study including 118 cycles submitted to Intracytoplasmic Sperm Injection (ICSI) using testicular or epididymal surgical sperm retrieval, from 2021 to 2023. The surgical procedure performed in each patient was decided by the clinician according to the male infertility factor, and all obstructive azoospermic men in this study went through vasectomy. After ICSI, all formed embryos were cultured to blastocyst stage and subsequent embryo transfer. Fertilization, cleavage, blastulation, blastocyst quality (top quality classified as AA, AB, BA), and clinical pregnancy rates were evaluated among types of surgical procedure (aspiration or extraction), considering maternal age (up to 37 years old and ≥ 38 years old), and the seminal sample status (fresh or frozen). To statistical analysis Linear Regression tests were performed, considering α = 5% and using statistical program JAMOVI 2.5.5.

**Results:** From all 118 cycles, 81 patients belonged to group 1 (epididymal sperm retrieval) and 37 to group 2 (testicular sperm retrieval). The median of injected oocytes for group 1 was 7.78 oocytes and 9.68 for group 2, totalizing 451 injected oocytes. Maternal age median on group 1 was 37.8 years old and male age median 49.2. On group 2, maternal median age was 36,8 and male median age was 47.0. A slightly higher fertilization and clevage rates were found in group 1 when comparing to group 2 (*p*=0.053; *p*=0.054) respectively. Although no significant *p* value was found, the proximity of p value considering significance (*p*<0.05) indicates a tendency to difference between analyzed groups. Blastulation and pregnancy rates didn’t show statistically significant results between groups (*p*=0.350; *p*=0.656, respectively). Yet, maternal age has shown to interfere blastulation rates, despite the analyzed group, corroborating to literature data that show maternal age as an important factor on blastulation rates. The sperm sample status (fresh or frozen) was also compared and fresh sample has shown higher blastulation rates than frozen (*p*=0.037). Finally, top quality blastocysts rates has shown no statistically significant results.

**Conclusion:** Epididymal and testicular sperm retrieval has shown no statistically significant results in fertilization, cleavage, blastulation and pregnancy rates, although there is a significant difference in blastulation rates when using fresh surgical samples compared to those frozen. Epididymal sperm tend to show higher laboratory rates when compared to the testicular. That being said, to obstructive azoospermic patients submitted to vasectomy, both ways of surgically retrieving spermatozoa are acceptable approaches on Assisted Reproduction treatments.

**P-168. Use of mild analgesics during Assisted Reproduction treatment: Associations with laboratory outcomes in Brazilian population**

**Carla Giovana Basso^1^, Ivana Rippel Hauer^2^, Fernanda Robin^1^, Francisco Furtado^2^, Anderson Martino Andrade^3^, Nilo Frantz^1^**

^1^Nilo Frantz Medicina Reprodutiva - São Paulo - SP - Brazil

^2^Fertway - Curitiba - PR - Brazil

^3^Universidade Federal do Paraná - Curitiba - PR - Brazil

**Objective:** Studies have already indicated that mild analgesics can disturb reproductive development by acting as endocrine disruptors, however further research is needed to investigate their associations with reproductive capacity. Therefore, our objective is to evaluate the associations of analgesics consumption on reproductive outcomes of women undergoing infertility treatment.

**Methods:** We conduct a prospective cohort study with 98 women (n=125 cycles), between 2018-2021 in South Brazil. Our study includes women aged 20-40 years, diagnosed with infertility and indication to undergo in vitro fertilization treatment (IVF). They responded to baseline questionnaires regarding their medication use during controlled ovarian hyperstimulation protocol for IVF (COH). They were asked specifically about their consumption of paracetamol (acetaminophen), dipyrone, ibuprofen, acetylsalicylic acid (AAS) and sodium diclofenac - the most used mild analgesics by Brazilian population. Reproductive outcomes information includes laboratory outcomes and clinical outcomes. General linear models and logistic regression were used to estimate the associations between each analgesic use during COH and the continuous and categorical reproductive outcomes. We also test potential cofounders and adjust the models to age, BMI and infertility cause (model I) and indication of use of each analgesic (model II).

**Results:** The report of paracetamol use during COH was negatively associated with the embryo quality rate in the crude model [*p*=0.01; -16.61(-27.52 to -5.70)], model I [*p*=0.01; -16.47(-27.46 to -5.47)] and model II [(*p*<0.01; - 29.02(-48.88 to -9.16)]. The use of dipyrone was positively associated with embryo blastulation rate in crude model [*p*=0.04; 15.45(0.95 to 29.95)] and model I [*p*=0.02; 16.70(2.42 to 30.98)], but not in model II. Ibuprofen consumption during the COH revealed a positive association with collection rate and a negative association with fertilization rate in crude model [(*p*=0.02; 12.21(1.71 to 22.71)), (*p*=0.02; -12.29(-22.51 to -2.07)) ] and in model I [(*p*=0.04; 10.97(0.33 to 21.62)), (*p*=0.02; -11.69(-21.83 to -1.55))], but no significant association after adjustment for indication of ibuprofen use (model II). No significant associations were observed with consumption of AAS or in relation to clinical reproductive outcomes for any analgesic.

**Conclusion:** We report associations between analgesic use and reproductive outcomes, however most of the associations were not sustain after accounting for confounding by indication. On the other hand, negative association of paracetamol use during COH with embryo quality was observed in all models. However, it is important to highlight that the infertility conditions of study participants may result in the use of mild analgesics and that both the medical conditions per se and the resulting use of analgesics have a potential to directly affect the outcomes of our study.

**P-169. Evaluating the impact of chronic endometritis on embryo development in IVF cycles: A case-control study**

**Jamille Ribeiro Santana^1^, Gustavo Nardini Cecchino^1^**

^1^Mater Prime and Mater Lab - São Paulo - SP - Brazil

**Objective:** Various theories propose that chronic endometritis might negatively influence in vitro fertilization (IVF) outcomes through changes in the endometrial microbiome. Animal studies suggest that this condition can disrupt cellular meiosis, reduce oocyte quality and quantity, and hinder oocyte maturation and embryonic development. This study aims to investigate the potential impact of chronic endometritis on the developmental potential of embryos in IVF cycles.

**Methods:** This retrospective matched case-control study included 70 patients undergoing IVF cycles at a single center between April 2018 and September 2023. Patients were divided into two groups based on the presence (n=35) or absence (n=35) of chronic endometritis. Given the low sensitivity and specificity of hysteroscopy with histological analysis, only patients who underwent endometrial biopsy followed by a molecular test (DNA extraction, ribosomal 16S gene amplification via RT-PCR, and hypervariable region analysis through next-generation sequencing) were included, achieving sensitivity and specificity > 90%. Oocytes and embryos were graded using the Istanbul consensus and Gardner blastocyst grading system. Day-3 embryos were classified into good quality (≥6 cells AND grade A or B) and low quality (<6 cells OR grade C). Blastocysts were categorized according to the SART classification. Descriptive statistics and appropriate statistical tests were performed using SPSS software.

**Results:** There were no significant differences in demographic variables and cycle parameters between groups. The mean number of mature oocytes retrieved was 8.9 in the endometritis group versus 10.1 in the control group (*p*=0.936). Fertilization rates were 85% for the endometritis group and 88% for the control group (*p*=0.793). Blastulation rates were 58% and 65% (*p*=0.124), respectively. The proportion of good quality day-3 embryos was similar between groups (81% *vs*. 84%, *p*=0.753). For the SART blastocyst scoring system, no significant differences were observed in the proportions of good (43% *vs*. 41%, *p*=0.593), moderate (45% vs. 50%, p=0.837), and low grade (12% *vs*. 8%, *p*=0.740) blastocysts between groups.

**Conclusion:** This study found no significant differences in embryo quality and developmental potential between women with and without chronic endometritis undergoing IVF treatments. However, these findings do not exclude the possibility that chronic endometritis could affect other reproductive outcomes. The study's limitations include its retrospective design and small sample size, which may impact the generalizability of the results. Further research is needed to validate these findings.

**P-170. The effect of EMBRYOGLUE on the motility and DNA fragmentation of cryopreserved spermatozoa**

**José Xavier Silva Neto^1^, Vera Lúcia Lângaro Amaral^1^, Alfred Senn^1^, Rafael Alonso Salvador^1^**

^1^Universidade do Vale do Itajaí - Itajaí - SC - Brazil

**Objective:** To evaluate the effects of EmbryoGlue medium on sperm viability and DNA fragmentation after cryopreservation and thawing.

**Methods:** Fifteen seminal samples from normozoospermic patients were cryopreserved in 0.5 mL straws in the presence of a cryoprotectant (Ingámed, Brazil), then thawed (10 min, 28°C) and washed by centrifugation (6 min, 1480 RPM) with GV-HEPES medium (Ingámed, Maringá, Brazil), and finally kept at 28°C. At time T0, motility, vitality, and DNA fragmentation parameters were analyzed (SCD method). The samples were then divided into three groups (G0, G3, and G10) of 200 µl each, containing 0%, 3%, and 10% EmbryoGlue^®^ (VitroLife, Brazil), respectively. Mobility and vitality parameters were reassessed at 10 min (T1), 1 h (T2), 3 h (T3), and 24 h (T4), and fragmentation at T3 and T4. Statistical data was analyzed using the RStudio program and the ANOVA and Tukey tests. Differences were considered significant when *p*<0.05.

**Results:** The addition of 10% EmbryoGlue led to a significant increase (*p*<0.001) in progressive motility and a significant increase (*p*≤0.005) in total motility without significantly decreasing the integrity of sperm DNA when compared to the control. The presence of 3% EmbryoGlue led to a significant increase (*p*=0.02) in progressive motility and a non-significant increase in total motility and sperm DNA fragmentation compared to the control. Vitality and motility remained similar in all groups during the test period.

**Conclusion:** Given that increased motility was not associated with loss of DNA integrity and reduced vitality, it is concluded that EmbryoGlue, at the concentrations used, has no adverse effect on the vitality and integrity of sperm DNA and has a positive impact on sperm motility. Its use in assisted reproduction techniques, such as intrauterine insemination, could increase fertilization efficiency. In the context of ICSI, EmbryoGlue would reveal which sperm show greater motility and hyperactivation, making it a new method of sperm selection.

**P-171. Fertility preservation: Profile of patients who underwent oocytes cryopreservation in a private Human Reproduction clinic**

**Fúlvia Estefânia Padre e Fechine^1^, Gabriela Cristina Fonseca Almeida^1^, Édlla Mikaine Padre e Fechine^1^, Raissa Ramos Coelho Penha^1^, Patrícia Tourinho da Silva^1^**

^1^FERTVIDA São Luís - São Luís - MA - Brazil

**Objective:** The objective of this study was to report on the profile of patients who underwent oocyte cryopreservation in a private Human Reproduction clinic and determine what was the most common motivation for cryopreservation.

**Methods:** The data used in this study is from anamnesis/clinical sheet and medical records of patients who performed oocytes cryopreservation between April 2020 and December 2023 in a private Human Reproduction clinic in Brazil. The patients were categorized into two groups: group A aged < 35 years and group B aged > 35 years. The sum (total), average (mean), standard deviation (sd) and percentage were performed in Microsoft Excel. Two terms were used to classify a motivation for cryopreservation being the term “medical reasons” refers to the presence of medical advice in the decision to cryopreserve the oocytes such as a diagnosis of some pathology. The term "social" refers to preventative treatment not related to medical diagnosis. Fisher's exact test was used to compare whether there was a statistical difference in the number of frozen oocytes between the groups. Statistics were performed using the GraphPad program. Differences were considered significant with a *p*-value of <0.05.

**Results:** As a result, 28 patient data was evaluated. The study found that the average age of patients was 33.8 (sd, ±5) years, 45% aged 22 to 35 years and 55% between the ages of 36 and 39 years. 85% cryopreservation for fertility preservation was for social reasons such as postponing motherhood and 15% for medical reasons (oncology patients). 79% were single marital status at the time of cryopreserving their oocytes. Regarding the identification of ethnic/race origin, 57% were identified as white and 42% as “parda” as described by the Brazilian Institute of Geography and Statistics (IBGE). 89% were university educated and in professional employment. A total of 285 eggs were frozen, 193 oocytes from group A, an average of 16 (sd, ±7) per patient and 92 oocytes from group B, an average of 6 (sd, ±3) per patient. The number of frozen oocytes in group A was statistically significant (*p*<0.05) higher than the total number of oocytes in group B.

**Conclusion:** In conclusion, the majority of patients in this study were single and with higher education. Furthermore, the data shows that the majority of patients froze their oocytes when they were > 35 years old, when their fertility appears to have already been compromised by age, considering the smaller number of frozen oocytes. The findings also suggest that the main motivation for freezing oocytes was the personal choice of preserving fertility for future reproductive purposes.

**P-172. Choline in Assisted Reproductive Technology: Boosting fetal brain development through maternal supplementation**

**Stephanie Tasselli Alencar da Assunção^1^, Marjorye Tasselli Alencar da Assunção^1^, Gabriel Monteiro Pinheiro^1^**

^1^Universidade Santo Amaro - São Paulo - SP - Brazil

**Objective:** To evaluate the crucial importance of choline in the context of pregnancy, specifically its fundamental role in fetal brain development and infant neurodevelopment.

**Methods:** For the present study, a systematic review method was adopted to assess the potential of maternal choline supplementation and investigate its possible impacts on fetal brain development. Articles indexed in the PubMed database, including reviews, meta-analyses, and clinical trials, dated between 2019 and 2024, in the English language, were selected. The following descriptors were used: "choline", "assisted reproduction", "pregnancy", and "neurodevelopment".

**Results:** Choline emerges as a crucial nutrient in the context of pregnancy, playing a fundamental role in fetal and infant brain development. Comprehensive reviews of studies, including meta-analyses and prospective studies, highlight significant benefits associated with adequate maternal choline intake during gestation. Studies in animal models have demonstrated that choline deficiencies can adversely impact cortical brain development, reduce neurogenesis, and negatively affect cognition and memory in offspring. Moreover, choline has been shown to mitigate the adverse effects of exposures such as alcohol during fetal development. In humans, research has revealed that maternal choline supplementation is associated with significant improvements in neurocognitive development in children, including benefits for visual memory, information processing speed, and reduction in attention problems. Studies suggest that choline may complement folate in preventing neural tube defects (NTDs), although the exact relationship between these nutrients still needs to be fully elucidated. Prospective studies have also indicated that higher choline intake during the second trimester of pregnancy is positively associated with child cognitive performance at 7 years of age, especially in visual memory skills. Proposed mechanisms include the formation of acetylcholine, which affects cholinergic transmission, and epigenetic changes in the fetal hippocampus, which may positively influence brain development. However, challenges persist in clinical and public health practice, as evidenced by inconsistencies in dietary choline intake during pregnancy. Longitudinal studies point out that even women with considered healthy eating habits often fail to meet daily choline recommendations, despite the presence of choline-rich foods such as eggs, meat, and dairy. Choline supplementation during pregnancy has been shown to effectively elevate maternal levels of choline and its metabolites, positively influencing lipoprotein biosynthesis and potentially improving lipid composition essential for fetal development. These findings underscore the importance of strategies to optimize choline intake during pregnancy, including consideration of prenatal supplements containing choline along with other essential nutrients such as folate.

**Conclusion:** In conclusion, choline emerges as a critical nutrient crucial for optimal fetal and infant brain development during pregnancy. Evidence from both animal models and human studies underscores its significant role in enhancing cognitive functions such as visual memory and information processing speed in children. Despite these promising findings, challenges persist regarding inconsistent dietary intake of choline among pregnant women, even those with ostensibly healthy eating habits. The potential benefits of maternal choline supplementation are evident, showing improvements in maternal choline levels and associated metabolites, which may positively impact fetal lipid metabolism and overall development. Moving forward, it is essential to prioritize strategies aimed at optimizing choline intake during pregnancy through education, dietary guidance, and potentially through the use of prenatal supplements that include choline alongside other essential nutrients like folate. By addressing these challenges and enhancing maternal choline intake, we can potentially improve neurodevelopmental outcomes for children and promote healthier pregnancies overall.

**P-173. Pregnancy rate after euploid embryo transfer in patients who underwent antibiotic therapy for chronic endometritis**

**Gérsia Araújo Viana^1^, Carlo Tantini^1^, Janaína Jardelha Mendes Maciel^1^, Ana Cláudia Moura Trigo^1^, Valentina Nascimento Cotrim^1^, Yara Viana Rodrigues Silveira^2^**

^1^CENAFERT - Salvador - BA - Brazil

^2^Hospital Municipal Dr. José de Carvalho Florence - São José dos Campos - SP - Brazil

**Objective:** The aim of this study was to evaluate the pregnancy rate in women diagnosed with chronic endometritis who underwent antibiotic therapy before the transfer of euploid embryos.

**Methods:** This retrospective study included 46 patients aged 31 to 42 who underwent in vitro fertilization with pre-implantation genetic testing at a private reproductive clinic, resulting in at least one euploid embryo. All participants underwent diagnostic hysteroscopy and endometrial biopsy for histopathological examination and immunohistochemical analysis using CD138. Patients diagnosed with chronic endometritis received antibiotic therapy. Embryo transfer was only performed after a follow-up endometrial biopsy confirmed the absence of endometritis.

**Results:** Among the 46 patients evaluated in this study, 13 were diagnosed with chronic endometritis, representing approximately 28.3% of the total. After initial antibiotic treatment, some patients required a second treatment regimen due to the persistence of endometritis in a subsequent biopsy. Embryo transfers were only performed after confirmation of a negative endometrial biopsy, ensuring the absence of endometritis. Of this group, 7 of the 13 treated patients, or 53.84%, achieved a clinical pregnancy.

**Conclusion:** The findings of our study suggest that effective treatment of chronic endometritis prior to euploid embryo transfer can normalize pregnancy outcomes to levels expected in infertile women receiving euploid embryos without a diagnosis of endometritis.

**P-174. Dual-dose Alfafolitropin for ovulation induction in ovarian stimulation Cycles for IVF: A case series**

**João Pedro Junqueira Caetano^1^, Luciana Campomizzi Calazans^1^, Herica Cristina Mendonça^1^, Isabela Mendonça Spina^2^, Erica Becker Sousa Xavier^1^, Ricardo Mello Marinho^1^**

^1^Huntington Pro Criar - Belo Horizonte - MG - Brazil

^2^Faculdade de Ciências Medicas de Minas Gerais - Belo Horizonte - MG - Brazil

**Objective:** This case series aims to evaluate the outcomes of ovulation induction in in vitro fertilization (IVF) cycles using dual doses of Alfafolitropin, including parameters such as the number of retrieved oocytes, number of metaphase II (MII) oocytes, number of Day 3 embryos, number of blastocysts per patient, and the duration of ovarian stimulation.

**Methods:** A retrospective study was conducted on IVF cycles using dual doses of Alfafolitropin for ovulation induction at a reproductive medicine center from August 2023 to April 2024. Ovulation induction was started on the 2nd or 3rd day of the menstrual cycle with an initial dose of Alfafolitropin (Elonva) 150 mcg. Ovarian monitoring was scheduled for the 6th or 7th day of induction, at which point a second dose of Alfafolitropin (Elonva) 100 mcg or 150 mcg was administered. Ovulation monitoring continued every 2-3 days to determine the optimal timing for triggering ovulation. Human chorionic gonadotropin (hCG) or a gonadotropin-releasing hormone (GnRH) agonist was administered for ovulation triggering based on follicular growth and hormone levels monitored during the cycle. Following ovulation induction, oocyte retrieval was performed under transvaginal ultrasound guidance approximately 36 hours after trigger administration. Subsequently, fertilization via intracytoplasmic sperm injection (ICSI) was conducted, followed by embryo culture and transfer as per standard clinical protocols. The following parameters were analyzed in this study: Baseline patient characteristics: age, antral follicle count (ACF) and cause of infertility; the total number of oocytes collected per patient; number of metaphase II stage (MII) oocytes per patient; number of day 3 Embryos and Blastocyst per patient.

**Results:** A total of 13 patients were included in this case series, with a mean age of 35.2 years old and an average antral follicle count of 15.1. Causes of infertility were categorized as follows: male factor (3 cases), endometriosis (1 case), tubal factor (2 cases), unexplained infertility (4 cases), advanced maternal age (3 cases). The average duration of ovarian stimulation was 11.4 days. On average, 12.1 oocytes were retrieved per patient, of which 7.1 were mature metaphase II (MII) oocytes. During embryo culture, patients produced an average of 4 high-quality Day 3 embryos and 2.3 blastocysts. In all cycles, pituitary suppression was achieved using progestins. None of the patients presented ovarian hyperstimulation syndrome, which could be a risk with the use of prolonged-duration gonadotropin.

**Conclusion:** The findings of this study align with expected standards in ovarian stimulation cycles for oocyte retrieval and in vitro fertilization. The use of Alfafolitropin reduces the number of injections required during stimulation and offers greater flexibility in ovulation monitoring, thereby enhancing treatment convenience and reducing stress levels for patients. Further comparative studies are warranted to validate these results and explore potential benefits in broader clinical settings.

**P-175. Evaluation of reproductive parameters in animal model of endometriosis**

**Luanna Prudencio de Araujo^1^, Michelle Guimarães dos Santos^1^, Caroline dos Santos da Fonseca^1^, Rafael Montenegro da Silva Lima^1^, Mariana Santos Costa^1^, Luiz Eurico Nasciutti^1^, Marcel Frajblat^1^**

^1^Universidade Federal do Rio de Janeiro - Rio de Janeiro - RJ - Brazil

**Objective:** Endometriosis is a chronic gynecological disease characterized by the presence of glandular and stromal-like uterine tissue outside the uterus, resulting in ectopic endometriotic lesions. The pelvic peritoneum and ovaries are the most common sites for these lesions, but they can also affect areas such as the intestine, cervix, vagina, and bladder. Processes like neoangiogenesis are necessary for nutritional supply, as well as the modulation of factors such as chemokines and interleukins, creating a pro-inflammatory microenvironment at the lesion site. Despite the well-established relationship between endometriosis and infertility in women, there is significant difficulty in understanding the exact mechanisms that impair fertility. Much of this is due to the multifactorial nature of endometriosis. This study aims to reproduce the relationship between endometriosis and fertility in an animal model through a surgery technique involving the suturing of uterine tissue grafts onto the peritoneum.

**Methods:** The C57BL6 isogenic mouse strain was used to avoid tissue rejection of the grafts. After the establishment of the lesion, confirmed by ultrasound after 30 days of the transplant, females from the Sham control group (n=7) and the induced endometriosis group (n=7) were paired with breeding males. Following observation of the vaginal plug to confirm mating, the pregnancy was monitored until day 15, when the animals were euthanized for analysis of the ovaries and gravid uteri. Parameters such as the number of fetuses, number of corpora lutea, ovulation rate, fertilization rate, implantation rate, and fetal resorption rate were evaluated. Histological analysis of the endometriotic lesions was performed to confirm the presence of stromal and glandular structures similar to the endometrium, and histological analysis of the uterus and ovaries was also conducted. This study was approved by the Animal Ethics Committee of UFRJ under protocol 144/23.

**Results:** The preliminary results indicate an increase in the percentage of fetal resorption in the endometriosis group (36.3%) compared to the control group (7.6%), indicating post-implantation fetal loss. Preliminary data also suggest no difference in the ovulation rate between the groups (9.5 and 8.5 corpora lutea for control and endometriosis, respectively) and no differences in the fertilization rate, which was 100% for both groups. In all animals with endometriosis, histology revealed the presence of stromal and glandular structures similar to the endometrium.

**Conclusion:** Although the results are preliminary and require further observations to establish final conclusions, there is a trend indicating a higher post-implantation gestational loss in females with endometriosis.

**P-176. Performing assisted hatching on day 3 facilitates herniation of biopsied cells but affects genetic outcomes?**

**Michelli Suemi Tanada^1^, Ivan Henrique Yoshida^1^, Caroline Zulim Berton^1^, Lívia Castro Onoda^1^, Rodrigo Angelo Souza^1^, Karla Pacheco Melo^1^, Priscila Carvalho do Ó^1^, Emerson Barchi Cordts^1^, Caio Parente Barbosa^1^**

^1^Instituto Ideia Fértil - Santo André - SP - Brazil

**Objective:** To compare the genetic-laboratory outcomes (euploidy, mosaicism, and types of chromosomal abnormalities) of embryonic biopsies performed with assisted hatching (AH) on Day 3 versus AH at the time of the procedure, in women of different age groups.

**Methods:** This retrospective study included 342 couples indicated for preimplantation genetic testing. The evaluated embryos were fertilized by intracytoplasmic sperm injection (ICSI) and cryopreserved by vitrification. A total of 1346 embryos met the inclusion criteria and were analyzed by NGS from January to December 2023, with the biopsy technique randomly chosen. The blastocysts were categorized into two groups based on when assisted hatching was performed: Group 1 had AH on Day 3 (1020 embryos), and Group 2 had AH from Day 5 onward, preceding biopsy at the expanded blastocyst stage (326 embryos). Culturing was done in K-Systems^®^ or EmbryoScope^®^ incubators using Global^®^ continuous medium. AH was performed using an infrared laser (ZILOS-tk^®^, Hamilton-Thorne Instruments Biosciences). LYKOS is a unique system where the laser is integrated into the objective lens, featuring a confocal laser with optical imaging and a 40× objective lens. AH performed on Day 3 can cause herniation of the inner cell mass (ICM), necessitating another ZP opening in a different region of the embryo. When performed at the time of biopsy, it can be a more time-consuming process, as it requires collapsing the cells to facilitate their removal. Several parameters between the two techniques were evaluated, such as general ploidy rate and by age group, as well as types of chromosomal abnormalities identified in preimplantation genetic testing. Statistical analysis involved three stages, including mixed-effects models to associate genetic responses with independent variables by age group.

**Results:** Comparing groups 1 and 2 under euploidy, we did not observe a difference between the groups (*p*=0.215). The rate of mosaicism and aneuploidys also showed no significant difference between the groups (*p*=0.696) and (*p*=0.283), respectively. For chromosomal abnormalities by technique used, Hatching on the Third Day of Embryonic Development showed a strong association between low-grade mosaic embryos (*p*=0.007); self-grade mosaics (*p*=0.001); monosomy (*p*<0.001); complex aneuploids (*p*<0.001); trisomies (*p*<0.001); triploidy (*p*=0.002); and chaotic embryos (*p*=0.010).

**Conclusion:** Although trophoectoderm (TE) biopsy is common, no standard protocol has been established for the timing of assisted hatching (AH) for embryonic biopsy. Moreover, little is known about the impact of this intervention on embryo ploidy. The method of ZP opening on Day 3 can cause early-stage cell herniation, necessitating further intervention. ZP opening at the expanded blastocyst stage can impede hatching in blastocysts with fewer cells. TE biopsy may be less invasive for expanded blastocysts, which have a higher total cell count than early-stage blastocysts, which have fewer cells. Despite the controversial efficacy of PGT-A, various factors, including diagnostic accuracy (sensitivity and specificity) and biopsy and tubing procedures, can affect outcomes. Two TE biopsy methods were used; however, a gold standard for TE biopsy has not yet been established. Biopsy of herniated TE samples simplifies and shortens the procedure, and an unassisted hatching protocol can complicate the approach if blastocysts collapse during the procedure. Some studies suggest that excess laser pulses can induce a higher frequency of mosaicism. Therefore, a quick movement within the minimum range and fewer laser pulses are recommended technically. Preliminary analyses did not identify associations between age, technique used, and ploidy alterations among the techniques employed. However, we observed a statistically significant difference in the types of genetic alterations depending on the technique used. The reason for this prevalence is not yet proven; further studies are needed for more conclusive findings.

**P-177. Impact of sperm capacitation techniques: Microfluidics and swim up in patients with high sperm DNA fragmentation**

**Rodrigo dos Santos Andrade^1^, Kahisa Natiele Fontana^1^, Tiago Cesar Mierzwa^1^, Lidio Jair Ribas Centa^1^, Mayara de Fatima Frazão Patussi^1^**

^1^ *Androlab - Clínica da Fertilidade - Curitiba - PR - Brazil*

**Objective:** To evaluate the superiority of the microfluidics technique compared to the conventional Swim Up technique for sperm capacitation in patients undergoing in vitro fertilization (IVF) treatments with high sperm DNA fragmentation and their outcomes, such as fertilization rate and blastulation rate.

**Methods:** A retrospective analysis of couples undergoing IVF cycles conducted at a private clinic in southern Brazil. Data collection was carried out through laboratory procedure records. Inclusion criteria for this study required patients to have previously undergone a sperm DNA fragmentation test with a result of 30% or more fragmented sperm. The study was divided into two groups: one where the semen sample was processed using the microfluidics technique and the other group where the preparation was carried out using the conventional Swim Up technique.

**Results:** The group with sperm capacitation performed using microfluidics showed a fertilization and blastulation rate of 70.7% and 42.7%, respectively. In contrast, the group prepared by Swim Up showed fertilization and blastulation rates of 72% and 53.8%, respectively.

**Conclusion:** The main indication for the use of the microfluidics technique is the high rate of sperm DNA fragmentation, and its use primarily improves embryonic development in assisted reproduction treatments. Our objective was to evaluate whether the use of this preparation is indeed superior to conventional preparation. Regarding the fertilization rate, the values did not present a statistically significant difference. However, in terms of the blastulation rate, there were more embryos available in the cycles performed with conventional Swim Up preparation, consequently increasing the chances of treatment success. Embryo quality and live birth rate were not evaluated in these groups due to the large number of cycles without fresh transfer due to freeze-all protocols, requiring further study to collect these data. This raises an important discussion about the need for individualized decision-making regarding the use of the microfluidics technique, especially considering the added cost to the treatment. This study also showed that the conventional Swim Up technique yields excellent results.

**P-178. MitoScore^®^ as a predictor of adverse reproductive outcomes in PGT-A cycles**

**Gustavo Nardini Cecchino^1,2^, Dóris Ferreira Moriyama^2^, Rodrigo da Rosa Filho^1,2^, Juliana Halley Hatty^1,2^, Rafael Portela^2^, Matheus Roque^1,2^**

^1^Mater Prime - São Paulo - SP - Brazil

^2^Mater Lab - São Paulo - SP - Brazil

**Objective:** Various studies have highlighted the significant role of mitochondrial function in reproductive success. MitoScore^®^, a measure of mitochondrial DNA content, has been proposed as a novel tool for embryo selection to enhance IVF outcomes. This study aims to determine if MitoScore^®^ can predict pregnancy rates (PR), clinical pregnancy rates (CPR), and ongoing pregnancy rates (OPR) in single euploid blastocyst transfers.

**Methods:** This retrospective study analyzed data from 178 single euploid embryo transfers at a private IVF center from January to November 2023. Patients were categorized into four quartiles based on their MitoScore^®^: Group 1 (<12.74; n=45), Group 2 (≥12.74 and <16.61; n=44), Group 3 (≥16.61 and <18.99; n=46), and Group 4 (≥18.99; n=43). Consistent endometrial preparation protocols were applied across all groups. Data dispersion and outlier values were addressed by this quartile classification. The primary outcome was OPR, with secondary outcomes including PR and CPR. Statistical analyses were performed using SPSS software, with Mann-Whitney U tests used to determine the significance of the results.

**Results:** The average age of participants was 36 years, with a mean MitoScore^®^ of 16.68. Overall, the PR was 74%, CPR was 69%, and OPR was 65%. There were no significant differences in demographic or cycle parameters across the groups. The median MitoScore^®^ of implanted embryos was 16.26 (range: 9.84-39.90), compared to 17.63 (range: 11.25-27.36) for non-implanted embryos, indicating a significant association with PR (*p*=0.046) and CPR (*p*=0.04). Group 4 exhibited significantly lower PR (56%), CPR (49%), and OPR (44%) compared to Groups 1 (PR 80%, *p*=0.015; CPR 73%, *p*=0.018; OPR 67%, *p*=0.034), 2 (PR 80%, *p*=0.018; CPR 80%, *p*=0.003; OPR 77%, p=0.002), and 3 (PR 80%, *p*=0.012; CPR 74%, *p*=0.015; OPR 72%, *p*=0.008). No significant differences in reproductive outcomes were observed between Groups 1, 2, and 3.

**Conclusion:** This study found that a MitoScore^®^ ≥18.99 is associated with significantly lower PR, CPR, and OPR following single euploid blastocyst transfers, despite comprising only 25% of cases. These findings suggest that while MitoScore^®^ can be a useful tool in embryo selection, high MitoScore^®^ levels may predict lower chances of successful outcomes. Limitations include the retrospective design and small sample size, which may affect the generalizability of the results. Further research is necessary to validate these findings and optimize the use of MitoScore^®^ in personalized IVF treatments.

**P-179. Severe male factor has no impact on blastulation rate in patients with good quality embryos at cleavage stage**

**Luiza da Silva Rodrigues^1^, Bruna Campos Galgaro^1^, João Sabino Lahorgue da Cunha Filho^1^**

^1^Centro de Reprodução Humana Insemine - Porto Alegre - RS - Brazil

**Objective:** To compare blastulation rate between conventional IVF, ICSI for non-severe male factor, and ICSI for severe male factor.

**Methods:** This retrospective analysis included fresh cycles performed from January 2023 to May 2024 in which all embryos underwent extended culture to the blastocyst stage. In our practice, only patients with at least three good quality embryos at cleavage stage or that undergo PGT qualify for extended culture. Patients were divided into three groups: (1) conventional IVF, (2) ICSI in couples with non-severe male factor, and (3) ICSI in couples with severe oligozoospermia (less than 5 million sperm per ml of ejaculate) or surgically retrieved sperm. The primary outcome of this study was blastulation rate. Age, AMH (ng/mL), number of oocytes retrieved, and fertilization rate were also assessed. Data were analyzed using the Kruskall-Wallis test, results are presented as [median (range), p value] and considered significant when p≤0.05.

**Results:** A total of 702 cleavage stage embryos (from 70 cycles) were included in the study: 73 embryos in the conventional IVF group, 534 in the non-severe male factor group, and 95 in the severe male factor group. Age did not differ between groups [35 (23-39) *vs*. 34 (24-44) *vs*. 35 (30-39), *p*=0.869)], as well as AMH [2.84(1.22-5.09) *vs*. 3.03 (0.53-16.0) *vs*. 2.80 (1.1-12.0), *p*=0.669)] and the number of oocytes retrieved [9 (4-18) *vs*. 14 (3-36) *vs*. 16 (2-34), *p*=0.190)]. There was no statistical difference in the fertilization rate [91,6% (63-100) *vs*. 89.4% (57-100) *vs.* 82.0% (60-100), *p*=0.512)], nor in the blastulation rate [71.4% (50-100) *vs*. 71.4% (17-100) *vs*. 63.8% (40-100), *p*=0.513)] between conventional IVF, non-severe male factor and severe male factor groups, respectively.

**Conclusion:** Our data indicates that severe oligozoospermia or epididymal/testicular retrieved sperm do not impair embryo development in patients with good quality embryos at cleavage stage.

**P-180. Optimizing *in vitro* maturation efficiency: Evaluating a stepwise protocol with C-type natriuretic peptide (CNP) supplementation**

**Nathalia Romeiro Silva^1^, Mariana Degli Esposti^1^, Thaisy Tino Dellaqua^1^, José Buratini^1^**

^1^Unesp - Botucatu - SP - Brazil

**Objective:** Despite its long history, in vitro maturation (IVM) has demonstrated lower efficiency in terms of cumulative live birth rate compared to IFV/ICSI following classical ovarian stimulation. However, the reduced burden and cost associated with IVM may justify further investment in studies aimed at enhancing its efficacy. Culture systems, utilizing a continuous 24-hour incubation in amphiregulin (AREG), neuregulin 1 (NRG1), and FSH-enriched medium have been recently proposed. Nonetheless, a stepwise maturation process, as opposed to a continuous (single-step) approach, holds potential to enhance cumulus-oocyte communication and nuclear-cytoplasmic synchrony. This advantage stems from the ability to tailor culture media compositions appropriately at each stage. The potential benefits of this approach have prompted investigations into culture protocols involving agents that can modulate nuclear maturation dynamics. Therefore, the objective of the present study was to evaluate the efficacy of a stepwise IVM approach associated with CNP supplementation, focusing on its ability to optimize the oocyte maturation rate and enhance oocyte quality.

**Methods:** Bovine cumulus-oocyte complexes (COCs) were aspirated from 2-8 mm follicles obtained from abattoir ovaries and allocated into treatment groups as follows: (i) Control Group (n=25): underwent continuous 24-hour incubation in high FSH-enriched medium; (ii) Stepwise Group (n=25): incubation in medium containing FSH, IGF1, 17β-estradiol, and progesterone in physiological concentrations for 6h, followed by supplementation with AREG and NRG1 for additional 16 hours; and (iii) CNP Group (n=25): identical to the Stepwise Group except for the addition of C-type natriuretic peptide (CNP) during the first 6h. The experiment was conducted in triplicates, and COCs were evaluated after 8, 10, and 22 hours of incubation. Following denudation, COCs were fixed in methanol, stained with Hoechst, and analyzed using epifluorescence microscopy. Genetic material was classified as germinal vesicle (GV), germinal vesicle breakdown (GVBD), metaphase I (MI), metaphase II (MII), or degenerate (DEG).

**Results:** After 8 hours of culture, significant differences were observed in the GVBD rates among the experimental groups. The Stepwise Group exhibited the highest rate (69.61±14.19%), followed by the Control Group (46.43±0.77%), whereas the CNP Group showed the lowest rate (26.75±5.60%, *p*=0.049). However, after 10 hours of incubation, although a significant difference in the GVBD rate was noted between the Control Group and both sequential IVM groups (Stepwise and CNP Groups), no difference was observed between the Stepwise and CNP Groups (Control: 70.32±9.17%; Stepwise Group: 92.42±0.56%; CNP Group: 94.33±3.03%; *p*=0.0414). Intriguingly, MII rates did not differ significantly among treatments after 22 hours of cultivation (FSH: 70.08±9.11%; Stepwise: 80.33±6.43%; Stepwise+CNP: 80.34±1.31%, *p*=0.5070).

**Conclusion:** The addition of CNP during the initial phase of sequential IVM culture effectively maintains oocytes in meiotic arrest during the early hours of maturation, potentially enhancing nuclear-cytoplasmic synchrony. Despite this initial delay, all treatments achieved comparable MII rates after 22 hours of cultivation, demonstrating that sequential stimulation does not hinder the overall maturation process. Therefore, the sequential stimulation approach, particularly with CNP supplementation, holds promise for improving the physiological relevance and efficiency of IVM protocols, enhancing oocyte developmental competence.

**P-181. Developmental potential of mature and immature oocytes in IVF treatments**

**Gustavo Nardini Cecchino^1,2^, Dóris Ferreira Moriyama^2^, Rafael Portela^2^, Matheus Roque^1,2^**

^1^Mater Prime - São Paulo - SP - Brazil

^2^Mater Lab - São Paulo - SP - Brazil

**Objective:** Traditionally, immature oocytes (MI) are often discarded during in vitro fertilization (IVF) procedures due to their perceived low developmental potential. However, in the context of IVF, each additional oocyte can be crucial for improving reproductive outcomes. This study aimed to compare the developmental potential of MI oocytes with mature (MII) oocytes in the same cohort of patients undergoing intracytoplasmic sperm injection (ICSI) at a private IVF lab.

**Methods:** A retrospective self-controlled case series study was conducted, involving 140 patients undergoing preimplantation genetic testing for aneuploidy (PGT-A) cycles at a single center between January 2022 and December 2023. Each patient had both MI and MII oocytes injected via ICSI. The development of MI oocytes was monitored following fertilization, and embryos derived from MI and MII oocytes were cultured separately until the blastocyst stage. Descriptive statistics and paired sample tests were performed using SPSS software.

**Results:** In the analysis of 140 cycles, 1,283 MII oocytes and 299 MI oocytes were retrieved. The mean age of the patients was 37.5 years. The fertilization rate for MI oocytes was 73.5%, compared to 83% for MII oocytes (*p*=0.116). A total of 175 blastocysts were derived from MI oocytes (130 biopsied), while 630 blastocysts came from MII oocytes (402 biopsied). The euploidy rates were 39% for MI-derived blastocysts *versus* 44% for MII-derived blastocysts (*p*=0.567). Notably, only strictly MI oocytes were included in this analysis, excluding those that progressed to MII before ICSI and/or rescued in vitro matured MII oocytes.

**Conclusion:** This study challenges the conventional practice of discarding MI oocytes due to their perceived low developmental potential and the strategy of waiting for MI to progress to MII before ICSI. It demonstrates that MI and MII oocytes have comparable developmental outcomes in patients undergoing ICSI. Utilizing MI oocytes in IVF cycles could significantly expand the pool of viable euploid embryos, potentially enhancing reproductive success rates and offering new hope to patients seeking fertility treatments. However, it is important to recognize that the retrospective design may introduce biases, and key reproductive outcomes such as implantation, pregnancy, and miscarriage rates were not assessed. Further validation across different laboratories is crucial for broader applicability of these findings.

